# Comparison of ^18^F-FDG PET/CT and DWI for detection of mediastinal nodal metastasis in non-small cell lung cancer: A meta-analysis

**DOI:** 10.1371/journal.pone.0173104

**Published:** 2017-03-02

**Authors:** Guohua Shen, You Lan, Kan Zhang, Pengwei Ren, Zhiyun Jia

**Affiliations:** 1 Department of Nuclear Medicine, West China Hospital, Sichuan University, Chengdu, Sichuan, People’s Republic of China; 2 Division of Laboratory Medicine, West China School of Medicine, Sichuan University, Chengdu, Sichuan, People’s Republic of China; 3 Department of Evidence-Based Medicine and Clinical Epidemiology, West China Hospital, Sichuan University, Chengdu, Sichuan, People’s Republic of China; Northwestern University Feinberg School of Medicine, UNITED STATES

## Abstract

**Background:**

Accurate clinical staging of mediastinal lymph nodes of patients with lung cancer is important in determining therapeutic options and prognoses. We aimed to compare the diagnostic performance of diffusion-weighted magnetic resonance imaging (DWI) and ^18^F-fluorodeoxyglucose positron emission tomography/computed tomography (^18^F-FDG PET/CT) in detecting mediastinal nodal metastasis of lung cancer.

**Methods:**

Relevant studies were systematically searched in the MEDLINE, EMBASE, PUBMED, and Cochrane Library databases. Based on extracted data, the pooled sensitivity, specificity, positive and negative likelihood ratios (PLR and NLR) with individual 95% confidence intervals were calculated. In addition, the publication bias was assessed by Deek’s funnel plot of the asymmetry test. The potential heterogeneity was explored by threshold effect analysis and subgroup analyses.

**Results:**

Forty-three studies were finally included. For PET/CT, the pooled sensitivity and specificity were 0.65 (0.63–0.67) and 0.93 (0.93–0.94), respectively. The corresponding values of DWI were 0.72 (0.68–0.76) and 0.97 (0.96–0.98), respectively. The overall PLR and NLR of DWI were 13.15 (5.98–28.89) and 0.32 (0.27–0.39), respectively. For PET/CT, the corresponding values were 8.46 (6.54–10.96) and 0.38 (0.33–0.45), respectively. The Deek’s test revealed no significant publication bias. Study design and patient enrollment were potential causes for the heterogeneity of DWI studies and the threshold was a potential source for PET/CT studies.

**Conclusion:**

Both modalities are beneficial in detecting lymph nodes metastases in lung cancer without significant differences between them. DWI might be an alternative modality for evaluating nodal status of NSCLC.

## Introduction

Lung cancer is the leading cause of all cancer-related deaths worldwide [1]. Non-small-cell cancer (NSCLC) is the main type of lung cancer, accounting for 80% of all cases. NSCLC typically metastasizes to the hilar and mediastinal lymph nodes (MLNs), and metastasis is a very important prognostic factor. The 5-year survival rates are 54.0% for patients without any metastases and 26.5% for subjects with MLNs metastases [2]. The selected treatment, such as surgery, radiotherapy and chemotherapy, is mainly dependent on the TNM staging. Therefore, accurate assessment of MLNs is necessary for TNM staging and optimal treatment selection.

Various diagnostic techniques, such as computed tomography (CT), positron emission tomography (PET), PET/CT, mediastinoscopy, and magnetic resonance imaging (MRI), are used for nodal staging assessment of NSCLC. CT is most widely used to assess the nodal status of lung cancer based on lymph node size, although lymph node size is not reliable for the evaluation of metastatic involvement [3]. FDG PET, a functional imaging modality, could detect potential tumor activity and facilitate earlier recognition of metastases [4]; however, this method has been limited by the low spatial resolution of stand-alone PET images [5]. Integrated PET/CT, which combines the anatomical detail and functional statue, is now commonly used for NSCLC staging.

Diffusion weighted imaging (DWI), an MRI technique, could detect the restricted diffusion of water molecules among tissues at the cellular level, which could be measured by apparent diffusion coefficient (ADC) value [5]. DWI and ADC values have been widely used in brain imaging for the evaluation of acute ischemic stroke, intracranial tumors and demyelinating disease [6]. However, DWI is highly sensitive to motion artifacts caused by breathing and movement of the heart and aorta, resulting in its limited application [7]. Recently, the rapid development of MRI techniques, such as echo-planar imaging sequence, multichannel coils and parallel imaging, has allowed for the application of DWI in anatomical regions prone to motion artifacts, such as the mediastinum [8]. Several studies have shown that diagnostic accuracy of DWI for nodal assessment in the mediastinum is 76–95% [9–13].

To our knowledge, the performance of DWI and FDG PET/CT in nodal staging has yet to be determined. Some studies validated the potential of DWI for N stage assessment and the characterization of mediastinal lymph nodes in patients with NSCLC with a capability similar to that of ^18^F-FDG PET/CT [14]. Some studies showed advantages of DWI over FDG PET/CT [4, 5], whereas other studies showed that DWI had lower capability than FDG PET/CT [8, 11]. Therefore, we performed a meta-analysis to compare the diagnostic performance of DWI and FDG PET/CT in lymph node staging in patients with NSCLC.

## Materials and methods

### Search strategy

An extensive search of the available literature, published from January 2001 to December 2014, was performed in the MEDLINE, EMBASE, PUBMED and Cochrane Library databases. The combination of keywords was as follows: (‘DW-MRI’ OR ‘diffusion-weighted magnetic resonance imaging’) AND (‘FDG’ OR ‘18F-FDG’ OR ‘FDG-F18’ OR ‘fluorodeoxyglucose’ OR ‘PET/CT’ OR ‘positron emission tomography/computed tomography’ OR ‘PET-CT’ OR ‘positron emission tomography-computed tomography’) AND (‘lung cancer’ OR ‘lung neoplasm’) AND (‘lymph node metastasis’ OR ‘lymphatic metastasis’) AND (‘specificity’ OR ‘sensitivity’ OR ‘false-positive’ OR ‘false-negative’ OR ‘detection’ OR ‘diagnosis’ OR ‘accuracy’).

### Inclusion and exclusion criteria

The inclusion criteria were as follows: (i) the diagnostic performances of ^18^F-FDG PET/CT or DWI in detecting nodal metastases in lung cancer were identified in the literature; (ii) pathological analysis, surgical biopsy, mediastinoscopy or follow-up results were used as the gold standard of diagnosis; (iii) the values of true positive (TN), false positive (FP), false negative (FN) and true negative (TN) depending on the original data could be obtained in the literature; (iv) the studies were based on a per-lesion analysis; and (v) the article with the most details or the most recent article was selected when similar data appeared in more than one article.

The exclusion criteria were as follows: (i) studies that focused on the therapy response or prognosis rather than on disease diagnoses; (ii) studies regarding mediastinal tumor or pleural diseases except for lung cancer; (iii) case reports, meeting abstracts, reviews, letters, comments, animal experiments, or the studies with less than 10 samples.

### Data extraction

The following information was extracted from the included studies: the first author, year of publication, study design (prospective or retrospective), country of the study, patient enrollment, technique characteristics, reference standard, and blinding method. The TP, FP, TN, and FN results were also extracted.

Two reviewers independently extracted the relevant data from each study. Any disagreements were resolved by discussion with a third reviewer.

### Statistical analysis

For lesion-based analyses, we obtained the pooled sensitivities and specificities of PET/CT and DWI, as well as their 95% confidence intervals using the weighted average method. We also calculated the pooled positive and negative likelihood ratios (PLR and NLR) with their 95% confidence intervals. The data were finally summarized in receiver-operating characteristic curves (SROC), with the area under the curve (AUC) and the Q* index obtained.

We used the I^2^ index for heterogeneity assessment. If the I^2^ index was higher than 50%, a random effect model was used; otherwise, a fixed model was used. In this study, we used the random-effect model to pool estimates. To explore the sources of heterogeneity, we performed subgroup analyses based on factors such as sample size (≥ 250 vs. <250), study design (retrospective vs. prospective), country (Asia vs. non- Asia), subject enrollment (consecutive vs. nonconsecutive), and analysis method (qualitative, quantitative, or both). The threshold effect analysis was also performed, and the publication bias was examined by Deek’s funnel plot.

The statistical computations were performed using Stata software version 12.0 (StataCorp LP, Texas, USA) and MetaDisc version 1.4 (Unit of Clinical Biostatistics, Ramóny Cajal Hospital, Madrid, Spain). For *P* value, the level of statistical significance was set to 5%.

## Results

### Study selection and description

**Fig 1:**
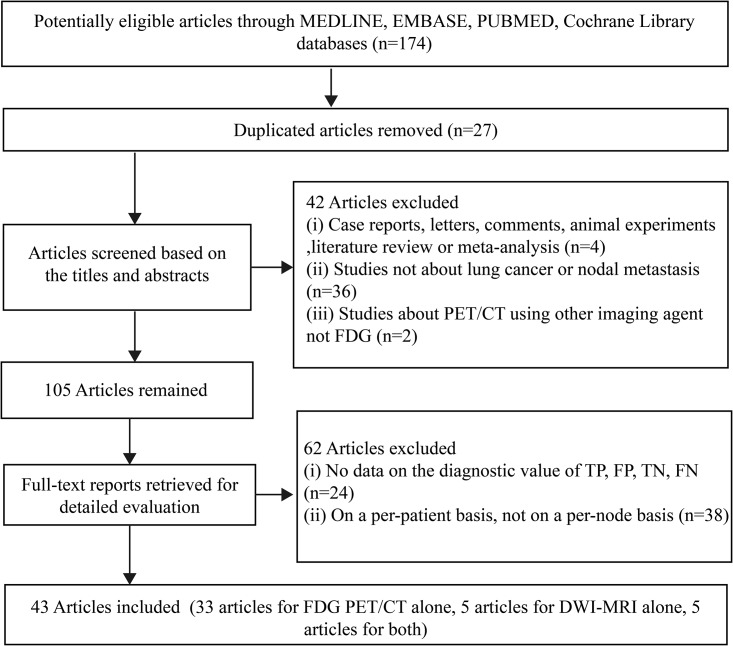
Flow chart of studies identified and included in the present meta-analysis.

A total of 174 articles were screened in the primary literature search, and 43 articles (in total 48 studies, 10 studies for DWI and 38 studies for ^18^F-FDG PET/CT) were included based on the inclusion and exclusion criteria. A flowchart depicting the study selection is shown in Fig 1.

The principal characteristics of the 43 selected articles [5, 9, 10, 12, 15–53] involving a total of 21,058 lymph nodes are listed in Table 1. Of these articles, 27 [15–18, 20–22, 24, 27–29, 31–35, 37, 41–43, 46, 47, 49–53] were retrospective, and 16 [5, 9, 10, 12, 19, 23, 25, 26, 30, 36, 38–40, 44, 45, 48] were prospective. Patients in 26 [5, 9, 10, 12, 15–20, 22, 23, 25, 26, 28, 29, 31, 32, 36, 38–40, 43, 44, 46, 47] articles were enrolled in a consecutive manner while the other 17 [21, 24, 27, 30, 33–35, 37, 41, 42, 45, 48–53] articles did not. In 29 articles [5, 9, 10, 12, 16–20, 22, 23, 25–28, 32, 35–38, 40, 44–50, 52], the DWI or ^18^F-FDG PET/CT reviewers were blinded to the histologic findings and clinical data; the remaining 14 articles [15, 21, 24, 29–31, 33, 34, 39, 41–43, 51, 53] did not specify whether the reviewers were blinded. Thirty-three articles [5, 9, 10, 12, 16, 21–26, 28–41, 43, 47–53] enrolled Asian patients. The majority of DWI studies were conducted under a magnetic field strength of 1.5 T, and the majority of PET scanning studies used an integrated PET/CT technique. The high variability regarding principal characteristics was observed between included studies.

**Table 1:** The principal characteristics of included studies.

First author/year	Study design	Country	Consecutive	Mean age	No. of patients and lesions	Blind	Technique characteristics	TP	FP	FN	TN	Reference standard	Analysis method
*DWI*													
Zhang/2013	R	China	ND	59	25/78	Y	3.0 T SE-EPI (0,800)	29	13	6	30	HP	QN
He/2011	R	China	ND	58	12/56	ND	1.5T ASSET/STIR/SE-EPI (0,500)	18	4	16	18	HP	QN
Usuda/2011	P	Japan	C	68	63/319	Y	1.5 T SS-EPI (0,800)	33	3	11	272	HP	QN
Zeng/2012	R	China	ND	58	45/68	Y	1.5 T SE-EPI (600,800,1000)	23	3	9	33	HP	QN
Ohno/2011	P	Japan	C	73	250/270	Y	1.5 T STIR-EPI (0,1000)	101	17	34	118	HP	QN
Nakayama/2010	R	Japan	ND	68	70/56	Y	1.5 T SS-SE-EPI (50,1000)	19	5	4	28	HP	QN
Nomori /2008	P	Japan	C	70	88/734	Y	1.5 T SE-EPI (0,1000)	24	5	12	693	HP	QN
Xu/2014	P	China	C	55	42/119	Y	1.5 T SS-SE-EPI (0,1000)	29	7	6	77	HP	QN
Usuda/2013	P	Japan	C	68	158/705	Y	1.5 T SS-EPI (0,800)	39	5	22	639	HP	QN
Kim/2012	P	Korea	C	62	49/206	Y	1.5 T SS-EPI (0,100,700)	26	6	13	161	HP	QN
*PET/CT*													
Al-Sarraf, Nael/2008	R	Ireland	C	64.5	206/1145	ND	PET-CT (Discovery ST, GE Medical systems).370MBq	75	27	93	950	HP	QN
An, Y. S/2008	R	South Korea	C	63	124/396	Y	PET-CT (Discovery ST Scanner, GE Healthcare, Milwaukee, WI, USA) 370MBq	62	87	19	228	HP	QN
Billé, Andrea/2009	R	Italy	C	67	159/1001	Y	PET/CT scanner (Discovery ST; GE Medical systems) 4.5–5.5 MBq/kg	41	14	30	916	HP	QL
Booth, K./2013	R	England	C	65	64/200	Y	GE Discovery LS fusion PET/CT scanner 375 MBq	7	8	11	174	HP	QN/QL/ND
Bryant, Ayesha S/2006	P	England	C	67	143/1252	Y	PET-CT scanner (GE Discovery LS, Milwaukee, WI). 555 MBq	120	67	34	1031	HP	QN
Hellwig, Dirk/2015	R	Germany	C	62	80/311	Y	ECAT ART scanner (Siemens Medical Solutions), 250±2 MBq	62	39	8	202	HP	QL
Hu, M/2008	R	China	ND	50	46/584	ND	PET-CT scanner 7.4 MBq/kg	117	72	17	378	HP	QN
Jeon, Tae Yeon/2010	R	Korea	C	65	168/617	Y	PET/CT device (Discovery LS, GE Healthcare) 370MBq	30	10	30	547	HP	QL
Kim, Byung-Tae/2006	P	Korea	C	59	150/568	Y	PET/CT device (Discovery LS, GE Medical Systems) 370MBq	23	0	32	513	HP	QL
Kim, D. W./2012	R	Korea	ND	68.4	69/268	ND	PET/CT (Biograph Sensation 16, Siemens Medical Systems) 4.0 MBq/kg	157	8	52	51	HP+CFU	QN
Kim, Yoon Kyung/2007	P	Korea	C	61	674/2477	Y	PET/CT device (Discovery LS, GE Healthcare, Milwaukee, WI) 370 MBq	126	48	149	2154	HP	QL
Kim, Y. N./2012	P	Korea	C	62	49/206	Y	PET/CT device (Discovery STE, GE Healthcare, Milwaukee, WI, USA) 370 MBq	18	6	21	161	HP	QL
Koksal, Deniz/2013	R	Turkey	ND	59.8	81/334	Y	PET/CT scanner (Siemens, Biograph-6- True Point) 145 μCi/kg	14	86	8	226	HP	QL
Kuo, W. H./2012	R	Taiwan	C	63.1	102/118	Y	PET/CT scanner Discovery ST16 scanner (GE Medical Systems, Milwaukee, WI), 370 to 555 MBq	12	25	9	72	HP	QL
Lee, A. Y./2014	R	Korea	C	64.5	104/372	ND	PET/CT scanner (Discovery STE, GE Healthcare, Milwaukee, WI, USA), 370 MBq	23	31	26	292	HP	QN
Lee, Jeong Won/2009	P	Korea	ND	60.7	182/778	ND	a Gemini PET/CT system (Philips, Milpitas). 5.18 MBq/kg	40	109	13	616	HP	QL
Lee, S. M./2012	R	Korea	C	60.0	160/756	ND	Gemini PET/CT (Philips Medical Systems, Cleveland, OH, USA) 5.2 MBq/kg	2	43	13	698	HP	QN
Li, Meng/2012	R	China	C	58	80/265	Y	PET—CT device (GE Discovery ST 16), 3.70–4.44 MBq/kg	33	7	18	207	HP	QN
Li, Xiaolin/2011	R	China	ND	60	200/1132	ND	PET/CT scanner (GE Discovery LS, ST, or DST) 5.55–7.40 MBq/kg	27	60	13	1032	HP	QN
Lin, W. Y./2012	R	Taiwan	ND	66	83/364	ND	PET-CT scanner (Discovery VCT; GE Healthcare,Waukesha, Wisconsin, USA), 370 MBq	18	50	20	276	HP	QN
Liu, Bao-jun/2009	R	China	ND	57.5	39/208	Y	PET/CT scanner (Siemens Biograph Sensation 16, Siemens, Germany) 7.4MBq/kg	40	24	26	120	HP	QN/QL
Morikawa, Miwa/2009	P	Japan	C	66.1	93/137	Y	PET/CT scanner (Discovery LS; GE Healthcare). 185 MBq	74	19	8	36	HP	QN
Nomori, H./2008	P	Japan	C	70	88/734	ND	PET-CT device (Discovery ST; GE Medical Systems), 3.7 MBq/kg	26	18	10	680	HP	QN
Ohno, Y./2007	P	Japan	C	68	115/891	ND	PET scanner (ALLEGRO; Philips)+ CT scanner, Aquilion 16 (Toshiba Medical Systems, Ohtawara, Japan), 4.44 MBq/kg	60	31	13	787	HP	QN
Shim, Sung Shine/2005	P	Korea	C	56	106/393	Y	PET/CT device (Discovery LS; GE Medical Systems, Milwaukee, Wis), 370 MBq	28	58	5	302	HP	QL
Sit, Alva KY/2010	R	China	ND	61	107/249	ND	PET/CT scanner, ND	18	31	34	166	HP	QN
Ohno, Y./2011	P	Japan	C	73	250/270	Y	PET/CT scanner (Discovery ST; GE Healthcare, Milwaukee, Wis). 3.3 MBq/kg	102	15	33	120	HP	QN
Tasci, Erdal/2010	R	Turkey	ND	58.2	127/826	ND	on a Biograph PET/CT (Siemens/CTI) scanner, 555MBq	41	50	24	711	HP	QL
Toba, H./2010	R	Japan	C	68.0	42/217	ND	PET/CT scanner Aquiduo (Toshiba Medical Systems, Tokyo, Japan)	17	15	4	181	HP	QL
Tournoy, KG/2007	P	Belgium	C	68	52/105	Y	FDG-PET/CT scanner (Philips Gemini FDG-PET/CT, Philips Medical Systems, Cleveland, Ohio, USA), 4 MBq/kg	32	10	6	57	HP	QN
Usuda, Katsuo/2013	P	Japan	C	68	158/705	Y	PET-CT (SIEMENS Biography Sensation 16, Erlangenm Germany), 3.7 MBq/Kg	24	3	37	641	HP	QN
Ventura, Elisa/2010	R	USA	C	66.32	31/90	Y	PET (CTI Molecular Imaging, Knoxville, TN, USA)+PET/CT Siemens Molecular Imaging, Knoxville, TN, USA), 555-740MBq	38	20	3	29	HP	QL
Xu, N/2014	R	China	C	61	101/528	Y	PET/CT scanner, 4.5–5.5 MBq/kg	52	18	49	409	HP	QL
Usuda, Katsuo/2011	P	Japan	C	68	63/319	Y	PET/CT scanner (Siemens Biography Sensation 16), 185 MBq	21	9	23	266	HP	QN
Yang, Wenfeng/2009	P	China	ND	69	122/639	Y	PET/CT system (Discovery LS; GE Healthcare), 370 MBq	132	73	21	413	HP	QL
Yi, Chin A/2007	R	Korea	N	60	143/453	Y	PET/CT device (Discovery LS, GE Healthcare), 370 MBq	22	4	28	399	HP	QN
Vansteenkiste, Johan F/1998	P	Belgium	ND	62	56/493	Y	PET scanner (CTI-Siemens 931/08/12), 6.5 MBq/kg	38	21	22	412	HP	QL
Zhou,YF/2014	R	China	ND	60	64/280	ND	PET/CT scanner (Philips Gemini TF 16), 2.96MBq/kg	25	9	9	237	HP	QN/QL

ND: no documented; No.: number; TP: true positive; FP: false positive; FN: false negative; TN: true negative. P: prospective; R: retrospective; Y: yes; QL: qualitative analysis; QN: quantitative analysis; HP: histopathology; C: consecutive

### Quality assessment

**Fig 2:**
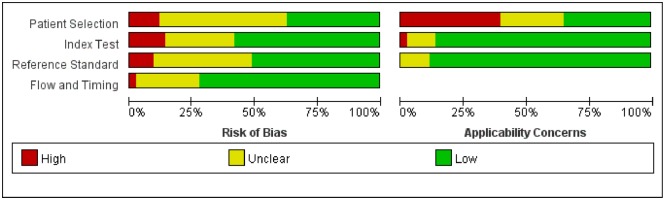
Proportion of studies with low, high and unclear risks of bias and applicability concerns. Review authors’ judgments about each domain presented as percentage across included studies.

We used QUADAS-2 to analyze the quality of the studies [54]. The methodological results are displayed in Fig 2. Participant selection was judged to be at low risk of bias in 16 of the studies and at high or unclear risk of bias in the remaining 27 studies. The majority of selected studies did not provide information regarding consecutive enrollment and did not avoid a case-control design. These inclusion restrictions artificially narrowed the range of patients who would undergo PET/CT in standard practice, which gave rise to a high concern about the applicability of these studies. For the index test and reference standard, common weaknesses focused on the fact that a blinding method was not provided or used when interpreting the results. With regard to the flow and timing, 12 articles displayed unclear or high risk because they lacked an explicit description of the time interval between the index test and reference standard. In a word, a substantial amount of underreporting in the included studies resulted in “unclear” or “high” bias or concern, hampering the methodological quality.

### Diagnostic accuracy of DWI and FDG-PET/CT

**Fig 3:**
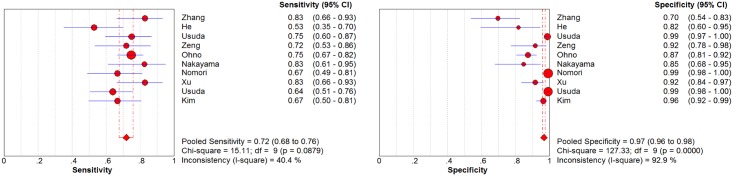
Forest plot of sensitivity and specificity for DWI. Each solid circle represents sensitivity and specificity of individual studies, and the size of the circle indicates the study size. The diamond means the pooled sensitivity and specificity of all 10 studies.

**Fig 4:**
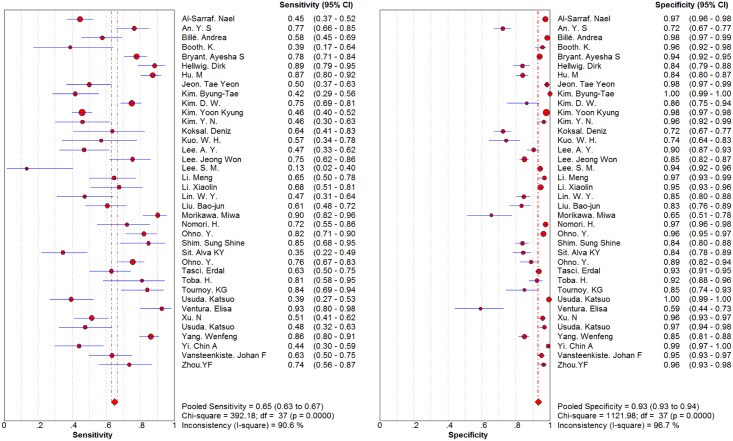
Forest plot of sensitivity and specificity for PET/CT. Each solid circle represents sensitivity and specificity of individual studies, and the size of the circle indicates the study size. The diamond means the pooled sensitivity and specificity of all 38 studies.

The pooled results are shown in Figs 3 and 4. Based on 10 studies, DWI had a sensitivity of 0.72 (0.68–0.76) and a specificity of 0.97 (0.96–0.98). In 33 studies, PET/CT achieved a sensitivity and specificity of 0.65 (0.63–0.67) and 0.93 (0.93–0.94), respectively. The LR syntheses gave an overall PLR of 13.15 (5.98–28.89) and NLR of 0.32 (0.27–0.39) for DWI. For ^18^F-FDG PET/CT, the overall PLR was 8.46 (6.54–10.96), and the NLR was 0.38 (0.33–0.45). The DOR was 46.11 (19.89–106.89) for DWI and 25.18 (18.58–34.13) for ^18^F-FDG PET/CT.

**Fig 5:**
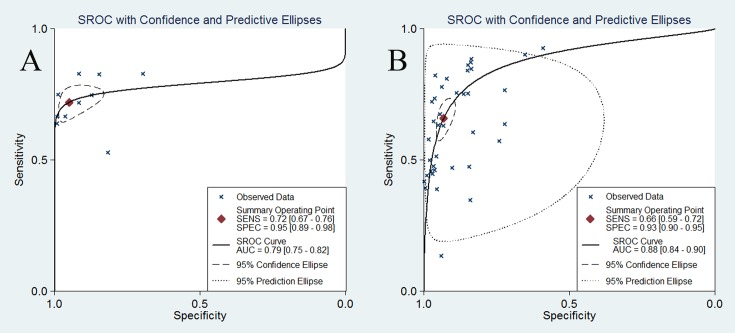
SROC curve of DWI (A) and 18F-FDG PET/CT (B) in detecting mediastinal nodal metastases in patients with NSCLC. Each x represents individual study estimates. The diamond is the summary point representing the average sensitivity and specificity estimates. The ellipses around this summary point are the 95% confidence region (dashed line) and the 95% prediction region (dotted line).

No differences were found between the pooled specificity, sensitivity, PLR and NLR between DWI and FDG-PET/CT (*P* > 0.05). Using a fitted SROC curve, the overall AUCs for DWI and FDG-PET/CT were 0.79 and 0.88, respectively (Fig 5). For nodal staging of NSCLC, the diagnostic capacities of these two modalities were not significantly different. However, based on the PLR and NLR, a positive finding of DWI can diagnose the malignancy while a negative DWI finding alone might not exclude the malignancy. With regard to PET/CT, it can neither rule in nor rule out the disease.

### Heterogeneity analysis

**Fig 6:**
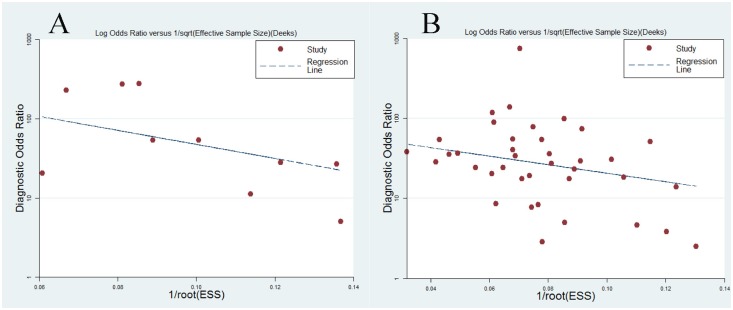
Funnel plot of publication bias for DWI (A) and 18F-FDG PET/CT (B). Each circle represents individual study. The dashed line means the regression line.

Our analysis revealed strong heterogeneity in sensitivity and specificity among the studies (*P* < 0.05, I^2^ > 90%). The Spearman rank correlation test indicated an absence of threshold effect in the DWI studies (coefficient = 0.364, *P* = 0.301) and showed a significant threshold effect in the PET/CT studies (coefficient = 0.556, *P* = 0.001). The threshold effect of PET/CT might arise from different cutoff values of SUV to differentiate malignant lesions from benign ones between included studies. Because of the small sample size of the DWI studies, we only performed subgroup analyses based on the sample size, study design and patient enrollment. Six studies using prospective design showed higher specificity (0.98 vs. 0.81, *P* < 0.05), and studies with consecutive enrollment showed higher specificity for nodal staging (0.98 vs. 0.81, *P* < 0.05). With regard to PET/CT studies, more factors including sample size, study design, country, patient enrollment, blinding method, and analysis method were explored in subgroup analyses; however, all these factors failed to explain the heterogeneity (*P* > 0.05). The results of the subgroup analyses are presented in Table 2. Deek’s funnel plot asymmetry tests indicated no significant publication bias (*P* = 0.277 for DWI and *P* = 0.098 for PET/CT) (Fig 6).

**Table 2:** The results of subgroup analysis for DWI and PET/CT.

Factors	No.of studies	Sensitivity (95%CI)	Specificity (95%)
**DWI**			
Sample size			
< 250	6	0.73 (0.66–0.79)	0.90 (0.87–0.93)
≥ 250	4	0.71 (0.66–0.77)	0.98 (0.98–0.99)
Study design*			
Prospective	6	0.72 (0.67–0.77)	0.98 (0.97–0.98)
Retrospective	4	0.72 (0.63–0.79)	0.81 (0.74–0.88)
Consecutive enrollment*			
Yes	6	0.72 (0.67–0.77)	0.98 (0.97–0.98)
No/Unclear	4	0.72 (0.63–0.79)	0.81 (0.74–0.88)
**PET/CT**			
Sample size			
< 250	9	0.68 (0.63–0.72)	0.86 (0.84–0.88)
≥ 250	29	0.64 (0.63–0.66)	0.94 (0.93–0.94)
Study design			
Prospective	15	0.67 (0.64–0.69)	0.94 (0.94–0.95)
Retrospective	23	0.63 (0.61–0.66)	0.92 (0.91–0.93)
Country			
non-Asia	10	0.66 (0.63–0.70)	0.93 (0.92–0.94)
Asia	28	0.64 (0.62–0.67)	0.93 (0.93–0.94)
Consecutive enrollment			
Yes	26	0.64 (0.61–0.66)	0.95 (0.94–0.95)
No/Unclear	12	0.68 (0.65–0.71)	0.90 (0.89–0.91)
Blind			
Yes	24	0.65 (0.62–0.67)	0.93 (0.93–0.94)
No/Unclear	14	0.65 (0.62–0.68)	0.93 (0.92–0.93)
Analysis method			
QN	19	0.67 (0.65–0.69)	0.93 (0.93–0.94)
QL	16	0.62 (0.60–0.65)	0.93 (0.92–0.94)
QN+QL	3	0.61 (0.52–0.70)	0.93 (0.90–0.95)

ND: no document; No.: number; QN: quantitative; QL: qualitative.

*There is significant difference between these subgroups.

## Discussion

Because integrated PET/CT directly combines PET data on metabolic changes with highly detailed anatomic CT information, this technique could detect lesions earlier and provide more precise location information than CT or PET alone [55]. DWI is a magnetic resonance imaging (MRI) technique based on the imaging of the molecular mobility of water [56]. Using this technique, the diagnoses of prostate cancer [57], urinary bladder cancer [58], uterine cancer [59] and rectal cancer [60] have shown promising results. Recently, some people have demonstrated that DWI could be used for the detection of mediastinal nodal metastases in lung cancer, but the diagnostic value of DWI for lung cancer has not yet been defined. The majority of the relevant meta-analyses only analyzed the diagnostic performance of PET or/and PET/CT for N staging of NSCLC [2, 61, 62]. Considering the increasing numbers of reports using DWI and the unclear diagnostic value of the method, we pooled the diagnostic performance and compared it with the diagnostic performance of ^18^F-FDG PET/CT. Our results in the present meta-analysis showed that the pooled sensitivity and specificity of DWI were 0.70 and 0.97 for node-based data, and the corresponding values of PET/CT were 0.69 and 0.93, respectively; these results indicated that both ^18^F-FDG PET/CT and DWI were beneficial in detecting mediastinal lymph nodes metastases in lung cancer without significant statistical differences in diagnostic capacity. Furthermore, the diagnostic capacity (low sensitivity and high specificity) of both modalities suggested that positive lymph nodes would be missed too often so that using individuals alone cannot make accurate evaluation of nodal status to make decisions about treatment plan, especially for those patients with potentially resectable NSCLC. Instead both modalities can help guide the next step: either mediastinoscopy with minimally invasive sampling or directly surgery.

The SROC curve and its AUC presented the relationship between the sensitivity and specificity across studies and the overall estimation of test performance. The AUC for DWI (0.93, 95% CI: 0.91–0.95) was slightly higher than the AUC for ^18^F-FDG PET/CT (0.89, 95% CI: 0.86–0.91), indicating that DWI might be more accurate in N staging in patients with NSCLC. By combining the sensitivity and specificity into a single number, the DOR can be regarded as a single measurement of diagnostic accuracy, and higher values indicate better discriminatory test performance [63]. The DOR of DWI is greater than that of ^18^F-FDG PET/CT, indicating that DWI might be more accurate in assessing mediastinal lymph nodes of NSCLC. LRs, which are more clinically meaningful estimates, are commonly used to rule in and rule out disease. A good diagnostic test might have a PLR greater than 10 and a NLR less than 0.1 [48]. In our study, the PLR of DWI was 13.15 and NLR was 0.32, meaning that DWI could be only helpful to diagnose metastatic lymph nodes, not useful to exclude metastatic lesions. PET/CT could neither diagnose metastatic lesions nor rule out metastatic lesions with the PLR of 8.46 and NLR of 0.38.

The heterogeneity between studies was notable for both PET/CT and DWI. To investigate the sources of heterogeneity, diagnostic threshold analyses and subgroup analyses were performed. The spearman correlation coefficient (0.439, *P* = 0.011) suggests the existence of the threshold effect for PET/CT in our meta-analysis; one possible explanation is that different diagnostic methods and thresholds were used in the individual studies. The PET/CT images were analyzed quantitatively, qualitatively or both. Although the images were all analyzed using quantitative methods, the SUV thresholds were different. Of the included PET/CT studies using quantitative methods, only 7 studies [15, 20, 21, 33, 35, 41, 48] adopted 2.5 as the SUV cutoff value, whereas the other studies used variable values. To date, the ideal cut-off value of the SUV for diagnosing malignant MLNs has not been determined. In addition, there is no standard reference for the visual interpretation. For DWI, the results of the threshold analysis showed that no significant threshold effect existed. We also conducted subgroup analyses based on factors including study design, country, sample size, analysis method, patient enrollment, and blinding. However, these factors failed to explain the heterogeneity between PET/CT studies. For the heterogeneity in DWI studies, study design and patient enrollment were potential sources. In addition, the differences in the technique characteristics of PET/CT and DWI were potential sources of heterogeneity.

In clinical practice, DWI and ^18^F-FDG PET/CT have satisfactory specificity, and these two highly specific techniques are suitable for confirming diseases, especially some diseases with distinctive clinical manifestations or diseases that are fatal. However, with the disappointing sensitivity, a large number of patients would be misdiagnosed because of the relatively greater false negative results. DWI appears to have several advantages over FDG PET/CT, including no radiation exposure, no fasting and short examining time [9, 38]. With comparative diagnostic capacity, the cost of DWI examination is approximately one third of PET/CT examination. Although DWI shows some advantages over PET/CT, its real value for evaluating nodal status of NSCLC in clinical practice has not been determined. There is still a long way to confirm the diagnostic value of DWI, and further confirm whether it can replace PET/CT examination for N stage of NSCLC.

The current analysis has several limitations. First and foremost, the number of DWI studies included in this meta-analysis was too small. More work is needed to enrich this field. Second, a wide variation in imaging techniques likely affected the assessment of diagnostic accuracy of DWI and PET/CT and resulted in heterogeneity. Due to limited information, these factors were not analyzed. Third, although no publication bias was found by using Deek’s funnel plot, a potential publication bias could still exist, especially with the exclusion of conference abstracts and case reports during the study selection. Finally, there was no single reference standard strategy for the histopathologic analyses, and a wide variation in patient histopathologic types was found in all studies. This factor was not analyzed because it is too mixed and difficult to classify.

## Conclusion

Our meta-analysis indicated that ^18^F-FDG PET/CT and DWI had high specificity and low sensitivity for identifying metastatic mediastinal lymph nodes in NSCLC, and they are noninvasive imaging methods that might aid in confirming the diagnosis of metastases in clinical practice. However, the true value of DWI remains unknown in clinical practice, although DWI did show some advantages over PET/CT in some aspects. Therefore, large-scale, prospective studies are needed to further justify the diagnostic value of DWI in comparison with ^18^F-FDG PET/CT.

## Supporting information

S1 PRISMA Checklist (DOC)

## References

[1] Siegel RL, Miller KD, Jemal A. Cancer statistics, 2015. CA Cancer J Clin. 2015;65:5–29. 10.3322/caac.21254 25559415

[2] Pak K, Park S, Cheon GJ, Kang KW, Kim IJ, Lee DS, et al Update on nodal staging in non-small cell lung cancer with integrated positron emission tomography/computed tomography: a meta-analysis. Ann Nucl Med. 2015;29:409–419. 10.1007/s12149-015-0958-6 25655120

[3] Wu Y, Li P, Zhang H, Shi Y, Wu H, Zhang J, et al Diagnostic value of fluorine 18 fluorodeoxyglucose positron emission tomography/computed tomography for the detection of metastases in non-small-cell lung cancer patients. Int J Cancer. 2013;132:E37–47. 10.1002/ijc.27779 22890912

[4] Wu LM, Xu JR, Gu HY, Hua J, Chen J, Zhang W, et al Preoperative mediastinal and hilar nodal staging with diffusion-weighted magnetic resonance imaging and fluorodeoxyglucose positron emission tomography/computed tomography in patients with non-small-cell lung cancer: which is better? J Surg Res. 2012;178:304–314. 10.1016/j.jss.2012.03.074 22541065

[5] Usuda K, Sagawa M, Motono N, Ueno M, Tanaka M, Machida Y, et al Advantages of diffusion-weighted imaging over positron emission tomography-computed tomography in assessment of hilar and mediastinal lymph node in lung cancer. Ann Surg Oncol. 2013;20:1676–1683. 10.1245/s10434-012-2799-z 23242821

[6] Matoba M, Tonami H, Kondou T, Yokota H, Higashi K, Toga H, et al Lung carcinoma: diffusion-weighted mr imaging—preliminary evaluation with apparent diffusion coefficient. Radiology. 2007;243:570–577. 10.1148/radiol.2432060131 17400757

[7] Koh DM, Collins DJ. Diffusion-weighted MRI in the body: applications and challenges in oncology. AJR Am J Roentgenol. 2007;188:1622–1635. 10.2214/AJR.06.1403 17515386

[8] Pauls S, Schmidt SA, Juchems MS, Klass O, Luster M, Reske SN, et al Diffusion-weighted MR imaging in comparison to integrated 18F-FDG PET/CT for N-staging in patients with lung cancer. Eur J Radiol. 2012;81:178–182. 10.1016/j.ejrad.2010.09.001 20932700

[9] Usuda K, Zhao X-T, Sagawa M, Matoba M, Kuginuki Y, Taniguchi M, et al Diffusion-weighted imaging is superior to positron emission tomography in the detection and nodal assessment of lung cancers. Ann Thorac Surgery. 2011;91:1689–1695. 10.1016/j.athoracsur.2011.02.037 21619964

[10] Xu L, Tian J, Liu Y, Li C. Accuracy of diffusion-weighted (DW) MRI with background signal suppression (MR-DWIBS) in diagnosis of mediastinal lymph node metastasis of non-small-cell lung cancer (NSCLC). J Magn Reson Imaging. 2014;40:200–205. 10.1002/jmri.24343 24923480

[11] Chen W, Jian W, Li HT, Li C, Zhang YK, Xie B, et al Whole-body diffusion-weighted imaging vs. FDG-PET for the detection of non-small-cell lung cancer. How do they measure up? Magn Reson Imaging. 2010;28:613–620. 10.1016/j.mri.2010.02.009 20418042

[12] Ohno Y, Koyama H, Yoshikawa T, Nishio M, Aoyama N, Onishi Y, et al N stage disease in patients with non-small cell lung cancer: efficacy of quantitative and qualitative assessment with STIR turbo spin-echo imaging, diffusion-weighted MR imaging, and fluorodeoxyglucose PET/CT. Radiology. 2011;261:605–615. 10.1148/radiol.11110281 21926377

[13] Mori T, Nomori H, Ikeda K, Kawanaka K, Shiraishi S, Katahira K, et al Diffusion-weighted magnetic resonance imaging for diagnosing malignant pulmonary nodules/masses: comparison with positron emission tomography. J Thorac Oncol. 2008;3:358–364. 10.1097/JTO.0b013e318168d9ed 18379353

[14] Sommer G, Wiese M, Winter L, Lenz C, Klarhofer M, Forrer F, et al Preoperative staging of non-small-cell lung cancer: comparison of whole-body diffusion-weighted magnetic resonance imaging and 18F-fluorodeoxyglucose-positron emission tomography/computed tomography. Eur Radiol. 2012;22:2859–2867. 10.1007/s00330-012-2542-y 22772365

[15] Al-Sarraf N, Gately K, Lucey J, Wilson L, McGovern E, Young V. Lymph node staging by means of positron emission tomography is less accurate in non-small cell lung cancer patients with enlarged lymph nodes: Analysis of 1145 lymph nodes. Lung Cancer. 2008;60:62–68. 10.1016/j.lungcan.2007.08.036 17920724

[16] An YS, Sun JS, Park KJ, Hwang SC, Park KJ, Sheen SS, et al Diagnostic performance of (18)F-FDG PET/CT for lymph node staging in patients with operable non-small-cell lung cancer and inflammatory lung disease. Lung. 2008;186:327–336. 10.1007/s00408-008-9109-3 18670805

[17] Billé A, Pelosi E, Skanjeti A, Arena V, Errico L, Borasio P, et al Preoperative intrathoracic lymph node staging in patients with non-small-cell lung cancer: accuracy of integrated positron emission tomography and computed tomography. Eur J Cardiothoracic Surg. 2009;36:440–445. 10.1016/j.ejcts.2009.04.003 19464906

[18] Booth K, Hanna GG, McGonigle N, McManus KG, McGuigan J, O'Sullivan J, et al The mediastinal staging accuracy of 18F-Fluorodeoxyglycose positron emission tomography/computed tomography in non-small cell lung cancer with variable time intervals to surgery. Ulster Med J. 2013;82:75–81. 24082283 PMC3756862

[19] Bryant AS, Cerfolio RJ, Klemm KM, Ojha B. Maximum standard uptake value of mediastinal lymph nodes on integrated FDG-PET-CT predicts pathology in patients with non-small cell lung cancer. Ann Thorac Surg. 2006;82:417–423. 10.1016/j.athoracsur.2005.12.047 16863739

[20] Hellwig D, Graeter TP, Ukena D, Groeschel A, Sybrecht GW, Schaefers HJ, et al 18F-FDG PET for mediastinal staging of lung cancer: which SUV threshold makes sense? J Nucl Med. 2007;48:1761–1766. 10.2967/jnumed.107.044362 17942814

[21] Hu M, Yu J, Liu N, Liu L, Guo H, Yang G, et al Significance of dual-time-point 18F-FDG PET imaging in evaluation of hilar and mediastinal lymph node metastasis in non-small-cell lung cancer. Chin J Oncol. 2008;30:306–309. 18788639

[22] Jeon TY, Lee KS, Yi CA, Chung MP, Kwon OJ, Kim B-T, et al Incremental Value of PET/CT Over CT for Mediastinal Nodal Staging of Non—Small Cell Lung Cancer: Comparison Between Patients With and Without Idiopathic Pulmonary Fibrosis. Am J Roentgenol. 2010;195:370–376. 20651192 10.2214/AJR.09.3772

[23] Kim BT, Lee KS, Shim SS, Choi JY, Kwon OJ, Kim H, et al Stage T1 Non—Small Cell Lung Cancer: Preoperative Mediastinal Nodal Staging with Integrated FDG PET/CT—A Prospective Study 1. Radiology. 2006;241:501–509. 10.1148/radiol.2412051173 16966480

[24] Kim DW, Kim WH, Kim CG. Dual-time-point FDG PET/CT: Is It Useful for Lymph Node Staging in Patients with Non-Small-Cell Lung Cancer? Nucl Med Mol Imaging. 2012;46:196–200. 10.1007/s13139-012-0141-0 24900060 PMC4043038

[25] Kim YK, Lee KS, Kim BT, Choi JY, Kim H, Kwon OJ, et al Mediastinal nodal staging of nonsmall cell lung cancer using integrated 18F-FDG PET/CT in a tuberculosis-endemic country. Cancer. 2007;109:1068–1077. 10.1002/cncr.22518 17311309

[26] Kim YN, Yi CA, Lee KS, Kwon OJ, Lee HY, Kim B-T, et al A proposal for combined MRI and PET/CT interpretation criteria for preoperative nodal staging in non-small-cell lung cancer. Eur Radiol. 2012;22:1537–1546. 10.1007/s00330-012-2388-3 22367469

[27] Koksal D, Demirag F, Bayiz H, Ozmen O, Tatci E, Berktas B, et al The correlation of SUVmax with pathological characteristics of primary tumor and the value of Tumor/Lymph node SUVmax ratio for predicting metastasis to lymph nodes in resected NSCLC patients. J Cardiothorac Surg. 2013;8:63 10.1186/1749-8090-8-63 23557204 PMC3622559

[28] Kuo WH, Wu YC, Wu CY, Ho KC, Chiu PH, Wang CW, et al Node/aorta and node/liver SUV ratios from 18F-FDG PET/CT may improve the detection of occult mediastinal lymph node metastases in patients with non-small cell lung carcinoma. Acad Radiol. 2012;19:685–692. 10.1016/j.acra.2012.02.013 22459646

[29] Lee AY, Choi SJ, Jung KP, Park JS, Lee SM, Bae SK. Characteristics of Metastatic Mediastinal Lymph Nodes of Non-Small Cell Lung Cancer on Preoperative F-18 FDG PET/CT. Nucl Med Mol Imaging. 2014;48:41–46. 10.1007/s13139-013-0244-2 24900137 PMC4035153

[30] Lee JW, Kim BS, Lee DS, Chung JK, Lee MC, Kim S, et al 18F-FDG PET/CT in mediastinal lymph node staging of non-small-cell lung cancer in a tuberculosis-endemic country: consideration of lymph node calcification and distribution pattern to improve specificity. Eur J Nucl Med Mol imaging. 2009;36:1794–1802. 10.1007/s00259-009-1155-4 19430783

[31] Lee SM, Park CM, Paeng JC, Im HJ, Goo JM, Lee HJ, et al Accuracy and predictive features of FDG-PET/CT and CT for diagnosis of lymph node metastasis of T1 non-small-cell lung cancer manifesting as a subsolid nodule. Eur Radiol. 2012;22:1556–1563. 10.1007/s00330-012-2395-4 22358427

[32] Li M, Wu N, Liu Y, Zheng R, Liang Y, Zhang W, et al Regional nodal staging with 18 F-FDG PET—CT in non-small cell lung cancer: Additional diagnostic value of CT attenuation and dual-time-point imaging. Eur J Radiol. 2012;81:1886–1890. 10.1016/j.ejrad.2011.03.074 21511421

[33] Li X, Zhang H, Xing L, Ma H, Xie P, Zhang L, et al Mediastinal lymph nodes staging by 18 F-FDG PET/CT for early stage non-small cell lung cancer: a multicenter study. Radiother Oncol. 2012;102:246–250. 10.1016/j.radonc.2011.10.016 22100657

[34] Lin WY, Hsu WH, Lin KH, Wang SJ. Role of preoperative PET-CT in assessing mediastinal and hilar lymph node status in early stage lung cancer. J Chin Med Assoc. 2012;75:203–208. 10.1016/j.jcma.2012.04.004 22632985

[35] Liu BJ, Dong JC, Xu CQ, Zuo CT, Le JJ, Guan YH, et al Accuracy of 18F-FDG PET/CT for lymph node staging in non-small-cell lung cancers. Chin Med J. 2009;122:1749 19781319

[36] Morikawa M, Demura Y, Ishizaki T, Ameshima S, Miyamori I, Sasaki M, et al The effectiveness of 18F-FDG PET/CT combined with STIR MRI for diagnosing nodal involvement in the thorax. J Nucl Med. 2009;50:81–87. 10.2967/jnumed.108.056408 19091887

[37] Nakayama J, Miyasaka K, Omatsu T, Onodera Y, Terae S, Matsuno Y, et al Metastases in mediastinal and hilar lymph nodes in patients with non-small cell lung cancer: quantitative assessment with diffusion-weighted magnetic resonance imaging and apparent diffusion coefficient. J Comput Assist Tomogr. 2010;34:1–8. 10.1097/RCT.0b013e3181a9cc07 20118713

[38] Nomori H, Mori T, Ikeda K, Kawanaka K, Shiraishi S, Katahira K, et al Diffusion-weighted magnetic resonance imaging can be used in place of positron emission tomography for N staging of non-small cell lung cancer with fewer false-positive results. J Thorac Cardiovasc Surg. 2008;135:816–822. 10.1016/j.jtcvs.2007.10.035 18374761

[39] Ohno Y, Koyama H, Nogami M, Takenaka D, Yoshikawa T, Yoshimura M, et al STIR turbo SE MR imaging vs. coregistered FDG-PET/CT: quantitative and qualitative assessment of N-stage in non-small-cell lung cancer patients. J Magn Reson Imaging. 2007;26:1071–1080. 10.1002/jmri.21106 17896365

[40] Shim SS, Lee KS, Kim B-T, Chung MJ, Lee EJ, Han J, et al Non—Small Cell Lung Cancer: Prospective Comparison of Integrated FDG PET/CT and CT Alone for Preoperative Staging 1. Radiology. 2005;236:1011–1019. 10.1148/radiol.2363041310 16014441

[41] Sit AK, Sihoe AD, Suen WS, Cheng LC. Positron-emission tomography for lung cancer in a tuberculosis-endemic region. Asian Cardiovascular and Thoracic Annals. 2010;18:33–38. 10.1177/0218492309352119 20124294

[42] Tascı E, Tezel C, Orki A, Akın O, Falay O, Kutlu CA. The role of integrated positron emission tomography and computed tomography in the assessment of nodal spread in cases with non-small cell lung cancer. Interact Cardiovasc Thorac Surg. 2010;10:200–203. 10.1510/icvts.2009.220392 19933240

[43] Toba H, Kondo K, Otsuka H, Takizawa H, Kenzaki K, Sakiyama S, et al Diagnosis of the presence of lymph node metastasis and decision of operative indication using fluorodeoxyglucose-positron emission tomography and computed tomography in patients with primary lung cancer. J Med Invest. 2010;57:305–313. 20847531 10.2152/jmi.57.305

[44] Tournoy K, Maddens S, Gosselin R, Van Maele G, Van Meerbeeck J, Kelles A. Integrated FDG-PET/CT does not make invasive staging of the intrathoracic lymph nodes in non-small cell lung cancer redundant: a prospective study. Thorax. 2007;62:696–701. 10.1136/thx.2006.072959 17687098 PMC2117288

[45] Vansteenkiste JF, Stroobants SG, Dupont PJ, De Leyn PR, De Wever WF, Verbeken EK, et al FDG-PET scan in potentially operable non-small cell lung cancer: do anatometabolic PET-CT fusion images improve the localisation of regional lymph node metastases? Eur J Nucl Med. 1998;25:1495–1501. 9799345 10.1007/s002590050327

[46] Ventura E, Islam T, Gee MS, Mahmood U, Braschi M, Harisinghani MG. Detection of nodal metastatic disease in patients with non-small cell lung cancer: comparison of positron emission tomography (PET), contrast-enhanced computed tomography (CT), and combined PET-CT. Clin Imaging. 2010;34:20–28. 10.1016/j.clinimag.2009.03.012 20122515

[47] Xu N, Wang M, Zhu Z, Zhang Y, Jiao Y, Fang W. Integrated positron emission tomography and computed tomography in preoperative lymph node staging of non-small cell lung cancer. Chin Med J (Engl). 2014;127:607–613. 24534208

[48] Yang W, Fu Z, Yu J, Yuan S, Zhang B, Li D, et al Value of PET/CT versus enhanced CT for locoregional lymph nodes in non-small cell lung cancer. Lung Cancer. 2008;61:35–43. 10.1016/j.lungcan.2007.11.007 18177978

[49] Yi CA, Lee KS, Kim B-T, Shim SS, Chung MJ, Sung YM, et al Efficacy of helical dynamic CT versus integrated PET/CT for detection of mediastinal nodal metastasis in non-small cell lung cancer. Am J Roentgenol. 2007;188:318–325. 17242237 10.2214/AJR.05.2081

[50] Zeng Z, Liao Q, Cai J, Liu A. Diffusion-weighted imaging and apparent diffusion coefficient values in the differential diagnosis of hilar and mediastinal lymph nodes of non-small cell lung cancer. Chin J Clin Oncol. 2012;39:706–710.

[51] He W, Zhou X, He W, Xu J, Guo L. Value of diffusion weighted imaging in diagnosis of metastatic lymph nodes in lung cancer. Chin J Med Imaging Technol. 2011;27:2013–2016.

[52] Zhang X, Xing W, Chen J, Ding J, Gao X, Shen N. Application of DWI in differentail dignosis of lymph nodes in patietns with lung cancer. Chin Compu Med Imaging. 2013;19:213–216.

[53] Zhou Y, Xia J. Clinical value of 18F-FDG PET-CT imaging in the preoperative diagnosis and staging of regional lymph nodes in non-small cell lung cancer. Chin J CT& MRI. 2014;12:70–74.

[54] Whiting PF, Rutjes AW, Westwood ME, Mallett S, Deeks JJ, Reitsma JB, et al QUADAS-2: a revised tool for the quality assessment of diagnostic accuracy studies. Ann Intern Med. 2011;155:529–536. 10.7326/0003-4819-155-8-201110180-00009 22007046

[55] Lv YL, Yuan DM, Wang K, Miao XH, Qian Q, Wei SZ, et al Diagnostic performance of integrated positron emission tomography/computed tomography for mediastinal lymph node staging in non-small cell lung cancer: a bivariate systematic review and meta-analysis. J Thorac Oncol. 2011;6:1350–1358. 10.1097/JTO.0b013e31821d4384 21642874

[56] Wu LM, Hu JN, Hua J, Liu MJ, Chen J, Xu JR. Diagnostic value of diffusion-weighted magnetic resonance imaging compared with fluorodeoxyglucose positron emission tomography/computed tomography for pancreatic malignancy: a meta-analysis using a hierarchical regression model. J Gastroenterol Hepatol. 2012;27:1027–1035. 10.1111/j.1440-1746.2012.07112.x 22414092

[57] Yamamura J, Salomon G, Buchert R, Hohenstein A, Graessner J, Huland H, et al Magnetic resonance imaging of prostate cancer: diffusion-weighted imaging in comparison with sextant biopsy. J Comput Assist Tomogr. 2011;35:223–228. 10.1097/RCT.0b013e3181fc5409 21412094

[58] Matsuki M, Inada Y, Tatsugami F, Tanikake M, Narabayashi I, Katsuoka Y. Diffusion-weighted MR imaging for urinary bladder carcinoma: initial results. Eur Radiol. 2007;17:201–204. 10.1007/s00330-006-0281-7 16865369

[59] Busard MP, Mijatovic V, van Kuijk C, Pieters-van den Bos IC, Hompes PG, van Waesberghe JH. Magnetic resonance imaging in the evaluation of (deep infiltrating) endometriosis: the value of diffusion-weighted imaging. J Magn Reson Imaging. 2010;31:1117–1123. 10.1002/jmri.22139 20432346

[60] Ichikawa T, Erturk SM, Motosugi U, Sou H, Iino H, Araki T, et al High-B-value diffusion-weighted MRI in colorectal cancer. AJR Am J Roentgenol. 2006;187:181–184. 10.2214/AJR.05.1005 16794174

[61] Schmidt-Hansen M, Baldwin DR, Hasler E, Zamora J, Abraira V, Roque IFM. PET-CT for assessing mediastinal lymph node involvement in patients with suspected resectable non-small cell lung cancer. Cochrane Database Syst Rev. 2014;11:CD009519. 10.1002/14651858.CD009519.pub2 PMC6472607 25393718

[62] Wang Z, Wang Y, Sui X, Zhang W, Shi R, Zhang Y, et al Performance of FLT-PET for pulmonary lesion diagnosis compared with traditional FDG-PET: A meta-analysis. Eur J Radiol. 2015;84:1371–1377. 10.1016/j.ejrad.2015.03.025 25864441

[63] Glas AS, Lijmer JG, Prins MH, Bonsel GJ, Bossuyt PM. The diagnostic odds ratio: a single indicator of test performance. J Clin Epidemiol. 2003;56:1129–1135. 14615004 10.1016/s0895-4356(03)00177-x

