# Correction: Comparison of ^18^F-FDG PET/CT and DWI for detection of mediastinal nodal metastasis in non-small cell lung cancer: A meta-analysis

**DOI:** 10.1371/journal.pone.0176150

**Published:** 2017-04-13

**Authors:** Guohua Shen, You Lan, Kan Zhang, Pengwei Ren, Zhiyun Jia

The affiliation for the last author is incorrect. Zhiyun Jia is not affiliated with #2 but with #1 Department of Nuclear Medicine, West China Hospital, Sichuan University, Chengdu, Sichuan, People’s Republic of China.
