# Supplementary material for: Expression of Concern: Comparison of 18F-FDG PET/CT and DWI for detection of mediastinal nodal metastasis in non-small cell lung cancer: A meta-analysis
Source: PLoS One. 2024 Feb 14;19(2):e0299045. doi: 10.1371/journal.pone.0299045 (PMC10866507; doi:10.1371/journal.pone.0299045)
Supplement: S1 File — (ZIP) [file pone.0299045.s001.zip › statistical analysis/PET╩2╛▌/π╨╓╡╨o╙a.docx]

**Analysis of Diagnostic Threshold**

--------------------------------------------------------------------------------

Spearman correlation coefficient: 0.556 p-value= 0.000

(Logit(TPR) vs Logit(FPR)

--------------------------------------------------------------------------------

Moses' model (D = a + bS)

Weighted regression (Inverse Variance)

Var Coeff. Std. Error T p-value

--------------------------------------------------------------------------------

a 2.879 0.232 12.417 0.0000

b( 1) -0.180 0.088 2.041 0.0486

--------------------------------------------------------------------------------

Tau-squared estimate = 0.7725 (Convergence is achieved after 6 iterations)

Restricted Maximum Likelihood estimation (REML)

No. studies = 38

Filter OFF

Add 1/2 to all cells of the studies with zero
