# Supplementary figures and images for: Expression of Concern: Comparison of 18F-FDG PET/CT and DWI for detection of mediastinal nodal metastasis in non-small cell lung cancer: A meta-analysis (part 2 of 2)
Source: PLoS One. 2024 Feb 14;19(2):e0299045. doi: 10.1371/journal.pone.0299045 (PMC10866507; doi:10.1371/journal.pone.0299045)

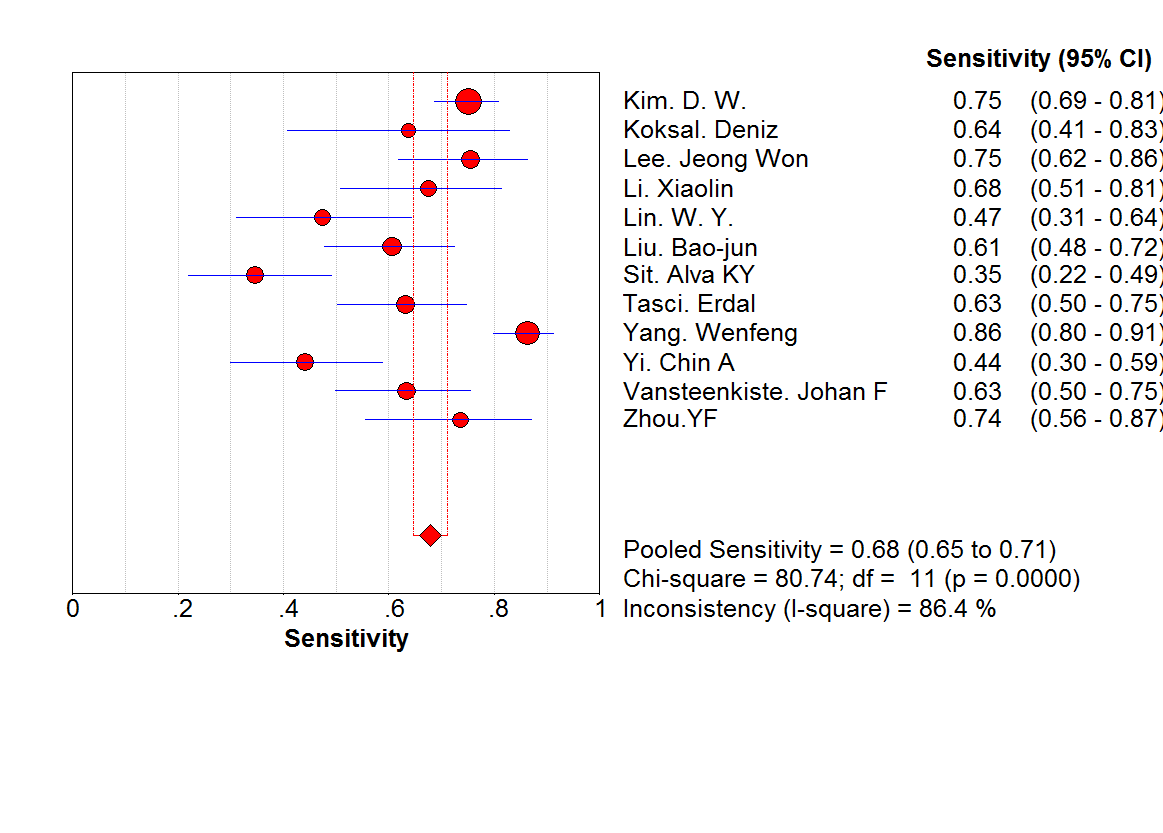

Supplement: S1 File — (ZIP) [file pone.0299045.s001.zip › statistical analysis/PET╩2╛▌/╤╟╫Θ╖╓╬÷/enrollment nonconsecutive/sen.bmp]

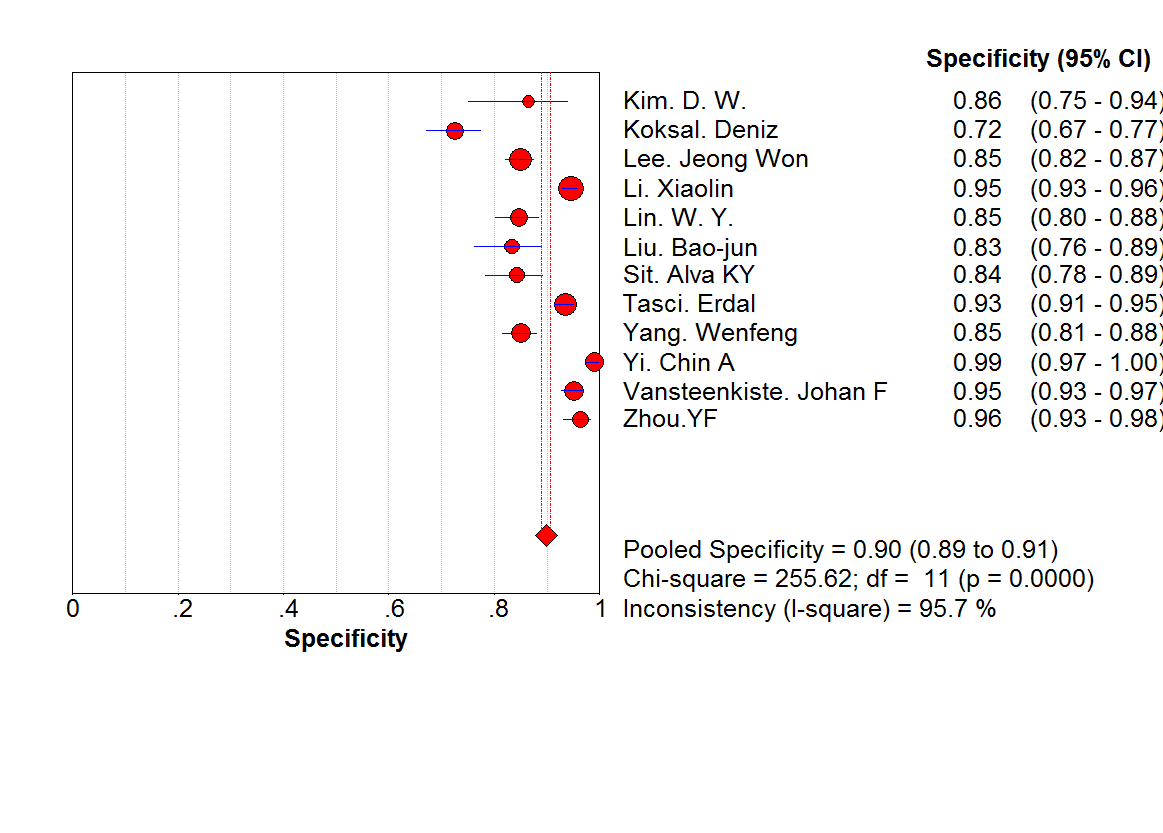

Supplement: S1 File — (ZIP) [file pone.0299045.s001.zip › statistical analysis/PET╩2╛▌/╤╟╫Θ╖╓╬÷/enrollment nonconsecutive/spe.bmp]

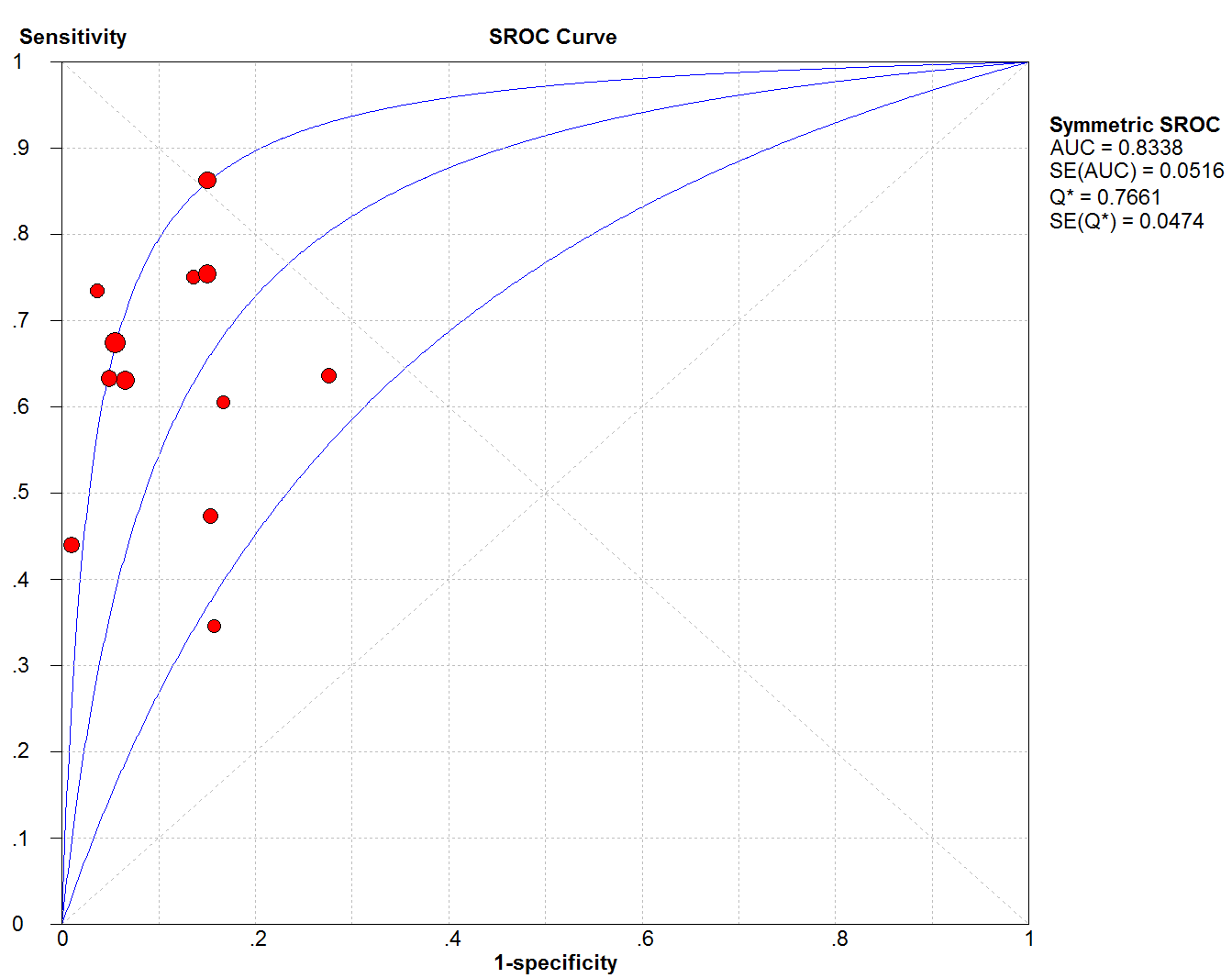

Supplement: S1 File — (ZIP) [file pone.0299045.s001.zip › statistical analysis/PET╩2╛▌/╤╟╫Θ╖╓╬÷/enrollment nonconsecutive/sroc.bmp]

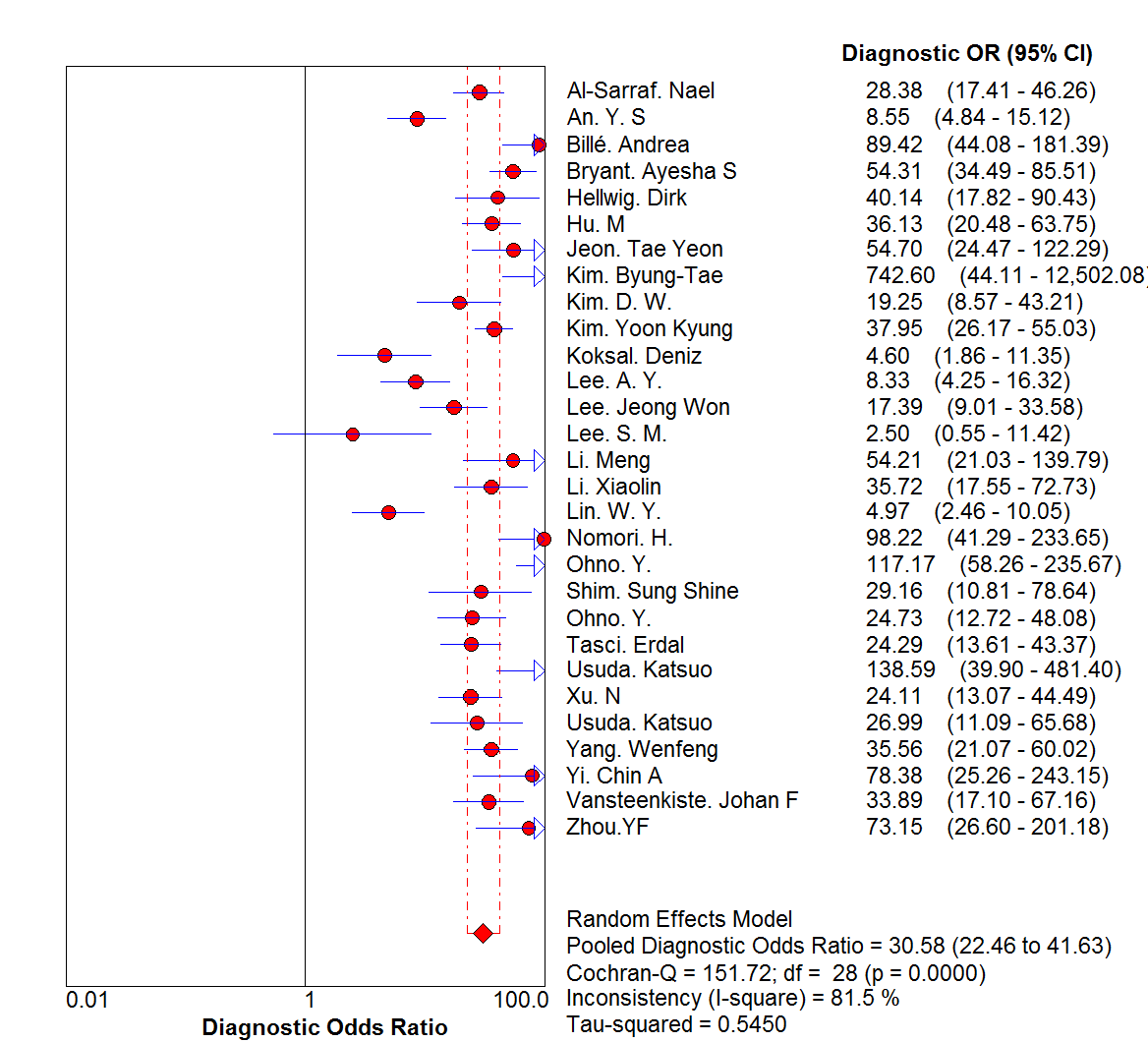

Supplement: S1 File — (ZIP) [file pone.0299045.s001.zip › statistical analysis/PET╩2╛▌/╤╟╫Θ╖╓╬÷/sample ┤≤╙┌250/dor.bmp]

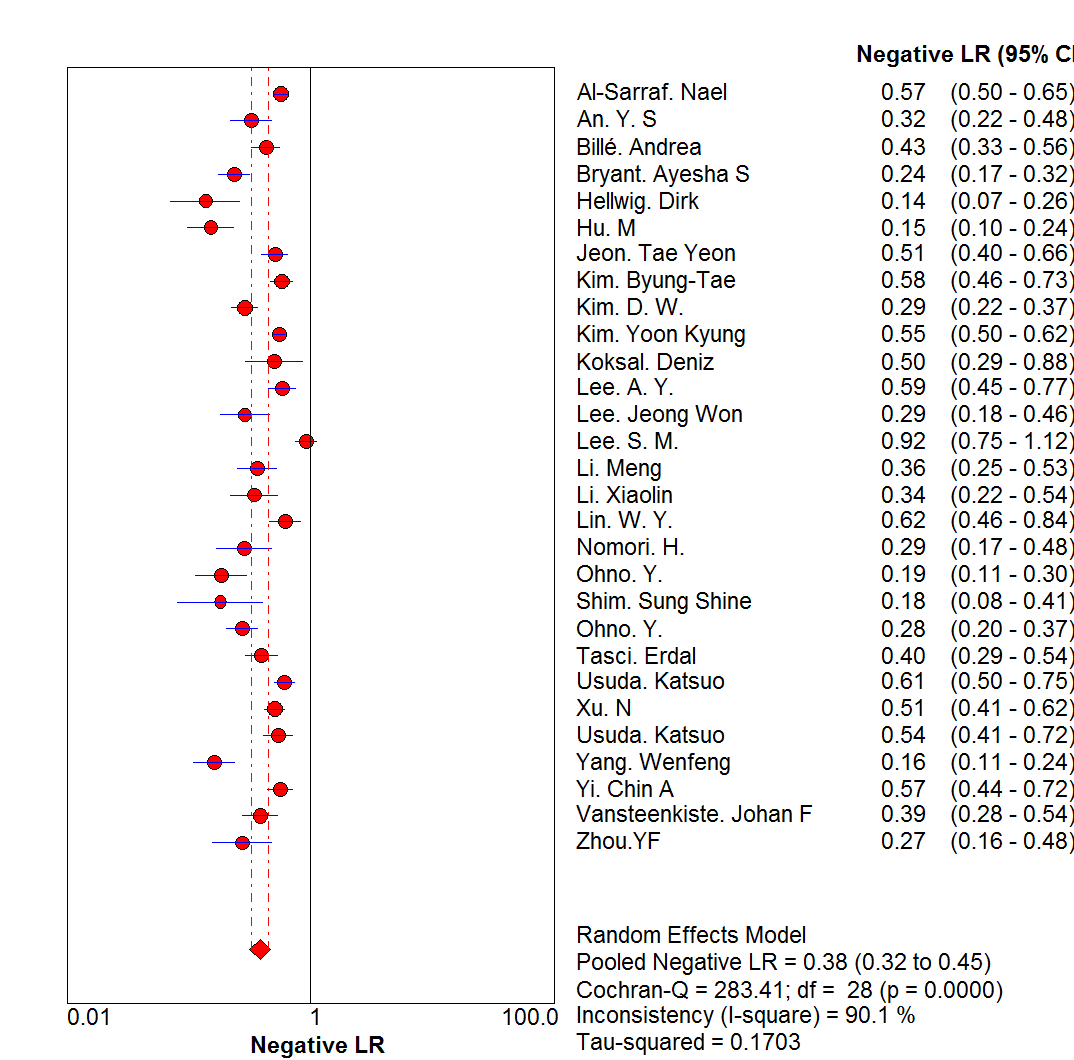

Supplement: S1 File — (ZIP) [file pone.0299045.s001.zip › statistical analysis/PET╩2╛▌/╤╟╫Θ╖╓╬÷/sample ┤≤╙┌250/nlr.bmp]

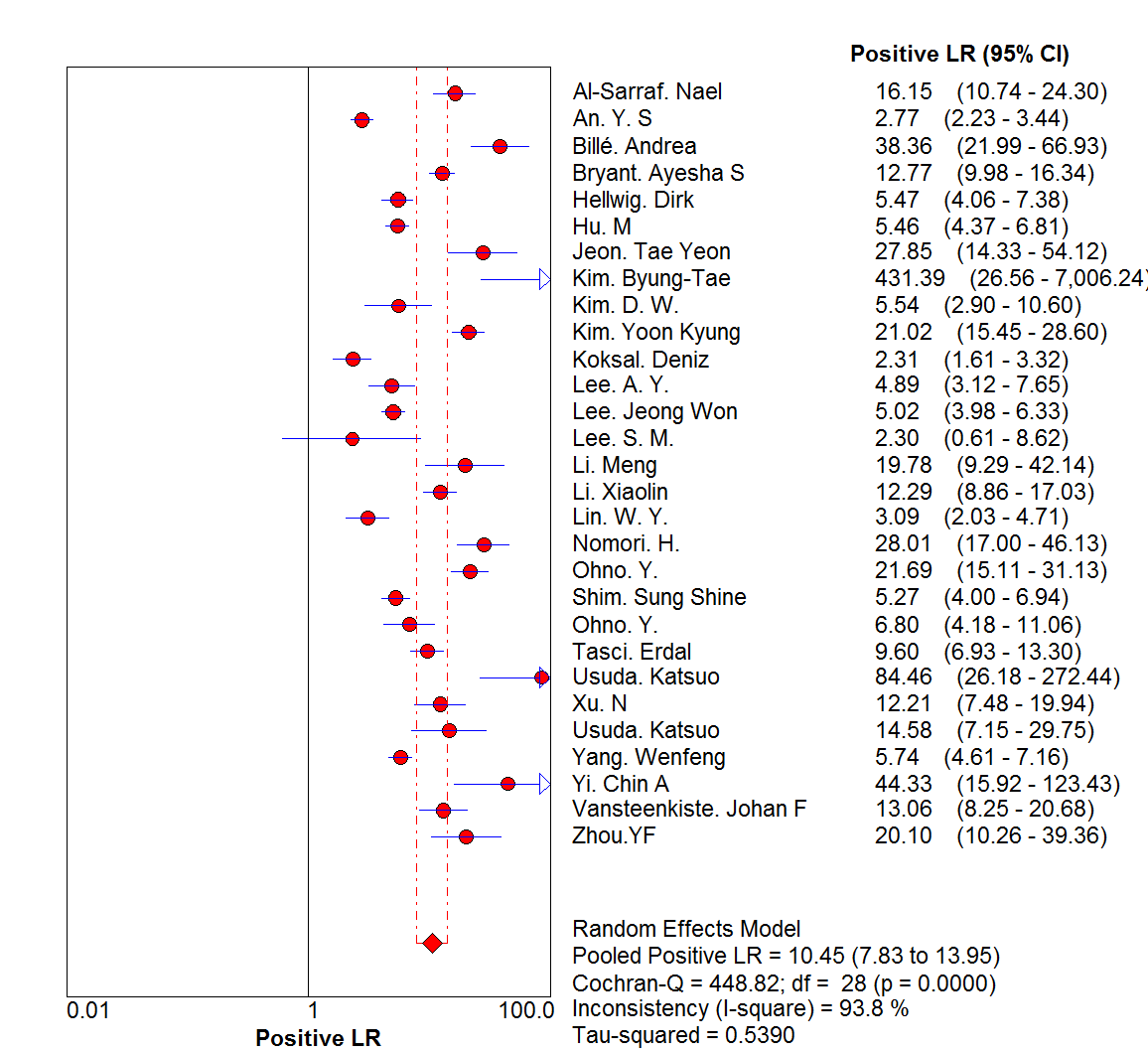

Supplement: S1 File — (ZIP) [file pone.0299045.s001.zip › statistical analysis/PET╩2╛▌/╤╟╫Θ╖╓╬÷/sample ┤≤╙┌250/plr.bmp]

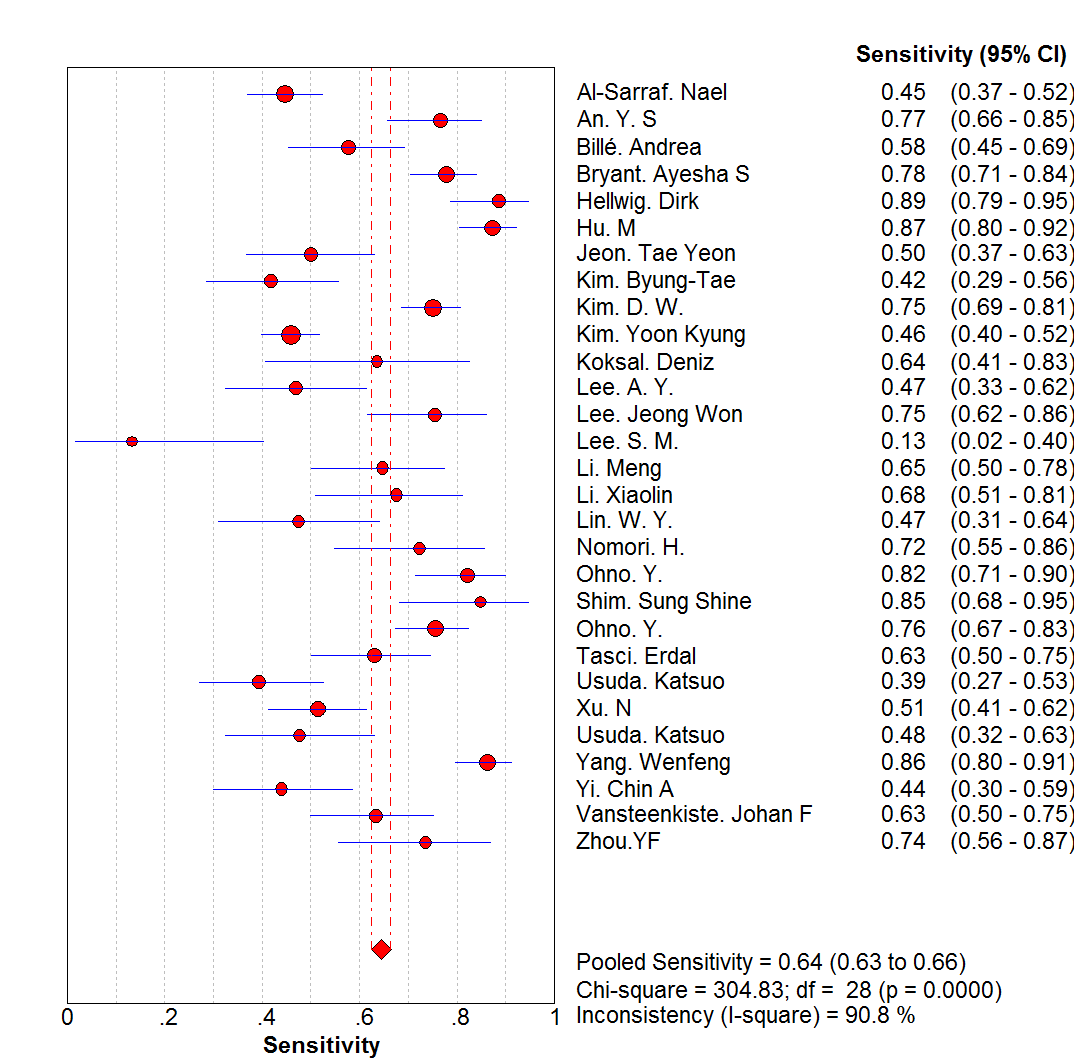

Supplement: S1 File — (ZIP) [file pone.0299045.s001.zip › statistical analysis/PET╩2╛▌/╤╟╫Θ╖╓╬÷/sample ┤≤╙┌250/sen.bmp]

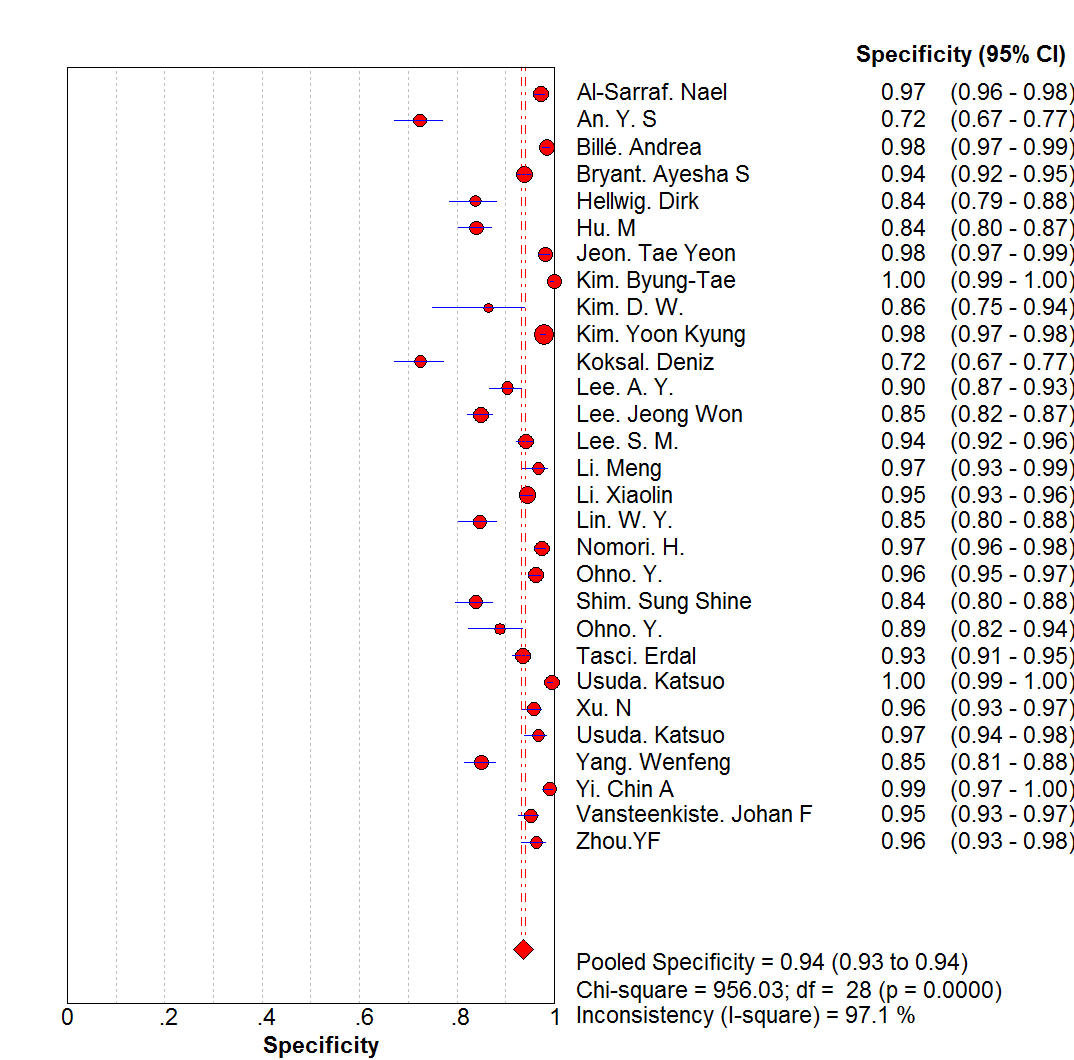

Supplement: S1 File — (ZIP) [file pone.0299045.s001.zip › statistical analysis/PET╩2╛▌/╤╟╫Θ╖╓╬÷/sample ┤≤╙┌250/spe.bmp]

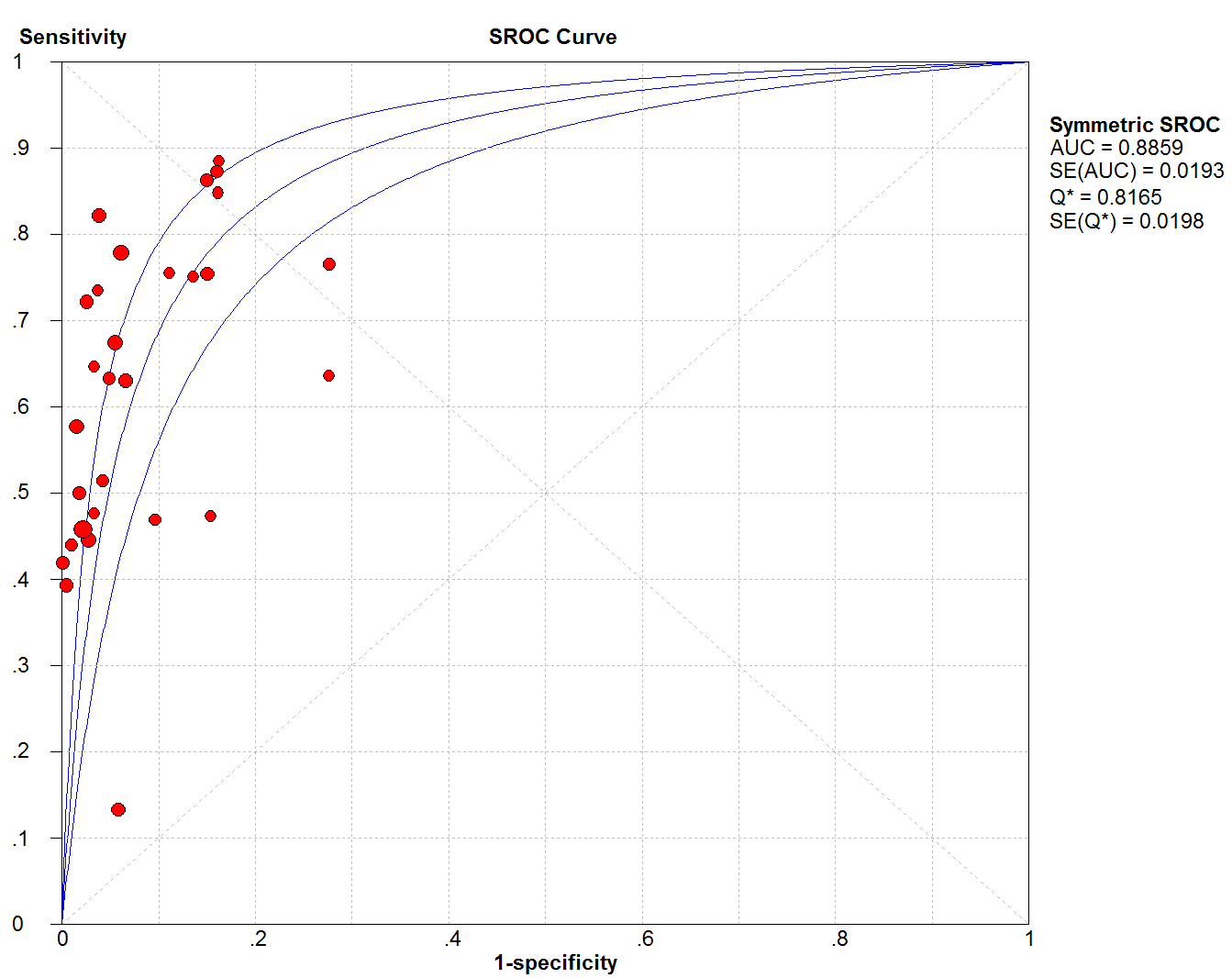

Supplement: S1 File — (ZIP) [file pone.0299045.s001.zip › statistical analysis/PET╩2╛▌/╤╟╫Θ╖╓╬÷/sample ┤≤╙┌250/sroc.bmp]

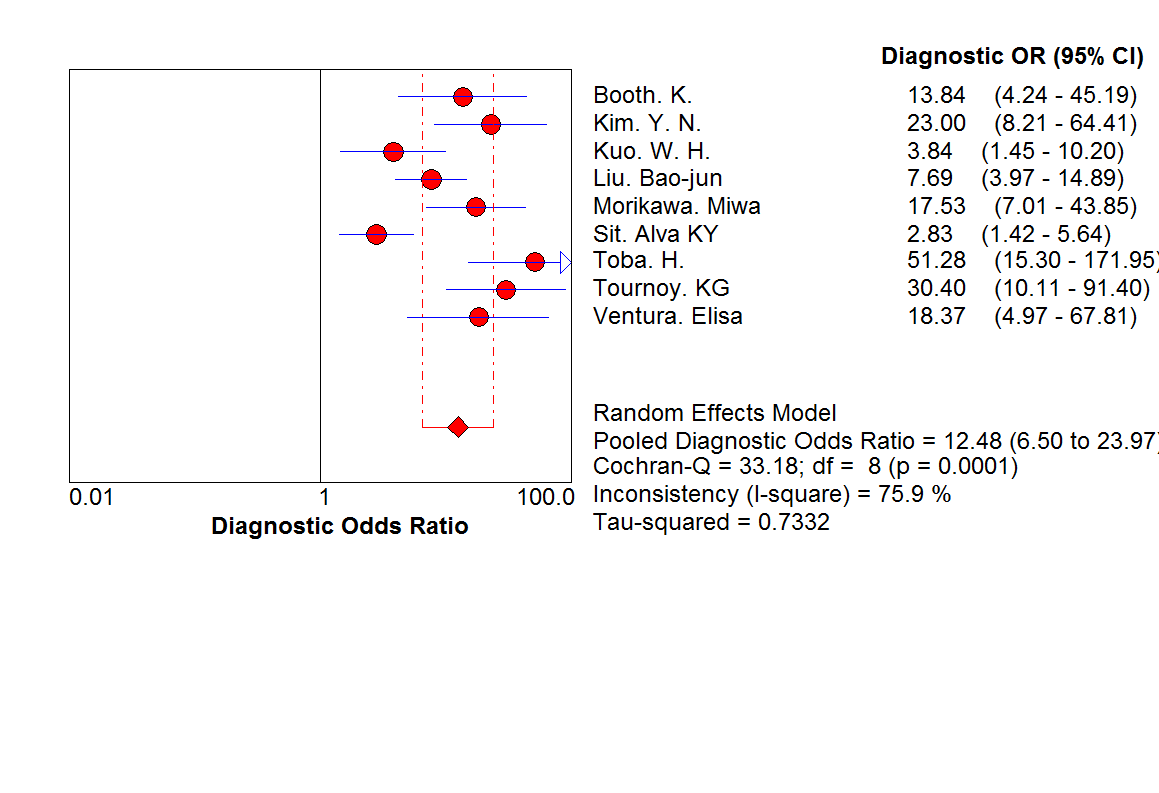

Supplement: S1 File — (ZIP) [file pone.0299045.s001.zip › statistical analysis/PET╩2╛▌/╤╟╫Θ╖╓╬÷/sample ╨í╙┌250/dor.bmp]

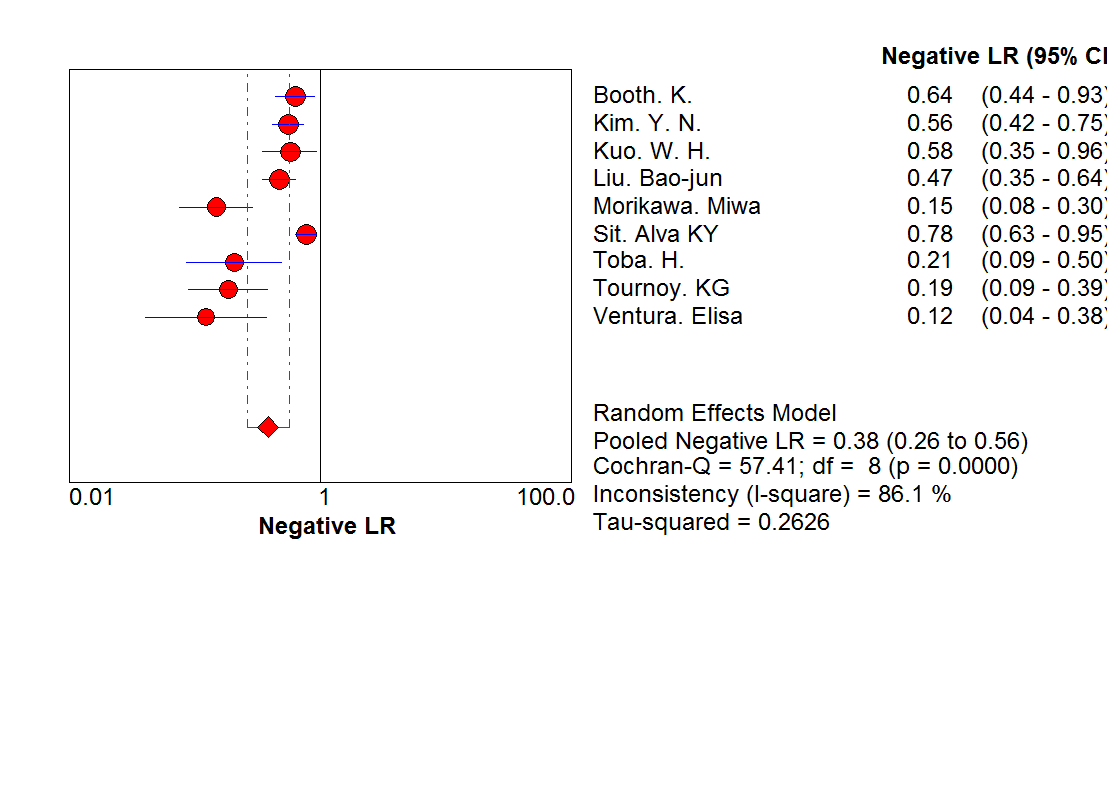

Supplement: S1 File — (ZIP) [file pone.0299045.s001.zip › statistical analysis/PET╩2╛▌/╤╟╫Θ╖╓╬÷/sample ╨í╙┌250/nlr.bmp]

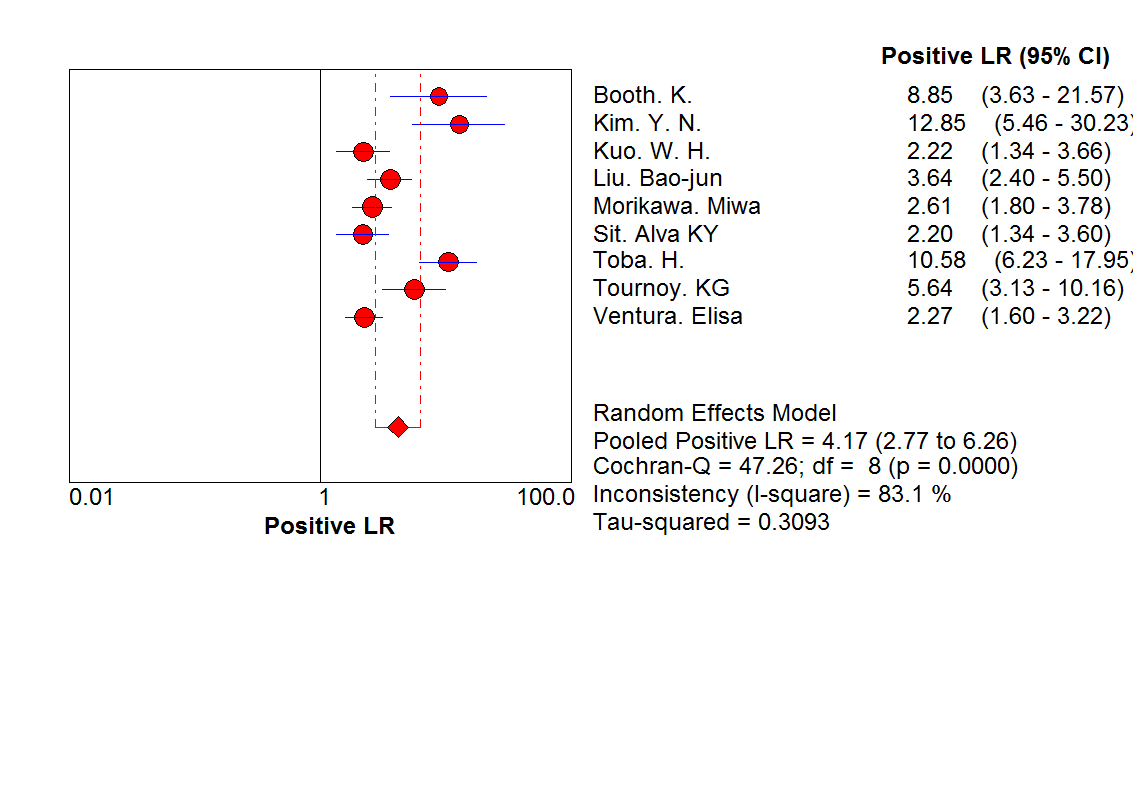

Supplement: S1 File — (ZIP) [file pone.0299045.s001.zip › statistical analysis/PET╩2╛▌/╤╟╫Θ╖╓╬÷/sample ╨í╙┌250/plr.bmp]

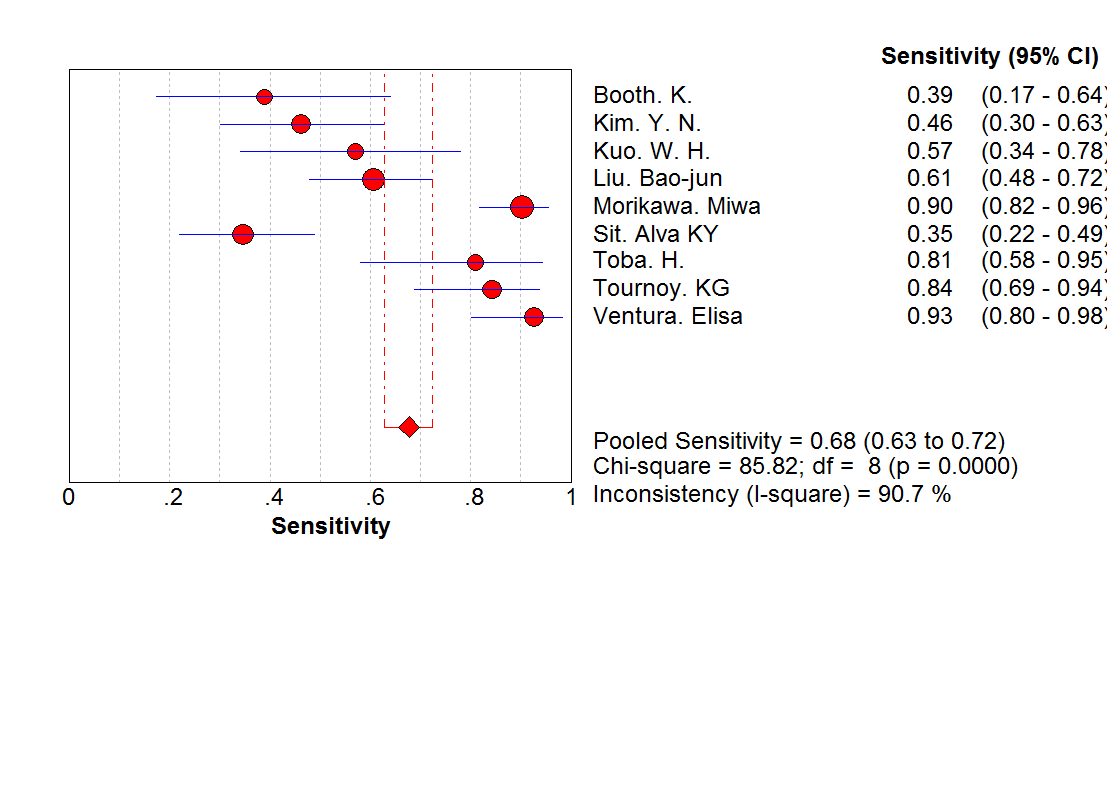

Supplement: S1 File — (ZIP) [file pone.0299045.s001.zip › statistical analysis/PET╩2╛▌/╤╟╫Θ╖╓╬÷/sample ╨í╙┌250/sen.bmp]

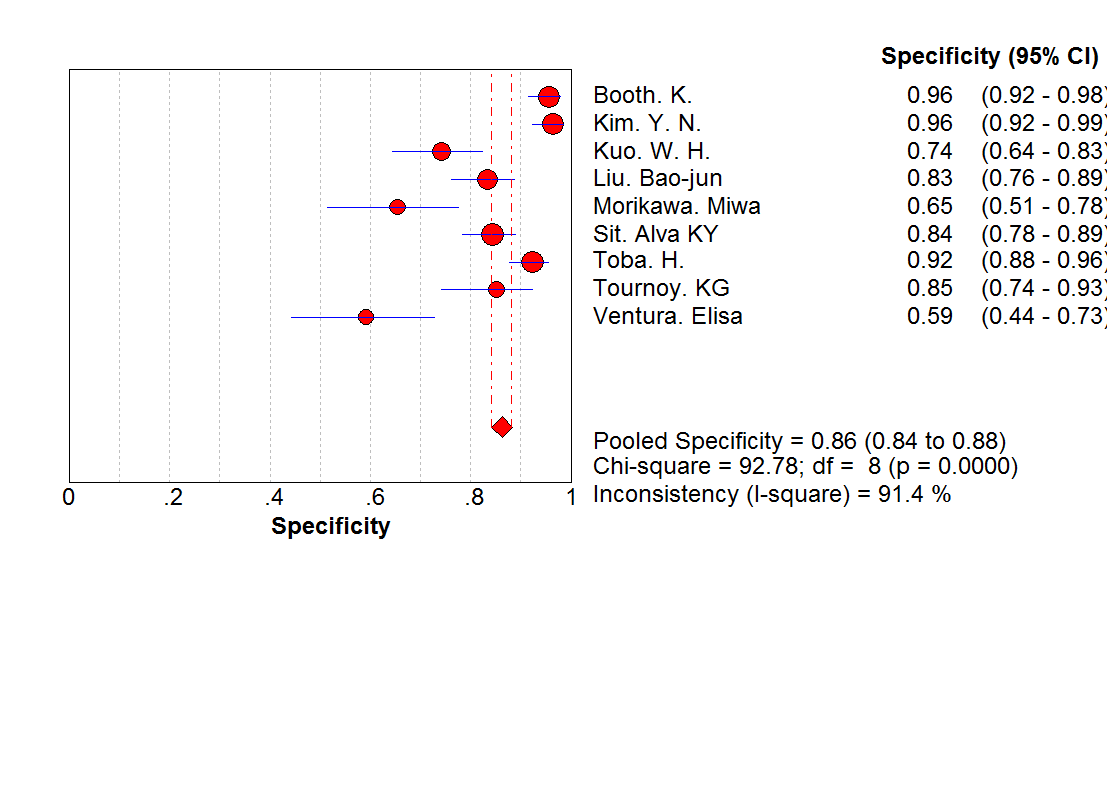

Supplement: S1 File — (ZIP) [file pone.0299045.s001.zip › statistical analysis/PET╩2╛▌/╤╟╫Θ╖╓╬÷/sample ╨í╙┌250/spe.bmp]

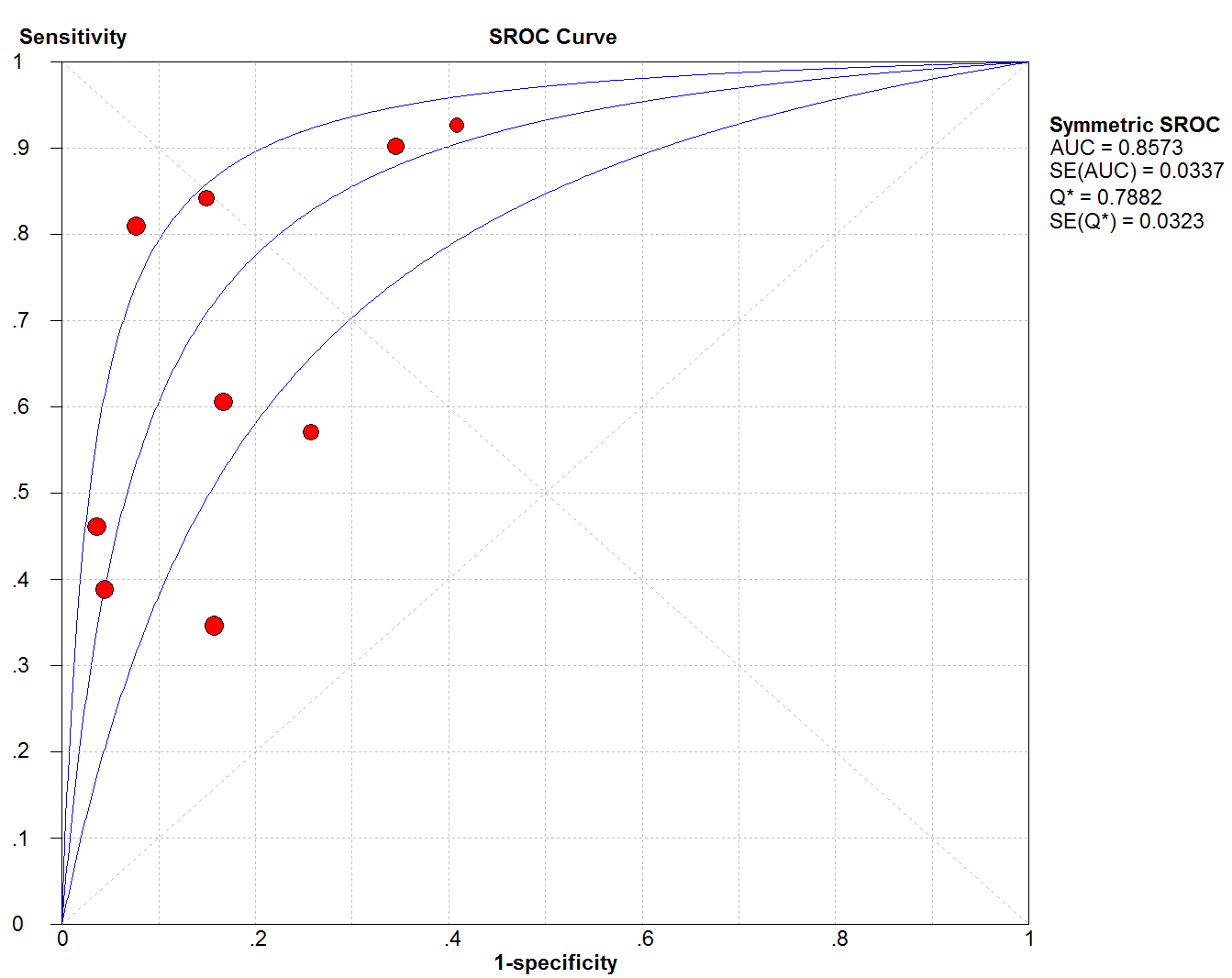

Supplement: S1 File — (ZIP) [file pone.0299045.s001.zip › statistical analysis/PET╩2╛▌/╤╟╫Θ╖╓╬÷/sample ╨í╙┌250/sroc.bmp]

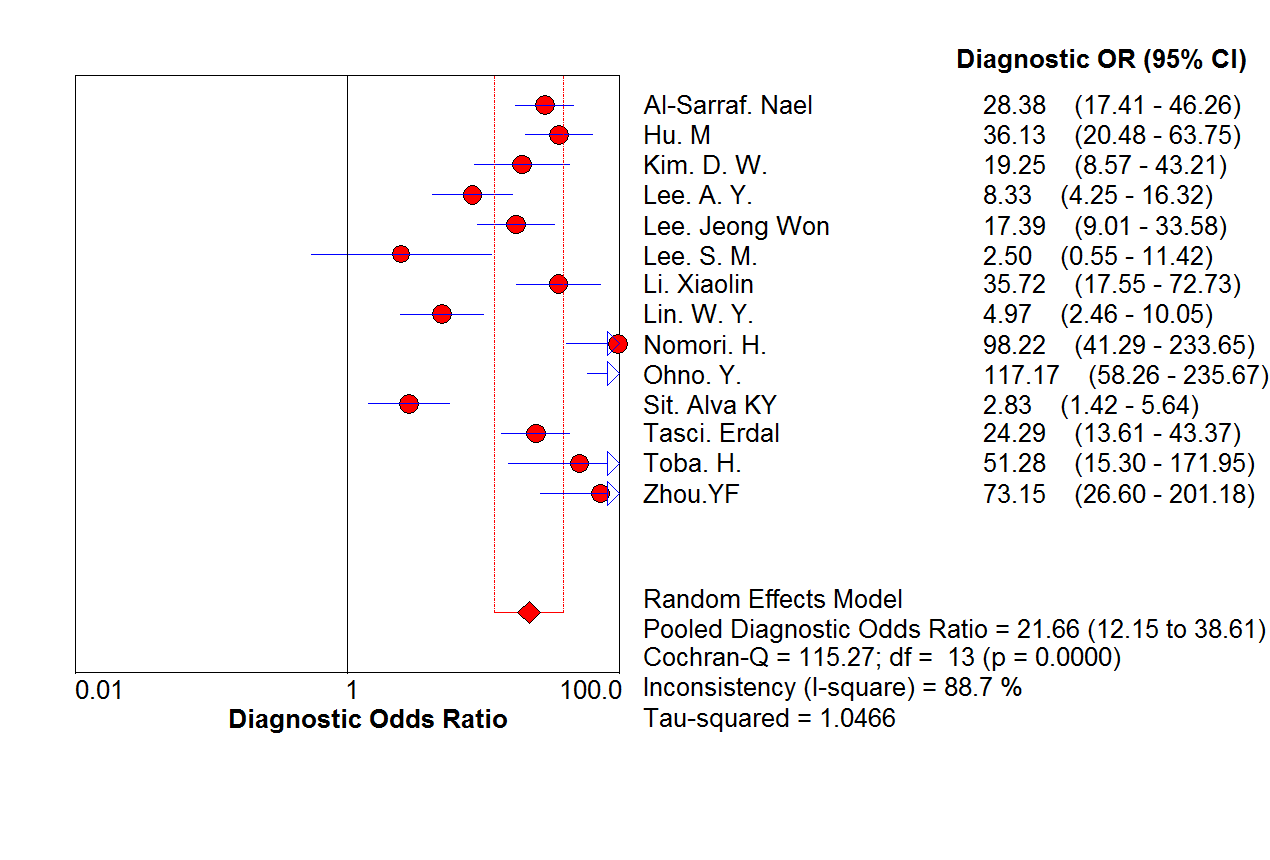

Supplement: S1 File — (ZIP) [file pone.0299045.s001.zip › statistical analysis/PET╩2╛▌/╤╟╫Θ╖╓╬÷/unblind/dor.bmp]

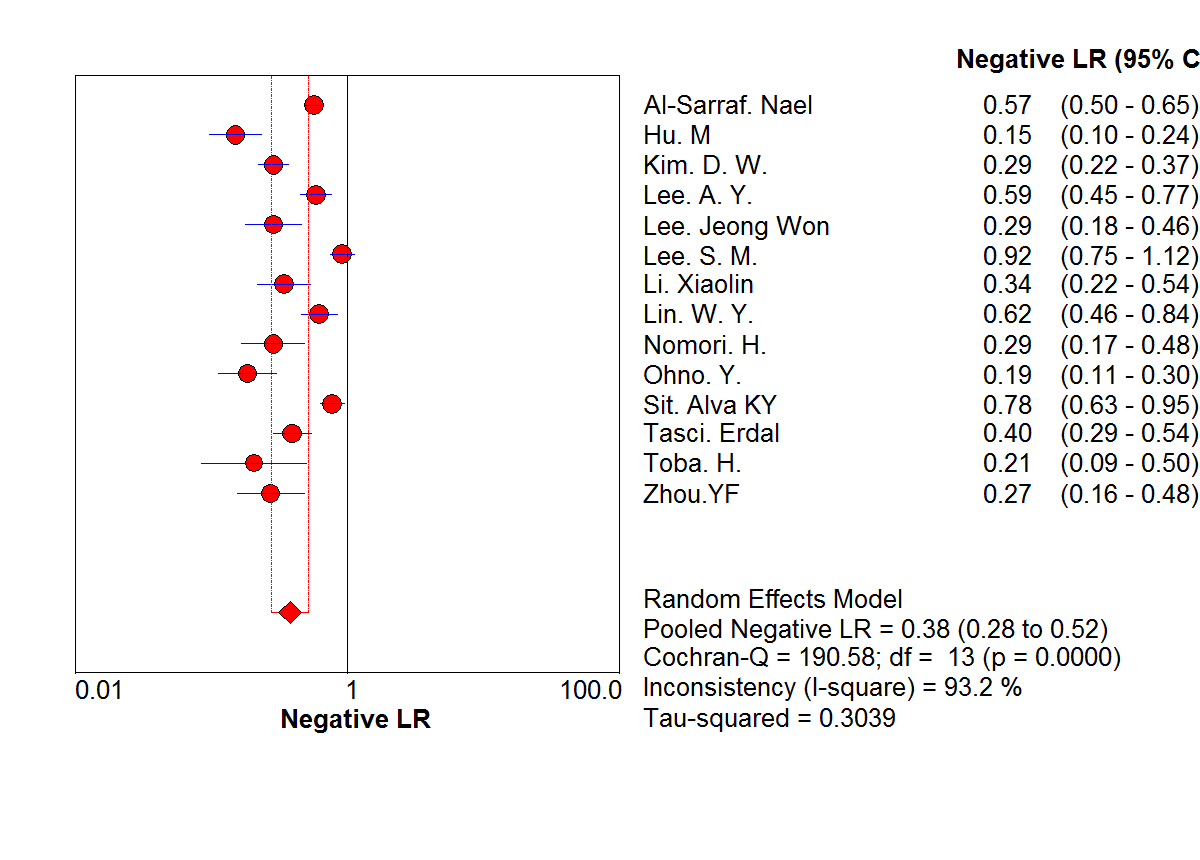

Supplement: S1 File — (ZIP) [file pone.0299045.s001.zip › statistical analysis/PET╩2╛▌/╤╟╫Θ╖╓╬÷/unblind/nlr.bmp]

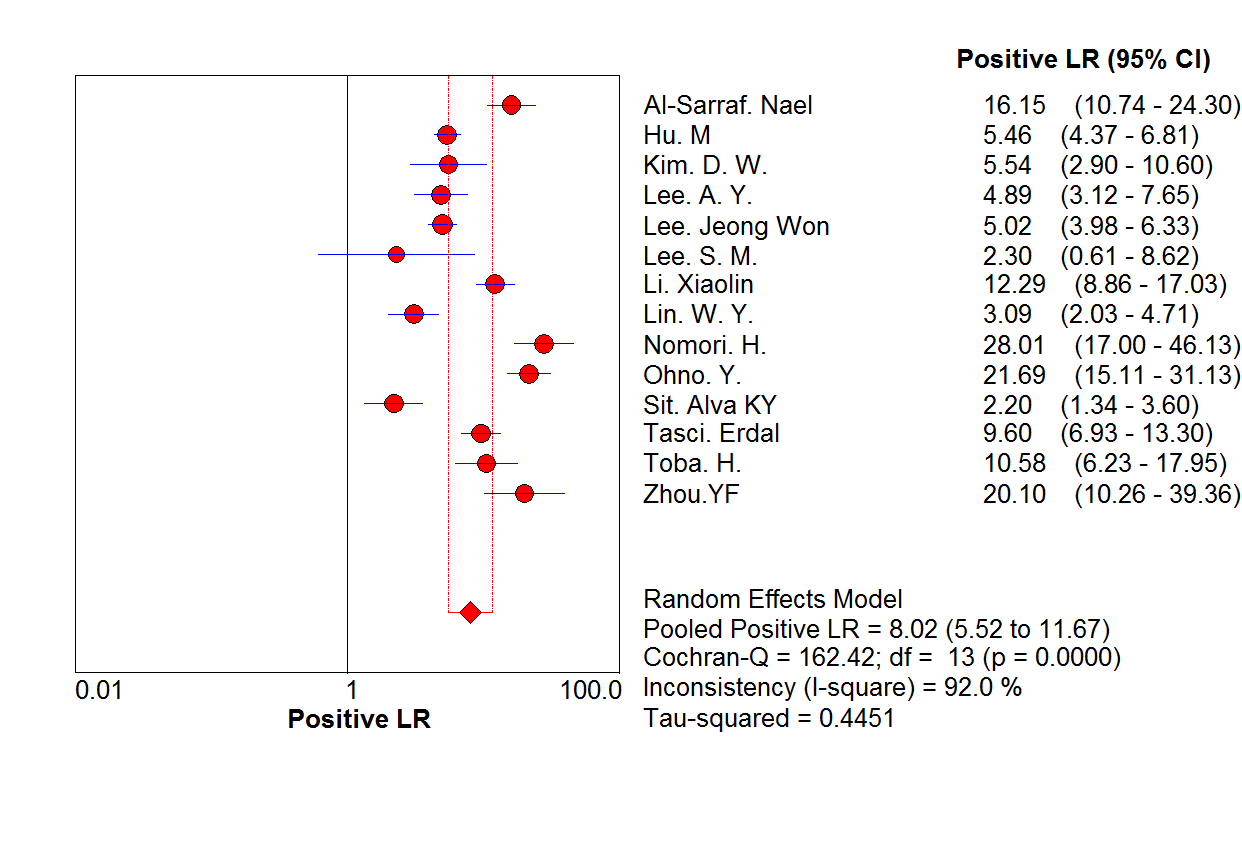

Supplement: S1 File — (ZIP) [file pone.0299045.s001.zip › statistical analysis/PET╩2╛▌/╤╟╫Θ╖╓╬÷/unblind/plr.bmp]

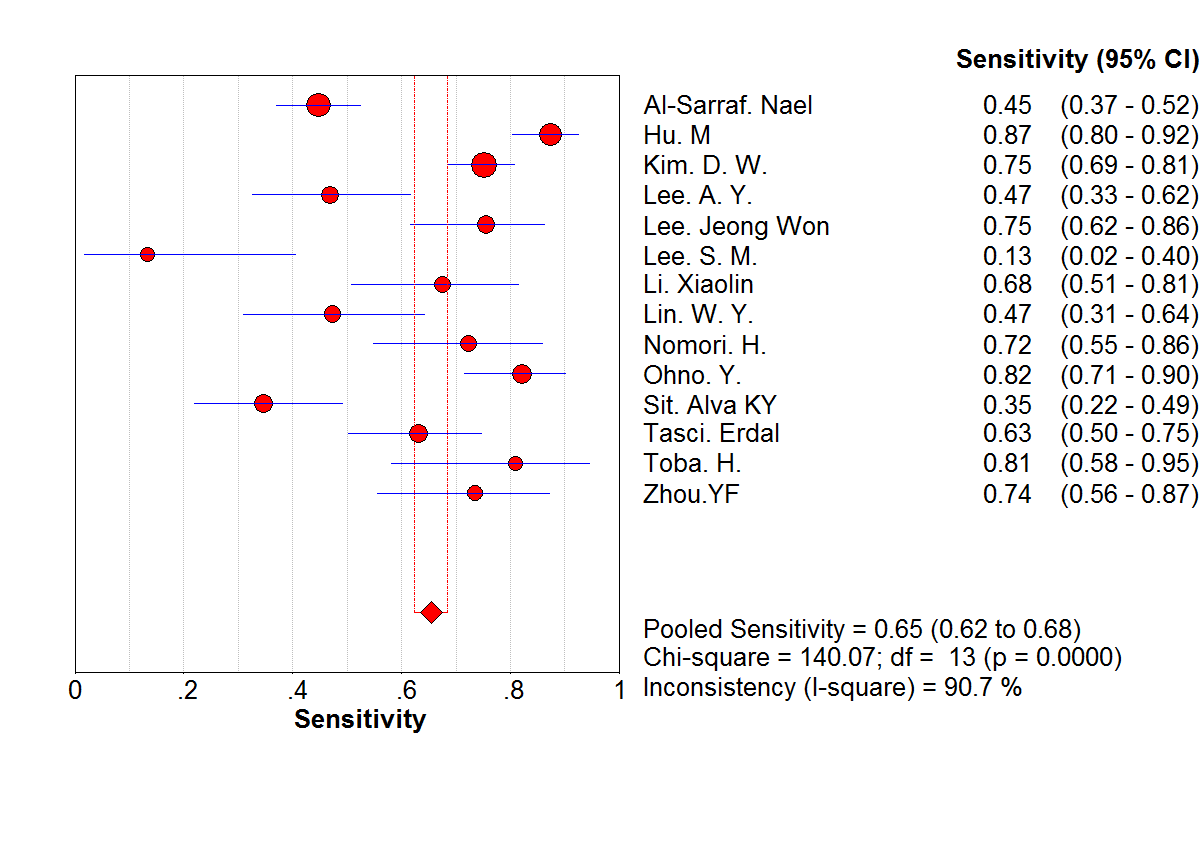

Supplement: S1 File — (ZIP) [file pone.0299045.s001.zip › statistical analysis/PET╩2╛▌/╤╟╫Θ╖╓╬÷/unblind/sen.bmp]

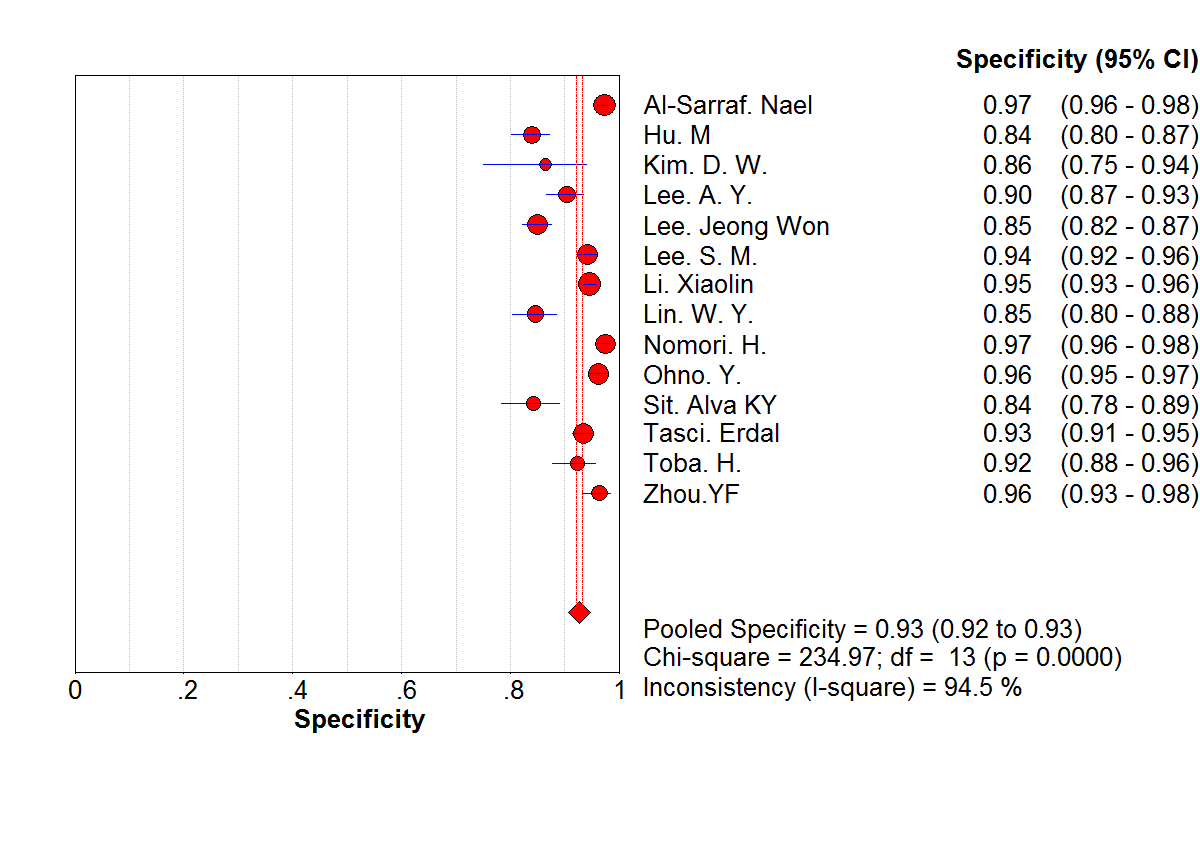

Supplement: S1 File — (ZIP) [file pone.0299045.s001.zip › statistical analysis/PET╩2╛▌/╤╟╫Θ╖╓╬÷/unblind/spe.bmp]

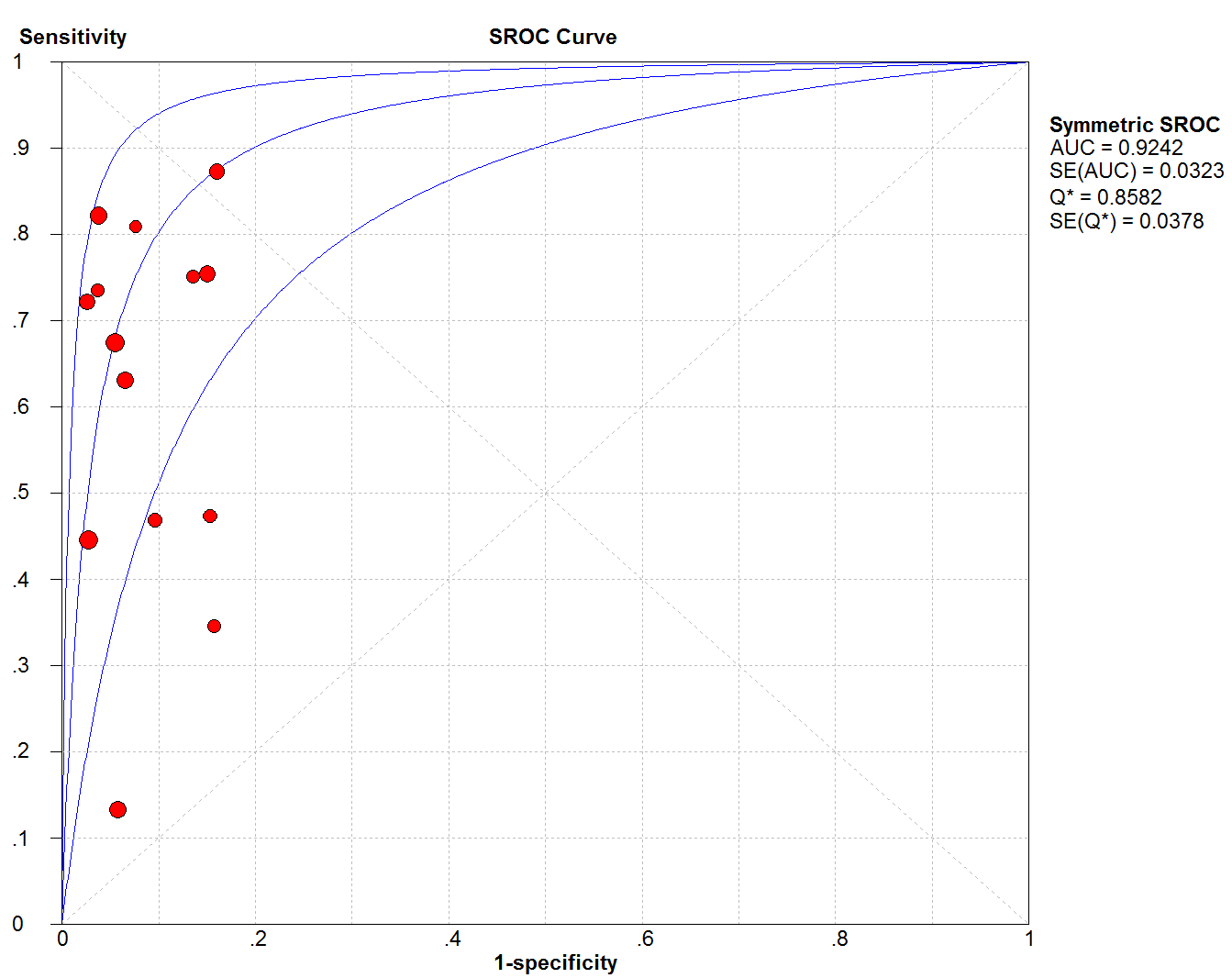

Supplement: S1 File — (ZIP) [file pone.0299045.s001.zip › statistical analysis/PET╩2╛▌/╤╟╫Θ╖╓╬÷/unblind/sroc.bmp]

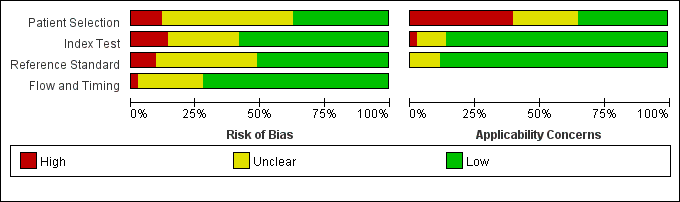

Supplement: S1 File — (ZIP) [file pone.0299045.s001.zip › statistical analysis/╓╩┴┐╞└╣└/Methodological quality graph.png]

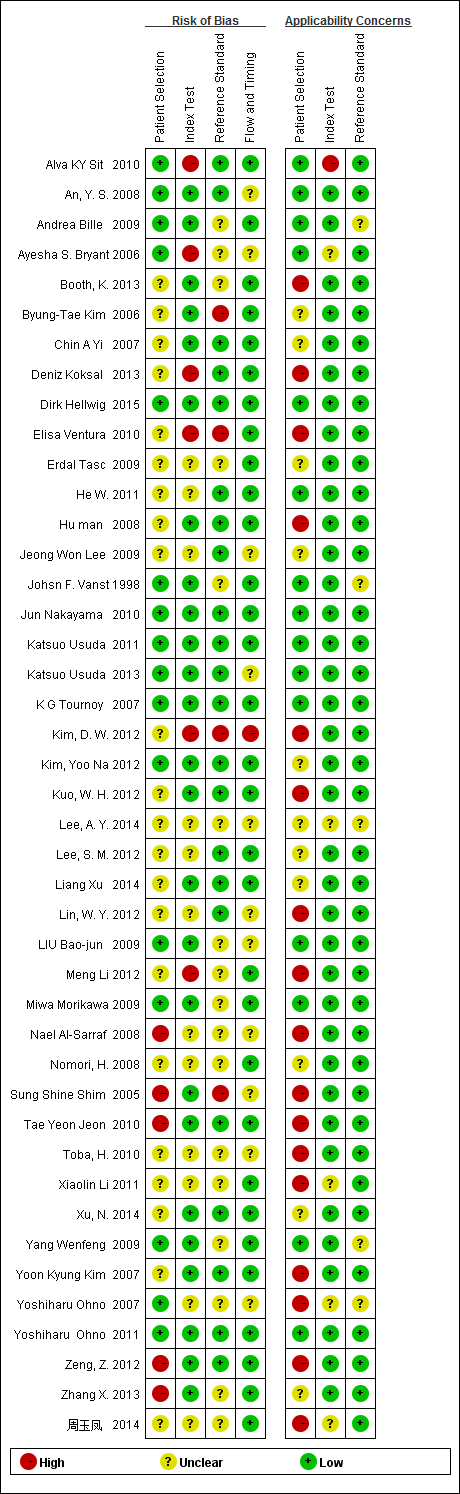

Supplement: S1 File — (ZIP) [file pone.0299045.s001.zip › statistical analysis/╓╩┴┐╞└╣└/Methodological quality summary.png]
