# Supplementary figures and images for: Expression of Concern: Comparison of 18F-FDG PET/CT and DWI for detection of mediastinal nodal metastasis in non-small cell lung cancer: A meta-analysis (part 1 of 2)
Source: PLoS One. 2024 Feb 14;19(2):e0299045. doi: 10.1371/journal.pone.0299045 (PMC10866507; doi:10.1371/journal.pone.0299045)

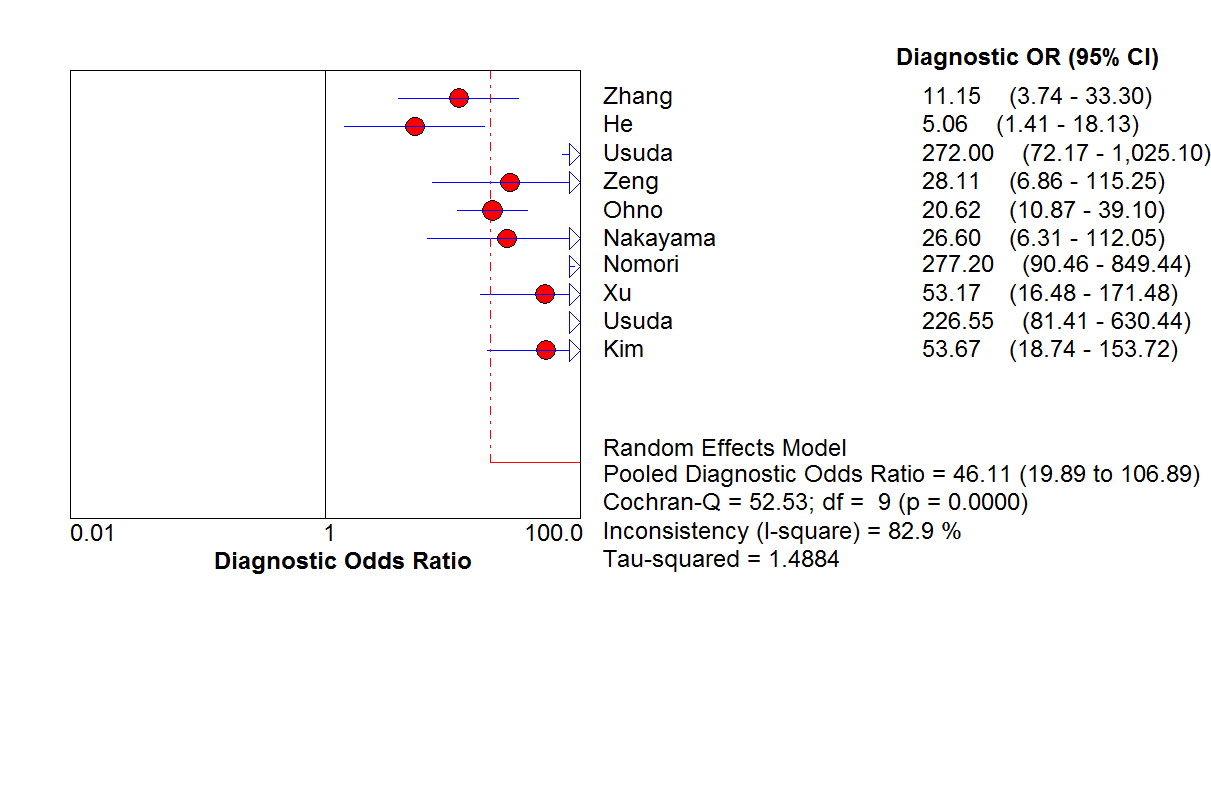

Supplement: S1 File — (ZIP) [file pone.0299045.s001.zip › statistical analysis/DWI╩2╛▌/DOR.bmp]

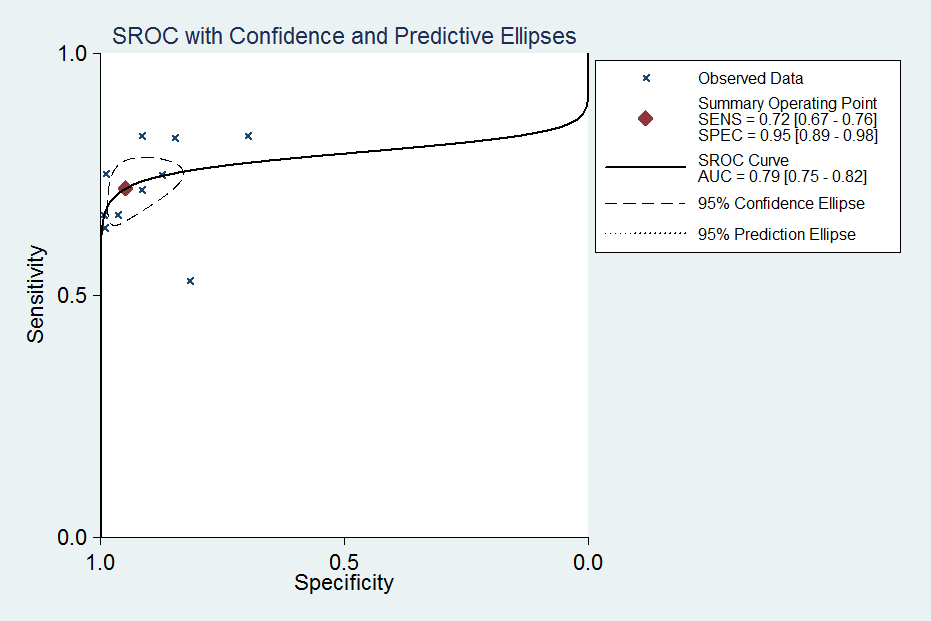

Supplement: S1 File — (ZIP) [file pone.0299045.s001.zip › statistical analysis/DWI╩2╛▌/Graph--SROC.tif]

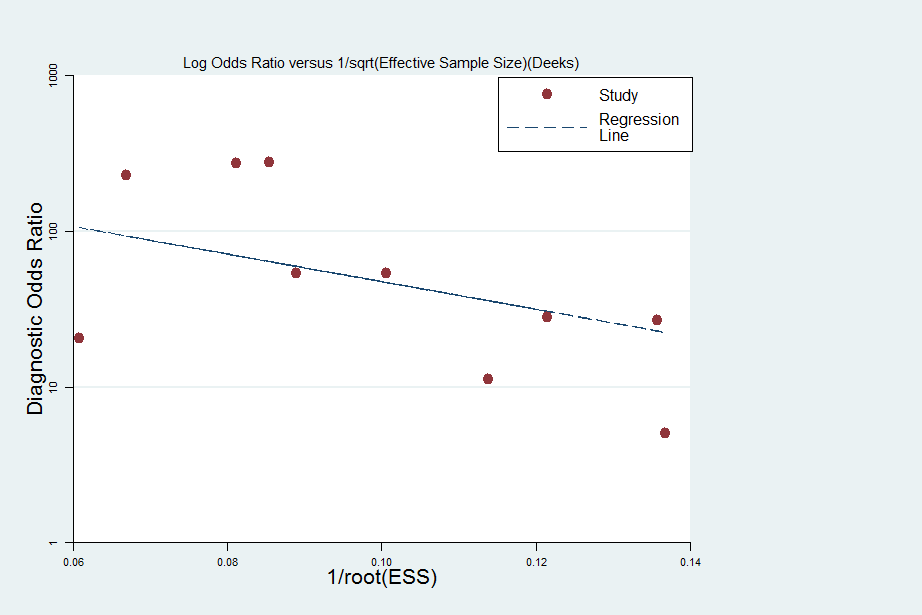

Supplement: S1 File — (ZIP) [file pone.0299045.s001.zip › statistical analysis/DWI╩2╛▌/graph--╖ó▒φ╞1⁄2╥╨.tif]

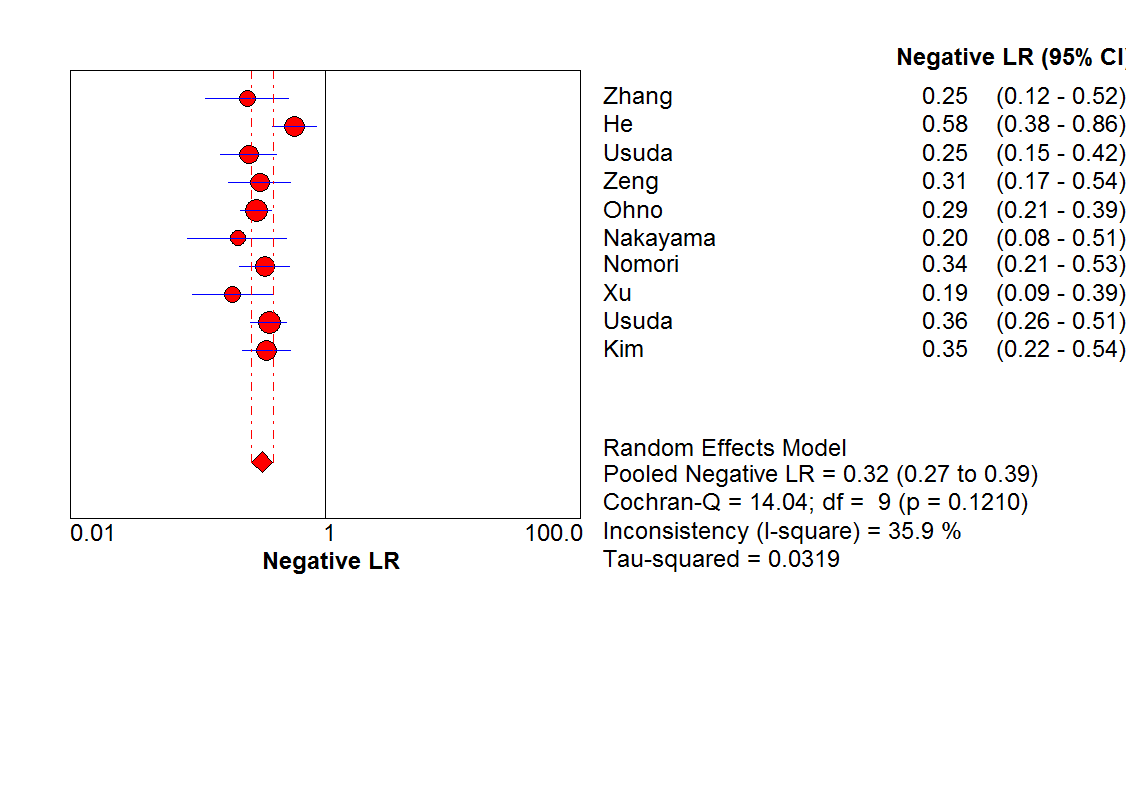

Supplement: S1 File — (ZIP) [file pone.0299045.s001.zip › statistical analysis/DWI╩2╛▌/NLR.bmp]

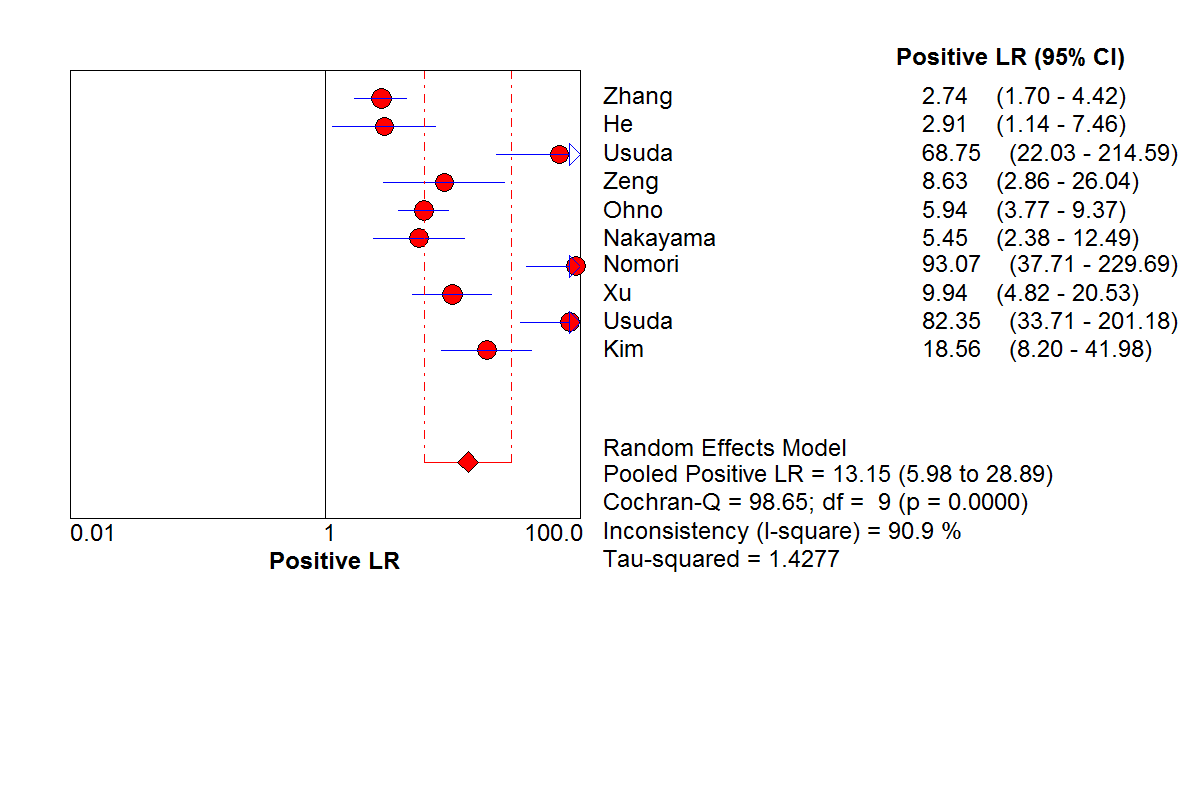

Supplement: S1 File — (ZIP) [file pone.0299045.s001.zip › statistical analysis/DWI╩2╛▌/PLR.bmp]

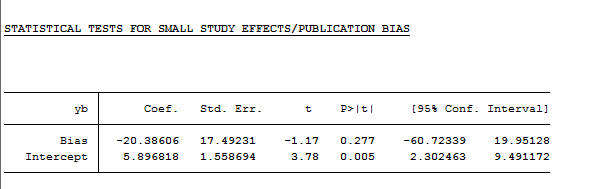

Supplement: S1 File — (ZIP) [file pone.0299045.s001.zip › statistical analysis/DWI╩2╛▌/QQ═╝╞1⁄420151220143043.png]

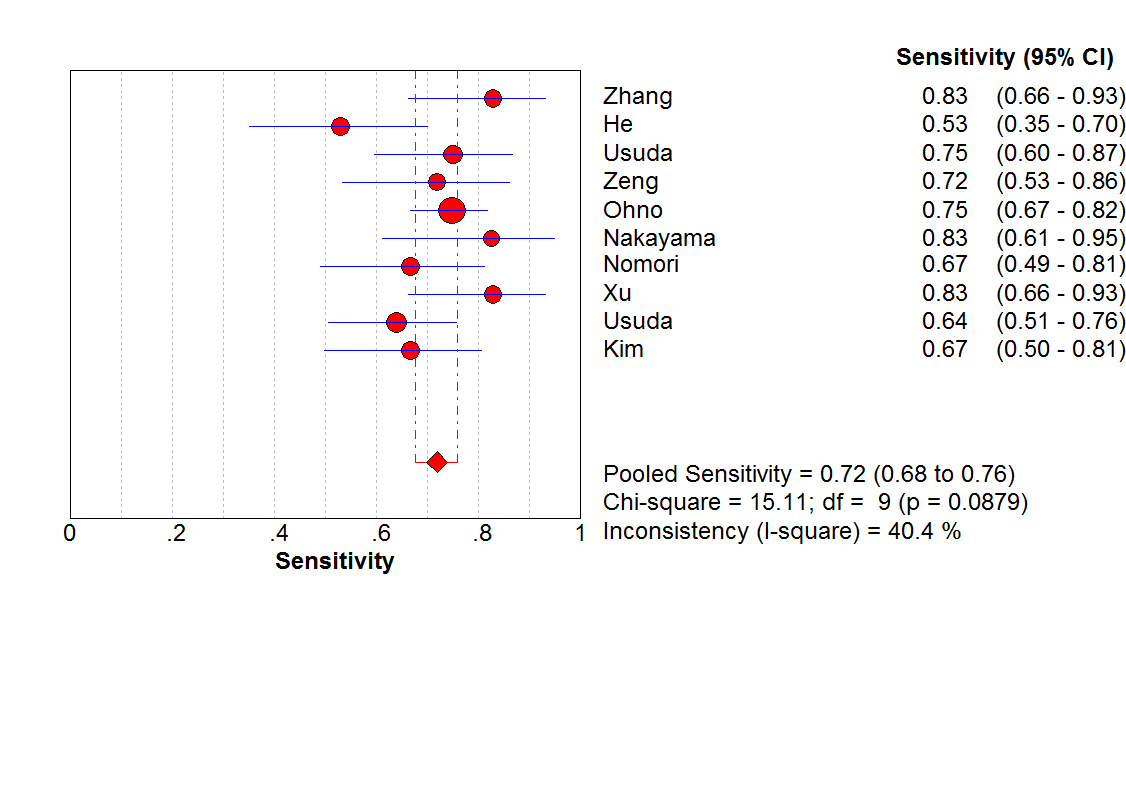

Supplement: S1 File — (ZIP) [file pone.0299045.s001.zip › statistical analysis/DWI╩2╛▌/SEN.bmp]

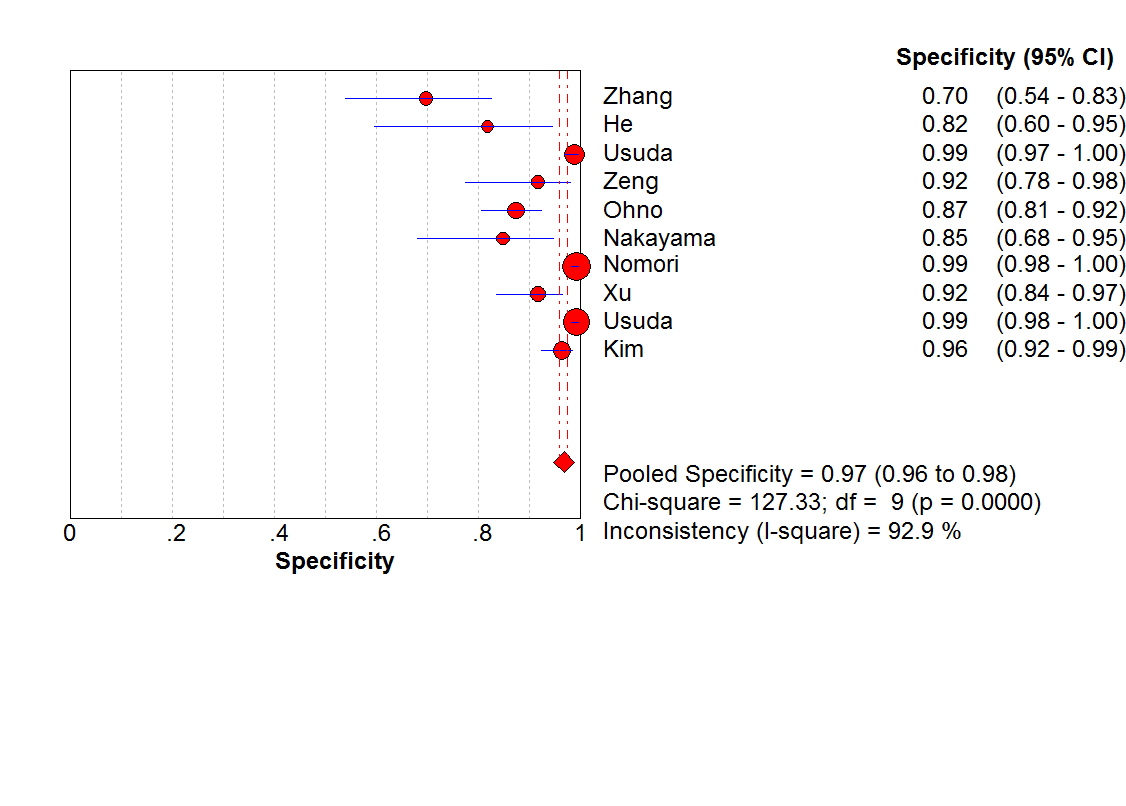

Supplement: S1 File — (ZIP) [file pone.0299045.s001.zip › statistical analysis/DWI╩2╛▌/SPE.bmp]

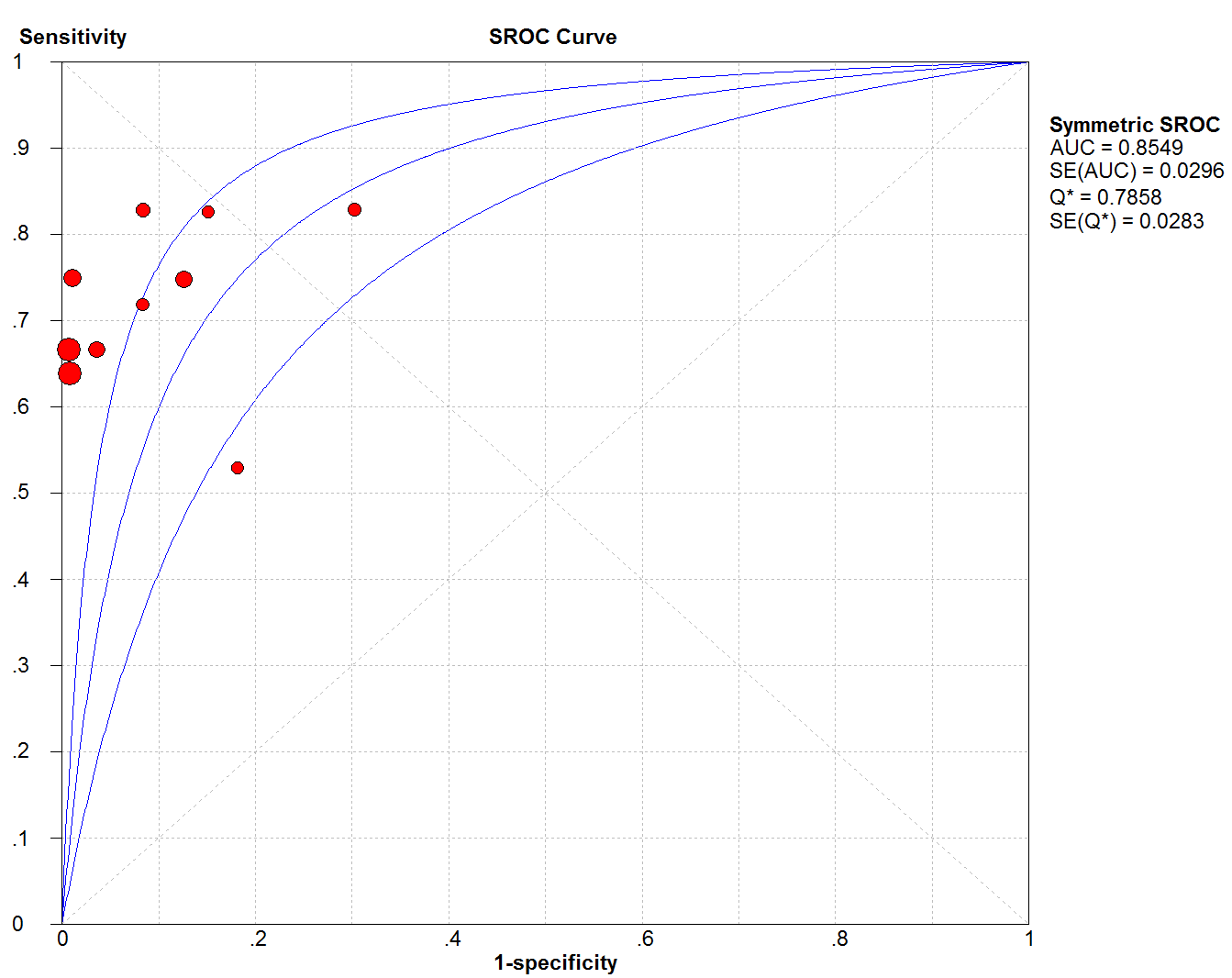

Supplement: S1 File — (ZIP) [file pone.0299045.s001.zip › statistical analysis/DWI╩2╛▌/SROC.bmp]

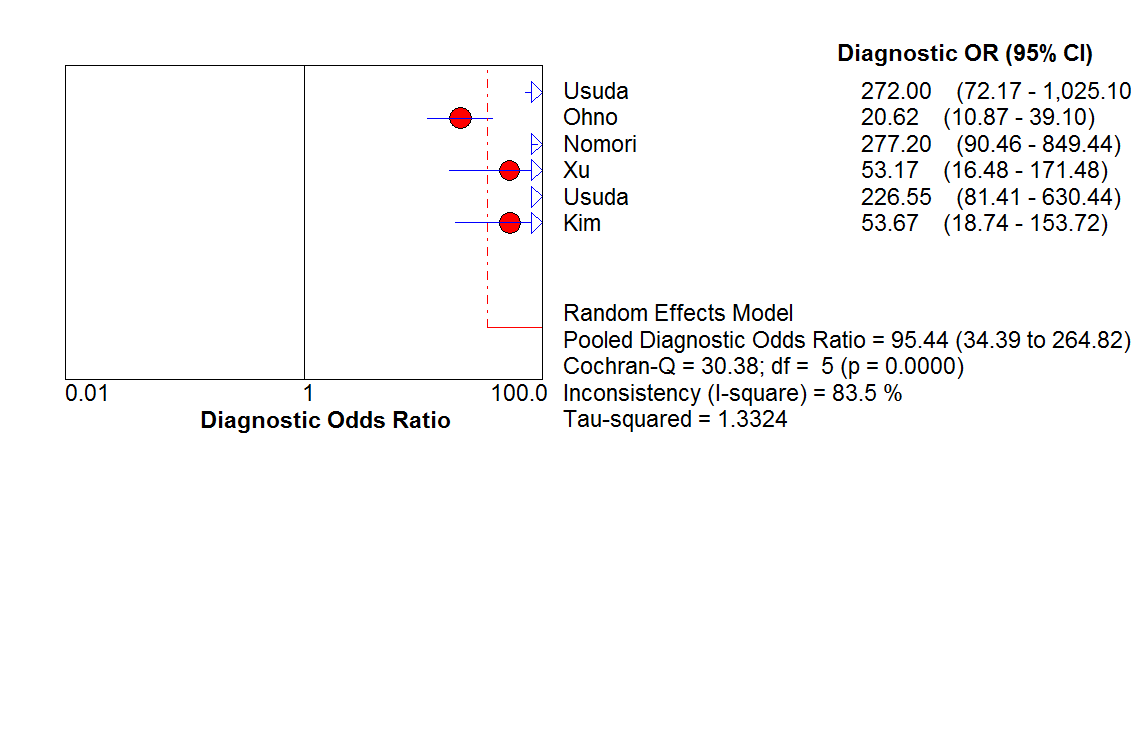

Supplement: S1 File — (ZIP) [file pone.0299045.s001.zip › statistical analysis/DWI╩2╛▌/╤╟╫Θ╖╓╬÷/design prospective/dor.bmp]

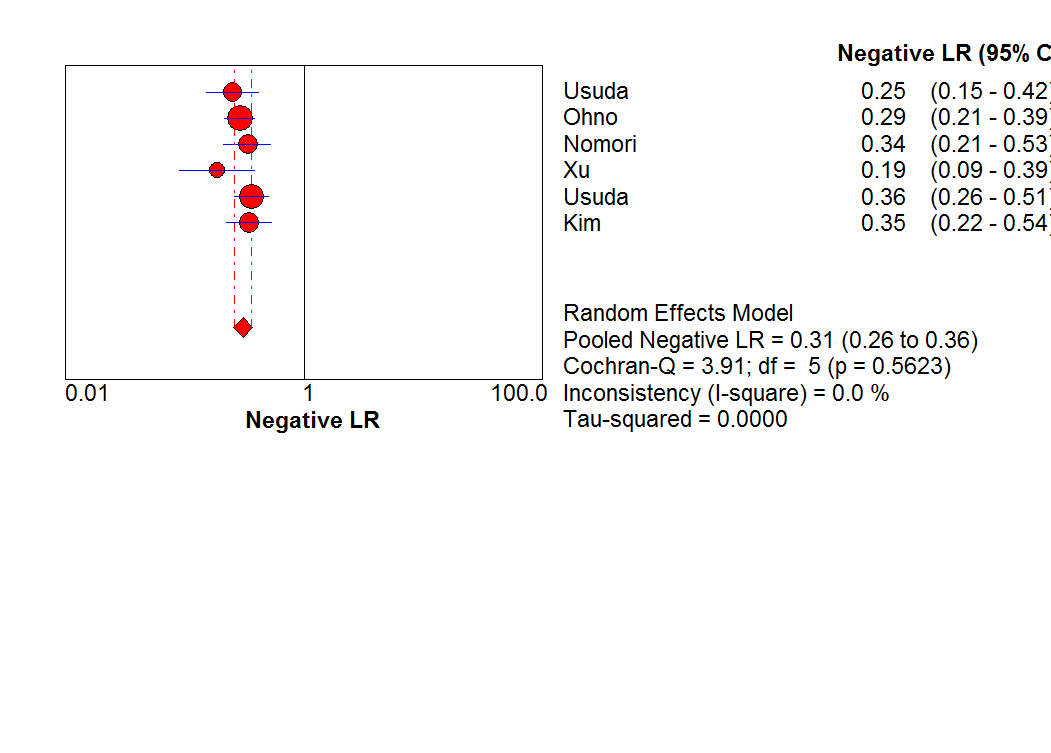

Supplement: S1 File — (ZIP) [file pone.0299045.s001.zip › statistical analysis/DWI╩2╛▌/╤╟╫Θ╖╓╬÷/design prospective/nlr.bmp]

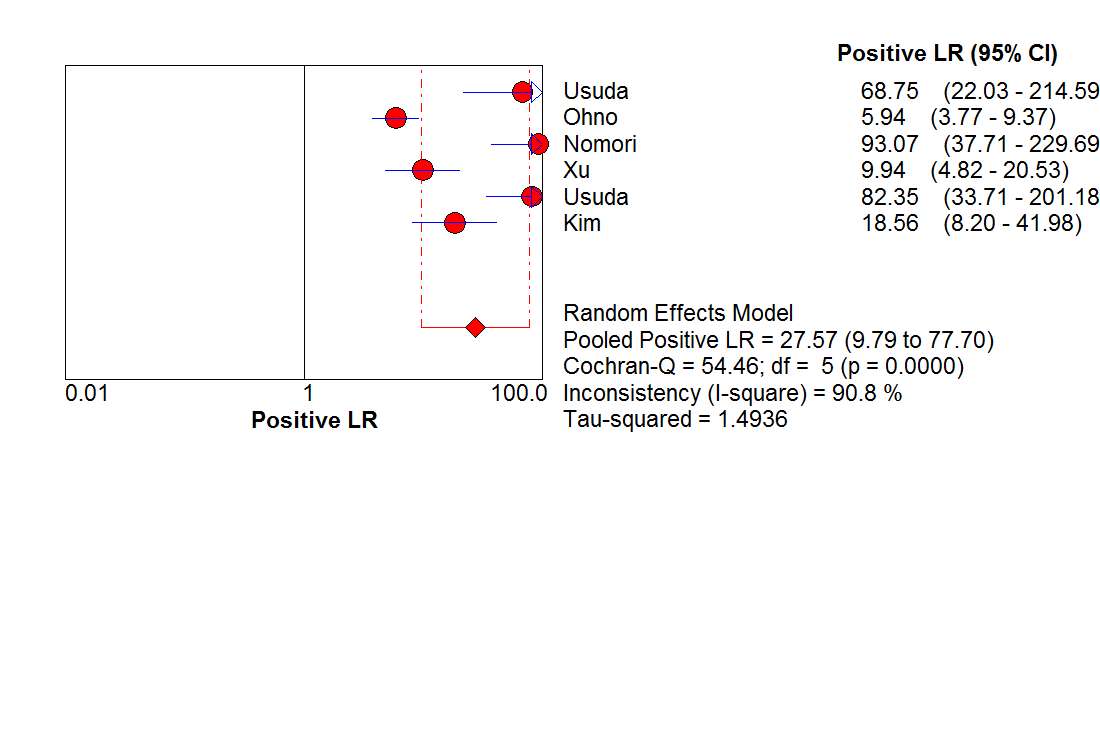

Supplement: S1 File — (ZIP) [file pone.0299045.s001.zip › statistical analysis/DWI╩2╛▌/╤╟╫Θ╖╓╬÷/design prospective/plr.bmp]

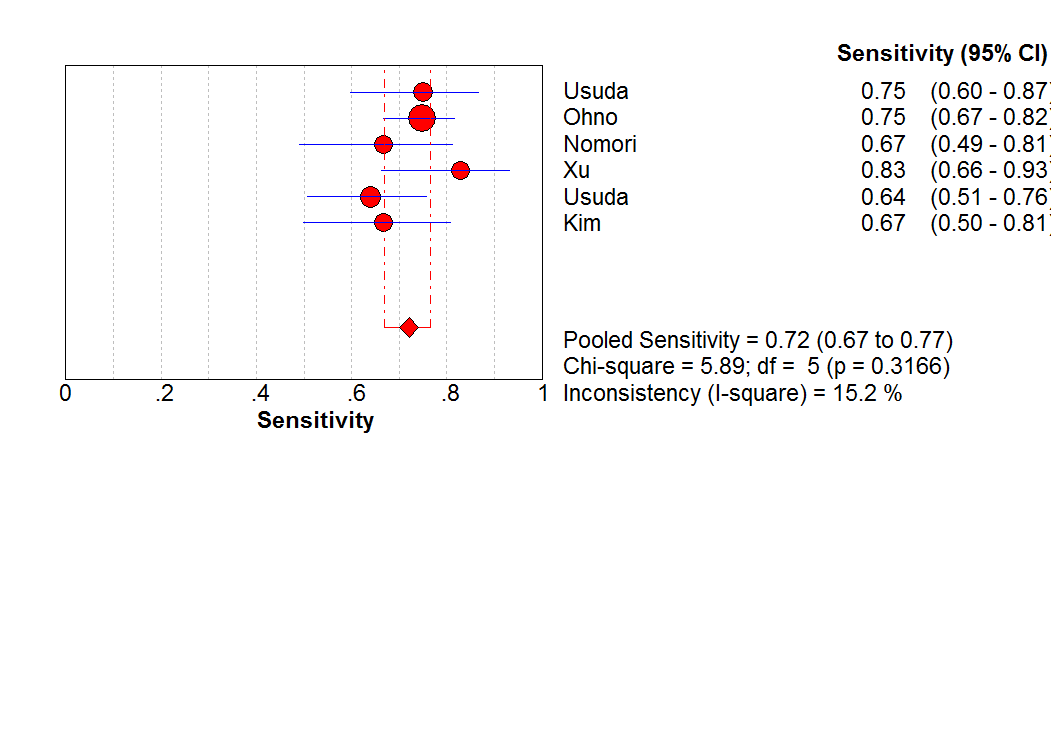

Supplement: S1 File — (ZIP) [file pone.0299045.s001.zip › statistical analysis/DWI╩2╛▌/╤╟╫Θ╖╓╬÷/design prospective/sen.bmp]

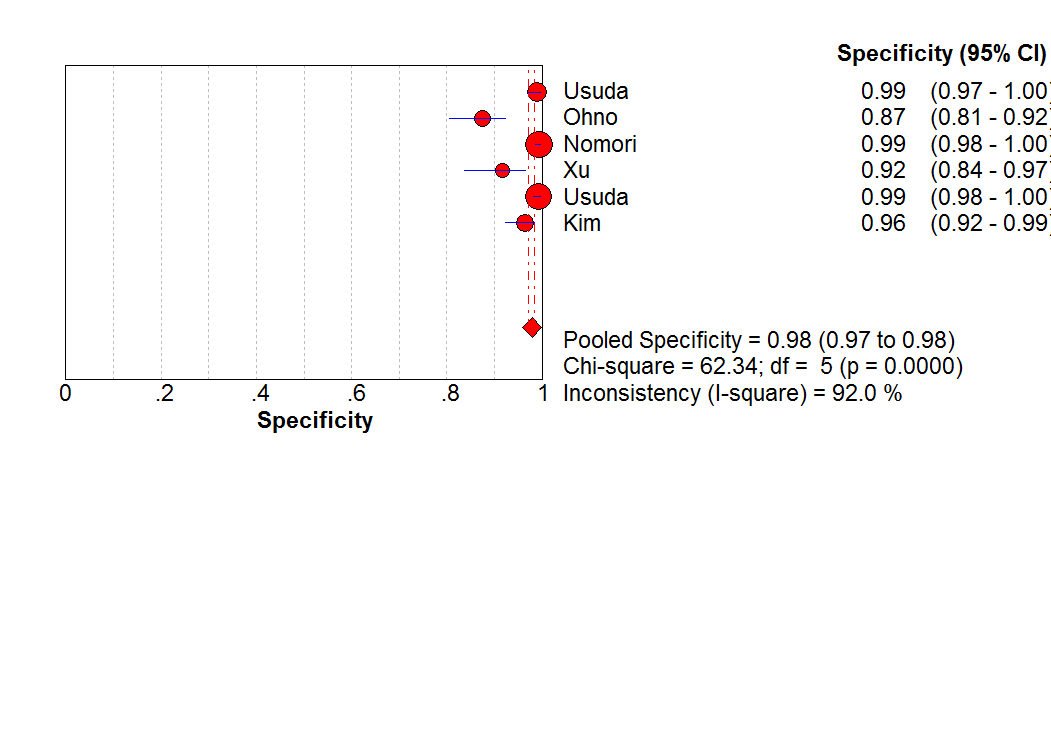

Supplement: S1 File — (ZIP) [file pone.0299045.s001.zip › statistical analysis/DWI╩2╛▌/╤╟╫Θ╖╓╬÷/design prospective/spe.bmp]

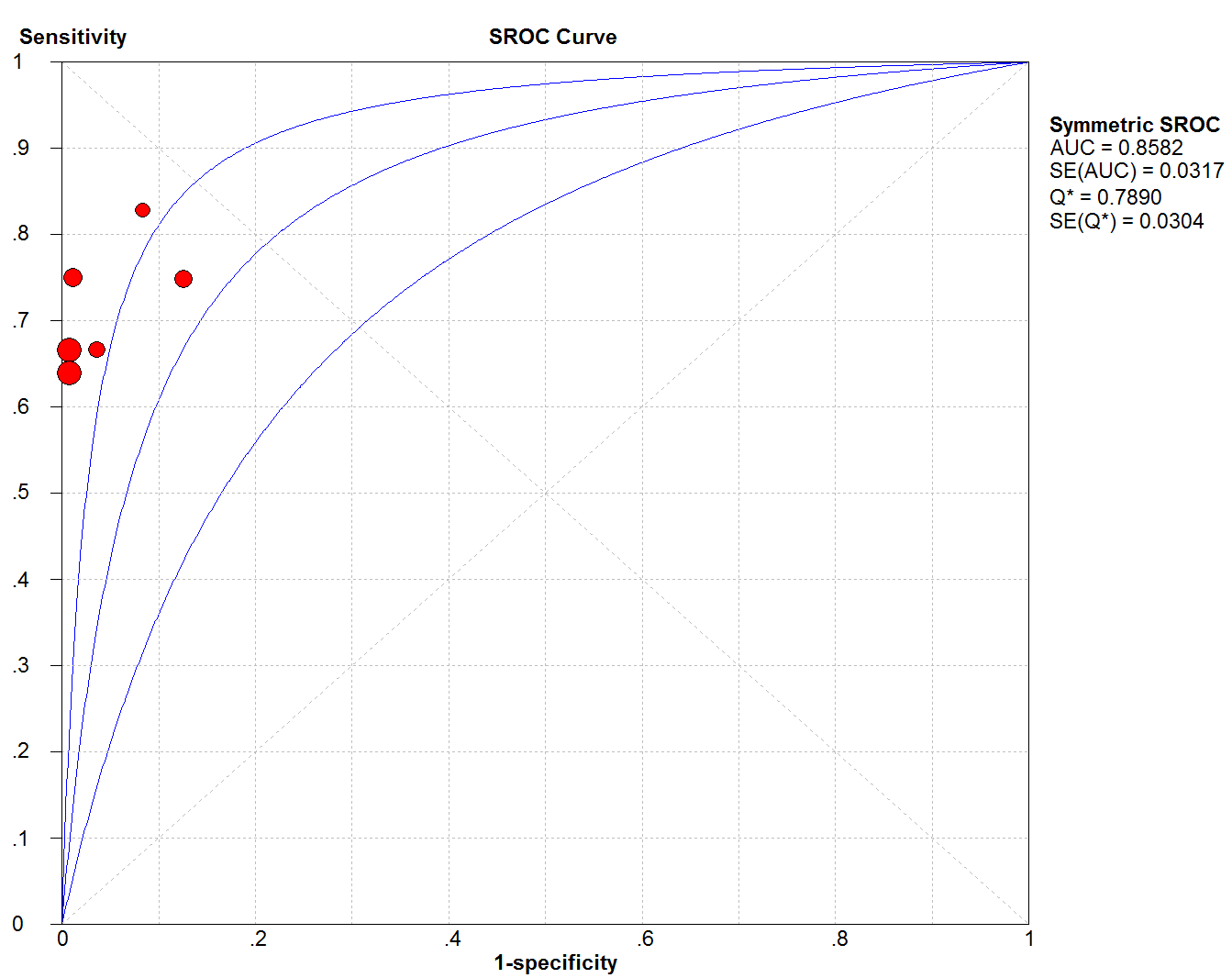

Supplement: S1 File — (ZIP) [file pone.0299045.s001.zip › statistical analysis/DWI╩2╛▌/╤╟╫Θ╖╓╬÷/design prospective/sroc.bmp]

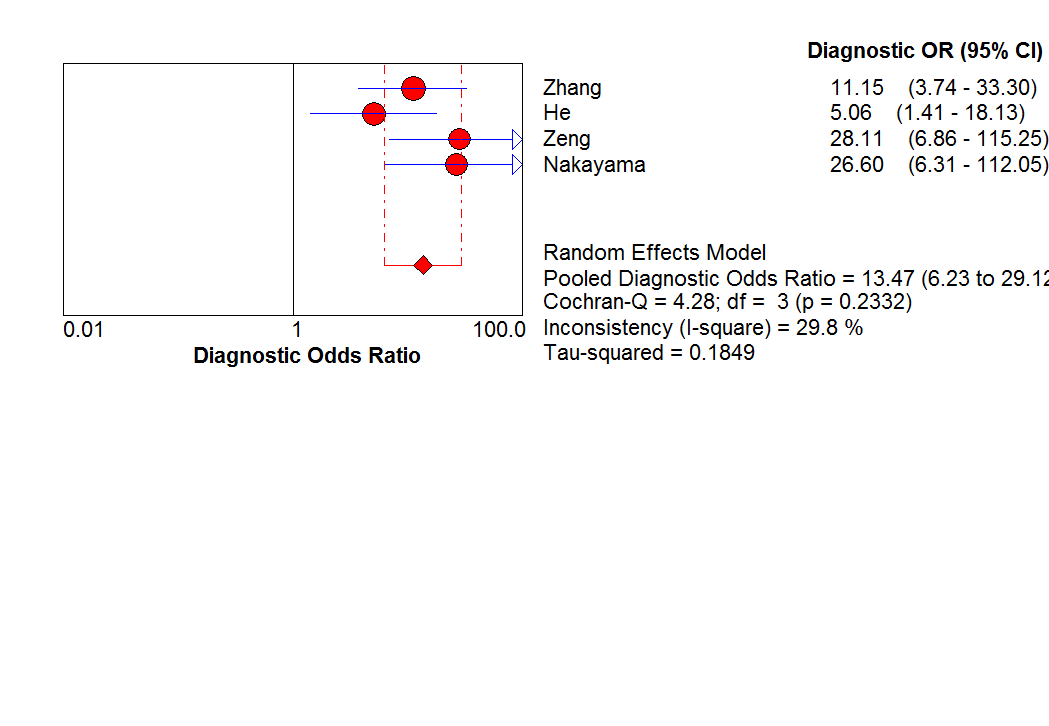

Supplement: S1 File — (ZIP) [file pone.0299045.s001.zip › statistical analysis/DWI╩2╛▌/╤╟╫Θ╖╓╬÷/design retrospective/dor.bmp]

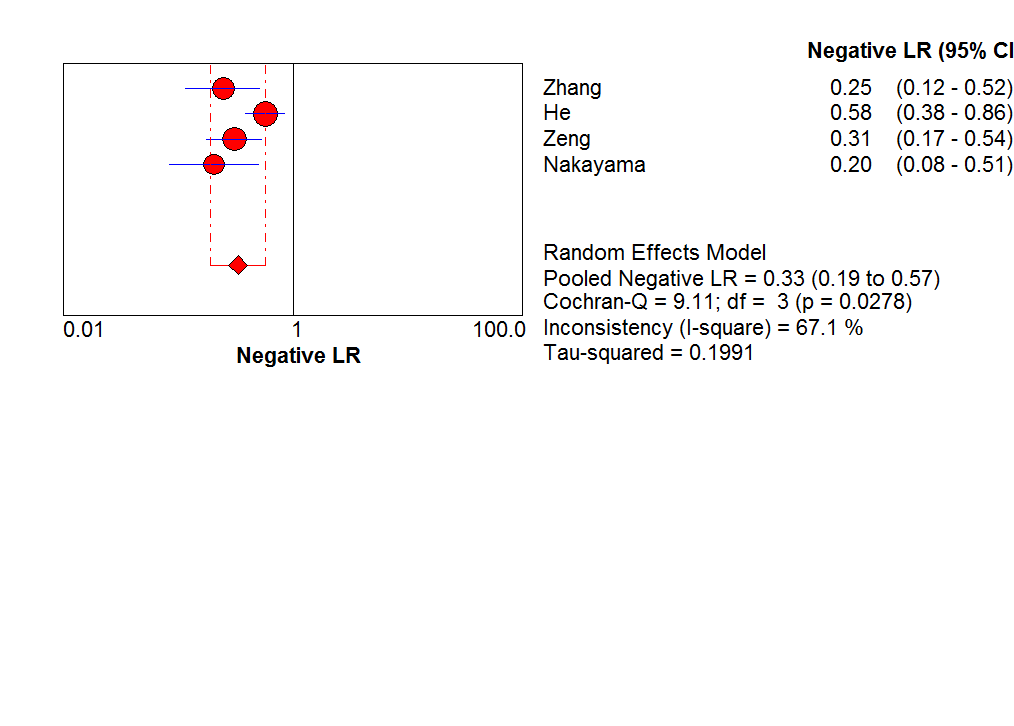

Supplement: S1 File — (ZIP) [file pone.0299045.s001.zip › statistical analysis/DWI╩2╛▌/╤╟╫Θ╖╓╬÷/design retrospective/nlr.bmp]

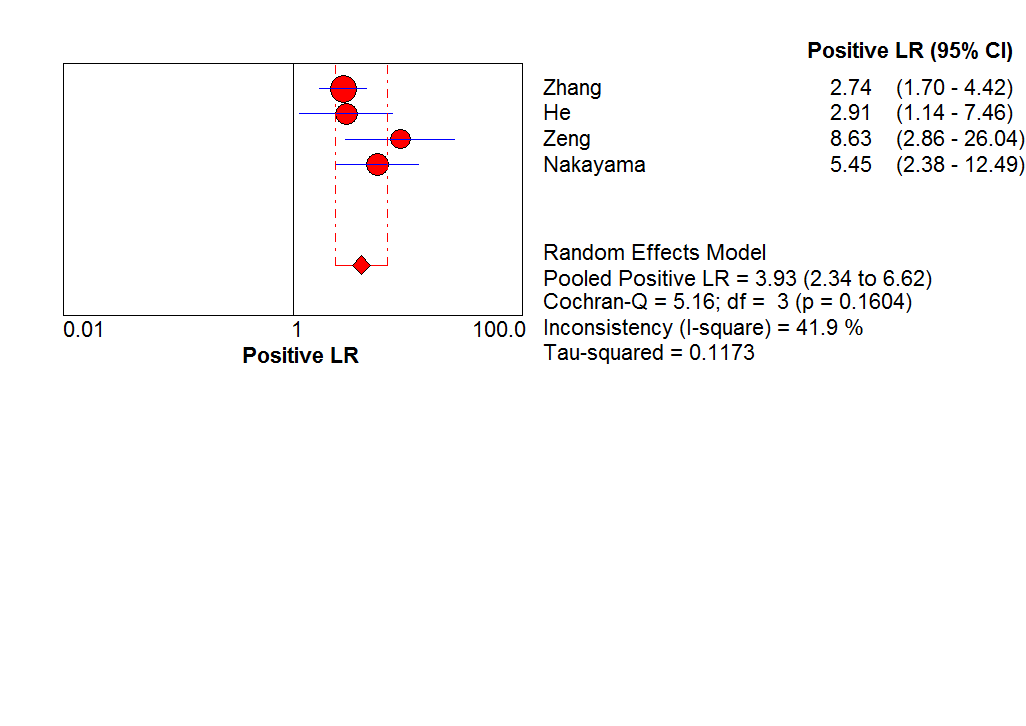

Supplement: S1 File — (ZIP) [file pone.0299045.s001.zip › statistical analysis/DWI╩2╛▌/╤╟╫Θ╖╓╬÷/design retrospective/plr.bmp]

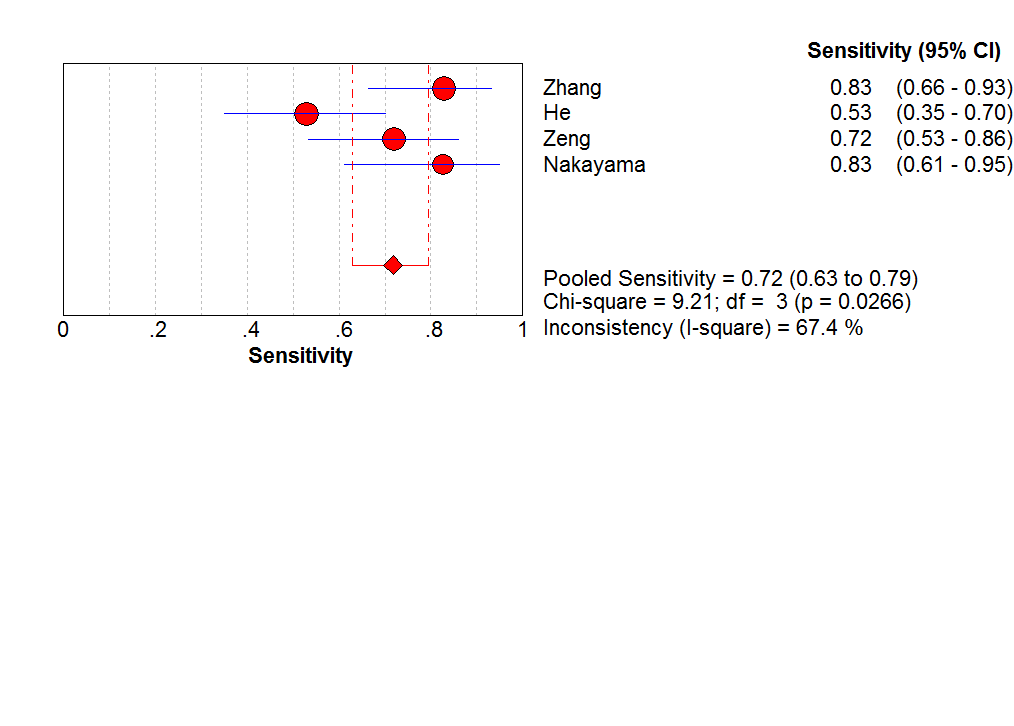

Supplement: S1 File — (ZIP) [file pone.0299045.s001.zip › statistical analysis/DWI╩2╛▌/╤╟╫Θ╖╓╬÷/design retrospective/sen.bmp]

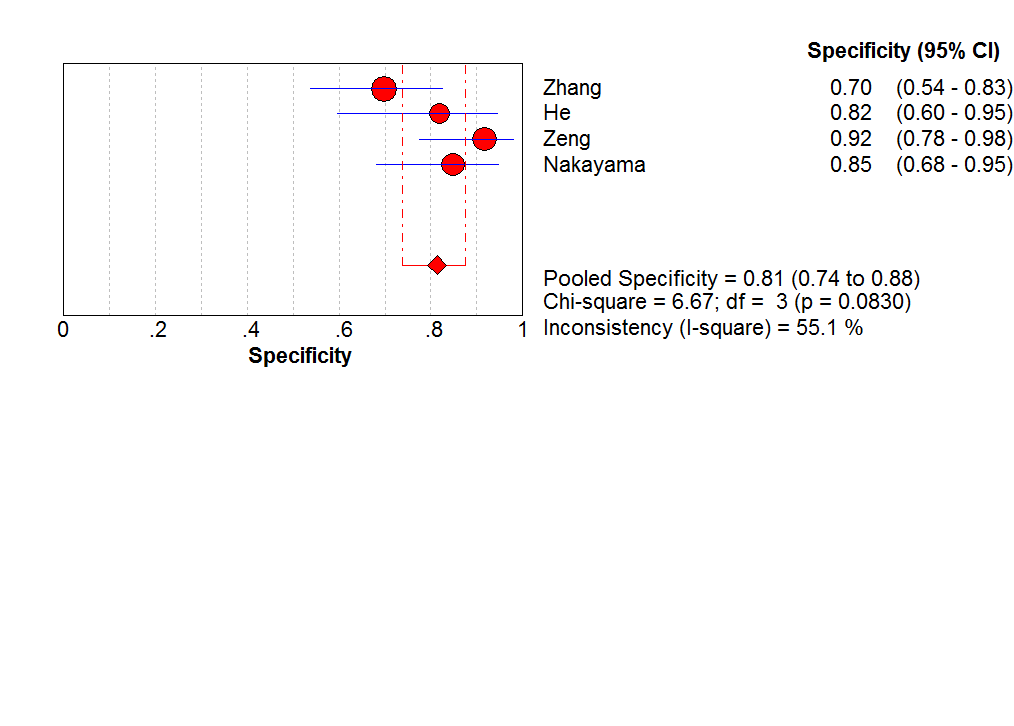

Supplement: S1 File — (ZIP) [file pone.0299045.s001.zip › statistical analysis/DWI╩2╛▌/╤╟╫Θ╖╓╬÷/design retrospective/spe.bmp]

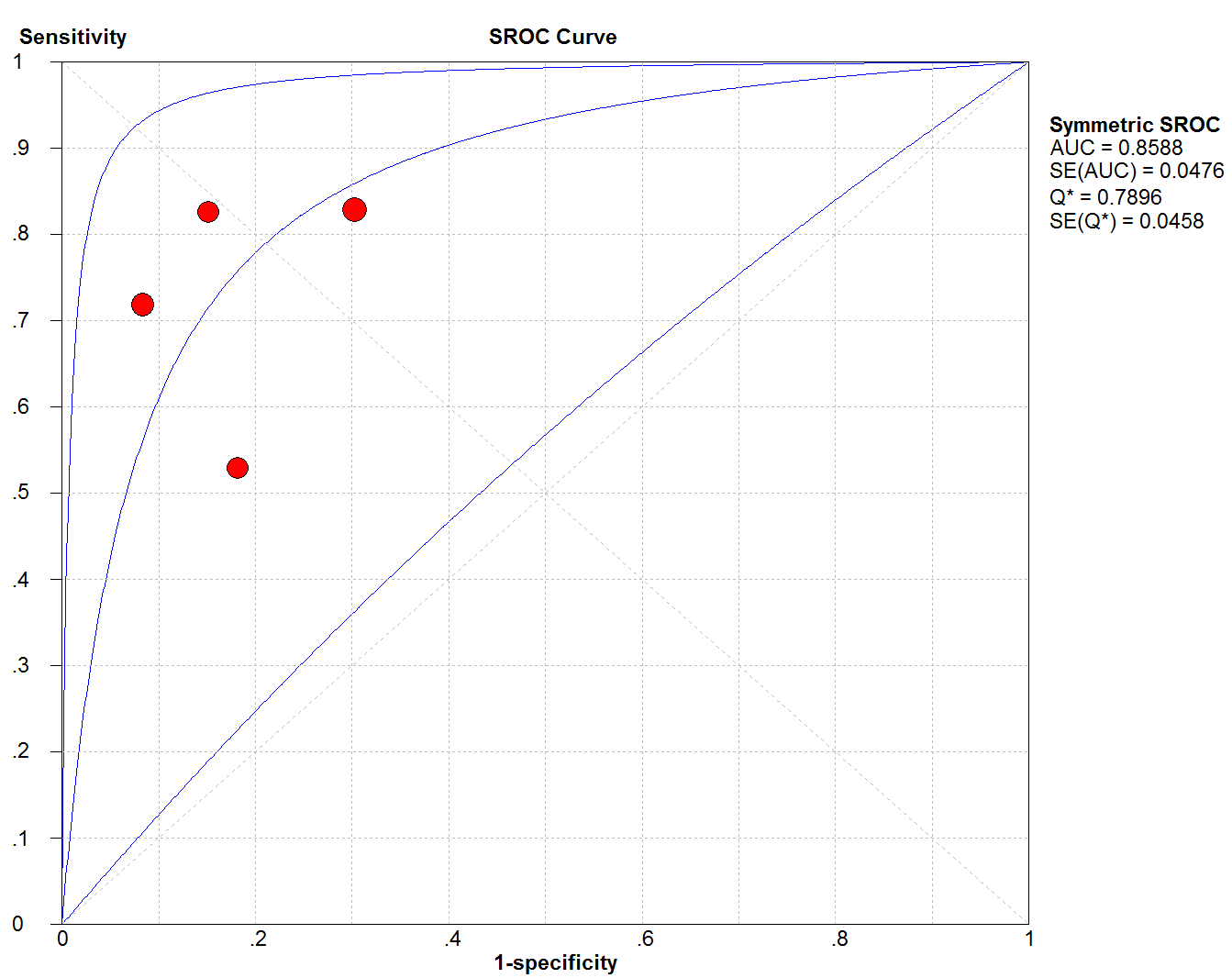

Supplement: S1 File — (ZIP) [file pone.0299045.s001.zip › statistical analysis/DWI╩2╛▌/╤╟╫Θ╖╓╬÷/design retrospective/sroc.bmp]

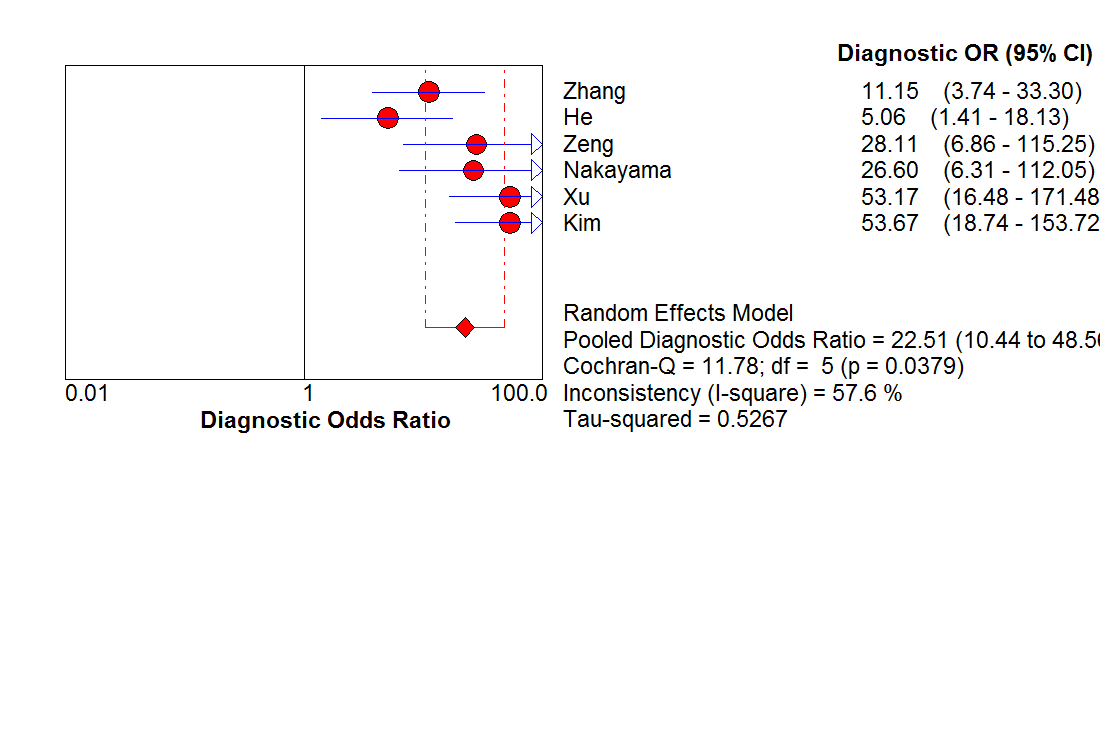

Supplement: S1 File — (ZIP) [file pone.0299045.s001.zip › statistical analysis/DWI╩2╛▌/╤╟╫Θ╖╓╬÷/sample sizeí╢250/dor.bmp]

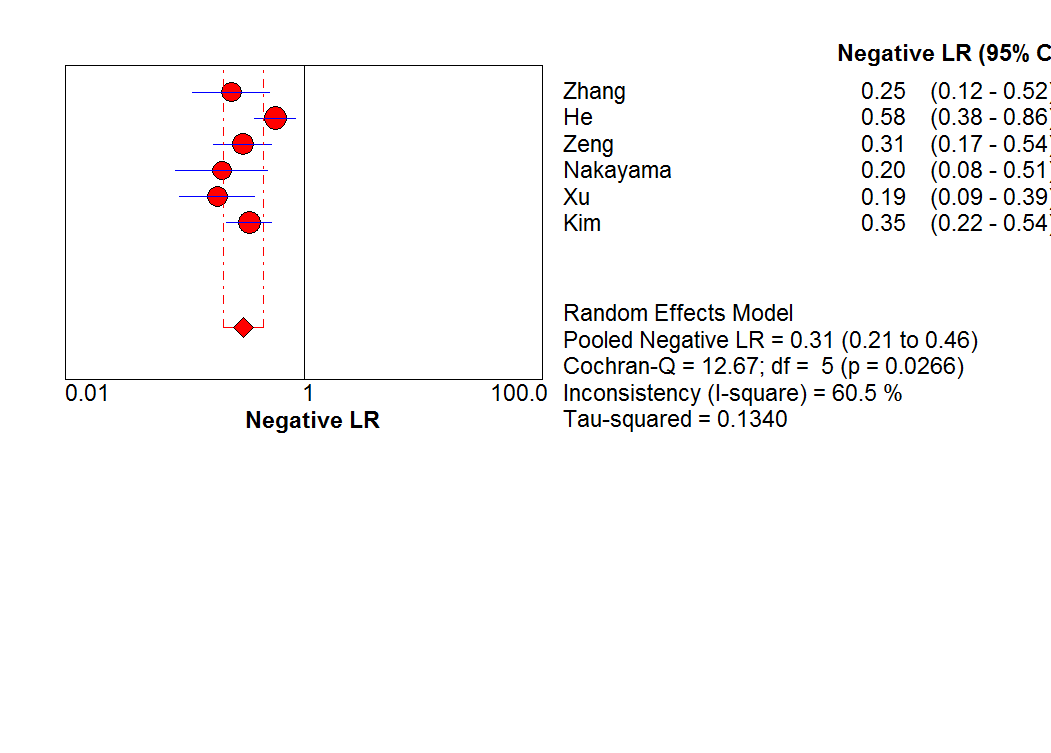

Supplement: S1 File — (ZIP) [file pone.0299045.s001.zip › statistical analysis/DWI╩2╛▌/╤╟╫Θ╖╓╬÷/sample sizeí╢250/nlr.bmp]

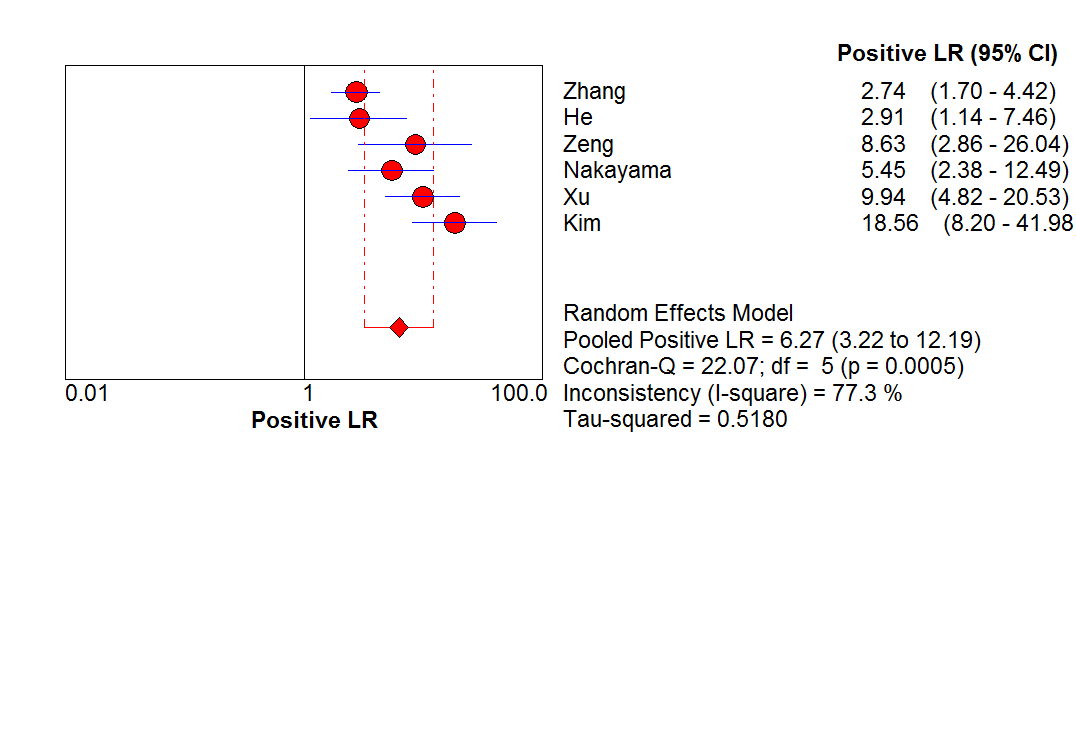

Supplement: S1 File — (ZIP) [file pone.0299045.s001.zip › statistical analysis/DWI╩2╛▌/╤╟╫Θ╖╓╬÷/sample sizeí╢250/plr.bmp]

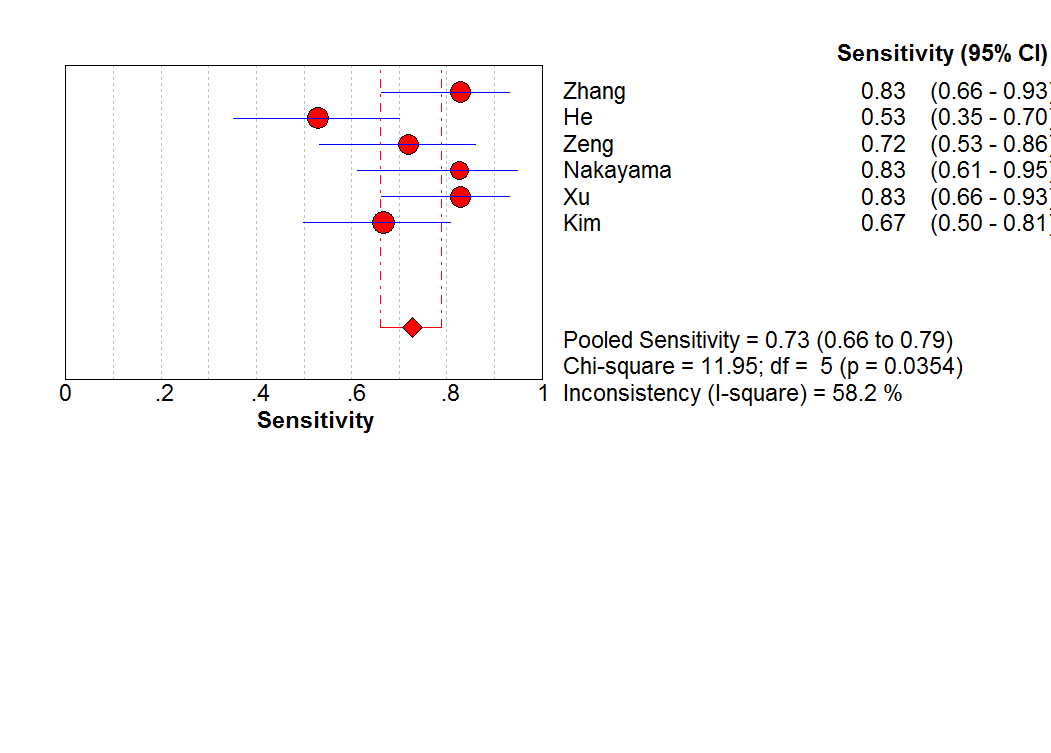

Supplement: S1 File — (ZIP) [file pone.0299045.s001.zip › statistical analysis/DWI╩2╛▌/╤╟╫Θ╖╓╬÷/sample sizeí╢250/sen.bmp]

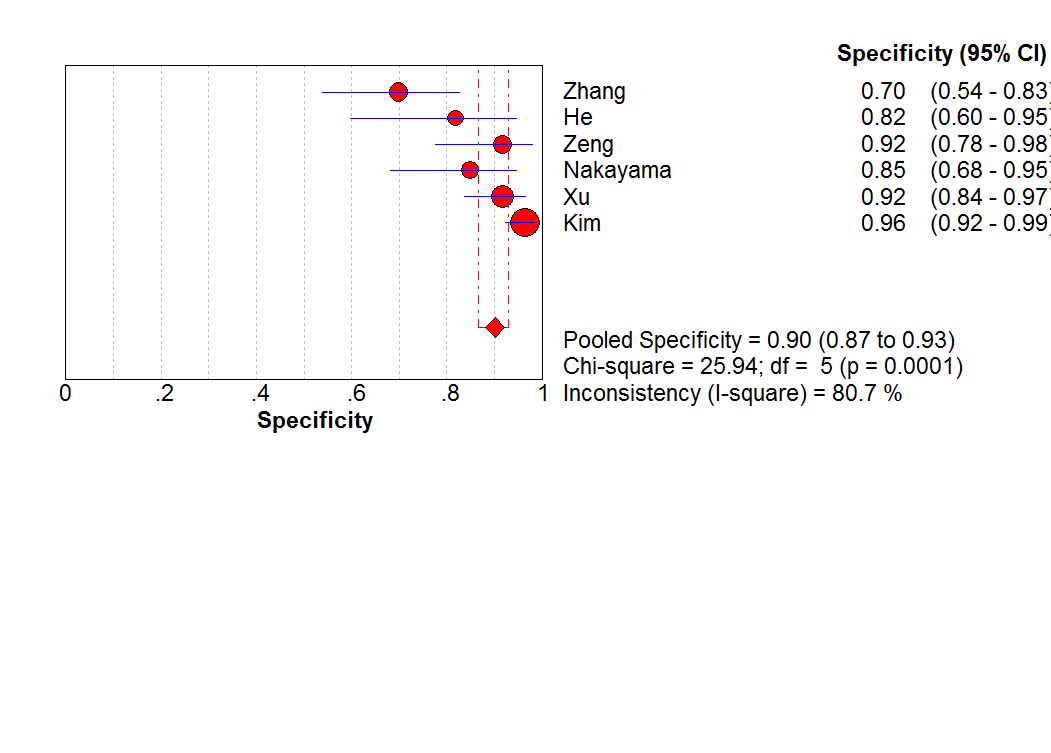

Supplement: S1 File — (ZIP) [file pone.0299045.s001.zip › statistical analysis/DWI╩2╛▌/╤╟╫Θ╖╓╬÷/sample sizeí╢250/spe.bmp]

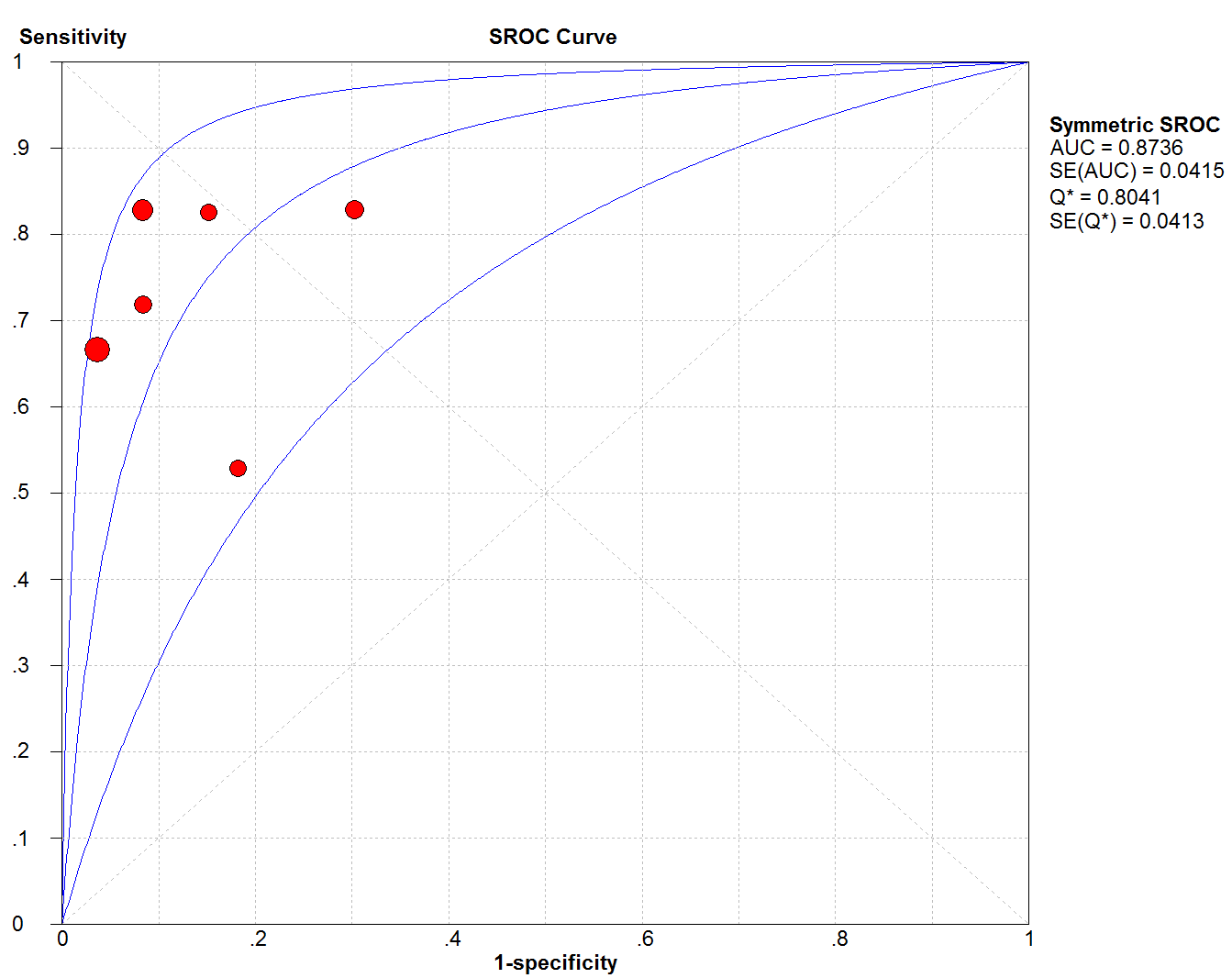

Supplement: S1 File — (ZIP) [file pone.0299045.s001.zip › statistical analysis/DWI╩2╛▌/╤╟╫Θ╖╓╬÷/sample sizeí╢250/sroc.bmp]

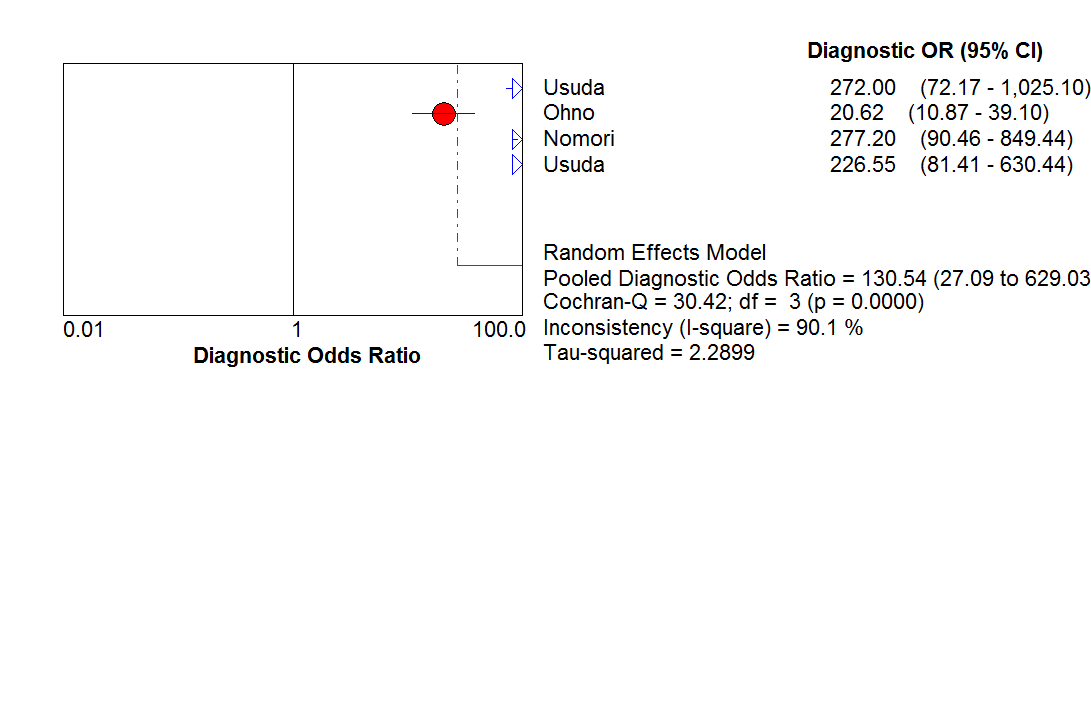

Supplement: S1 File — (ZIP) [file pone.0299045.s001.zip › statistical analysis/DWI╩2╛▌/╤╟╫Θ╖╓╬÷/sample sizeí╖250/dor.bmp]

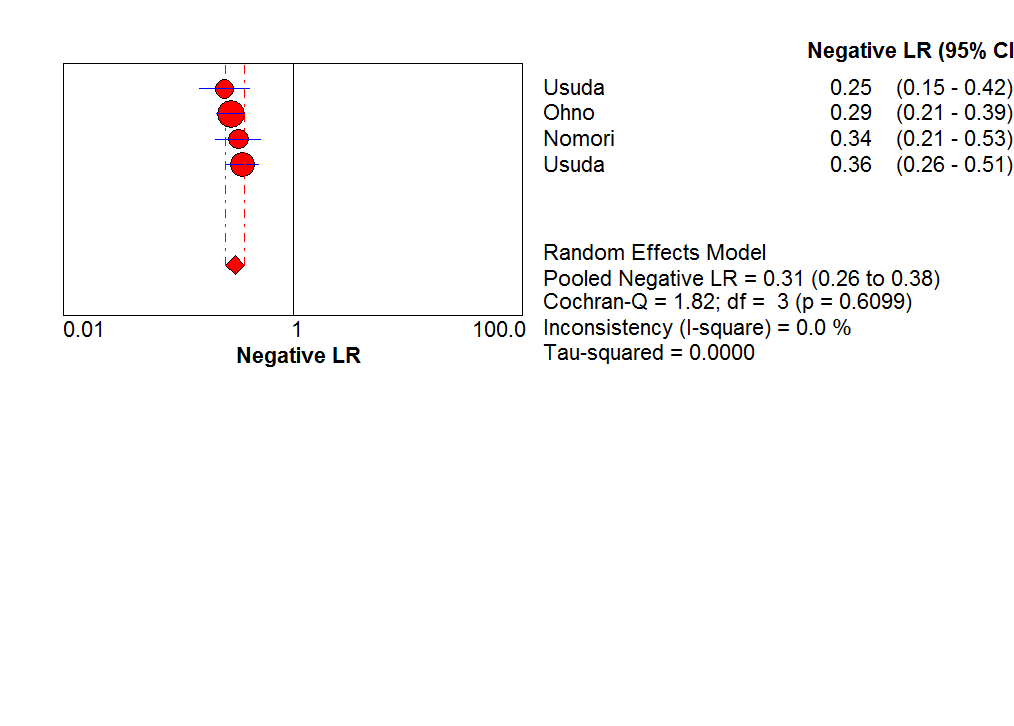

Supplement: S1 File — (ZIP) [file pone.0299045.s001.zip › statistical analysis/DWI╩2╛▌/╤╟╫Θ╖╓╬÷/sample sizeí╖250/nlr.bmp]

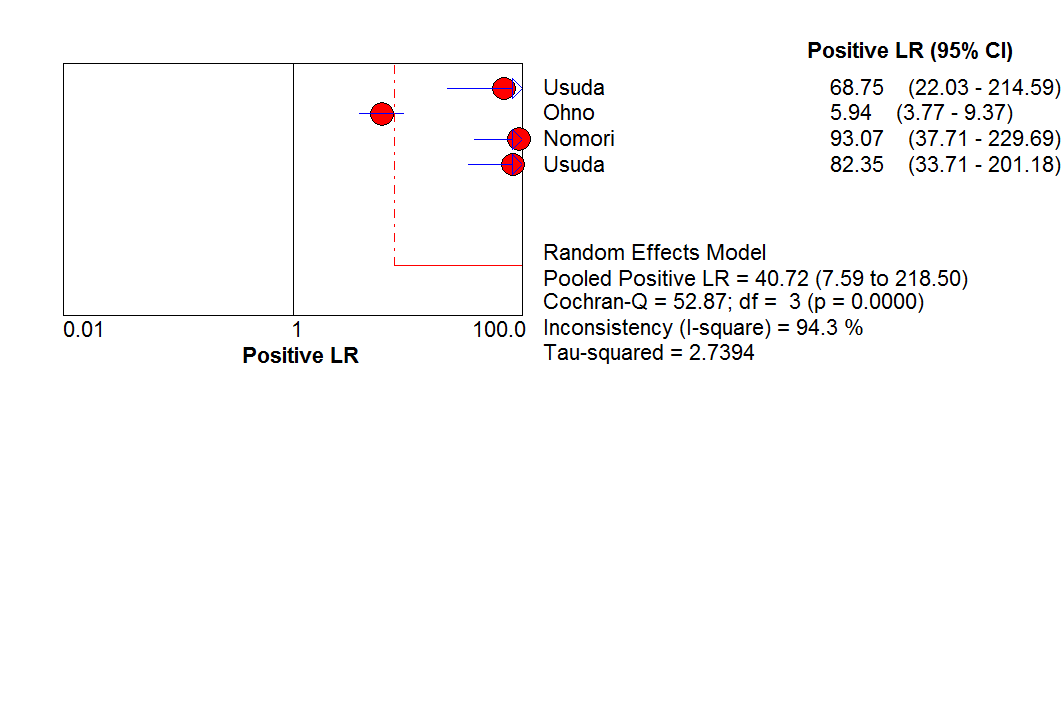

Supplement: S1 File — (ZIP) [file pone.0299045.s001.zip › statistical analysis/DWI╩2╛▌/╤╟╫Θ╖╓╬÷/sample sizeí╖250/plr.bmp]

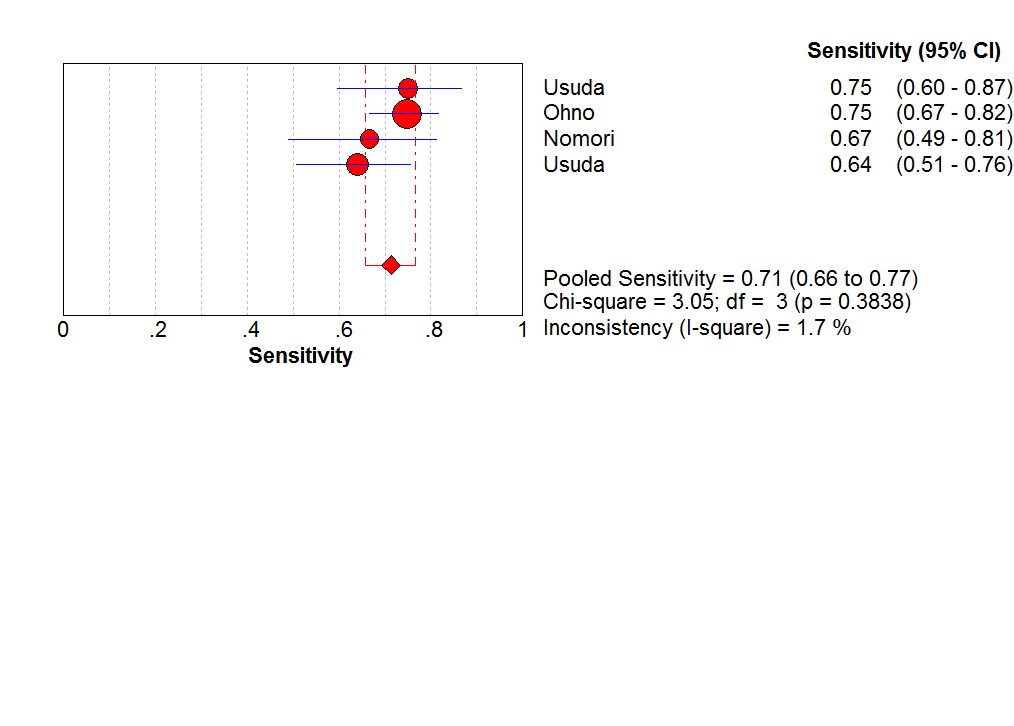

Supplement: S1 File — (ZIP) [file pone.0299045.s001.zip › statistical analysis/DWI╩2╛▌/╤╟╫Θ╖╓╬÷/sample sizeí╖250/sen.bmp]

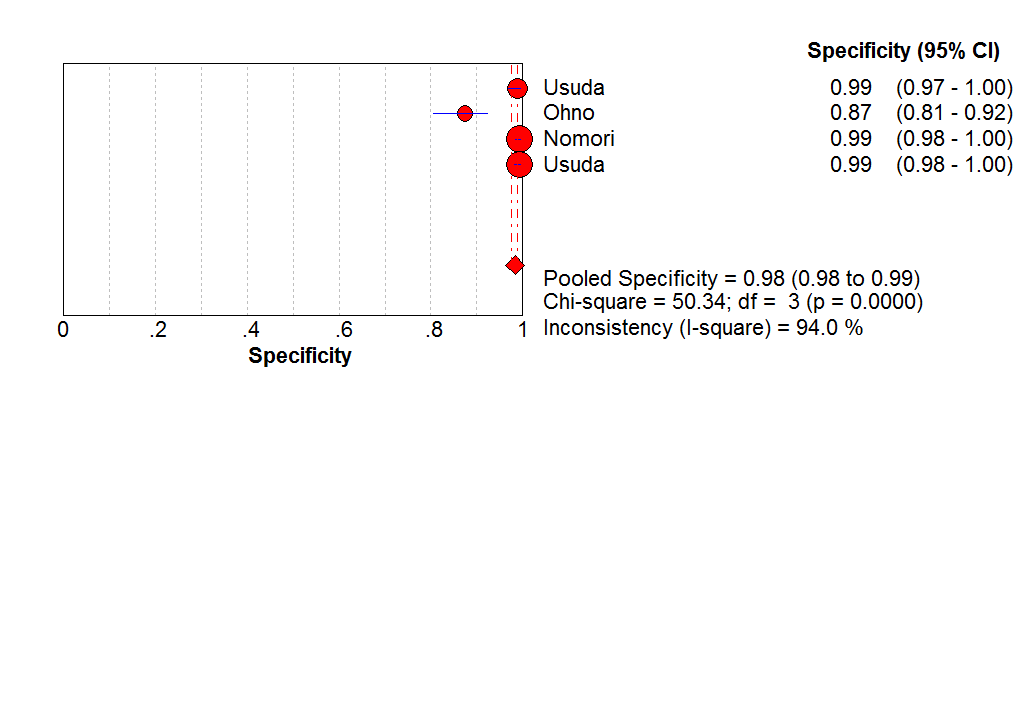

Supplement: S1 File — (ZIP) [file pone.0299045.s001.zip › statistical analysis/DWI╩2╛▌/╤╟╫Θ╖╓╬÷/sample sizeí╖250/spe.bmp]

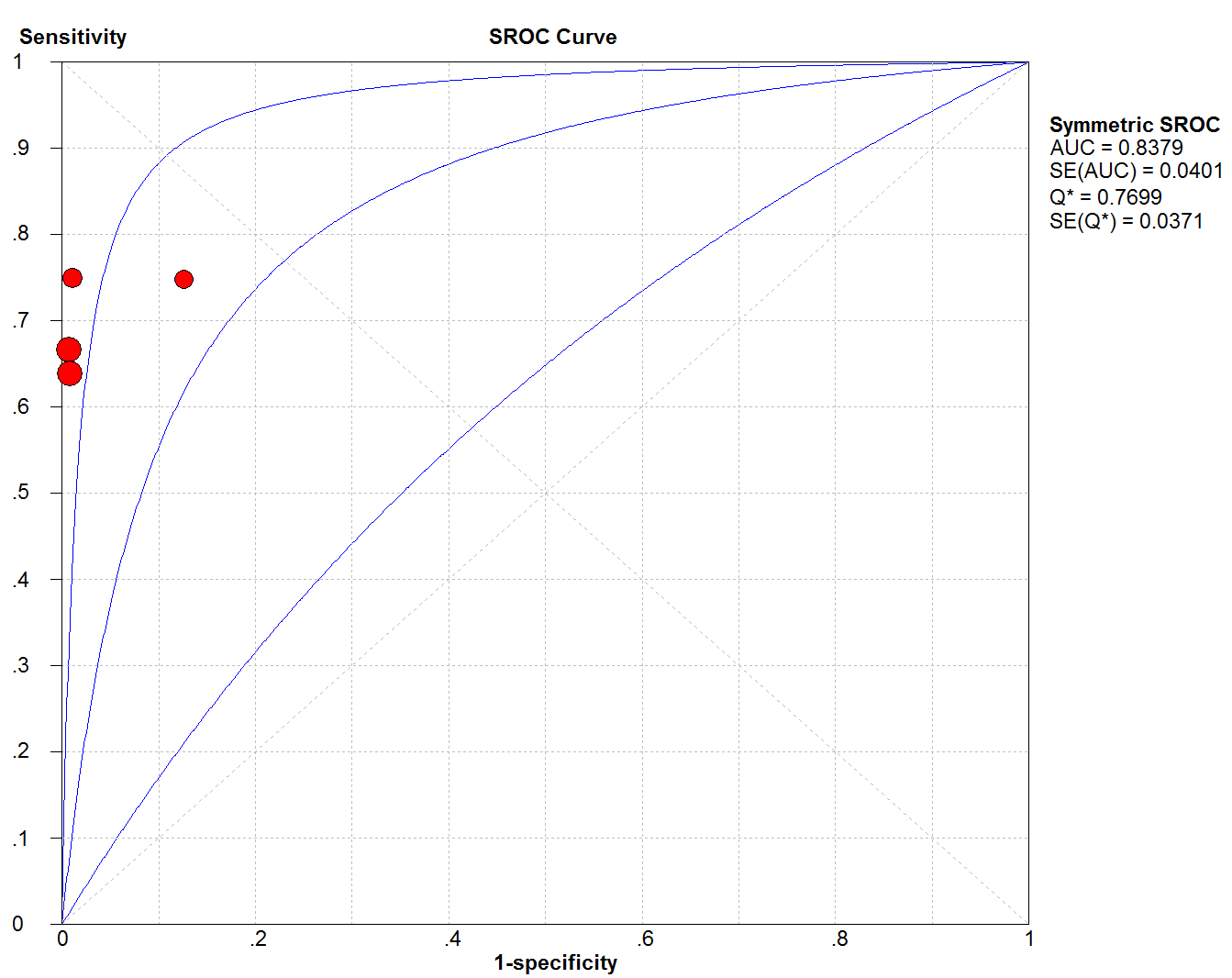

Supplement: S1 File — (ZIP) [file pone.0299045.s001.zip › statistical analysis/DWI╩2╛▌/╤╟╫Θ╖╓╬÷/sample sizeí╖250/sroc.bmp]

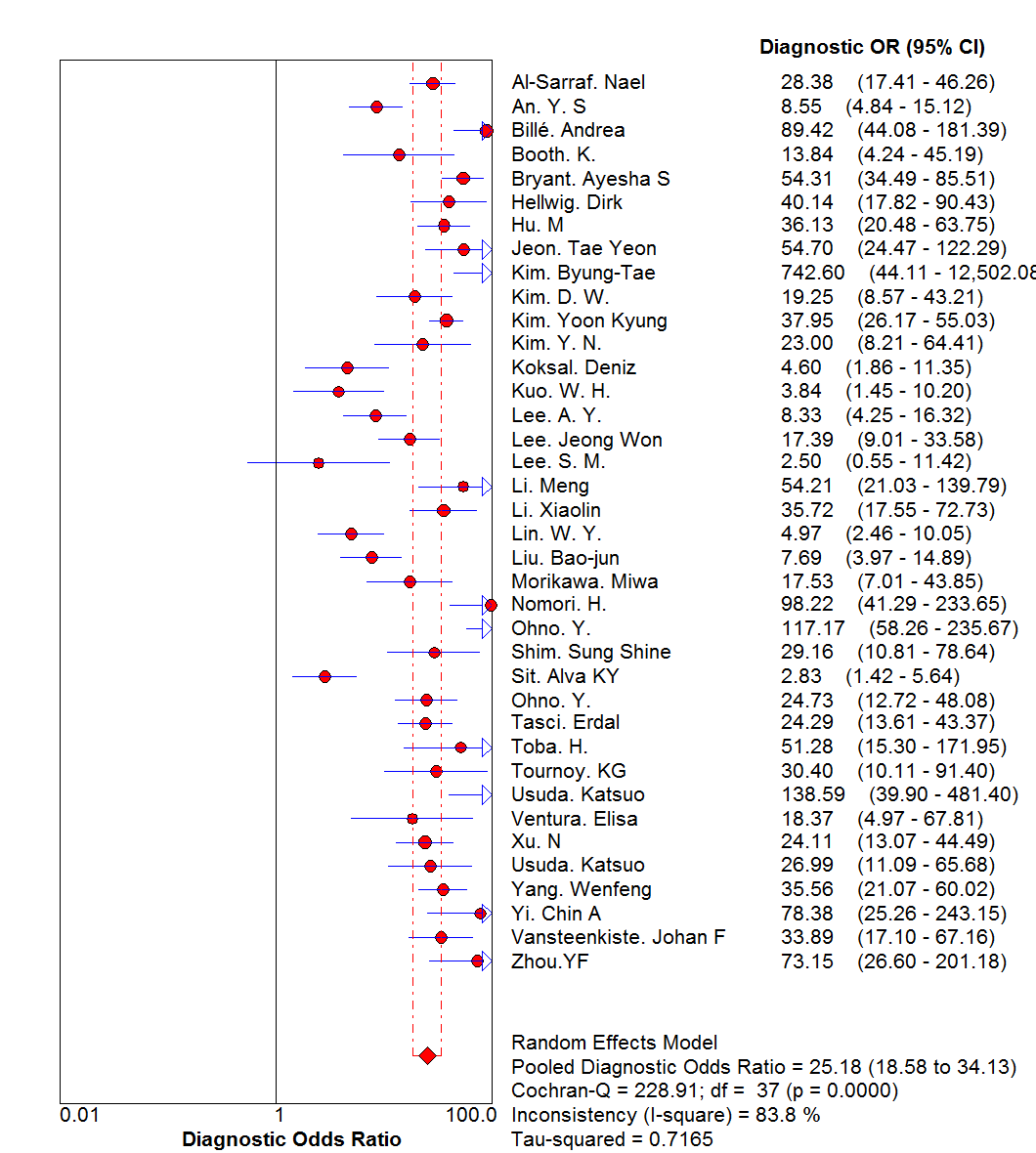

Supplement: S1 File — (ZIP) [file pone.0299045.s001.zip › statistical analysis/PET╩2╛▌/DOR.bmp]

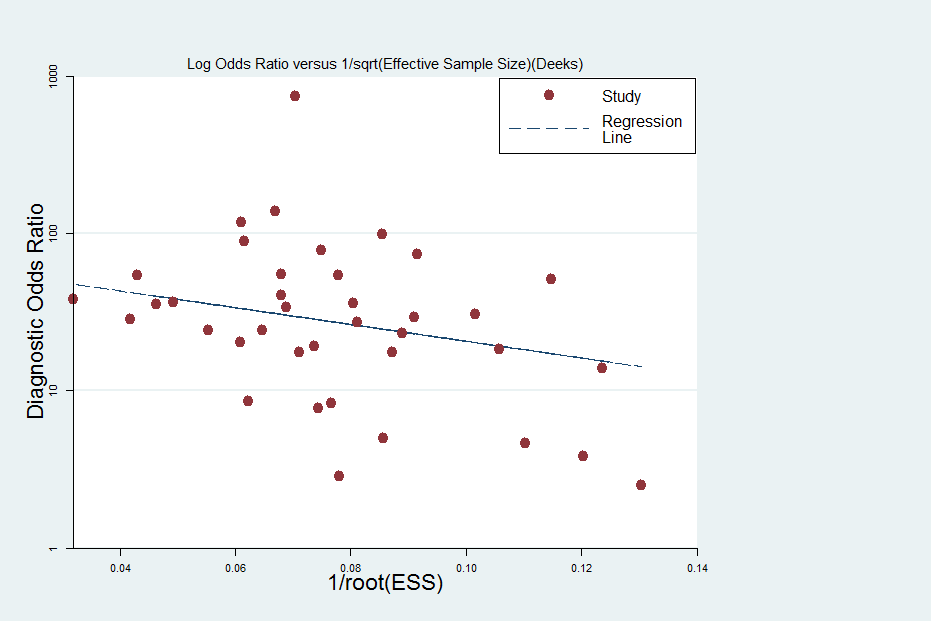

Supplement: S1 File — (ZIP) [file pone.0299045.s001.zip › statistical analysis/PET╩2╛▌/graph1.tif]

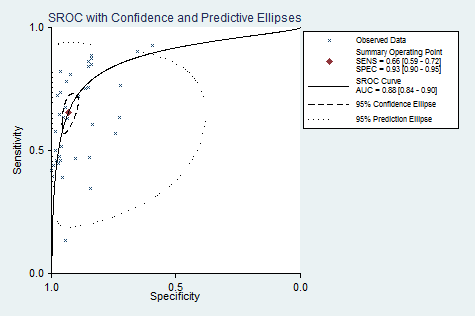

Supplement: S1 File — (ZIP) [file pone.0299045.s001.zip › statistical analysis/PET╩2╛▌/graph2.tif]

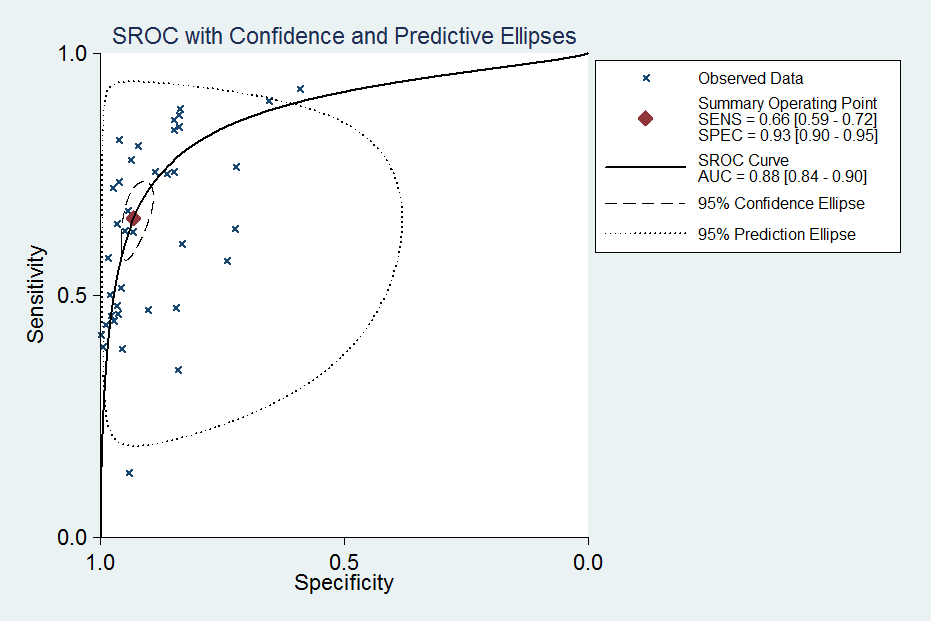

Supplement: S1 File — (ZIP) [file pone.0299045.s001.zip › statistical analysis/PET╩2╛▌/Graph-SROC.tif]

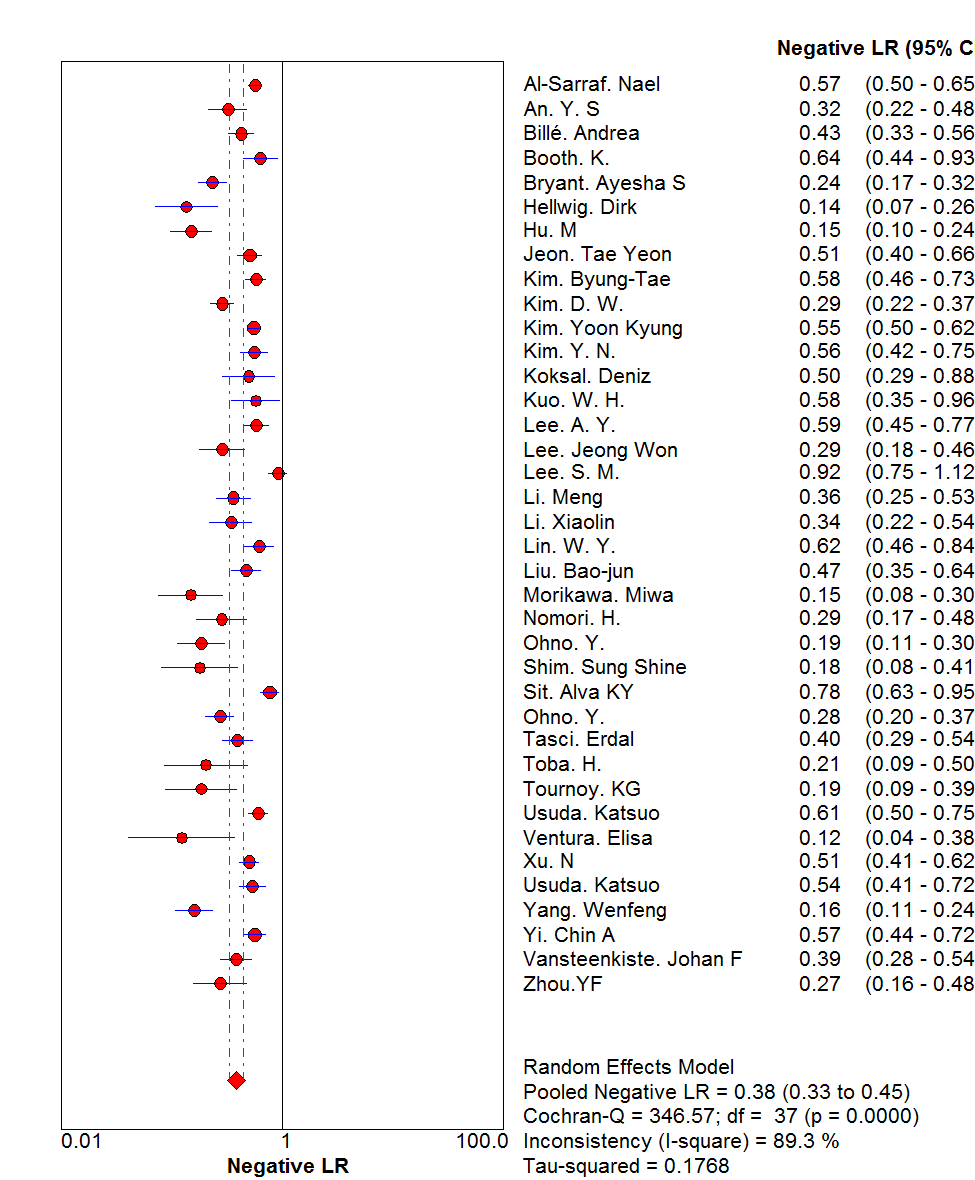

Supplement: S1 File — (ZIP) [file pone.0299045.s001.zip › statistical analysis/PET╩2╛▌/NLR.bmp]

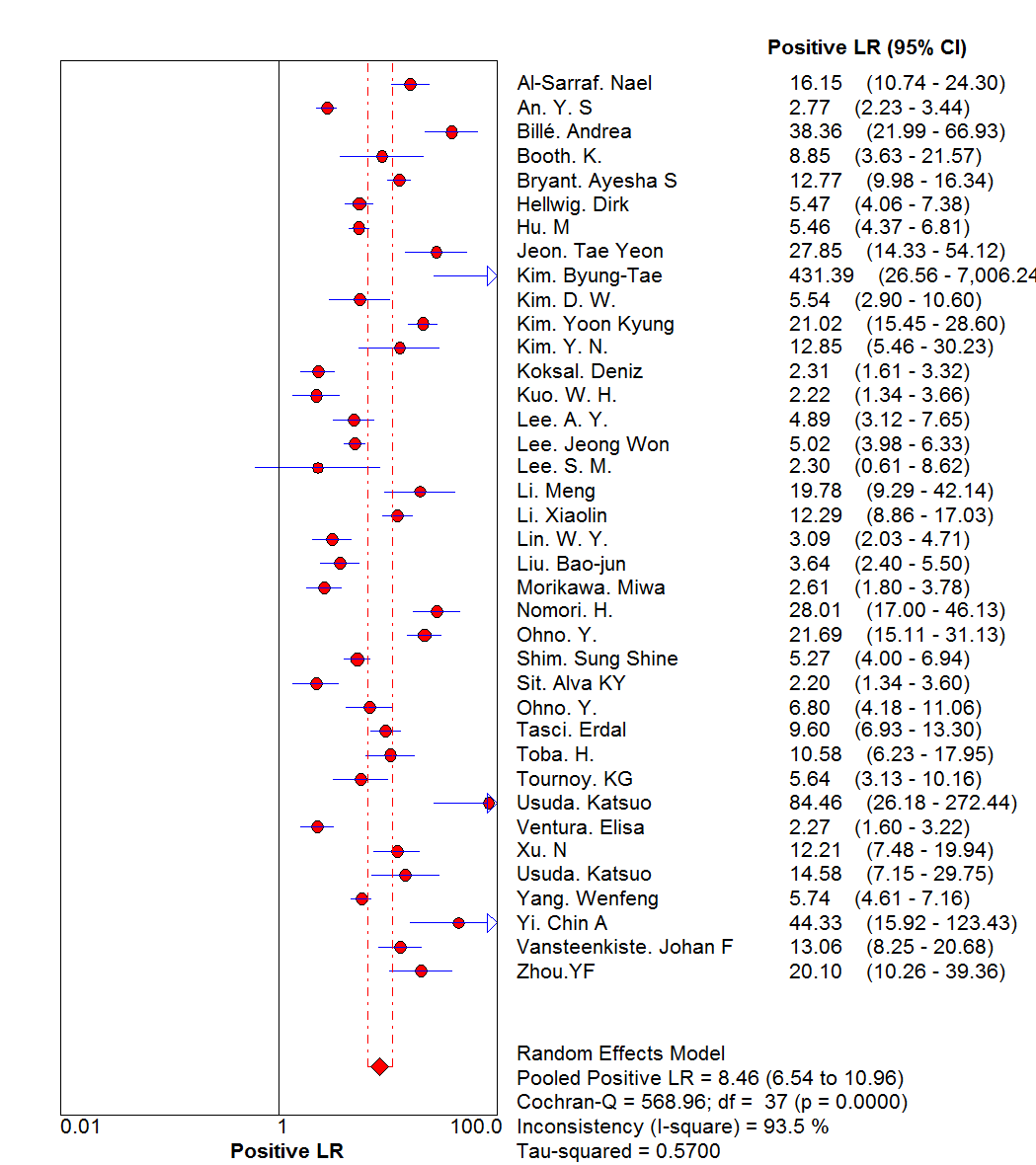

Supplement: S1 File — (ZIP) [file pone.0299045.s001.zip › statistical analysis/PET╩2╛▌/PLR.bmp]

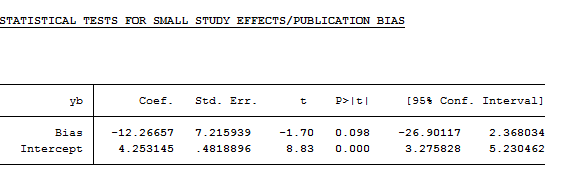

Supplement: S1 File — (ZIP) [file pone.0299045.s001.zip › statistical analysis/PET╩2╛▌/QQ═╝╞1⁄420151220145909.png]

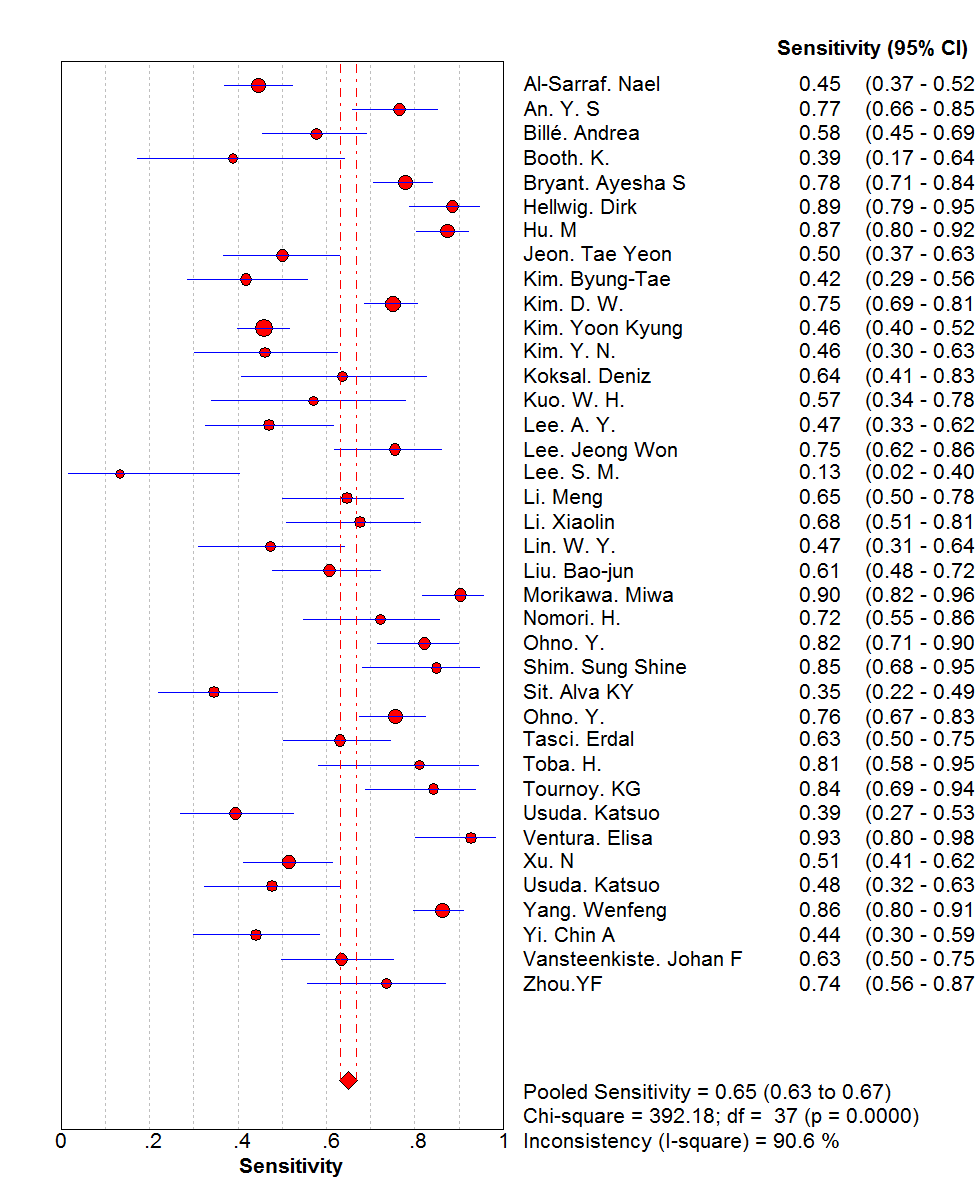

Supplement: S1 File — (ZIP) [file pone.0299045.s001.zip › statistical analysis/PET╩2╛▌/SEN.bmp]

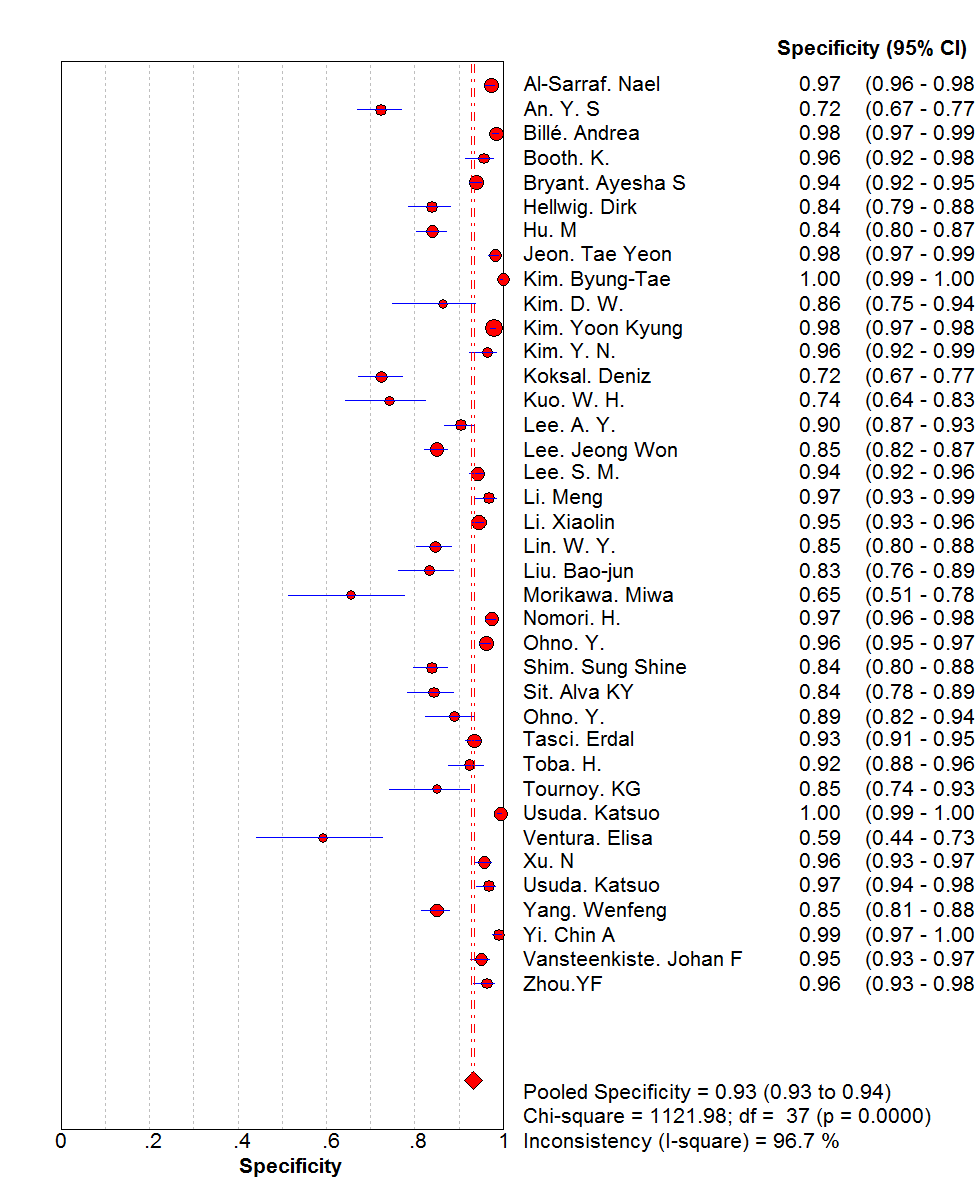

Supplement: S1 File — (ZIP) [file pone.0299045.s001.zip › statistical analysis/PET╩2╛▌/SPE.bmp]

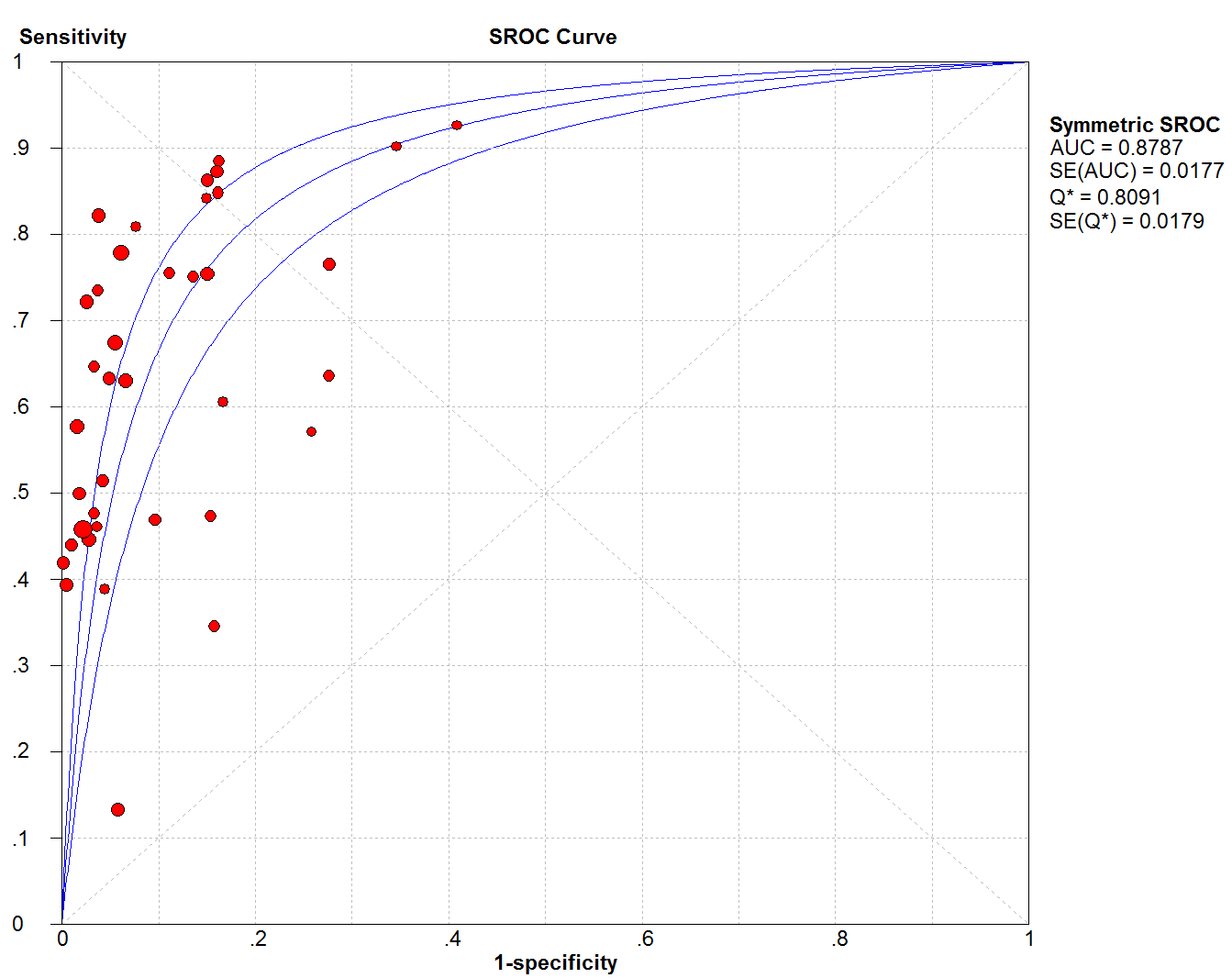

Supplement: S1 File — (ZIP) [file pone.0299045.s001.zip › statistical analysis/PET╩2╛▌/SROC.bmp]

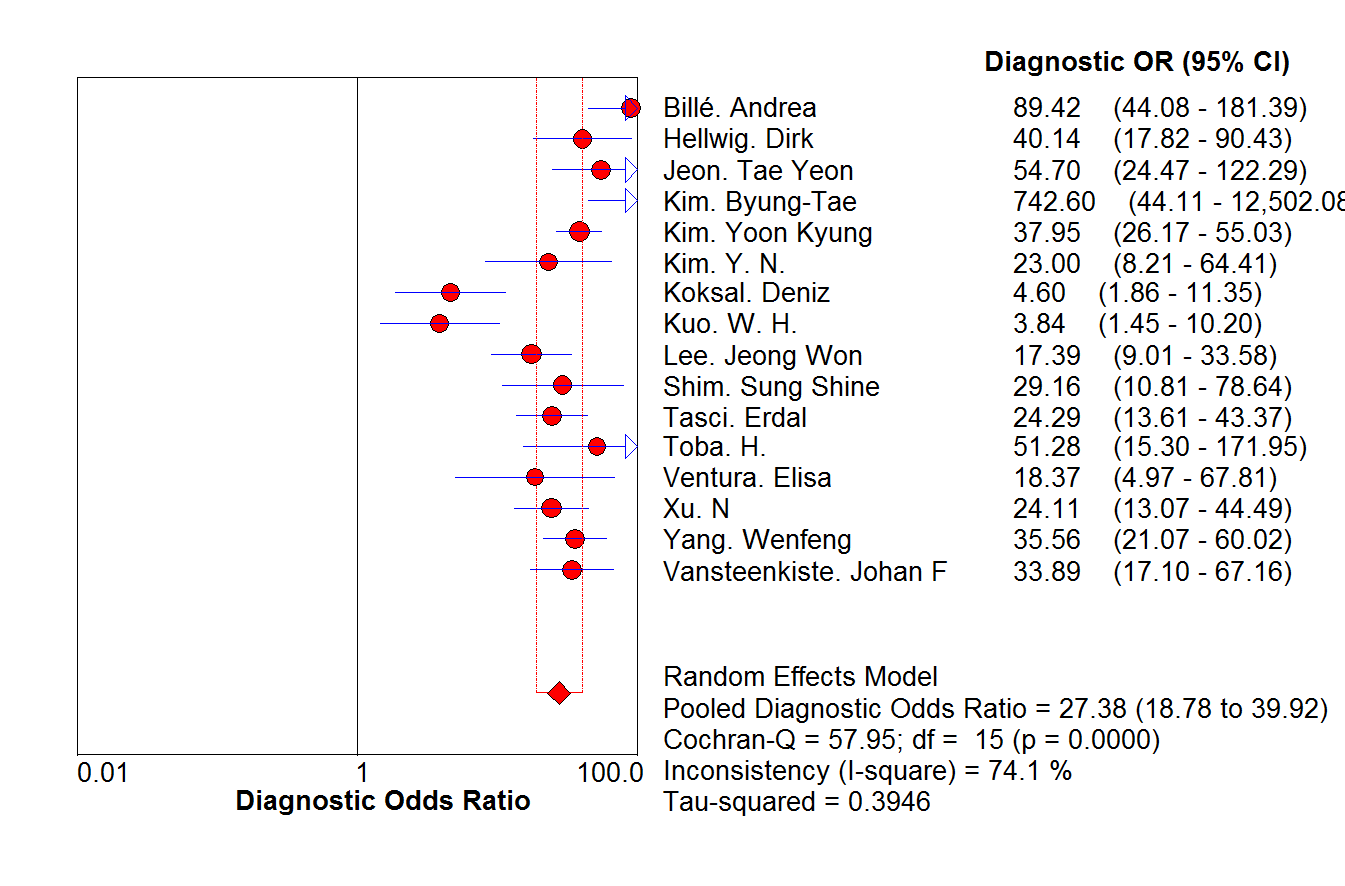

Supplement: S1 File — (ZIP) [file pone.0299045.s001.zip › statistical analysis/PET╩2╛▌/╤╟╫Θ╖╓╬÷/analysis method QL/DOR.bmp]

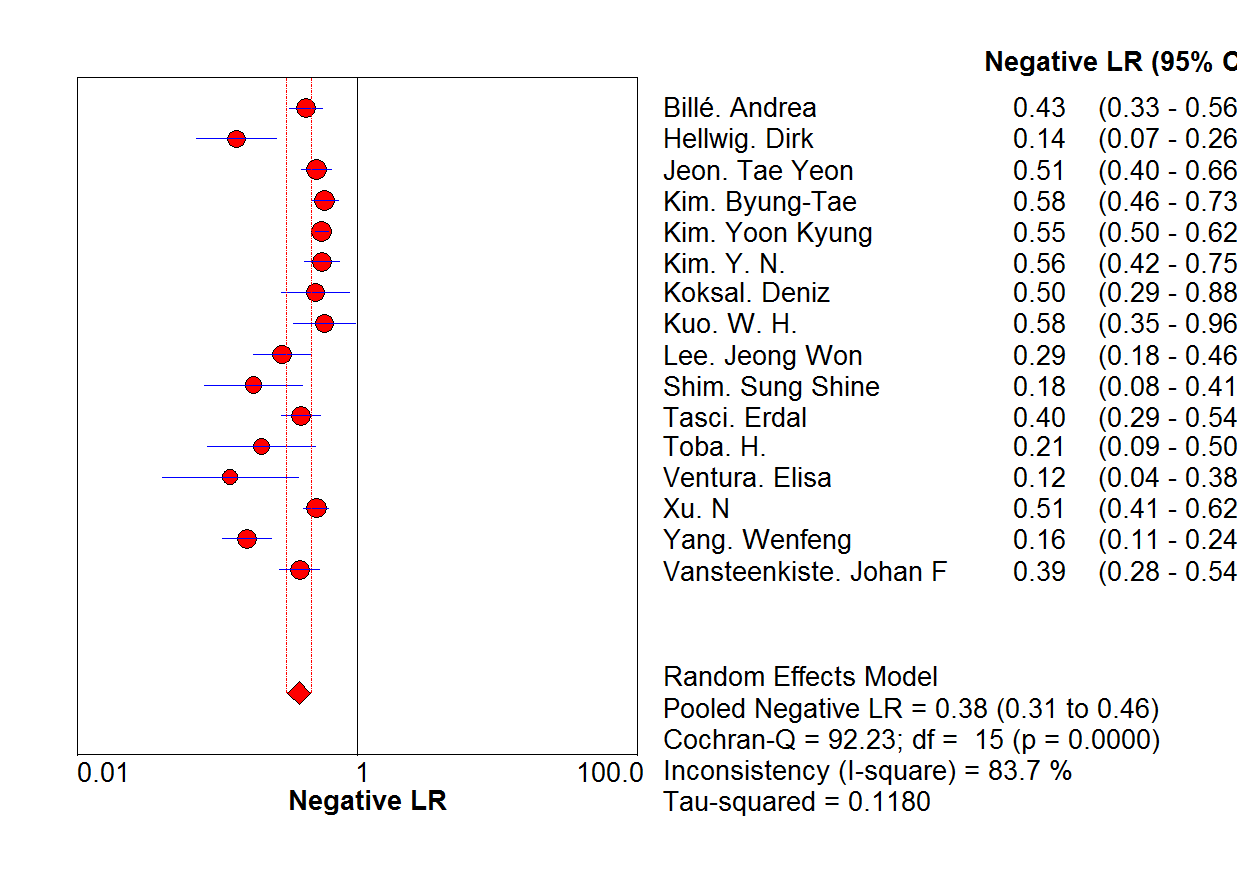

Supplement: S1 File — (ZIP) [file pone.0299045.s001.zip › statistical analysis/PET╩2╛▌/╤╟╫Θ╖╓╬÷/analysis method QL/NLR.bmp]

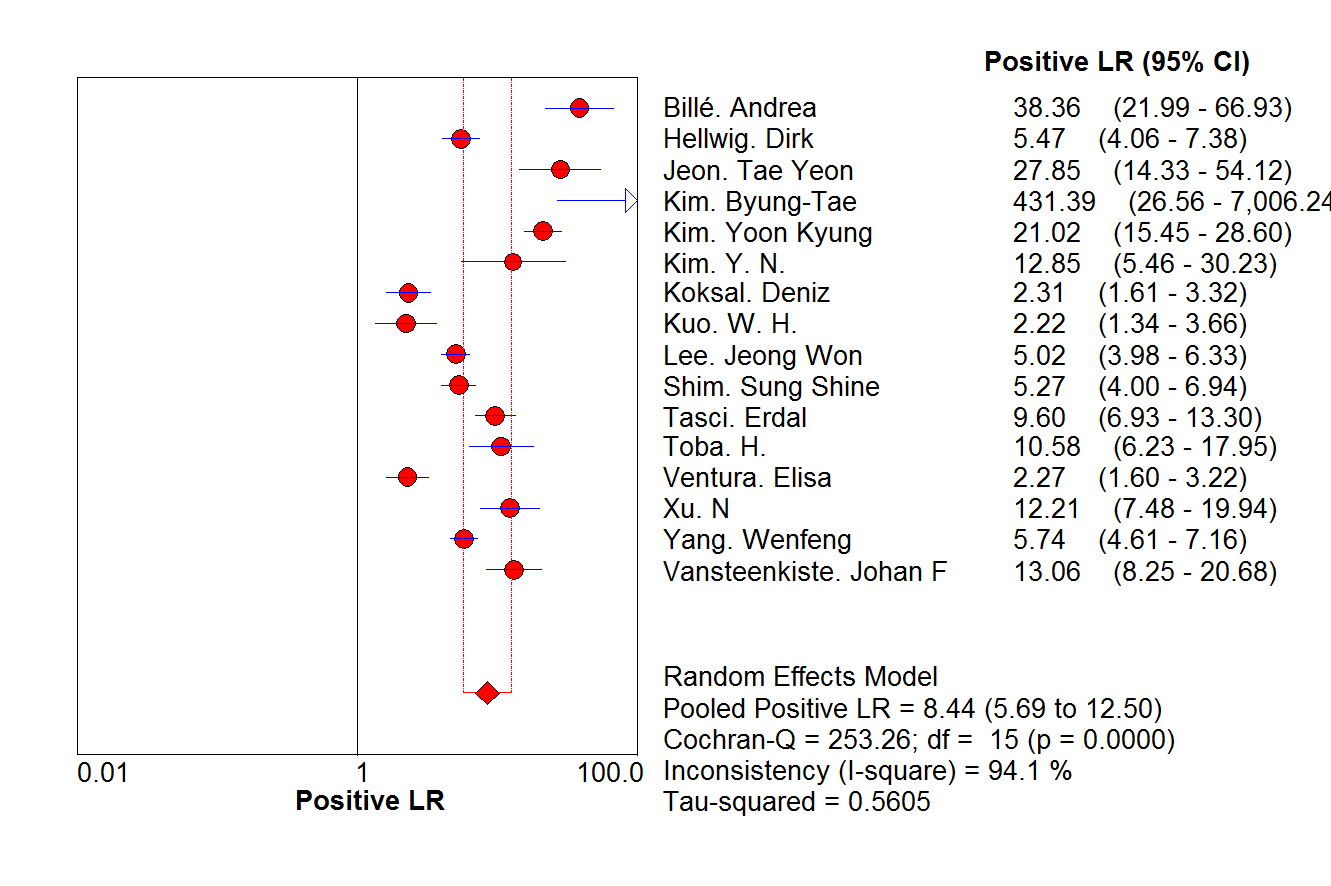

Supplement: S1 File — (ZIP) [file pone.0299045.s001.zip › statistical analysis/PET╩2╛▌/╤╟╫Θ╖╓╬÷/analysis method QL/PLR.bmp]

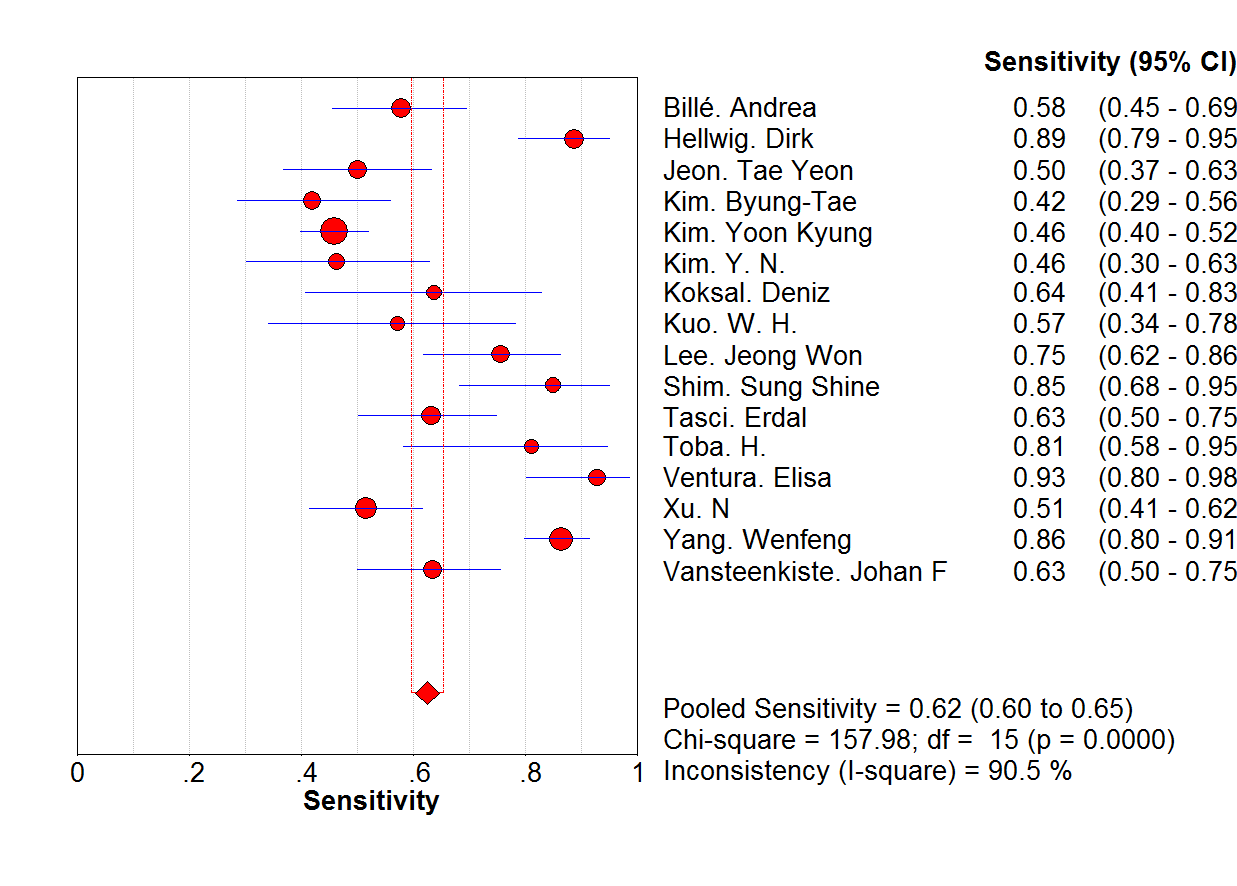

Supplement: S1 File — (ZIP) [file pone.0299045.s001.zip › statistical analysis/PET╩2╛▌/╤╟╫Θ╖╓╬÷/analysis method QL/SEN.bmp]

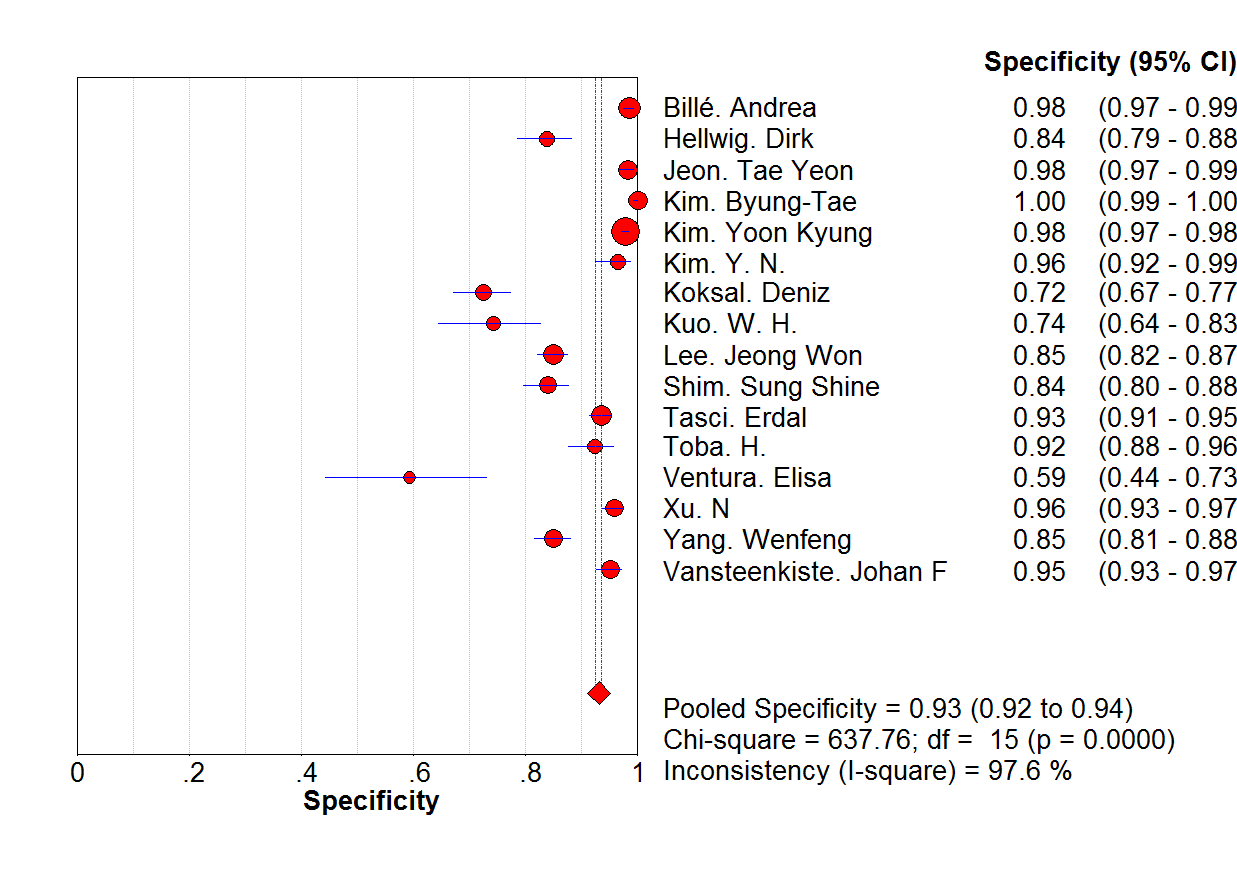

Supplement: S1 File — (ZIP) [file pone.0299045.s001.zip › statistical analysis/PET╩2╛▌/╤╟╫Θ╖╓╬÷/analysis method QL/SPE.bmp]

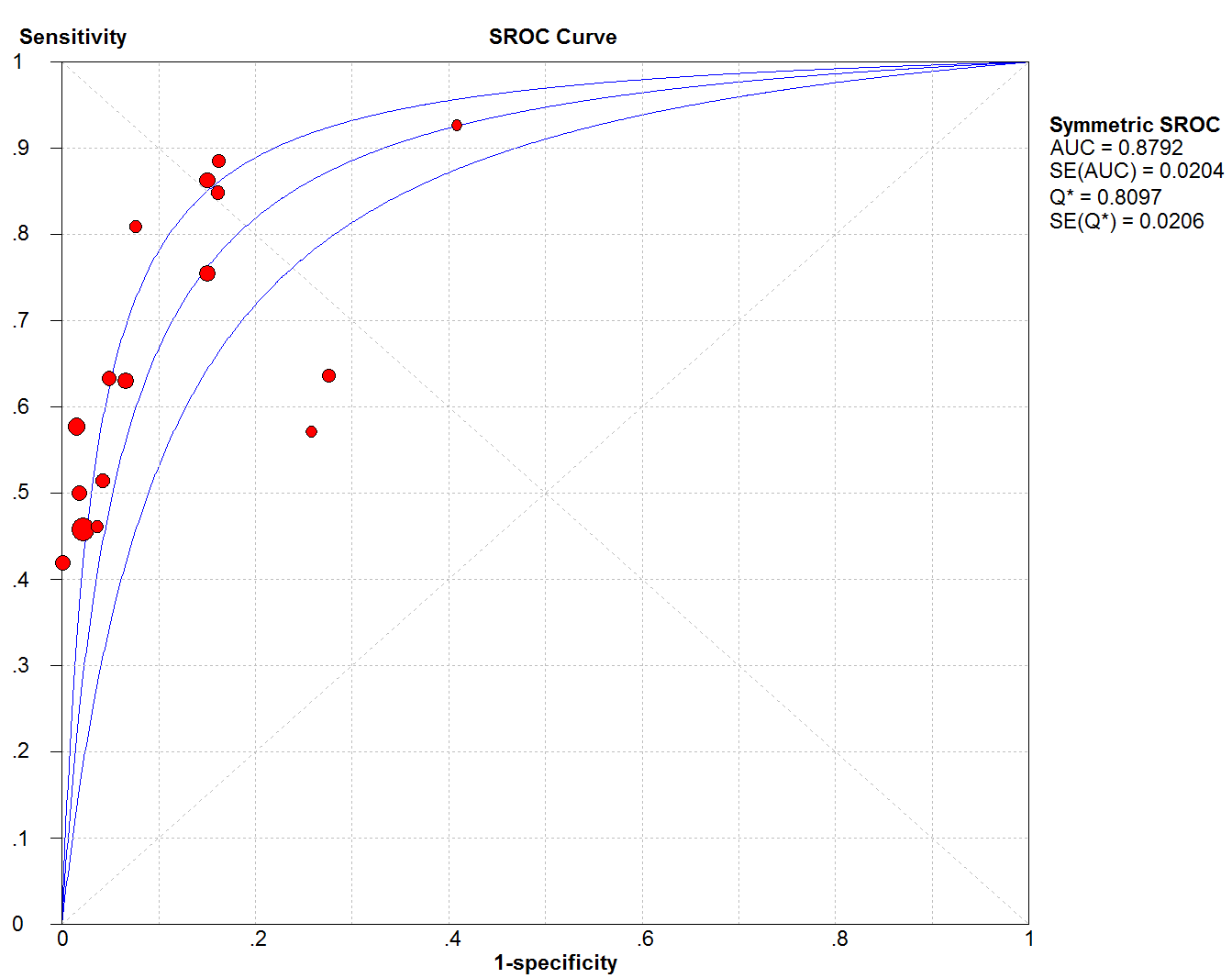

Supplement: S1 File — (ZIP) [file pone.0299045.s001.zip › statistical analysis/PET╩2╛▌/╤╟╫Θ╖╓╬÷/analysis method QL/SROC.bmp]

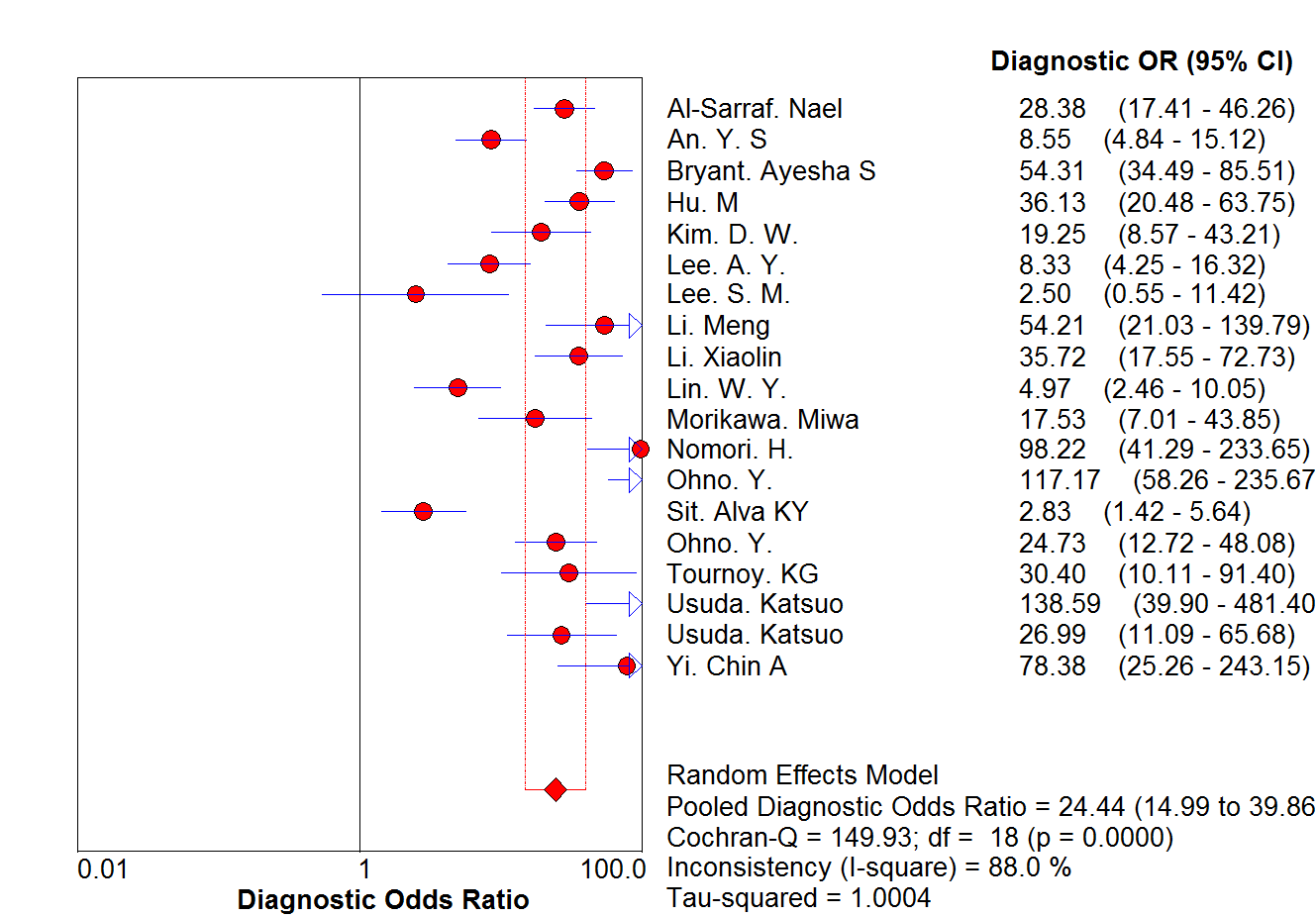

Supplement: S1 File — (ZIP) [file pone.0299045.s001.zip › statistical analysis/PET╩2╛▌/╤╟╫Θ╖╓╬÷/analysis method QN/DOR.bmp]

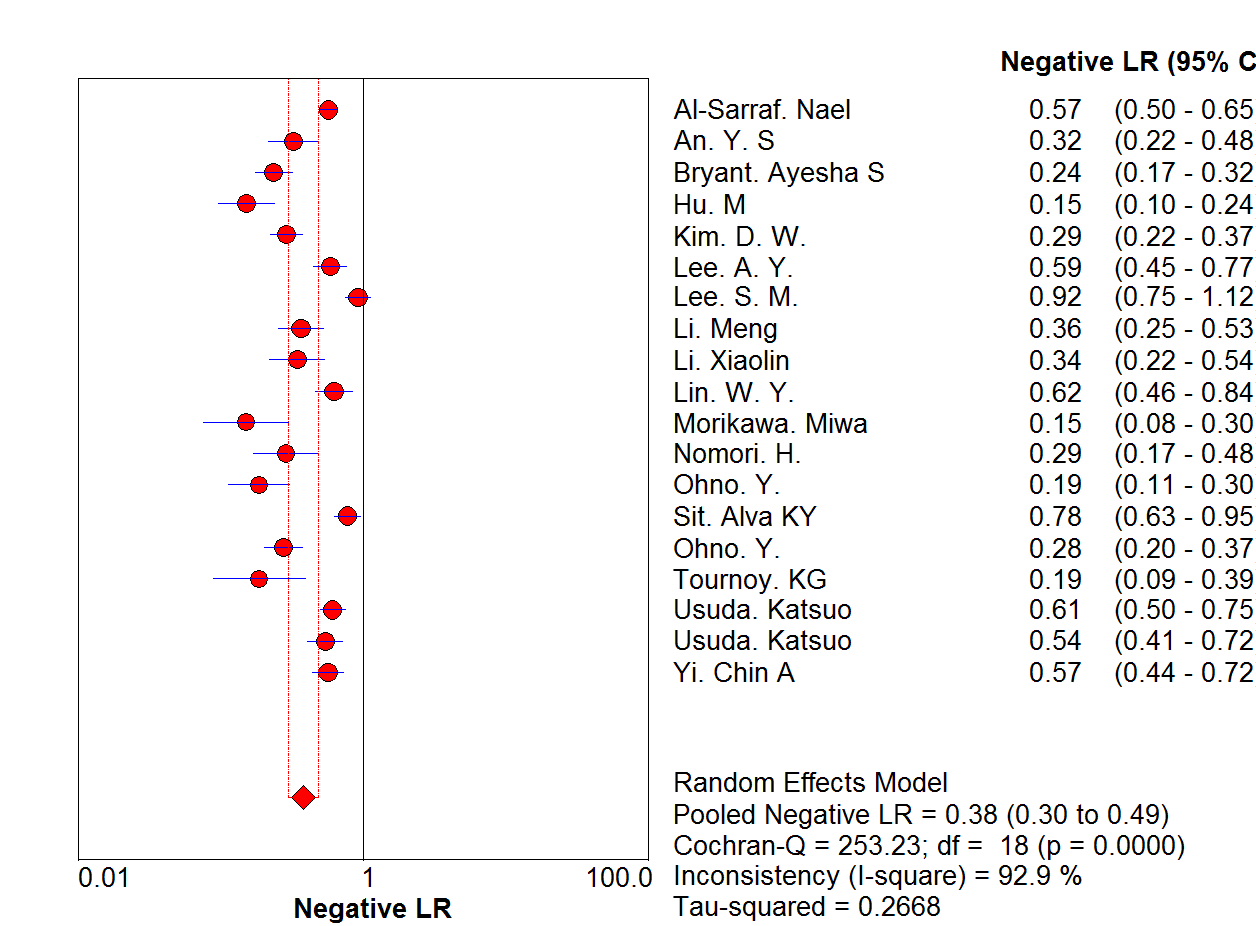

Supplement: S1 File — (ZIP) [file pone.0299045.s001.zip › statistical analysis/PET╩2╛▌/╤╟╫Θ╖╓╬÷/analysis method QN/NLR.bmp]

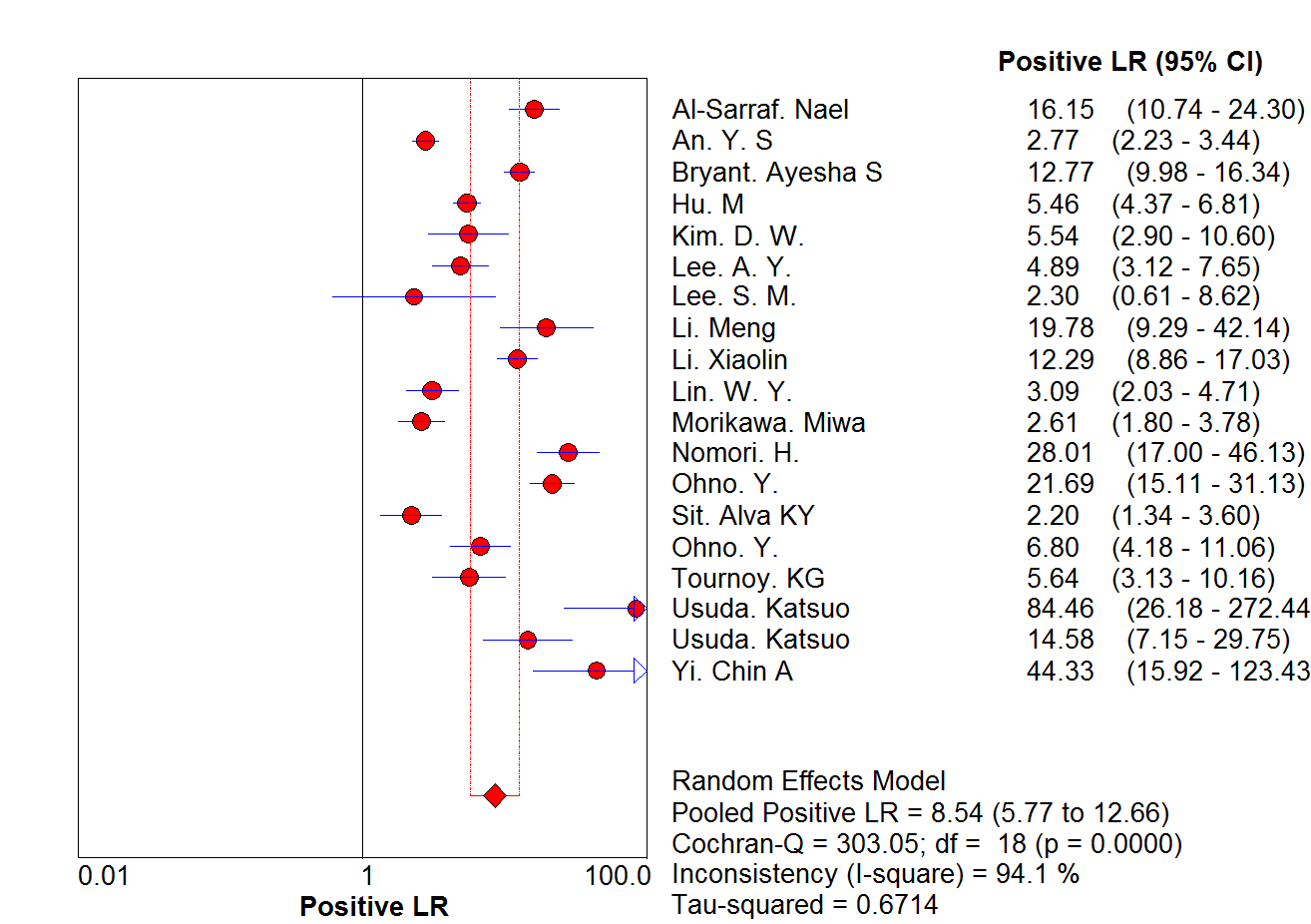

Supplement: S1 File — (ZIP) [file pone.0299045.s001.zip › statistical analysis/PET╩2╛▌/╤╟╫Θ╖╓╬÷/analysis method QN/PLR.bmp]

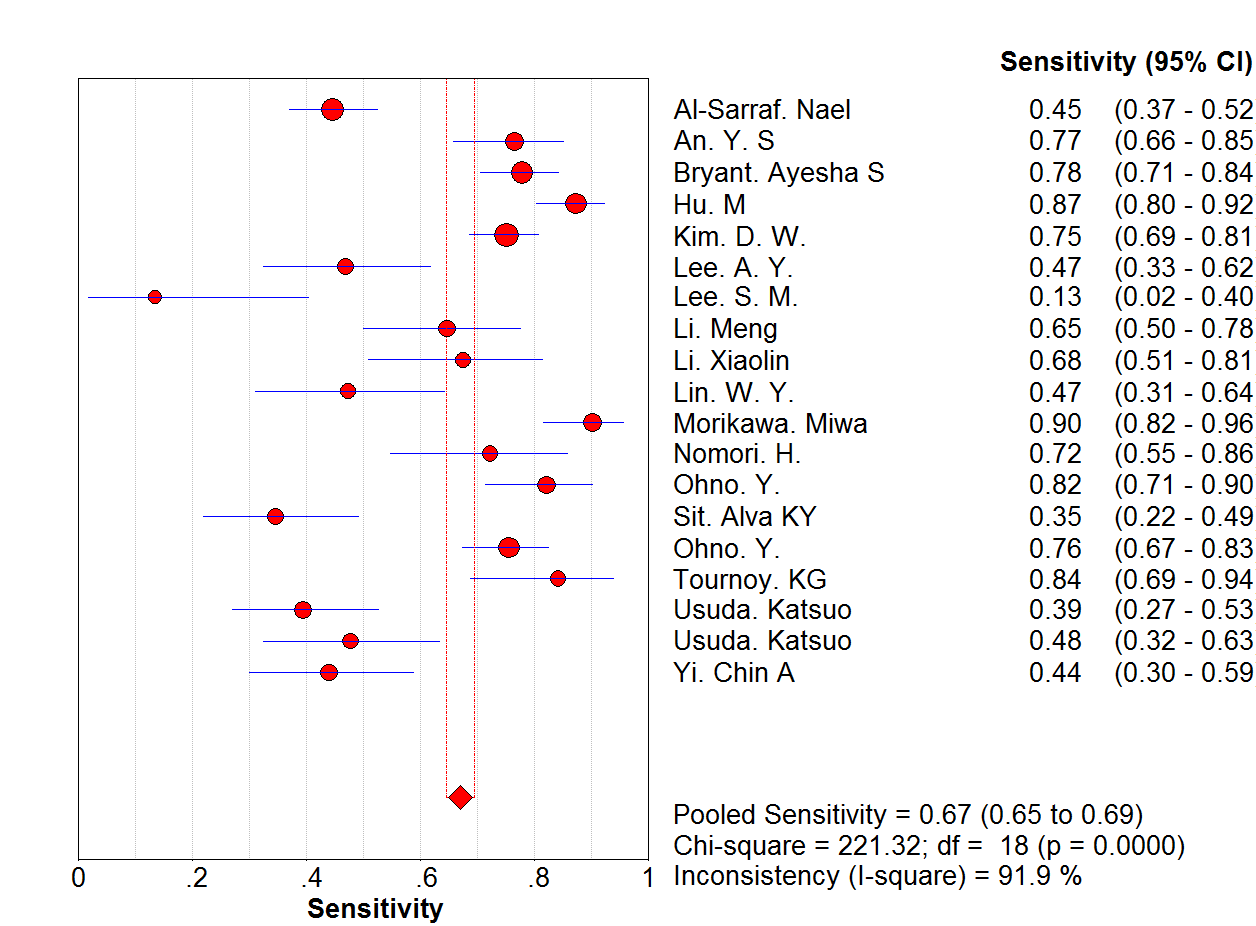

Supplement: S1 File — (ZIP) [file pone.0299045.s001.zip › statistical analysis/PET╩2╛▌/╤╟╫Θ╖╓╬÷/analysis method QN/SEN.bmp]

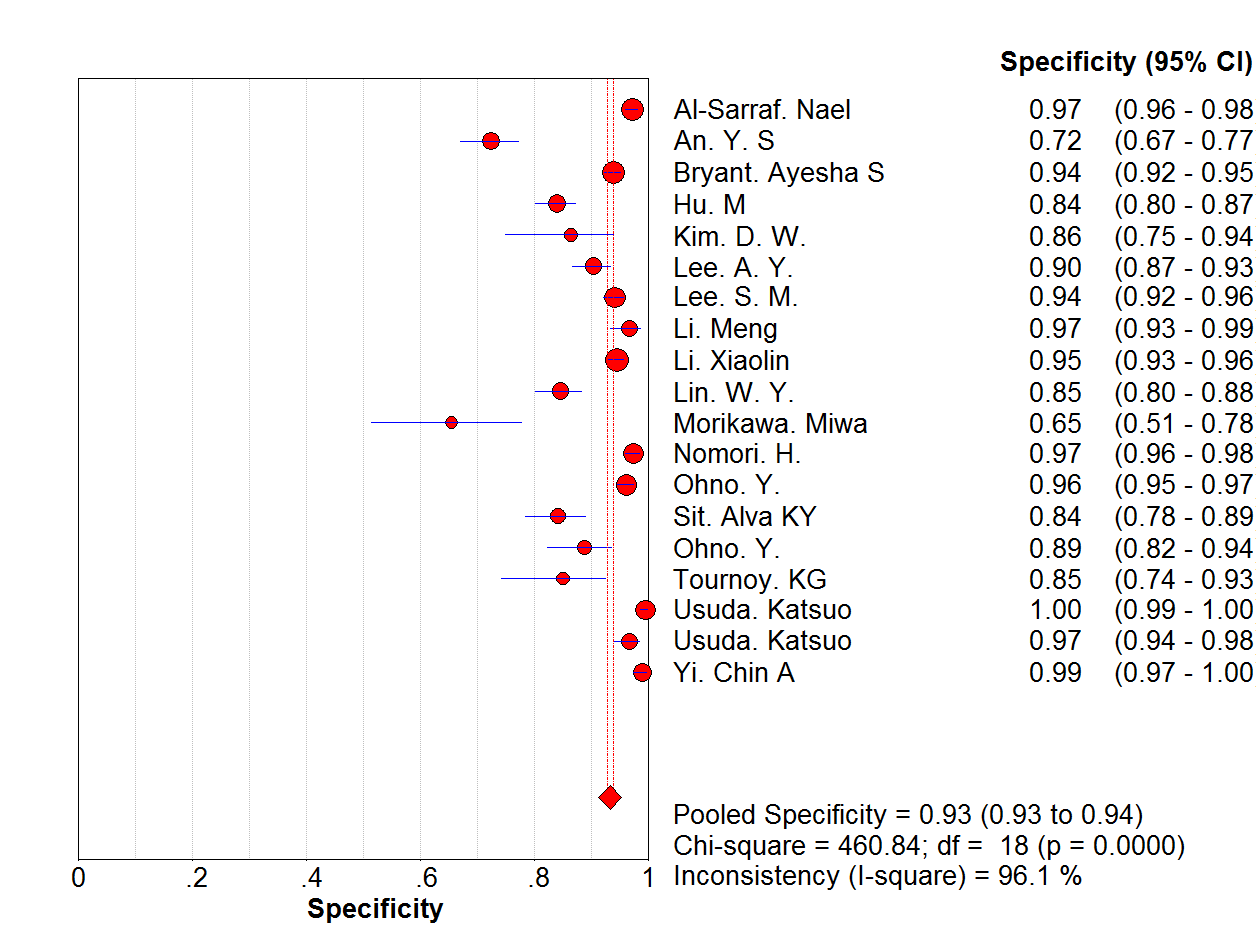

Supplement: S1 File — (ZIP) [file pone.0299045.s001.zip › statistical analysis/PET╩2╛▌/╤╟╫Θ╖╓╬÷/analysis method QN/SPE.bmp]

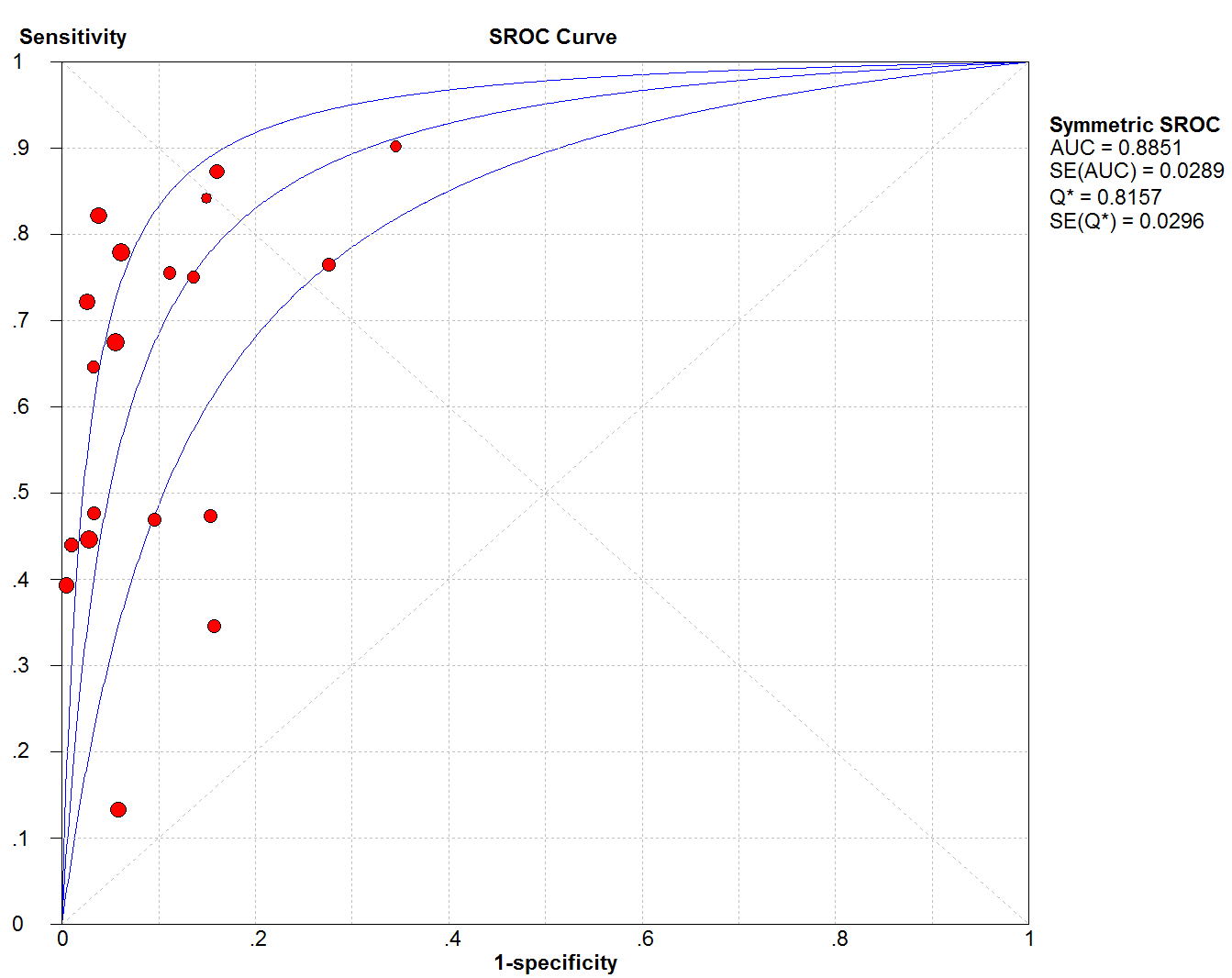

Supplement: S1 File — (ZIP) [file pone.0299045.s001.zip › statistical analysis/PET╩2╛▌/╤╟╫Θ╖╓╬÷/analysis method QN/SROC.bmp]

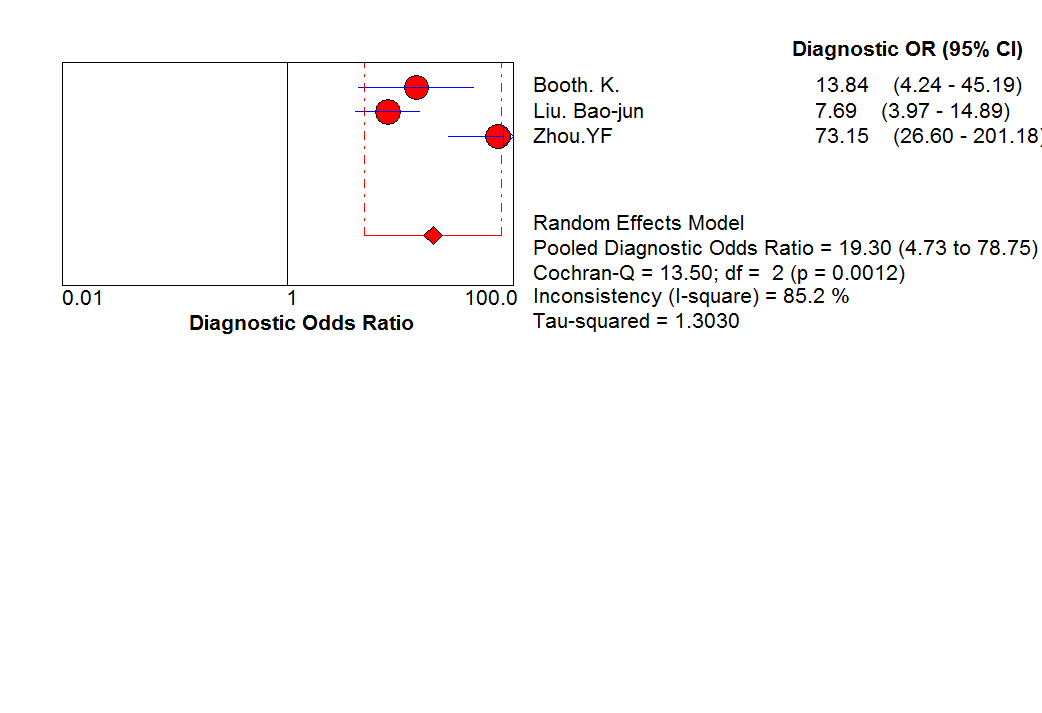

Supplement: S1 File — (ZIP) [file pone.0299045.s001.zip › statistical analysis/PET╩2╛▌/╤╟╫Θ╖╓╬÷/analysis method QN+QL/DOR.bmp]

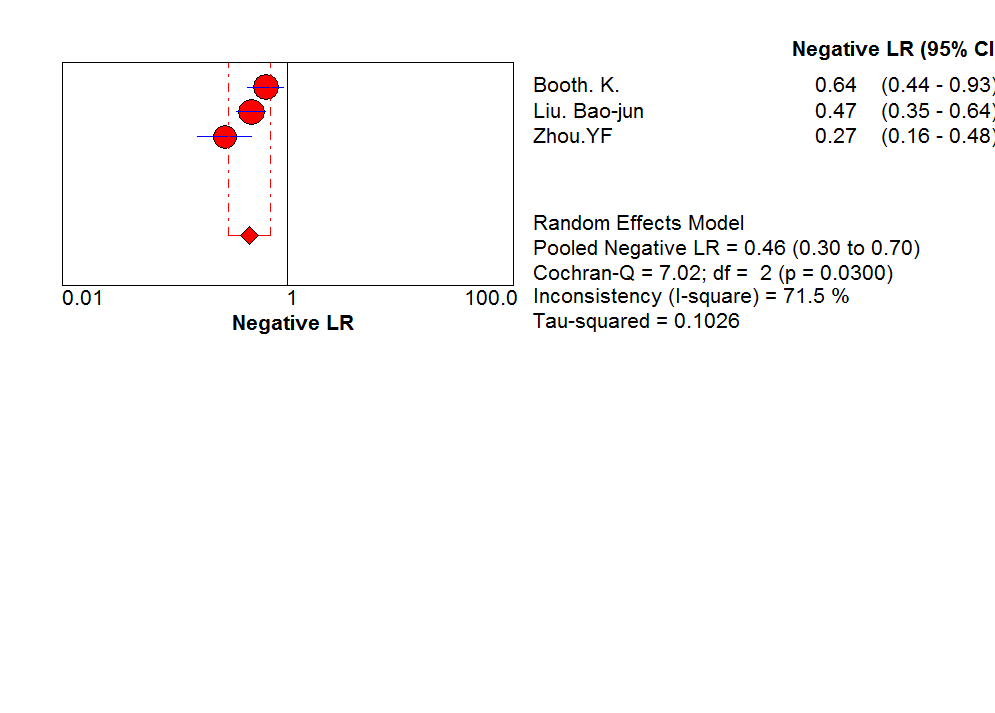

Supplement: S1 File — (ZIP) [file pone.0299045.s001.zip › statistical analysis/PET╩2╛▌/╤╟╫Θ╖╓╬÷/analysis method QN+QL/NLR.bmp]

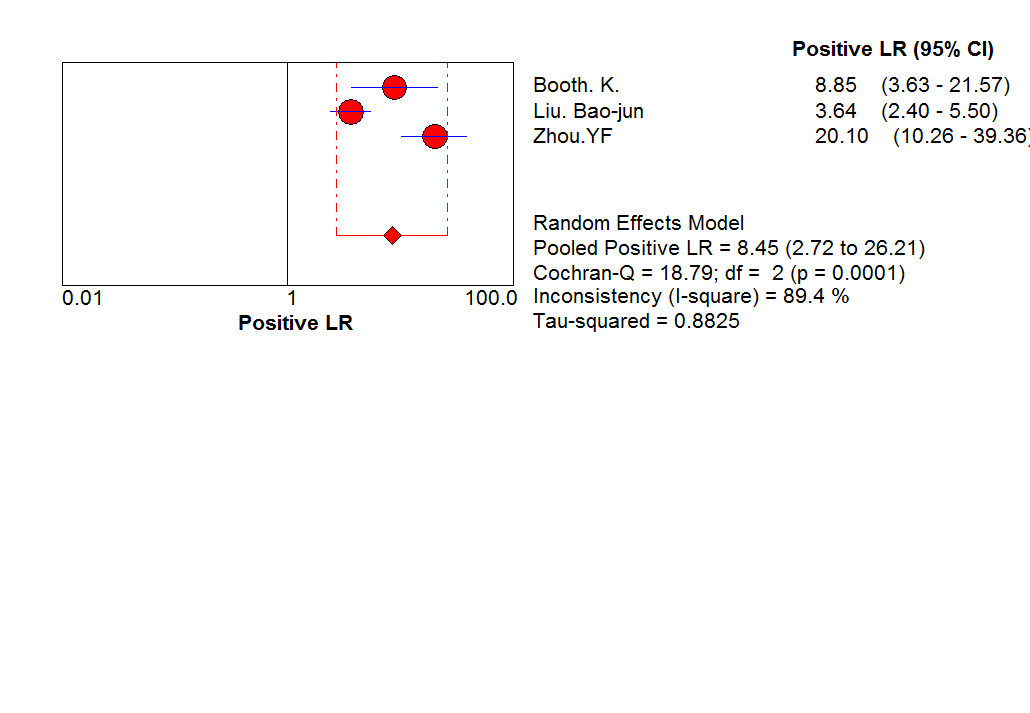

Supplement: S1 File — (ZIP) [file pone.0299045.s001.zip › statistical analysis/PET╩2╛▌/╤╟╫Θ╖╓╬÷/analysis method QN+QL/PLR.bmp]

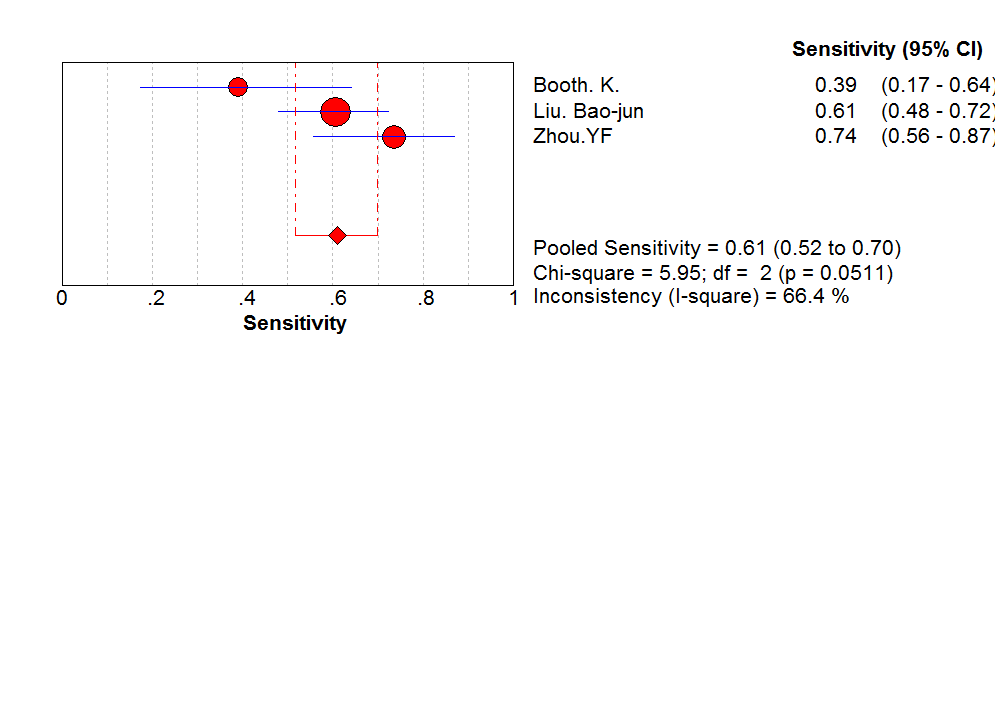

Supplement: S1 File — (ZIP) [file pone.0299045.s001.zip › statistical analysis/PET╩2╛▌/╤╟╫Θ╖╓╬÷/analysis method QN+QL/SEN.bmp]

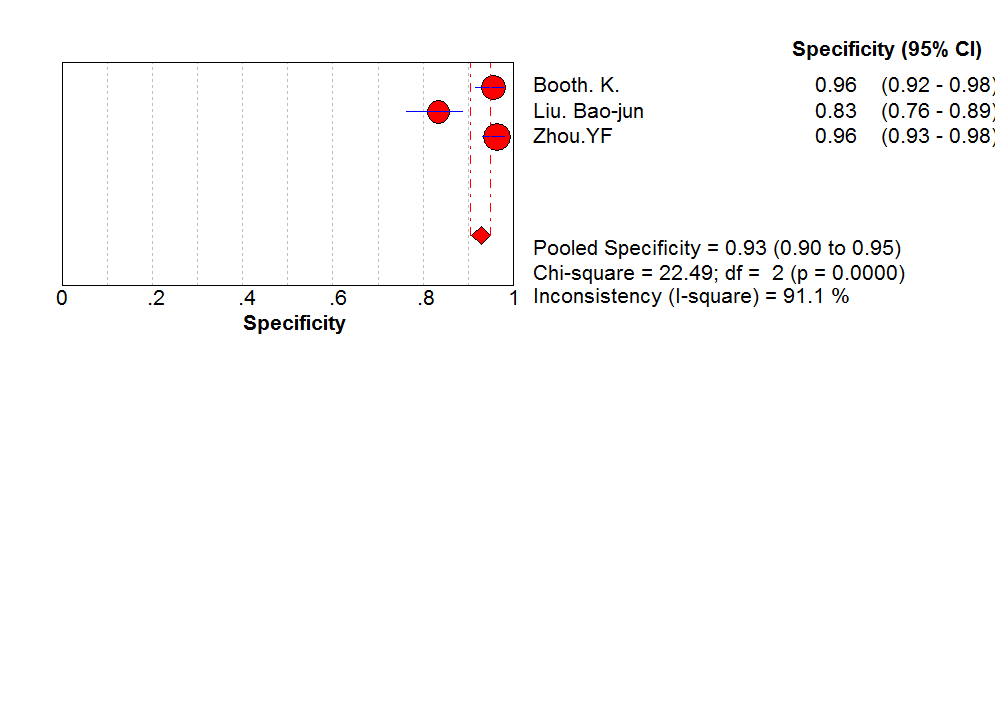

Supplement: S1 File — (ZIP) [file pone.0299045.s001.zip › statistical analysis/PET╩2╛▌/╤╟╫Θ╖╓╬÷/analysis method QN+QL/SPE.bmp]

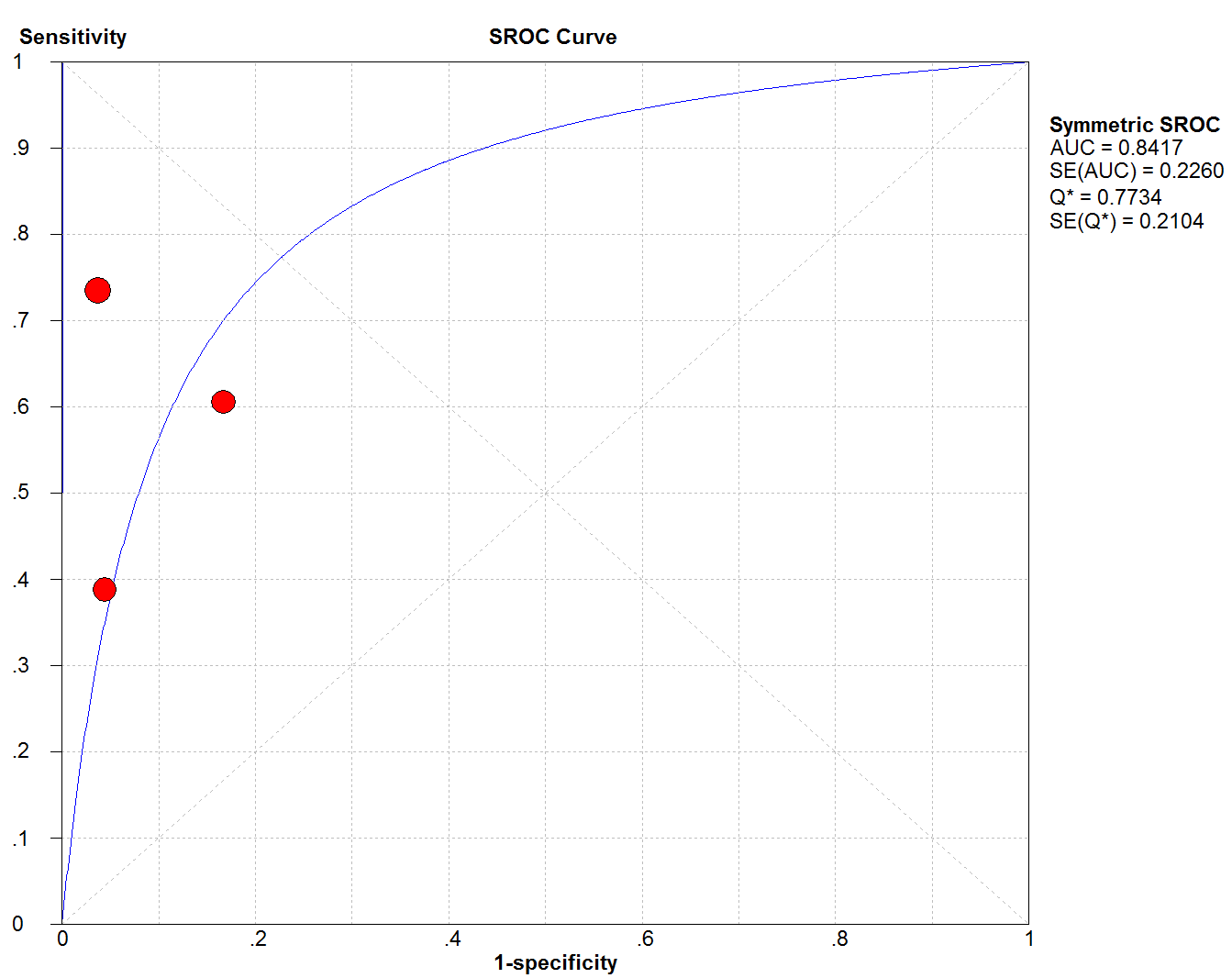

Supplement: S1 File — (ZIP) [file pone.0299045.s001.zip › statistical analysis/PET╩2╛▌/╤╟╫Θ╖╓╬÷/analysis method QN+QL/SROC.bmp]

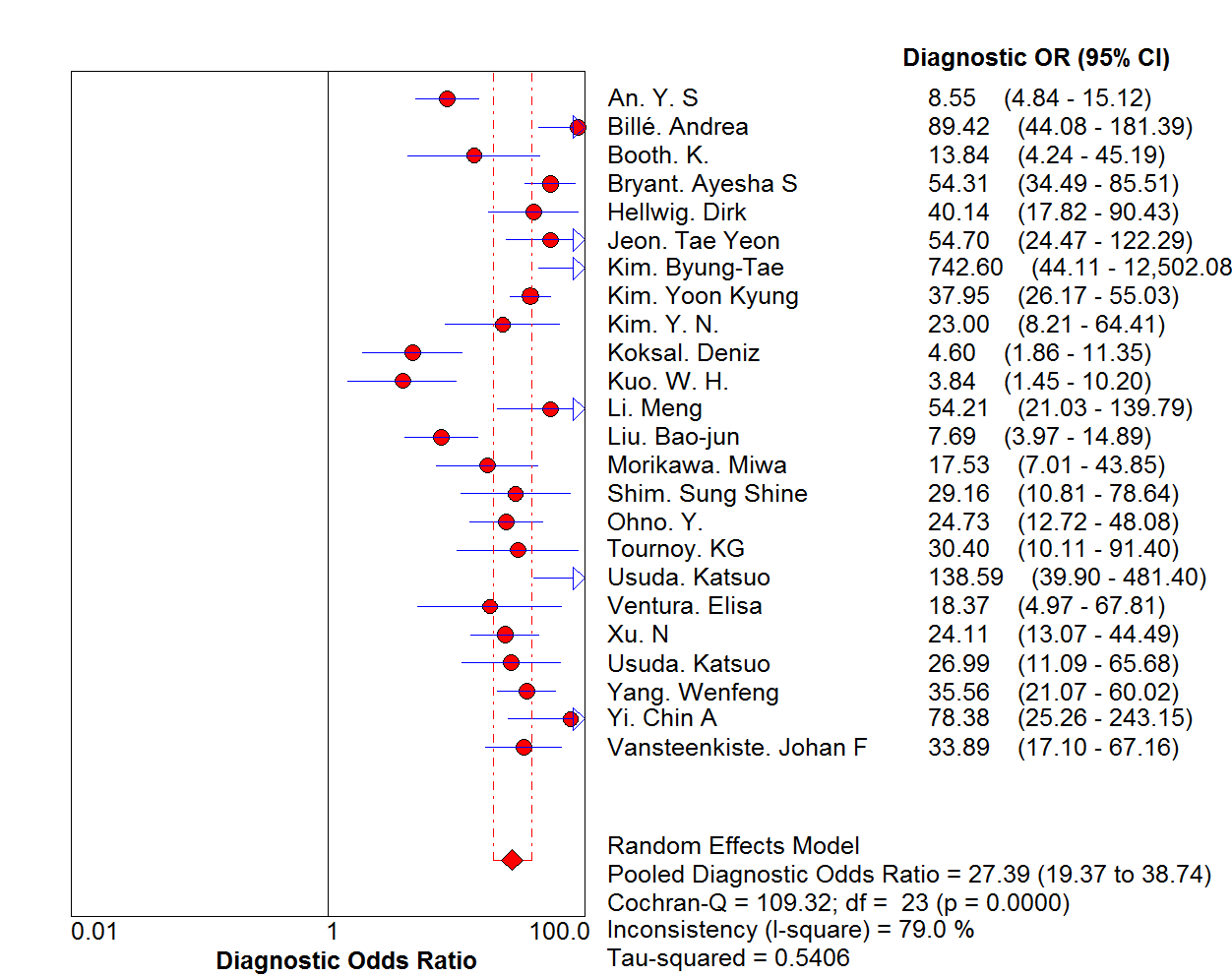

Supplement: S1 File — (ZIP) [file pone.0299045.s001.zip › statistical analysis/PET╩2╛▌/╤╟╫Θ╖╓╬÷/blind/dor.bmp]

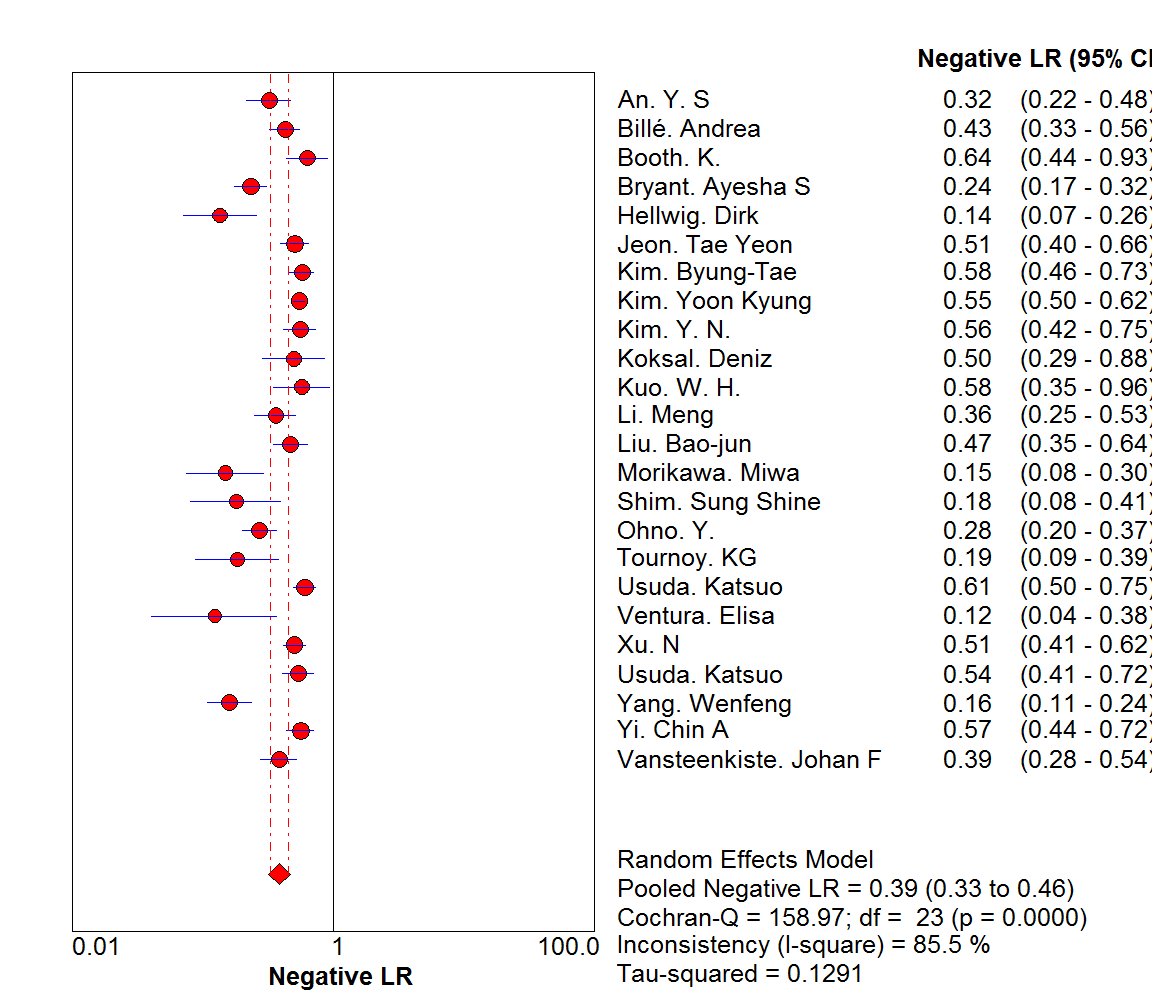

Supplement: S1 File — (ZIP) [file pone.0299045.s001.zip › statistical analysis/PET╩2╛▌/╤╟╫Θ╖╓╬÷/blind/nlr.bmp]

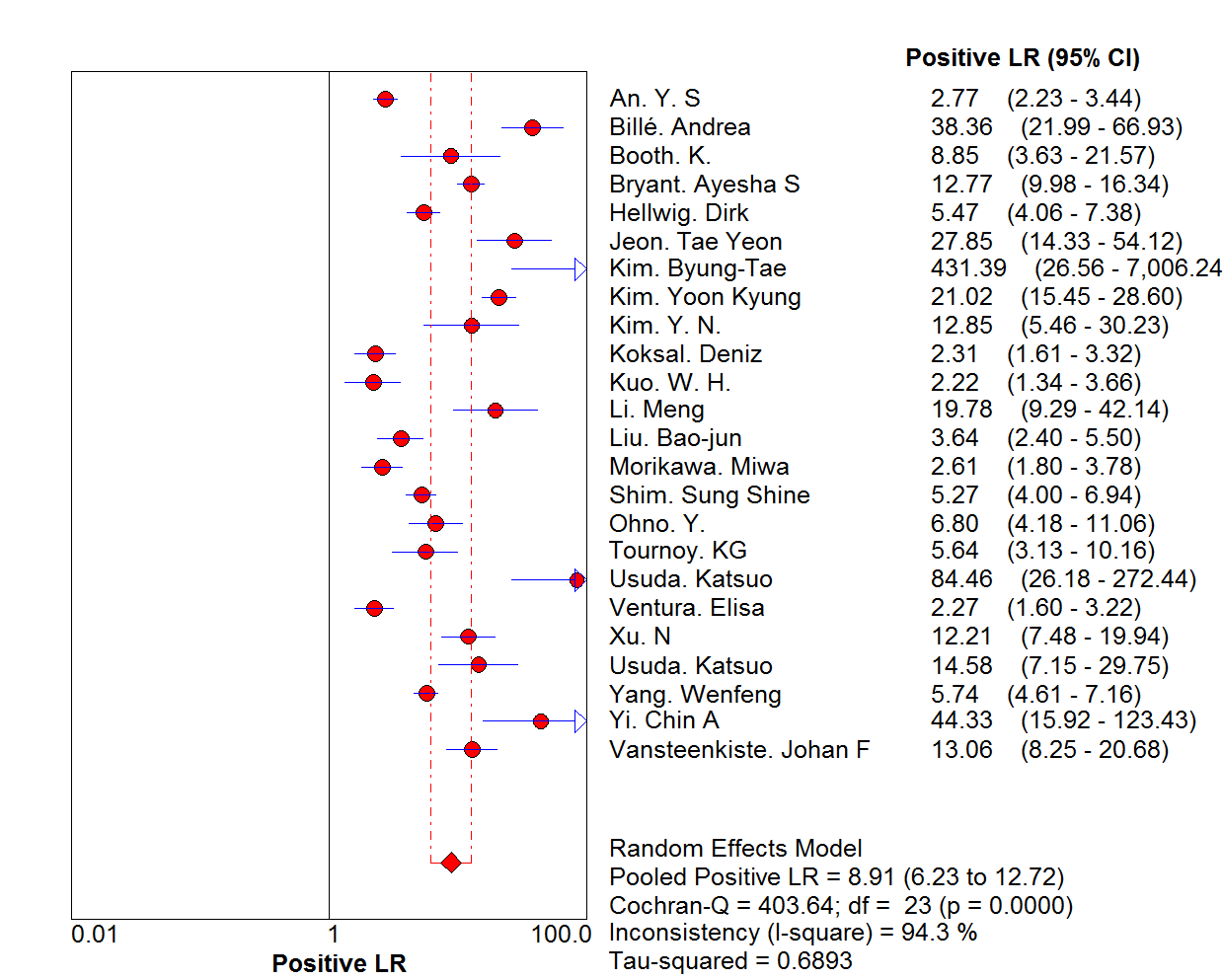

Supplement: S1 File — (ZIP) [file pone.0299045.s001.zip › statistical analysis/PET╩2╛▌/╤╟╫Θ╖╓╬÷/blind/plr.bmp]

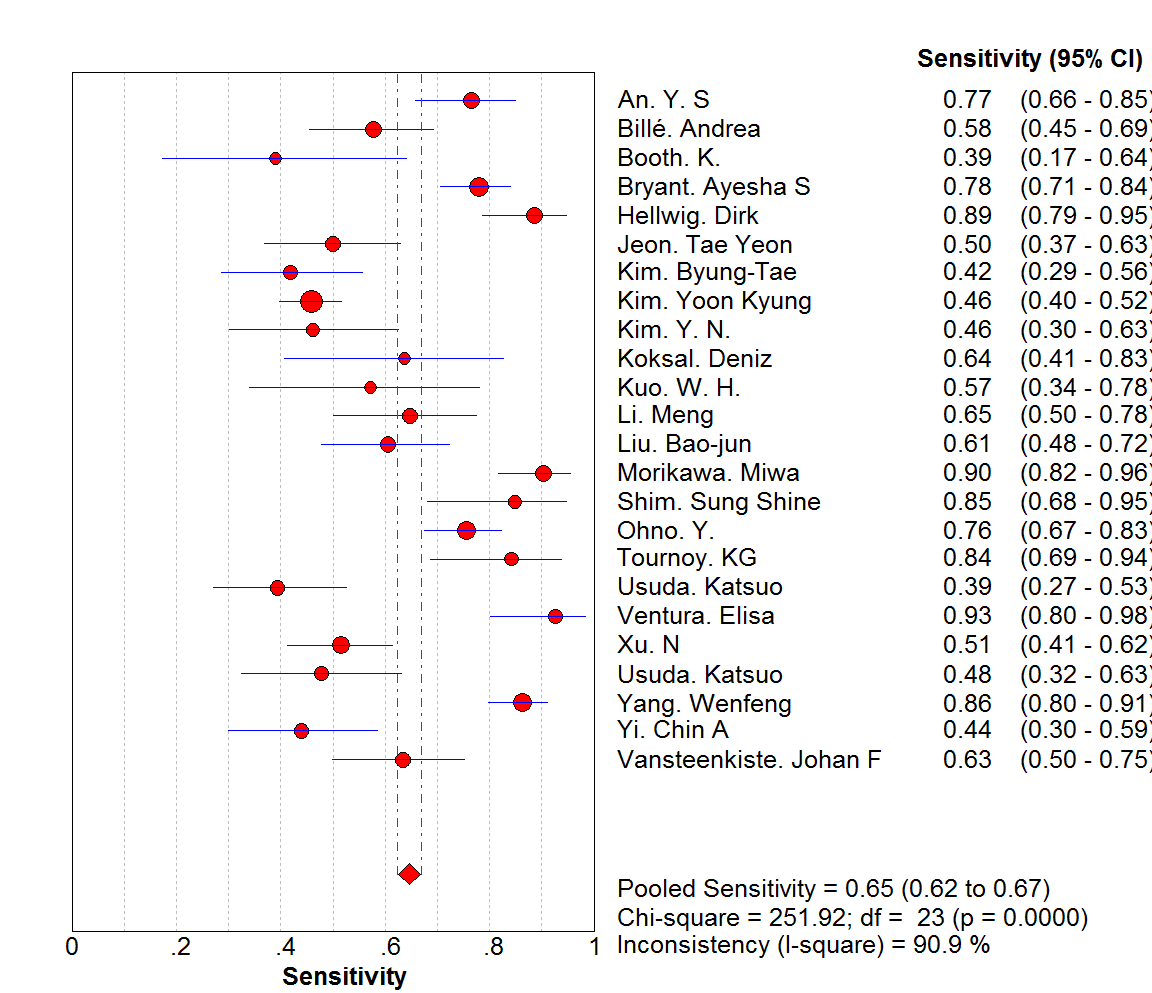

Supplement: S1 File — (ZIP) [file pone.0299045.s001.zip › statistical analysis/PET╩2╛▌/╤╟╫Θ╖╓╬÷/blind/sen.bmp]

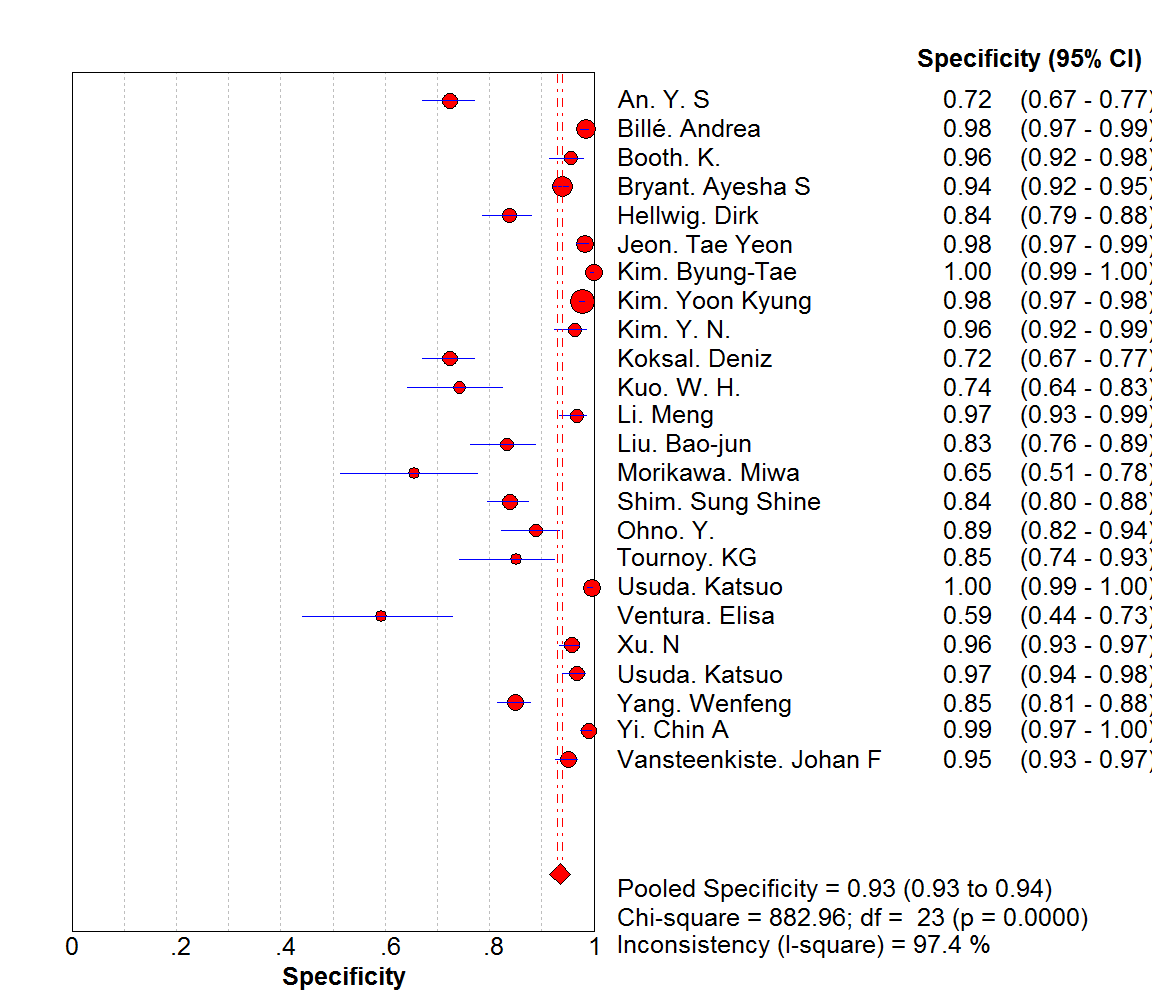

Supplement: S1 File — (ZIP) [file pone.0299045.s001.zip › statistical analysis/PET╩2╛▌/╤╟╫Θ╖╓╬÷/blind/spe.bmp]

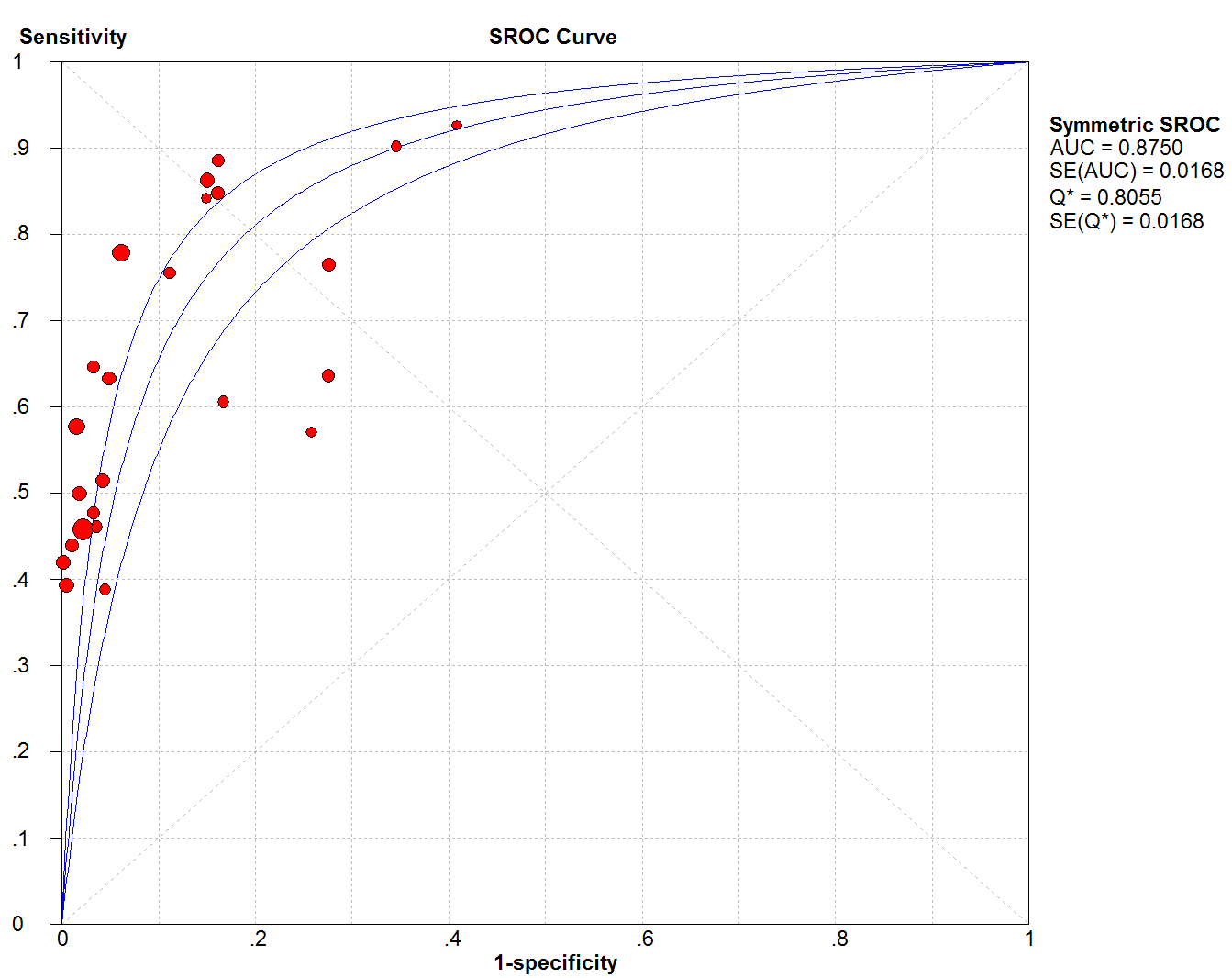

Supplement: S1 File — (ZIP) [file pone.0299045.s001.zip › statistical analysis/PET╩2╛▌/╤╟╫Θ╖╓╬÷/blind/sroc.bmp]

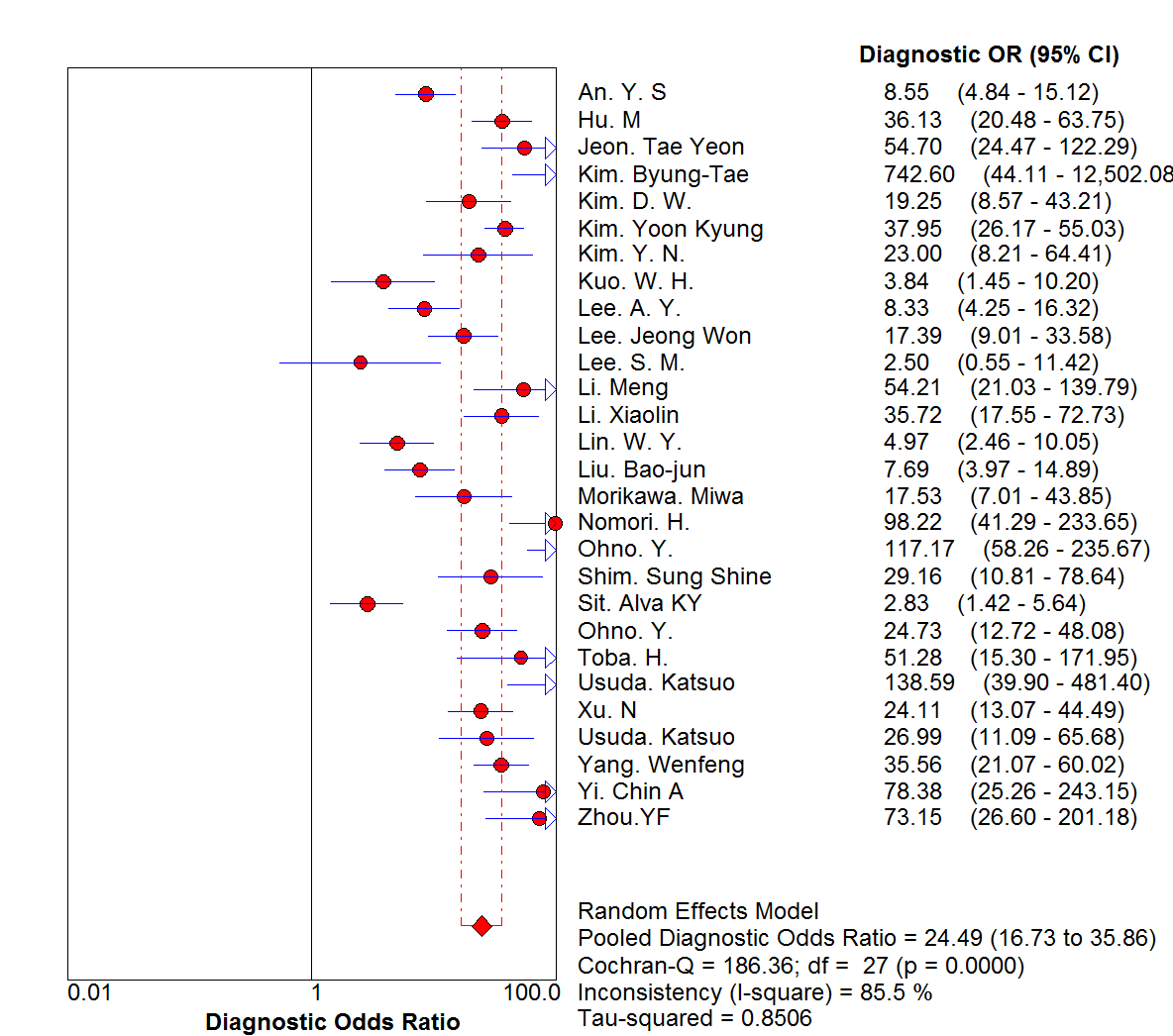

Supplement: S1 File — (ZIP) [file pone.0299045.s001.zip › statistical analysis/PET╩2╛▌/╤╟╫Θ╖╓╬÷/country Asian/dor.bmp]

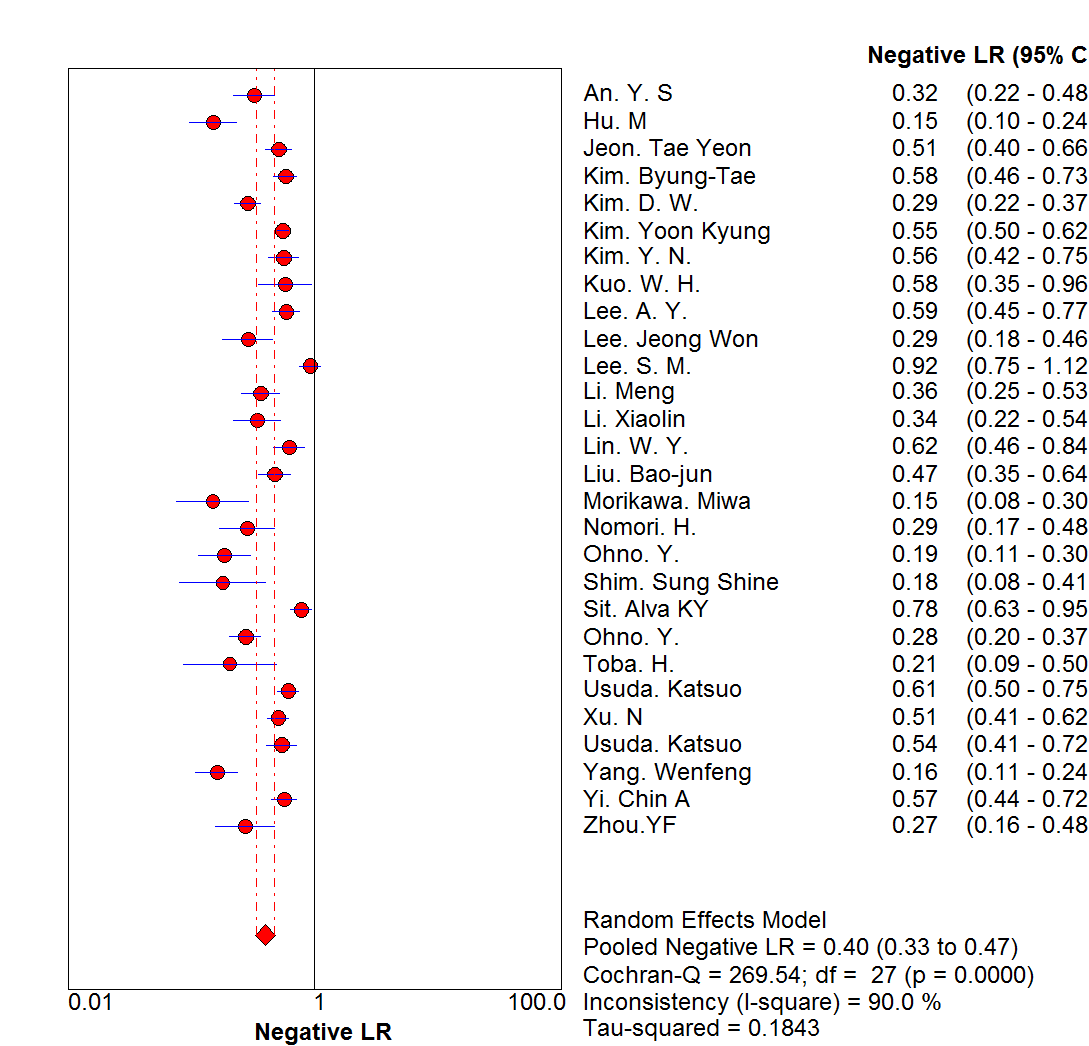

Supplement: S1 File — (ZIP) [file pone.0299045.s001.zip › statistical analysis/PET╩2╛▌/╤╟╫Θ╖╓╬÷/country Asian/nlr.bmp]

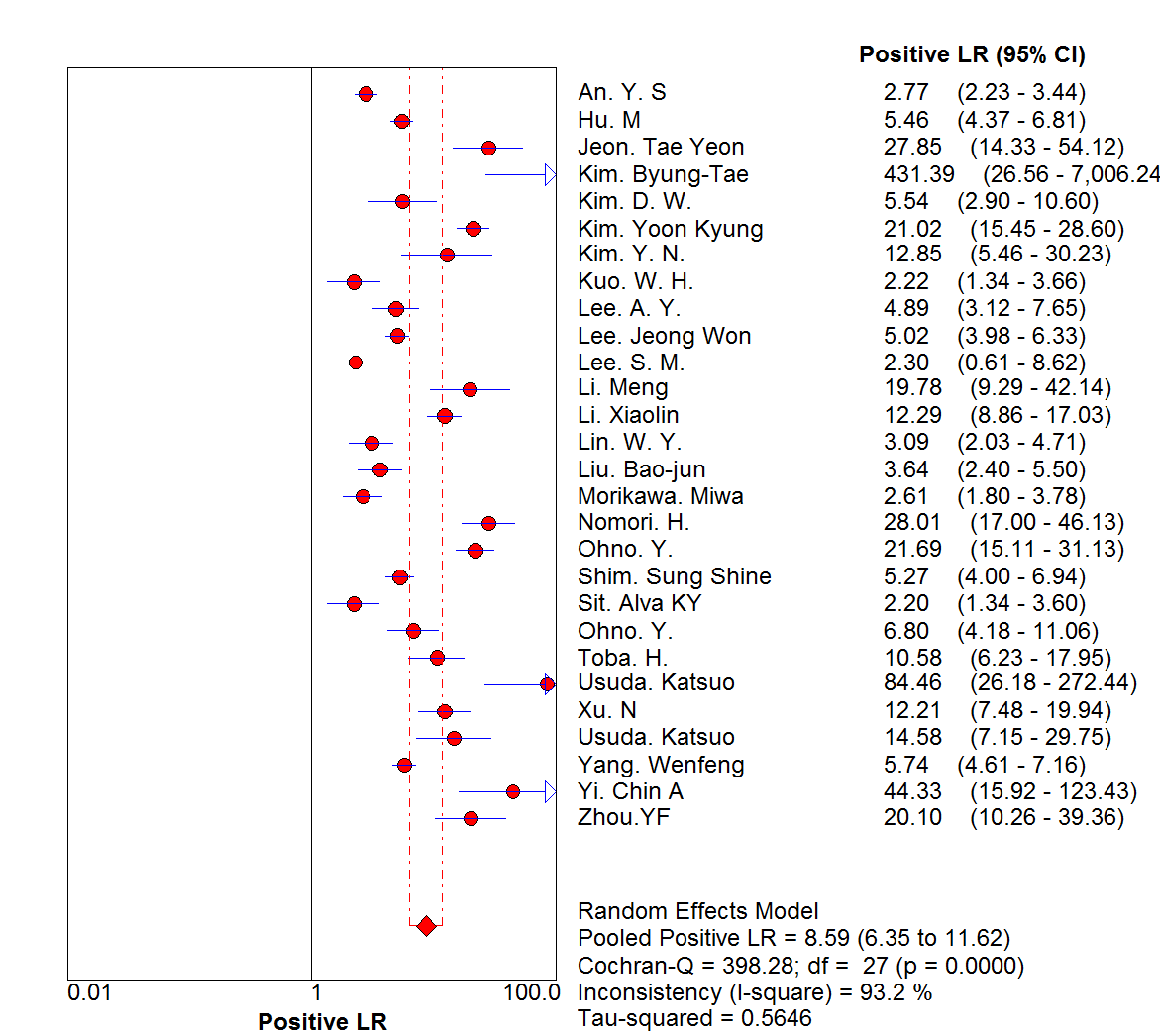

Supplement: S1 File — (ZIP) [file pone.0299045.s001.zip › statistical analysis/PET╩2╛▌/╤╟╫Θ╖╓╬÷/country Asian/plr.bmp]

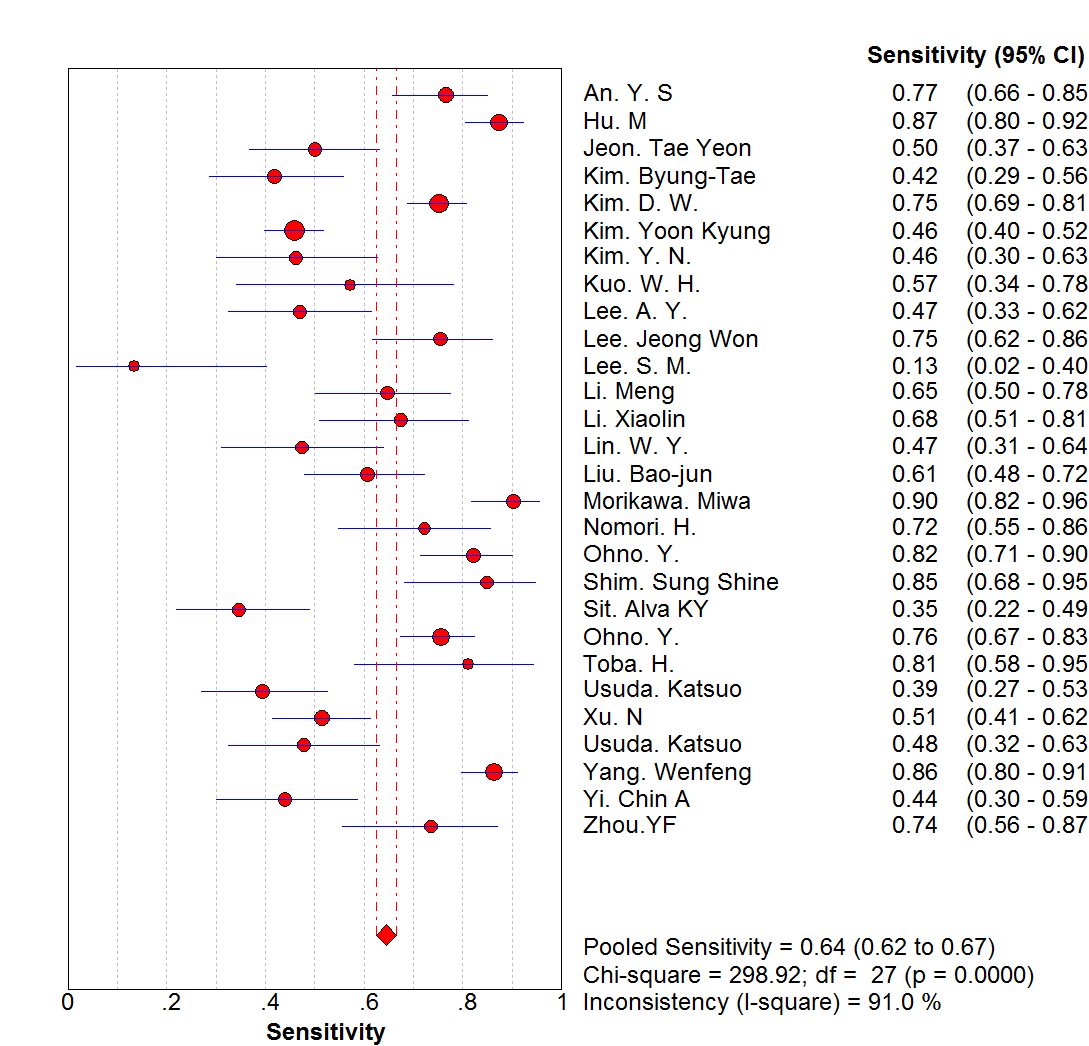

Supplement: S1 File — (ZIP) [file pone.0299045.s001.zip › statistical analysis/PET╩2╛▌/╤╟╫Θ╖╓╬÷/country Asian/sen.bmp]

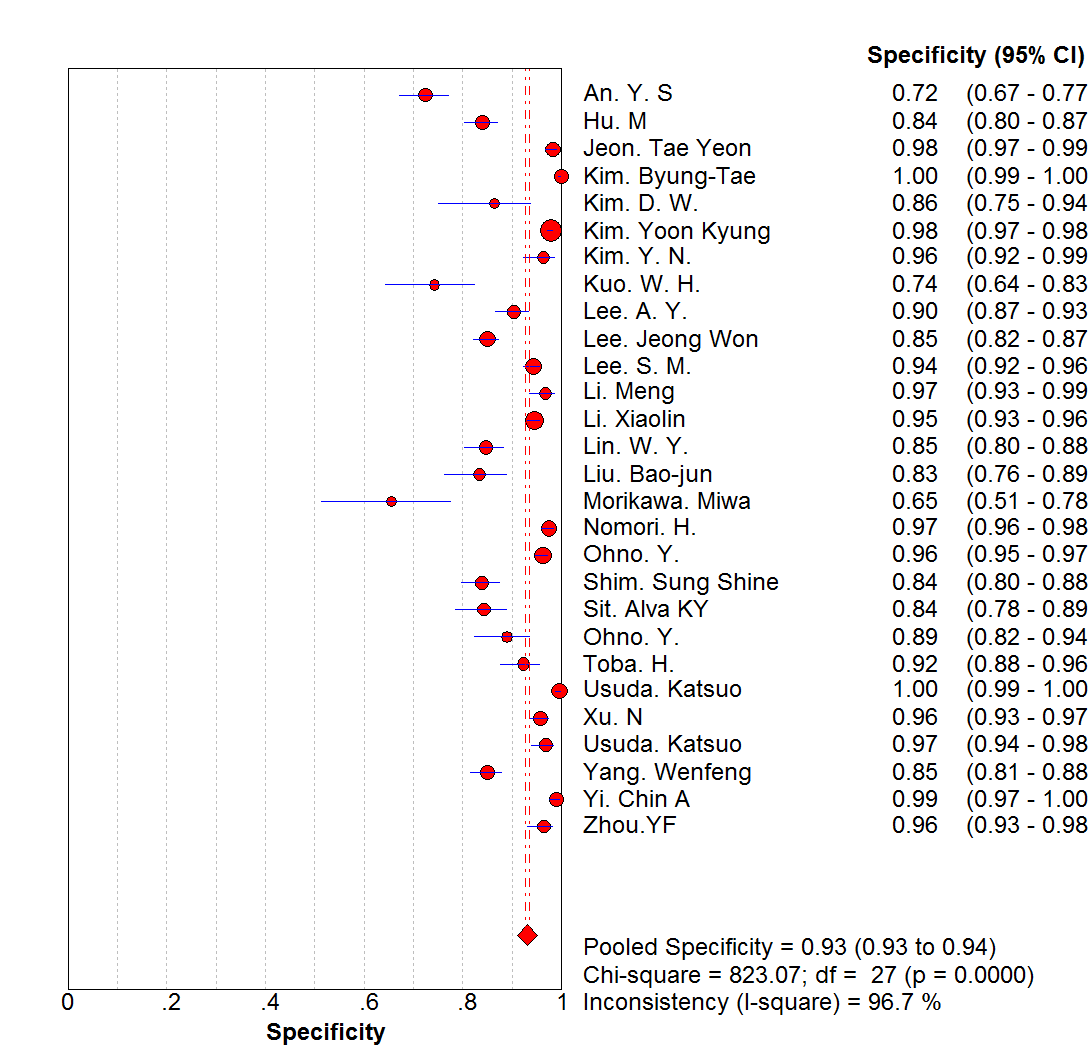

Supplement: S1 File — (ZIP) [file pone.0299045.s001.zip › statistical analysis/PET╩2╛▌/╤╟╫Θ╖╓╬÷/country Asian/spe.bmp]

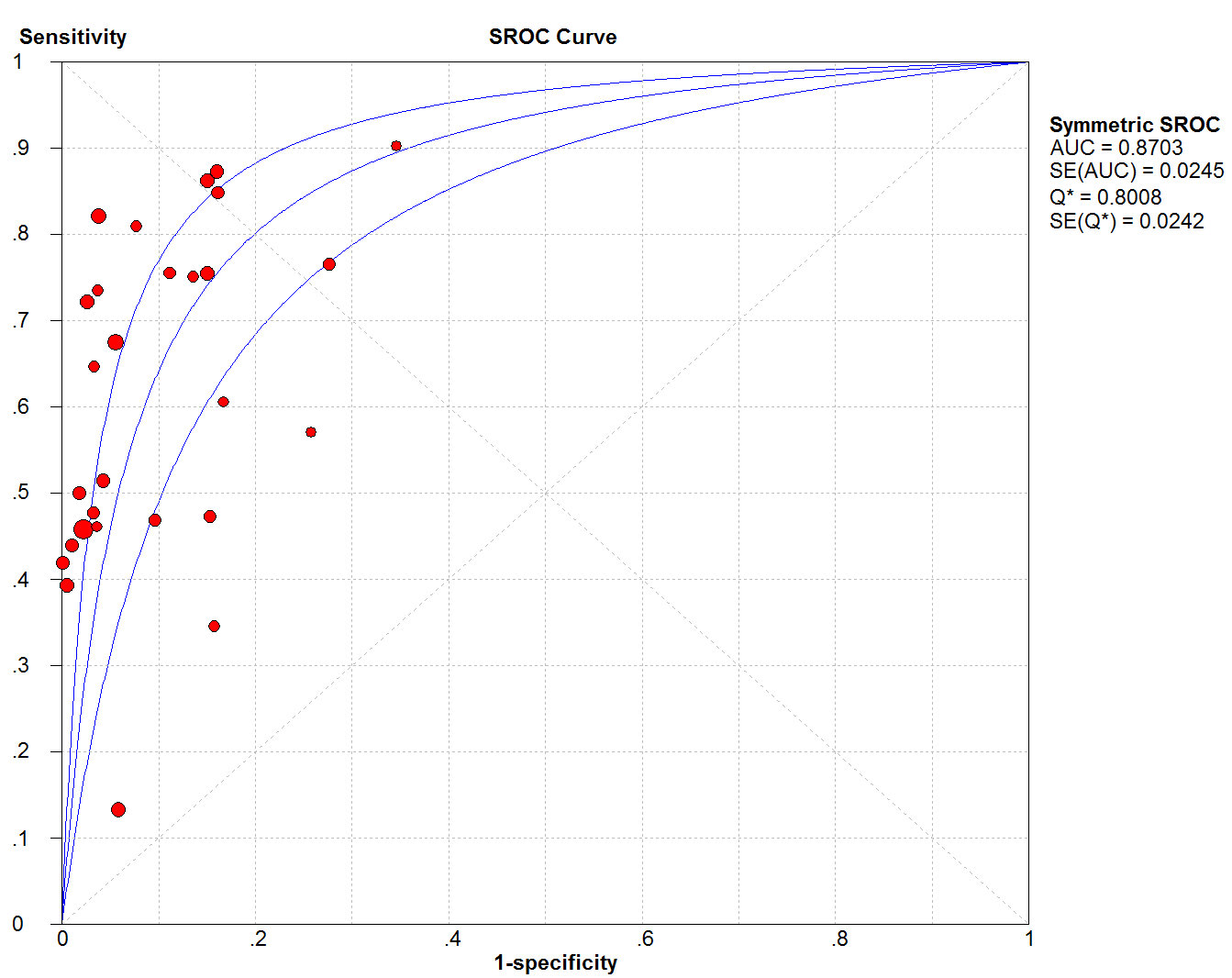

Supplement: S1 File — (ZIP) [file pone.0299045.s001.zip › statistical analysis/PET╩2╛▌/╤╟╫Θ╖╓╬÷/country Asian/sroc.bmp]

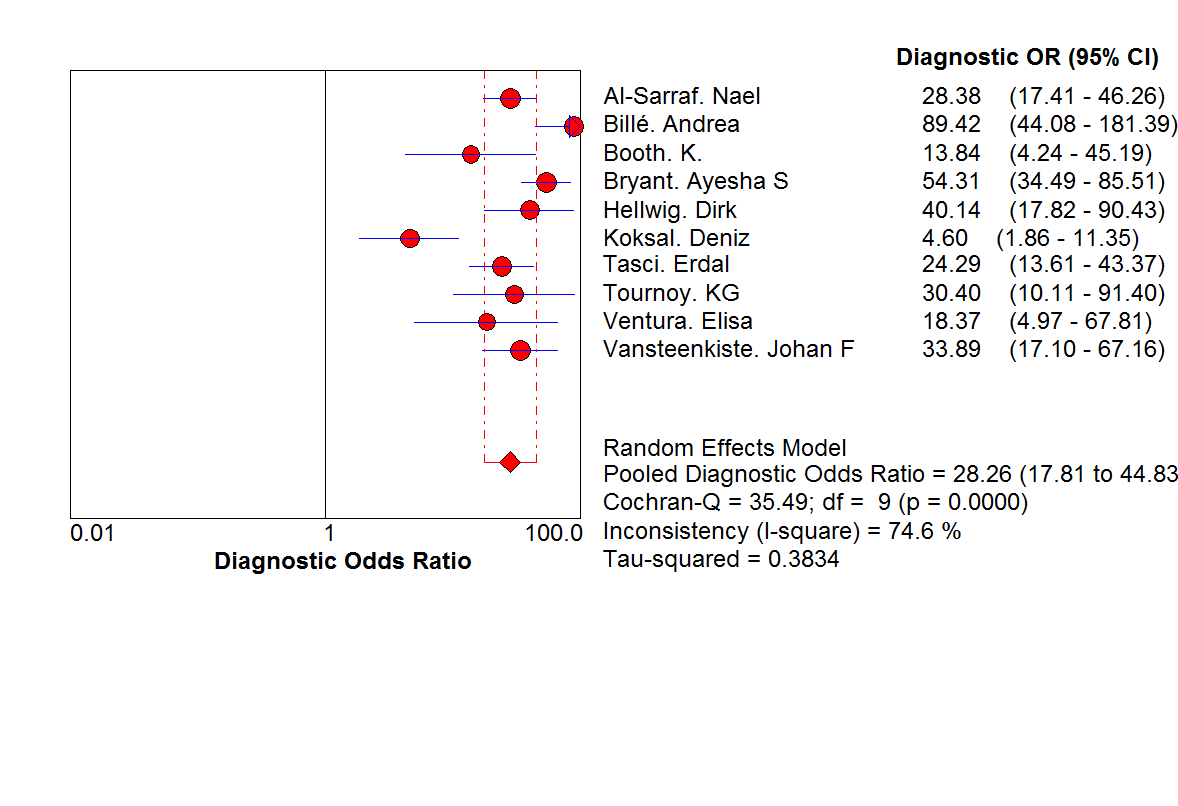

Supplement: S1 File — (ZIP) [file pone.0299045.s001.zip › statistical analysis/PET╩2╛▌/╤╟╫Θ╖╓╬÷/country non-Asian/dor.bmp]

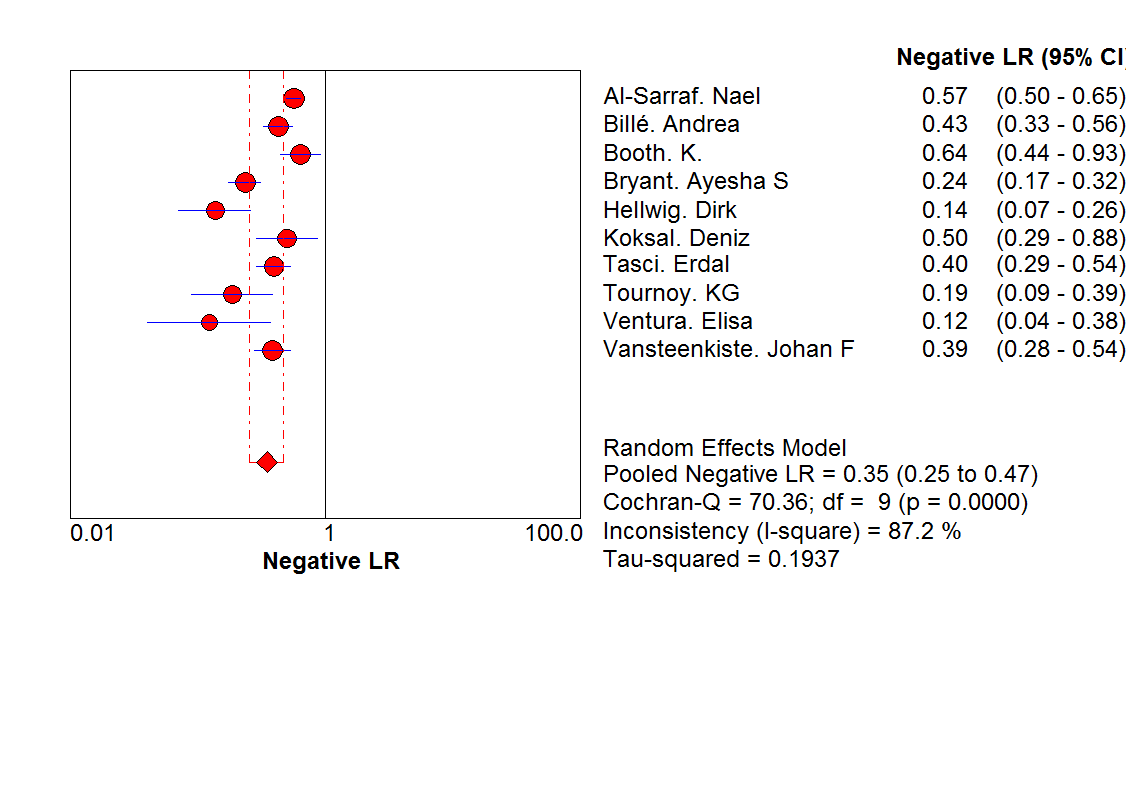

Supplement: S1 File — (ZIP) [file pone.0299045.s001.zip › statistical analysis/PET╩2╛▌/╤╟╫Θ╖╓╬÷/country non-Asian/nlr.bmp]

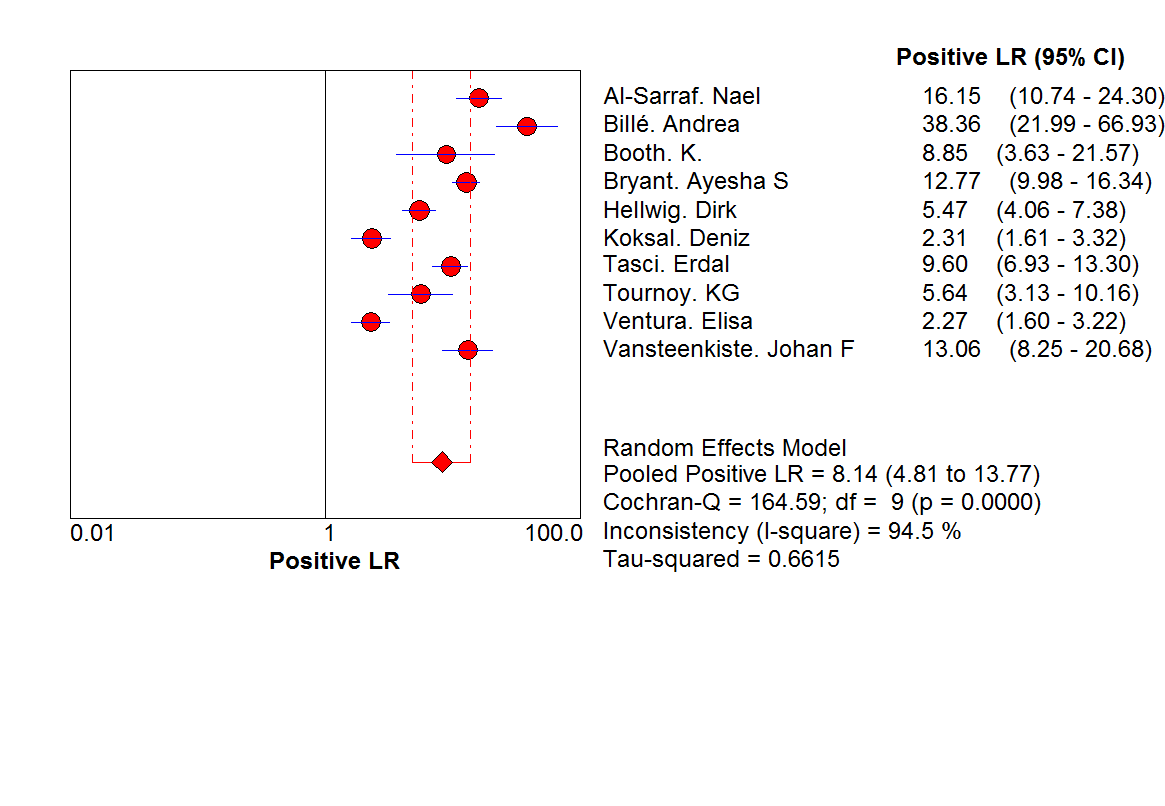

Supplement: S1 File — (ZIP) [file pone.0299045.s001.zip › statistical analysis/PET╩2╛▌/╤╟╫Θ╖╓╬÷/country non-Asian/plr.bmp]

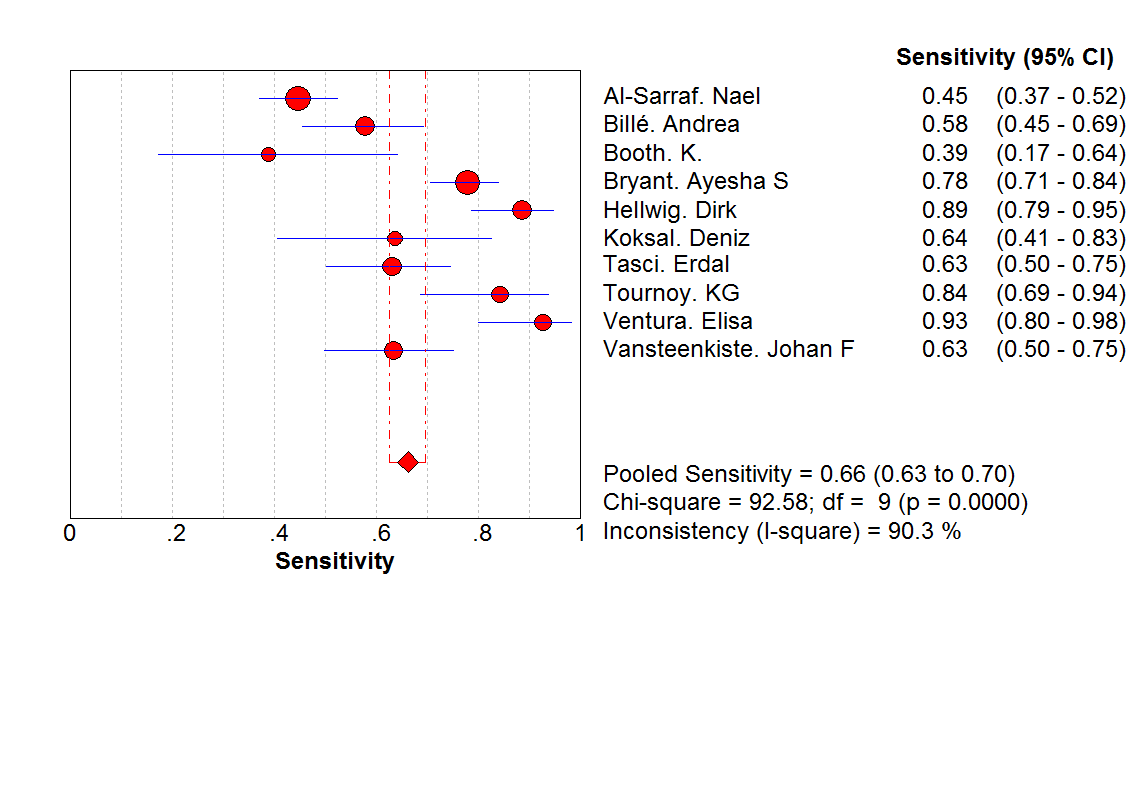

Supplement: S1 File — (ZIP) [file pone.0299045.s001.zip › statistical analysis/PET╩2╛▌/╤╟╫Θ╖╓╬÷/country non-Asian/sen.bmp]

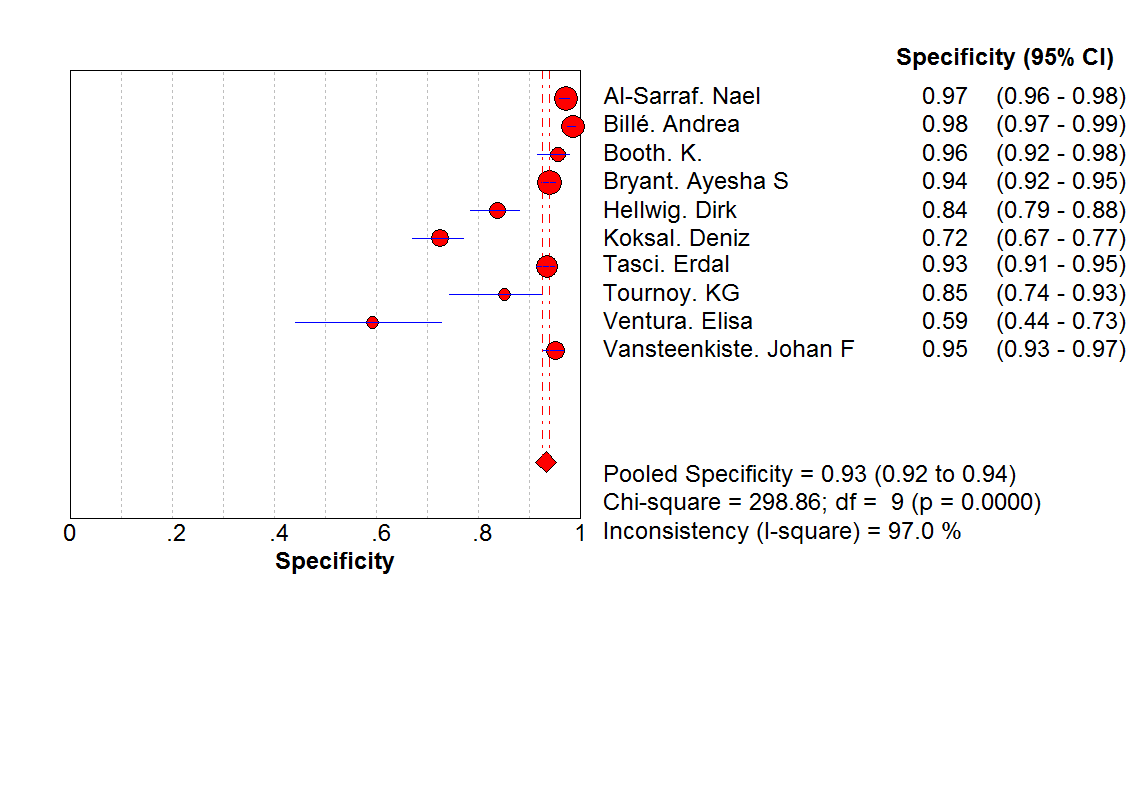

Supplement: S1 File — (ZIP) [file pone.0299045.s001.zip › statistical analysis/PET╩2╛▌/╤╟╫Θ╖╓╬÷/country non-Asian/spe.bmp]

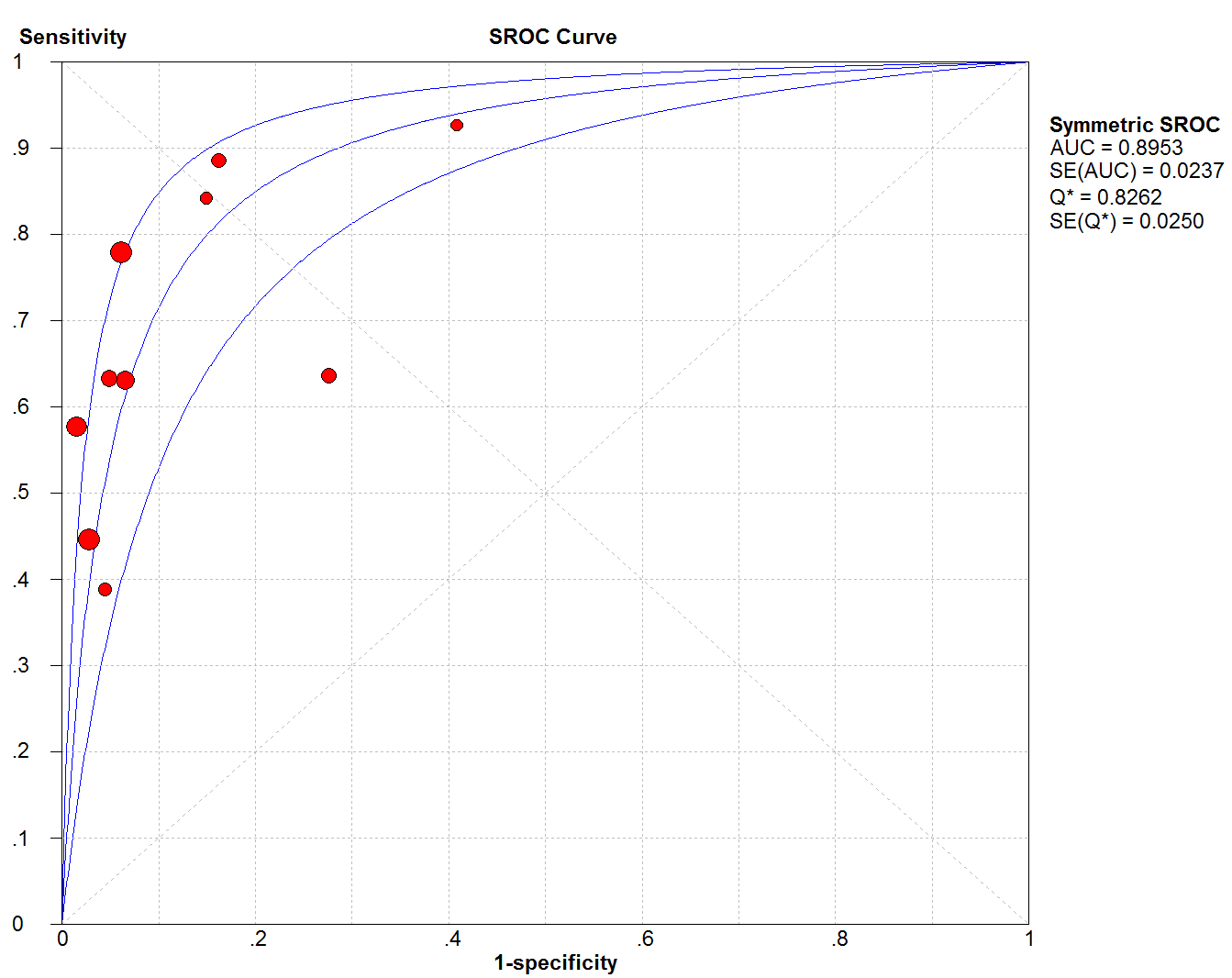

Supplement: S1 File — (ZIP) [file pone.0299045.s001.zip › statistical analysis/PET╩2╛▌/╤╟╫Θ╖╓╬÷/country non-Asian/sroc.bmp]

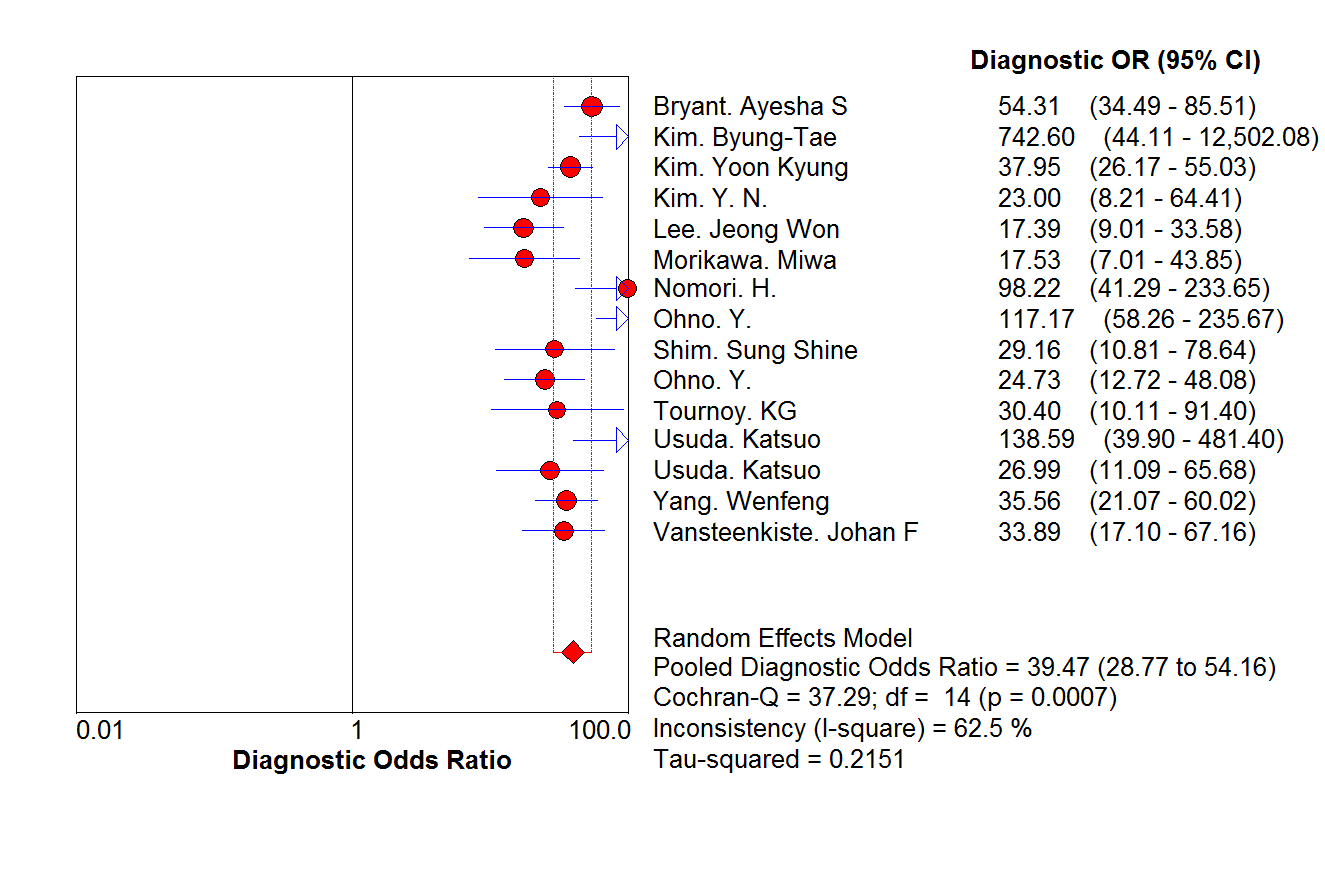

Supplement: S1 File — (ZIP) [file pone.0299045.s001.zip › statistical analysis/PET╩2╛▌/╤╟╫Θ╖╓╬÷/design prospective/dor.bmp]

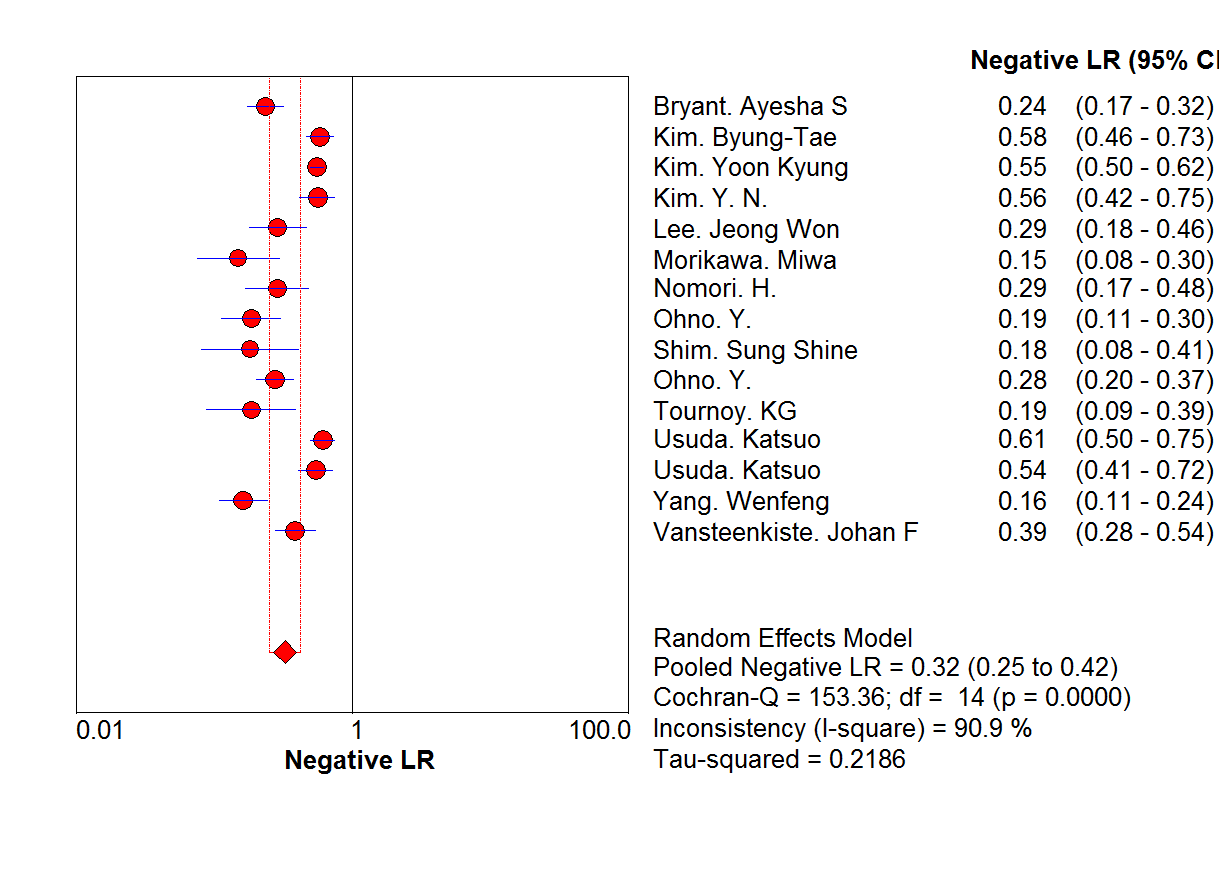

Supplement: S1 File — (ZIP) [file pone.0299045.s001.zip › statistical analysis/PET╩2╛▌/╤╟╫Θ╖╓╬÷/design prospective/nlr.bmp]

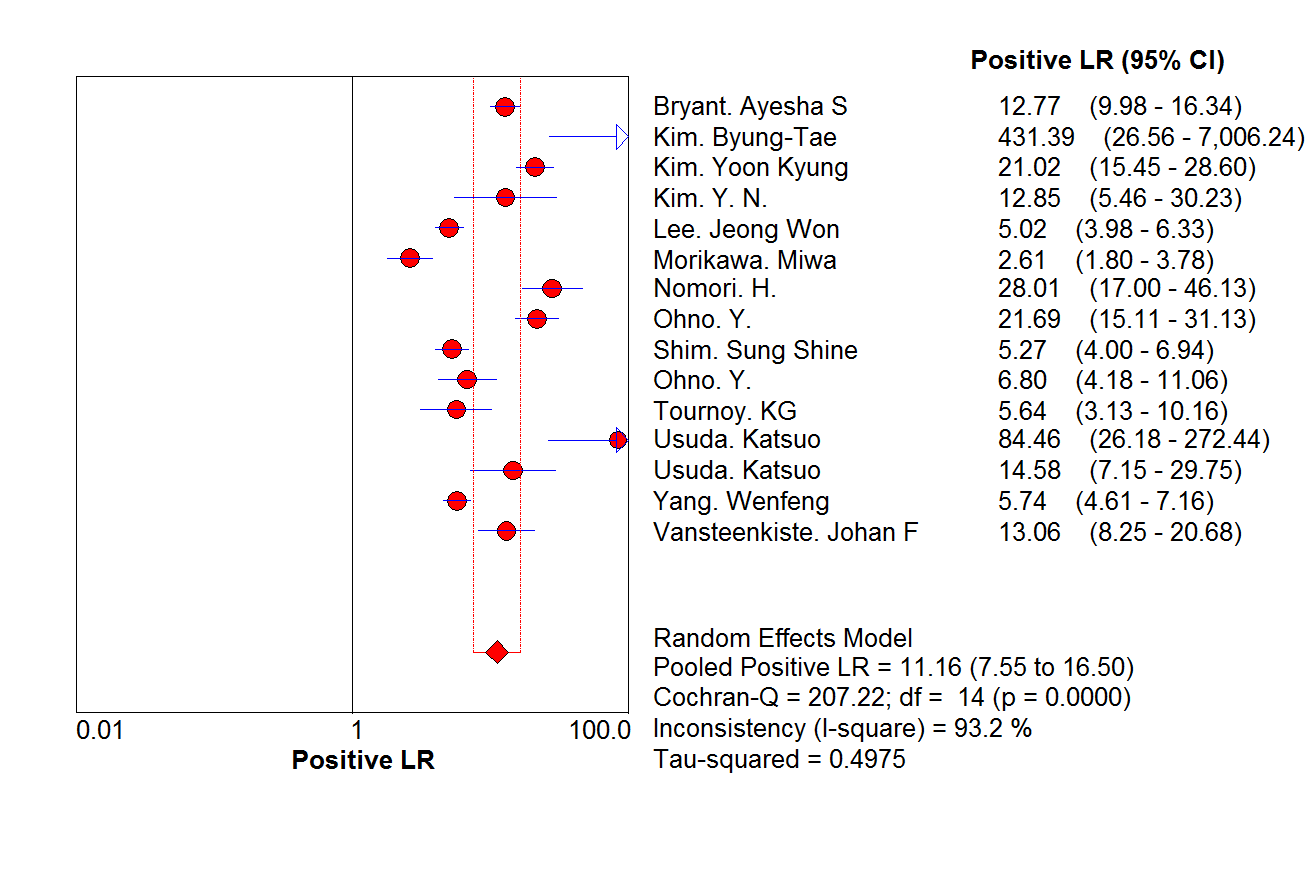

Supplement: S1 File — (ZIP) [file pone.0299045.s001.zip › statistical analysis/PET╩2╛▌/╤╟╫Θ╖╓╬÷/design prospective/plr.bmp]

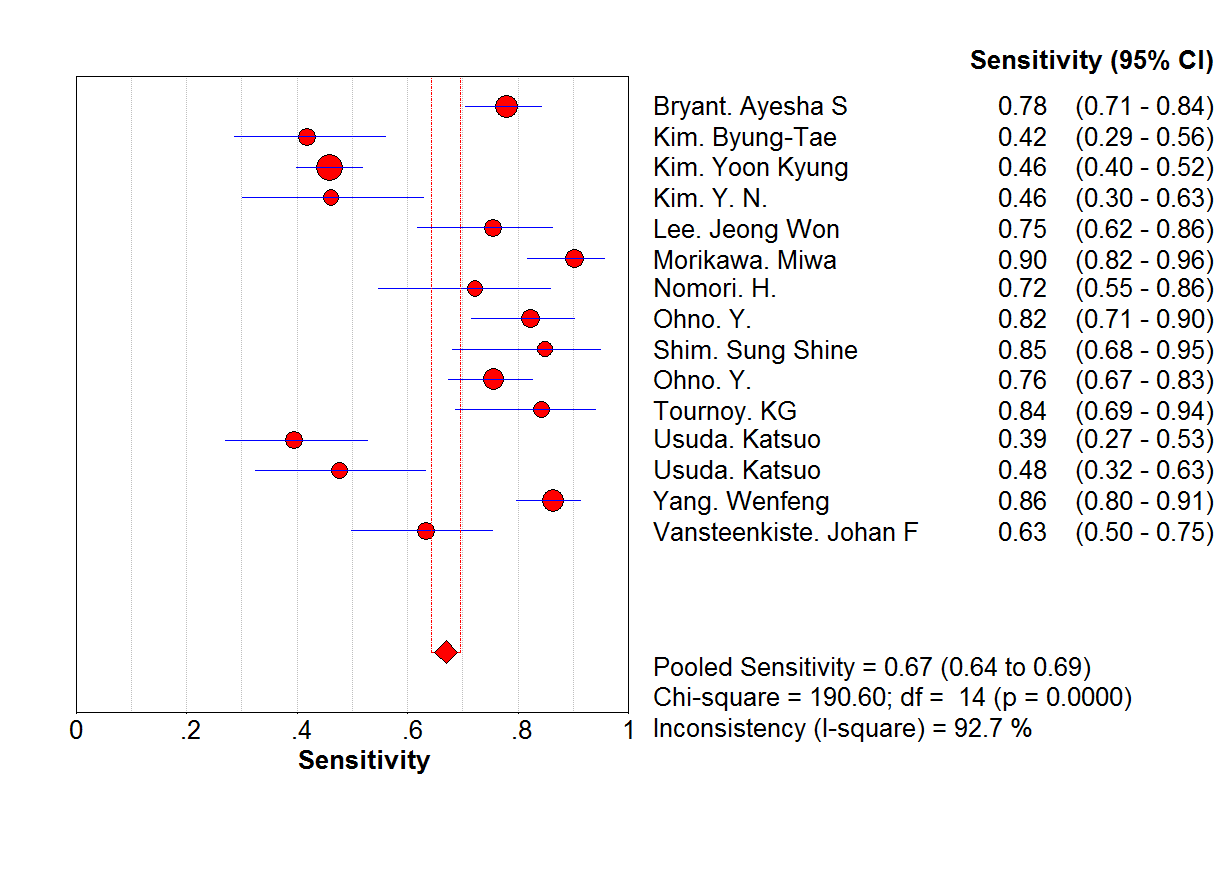

Supplement: S1 File — (ZIP) [file pone.0299045.s001.zip › statistical analysis/PET╩2╛▌/╤╟╫Θ╖╓╬÷/design prospective/sen.bmp]

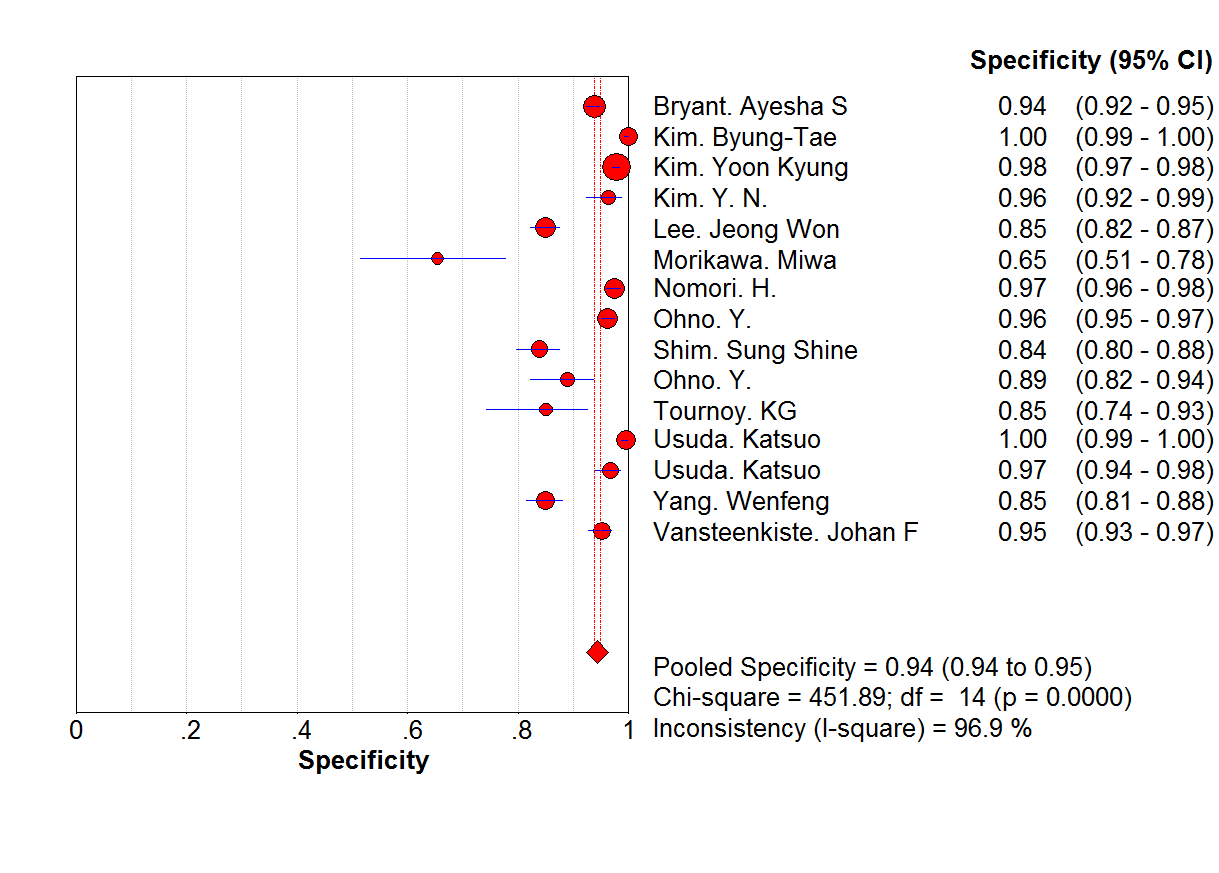

Supplement: S1 File — (ZIP) [file pone.0299045.s001.zip › statistical analysis/PET╩2╛▌/╤╟╫Θ╖╓╬÷/design prospective/spe.bmp]

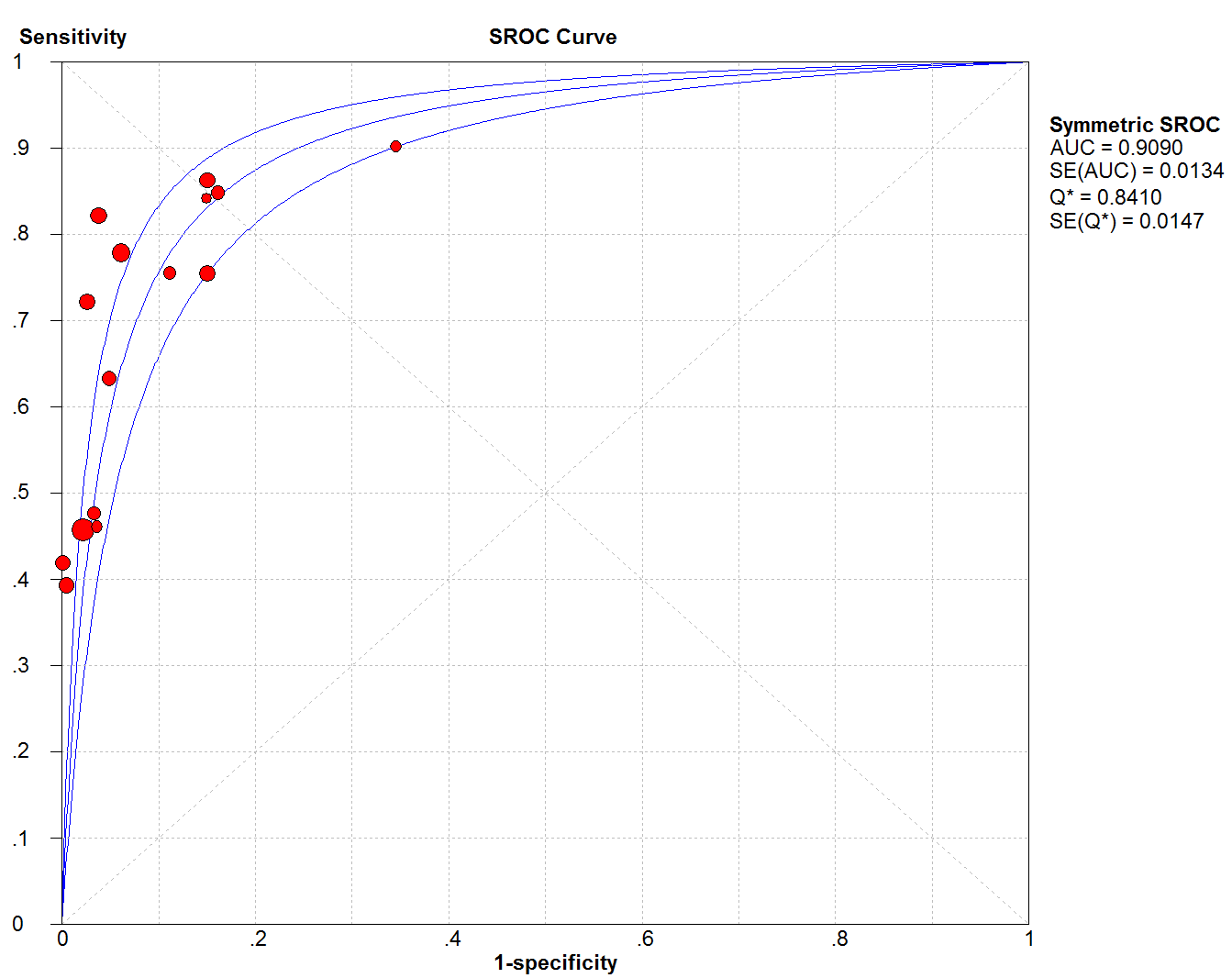

Supplement: S1 File — (ZIP) [file pone.0299045.s001.zip › statistical analysis/PET╩2╛▌/╤╟╫Θ╖╓╬÷/design prospective/sroc.bmp]

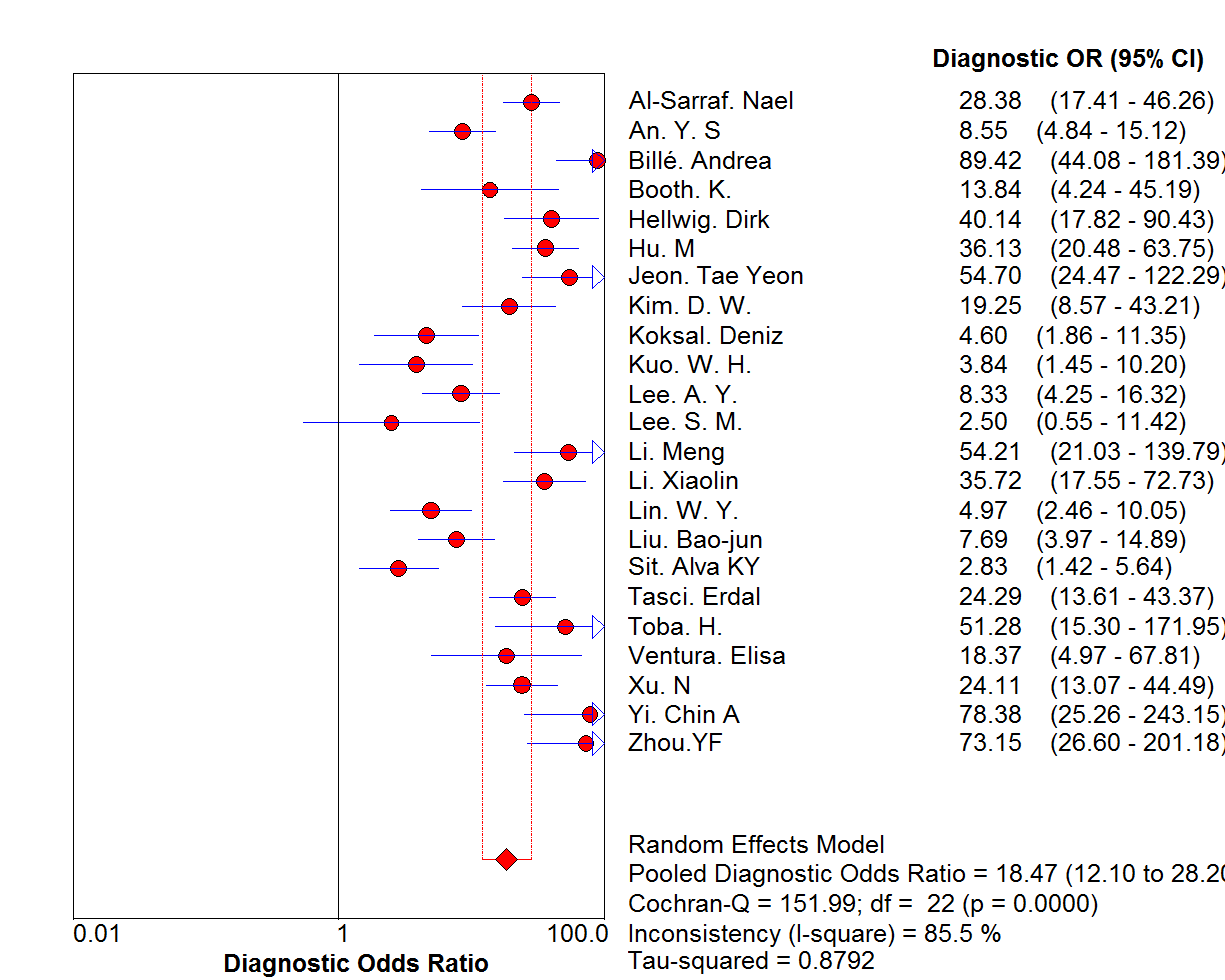

Supplement: S1 File — (ZIP) [file pone.0299045.s001.zip › statistical analysis/PET╩2╛▌/╤╟╫Θ╖╓╬÷/design retrospective/dor.bmp]

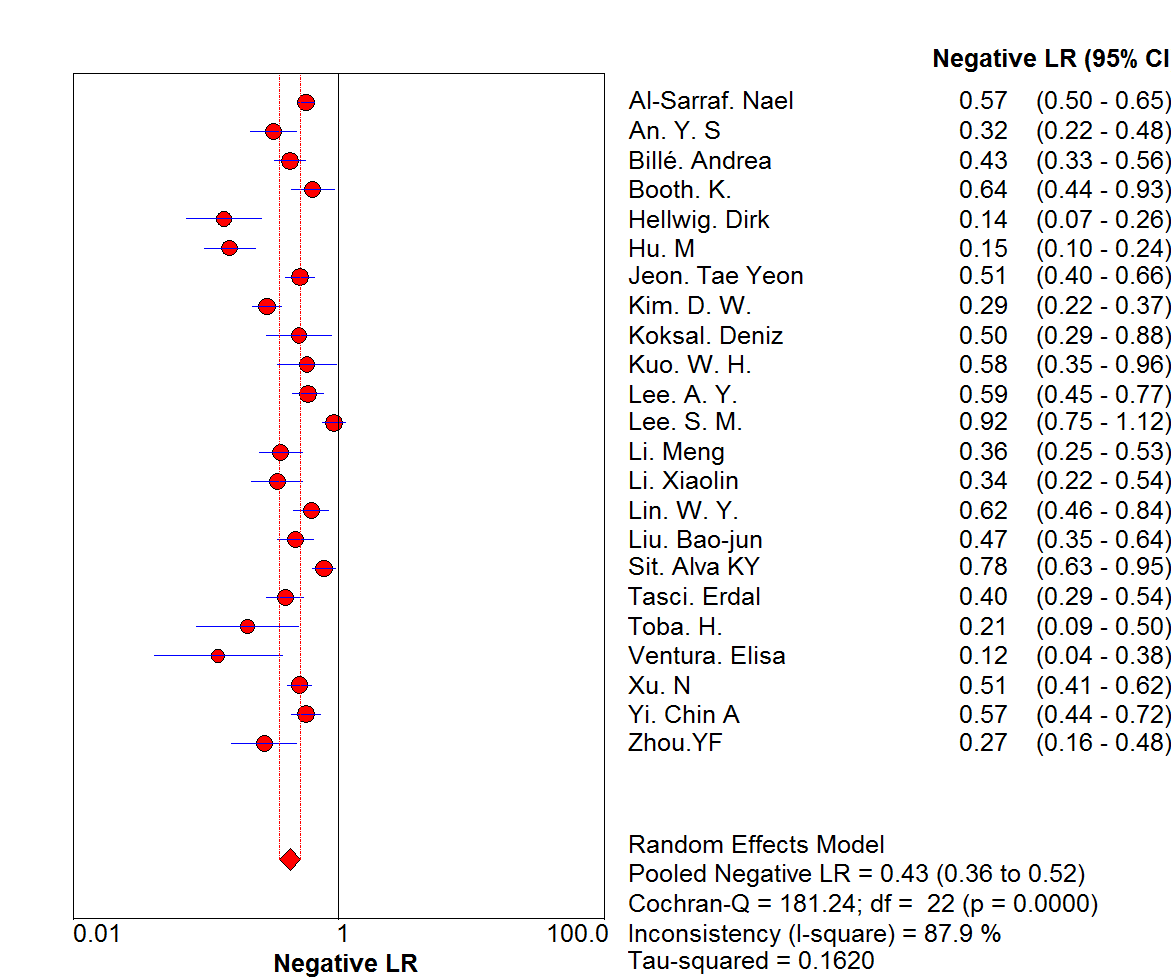

Supplement: S1 File — (ZIP) [file pone.0299045.s001.zip › statistical analysis/PET╩2╛▌/╤╟╫Θ╖╓╬÷/design retrospective/nlr.bmp]

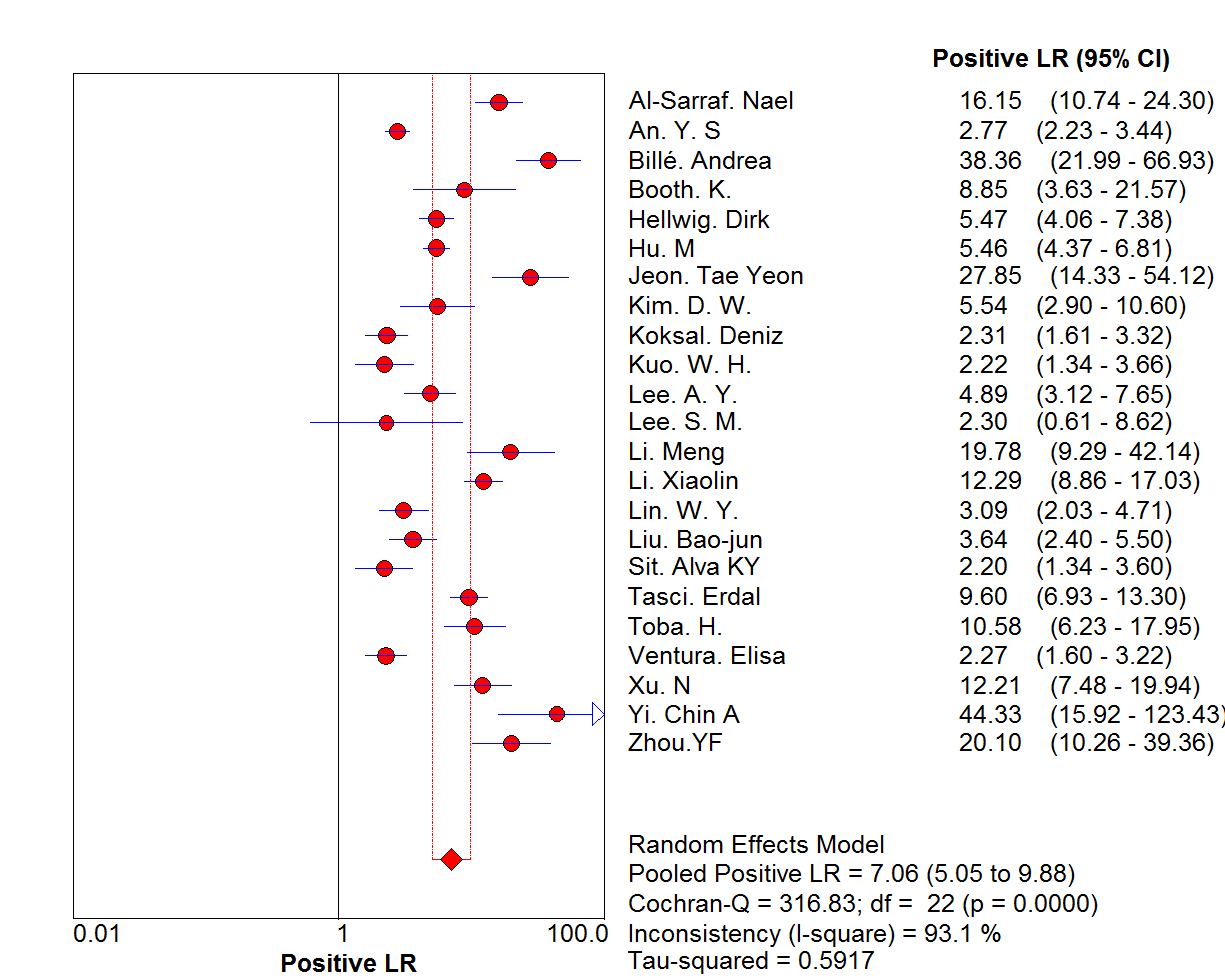

Supplement: S1 File — (ZIP) [file pone.0299045.s001.zip › statistical analysis/PET╩2╛▌/╤╟╫Θ╖╓╬÷/design retrospective/plr.bmp]

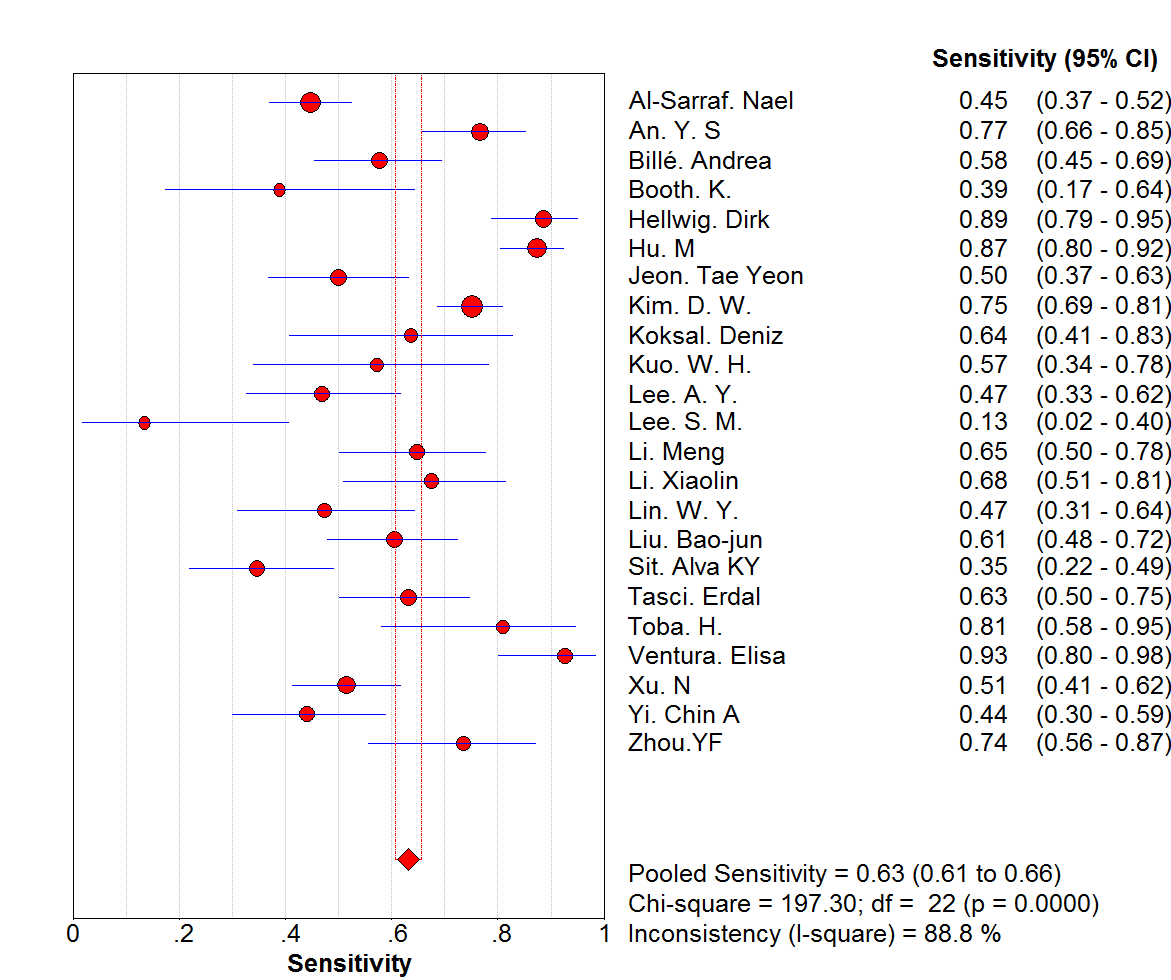

Supplement: S1 File — (ZIP) [file pone.0299045.s001.zip › statistical analysis/PET╩2╛▌/╤╟╫Θ╖╓╬÷/design retrospective/sen.bmp]

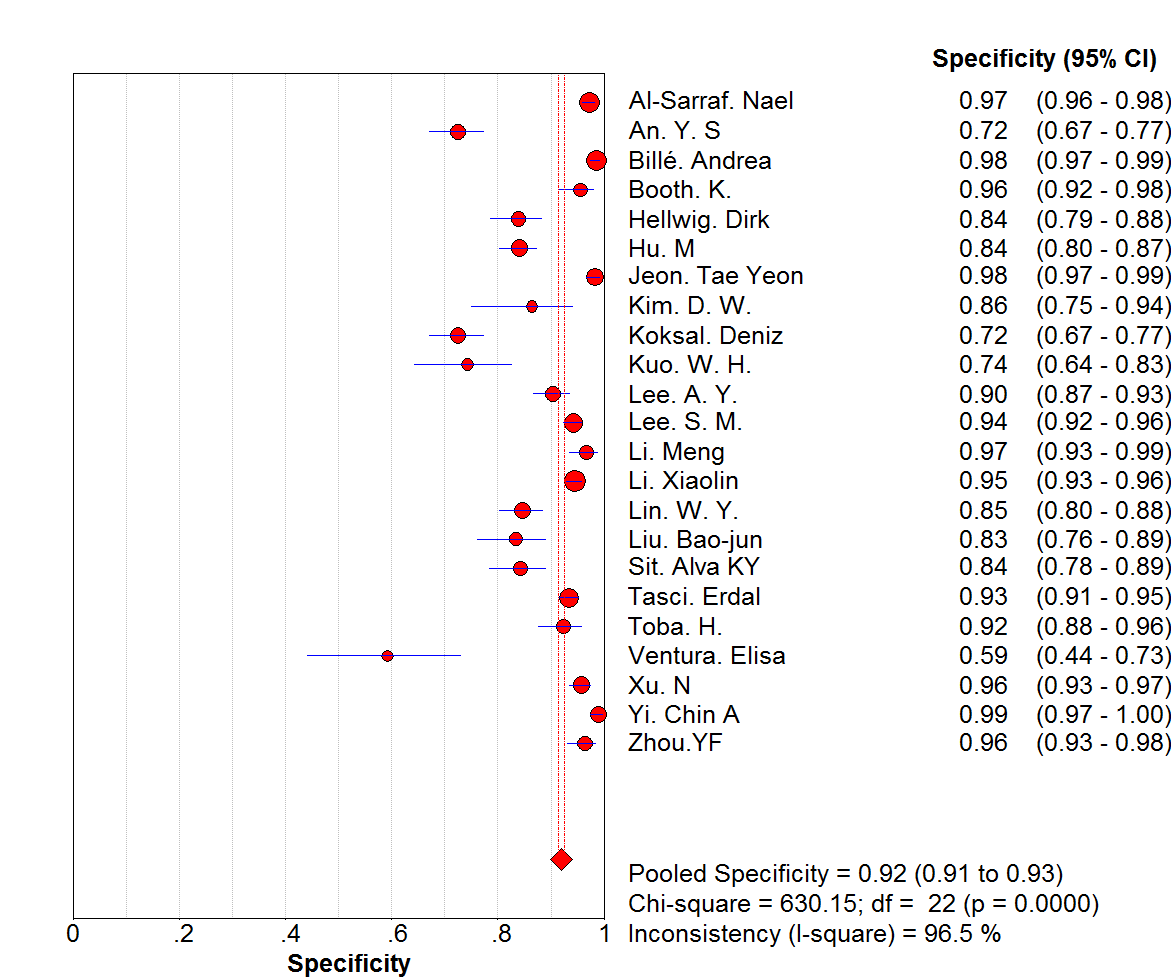

Supplement: S1 File — (ZIP) [file pone.0299045.s001.zip › statistical analysis/PET╩2╛▌/╤╟╫Θ╖╓╬÷/design retrospective/spe.bmp]

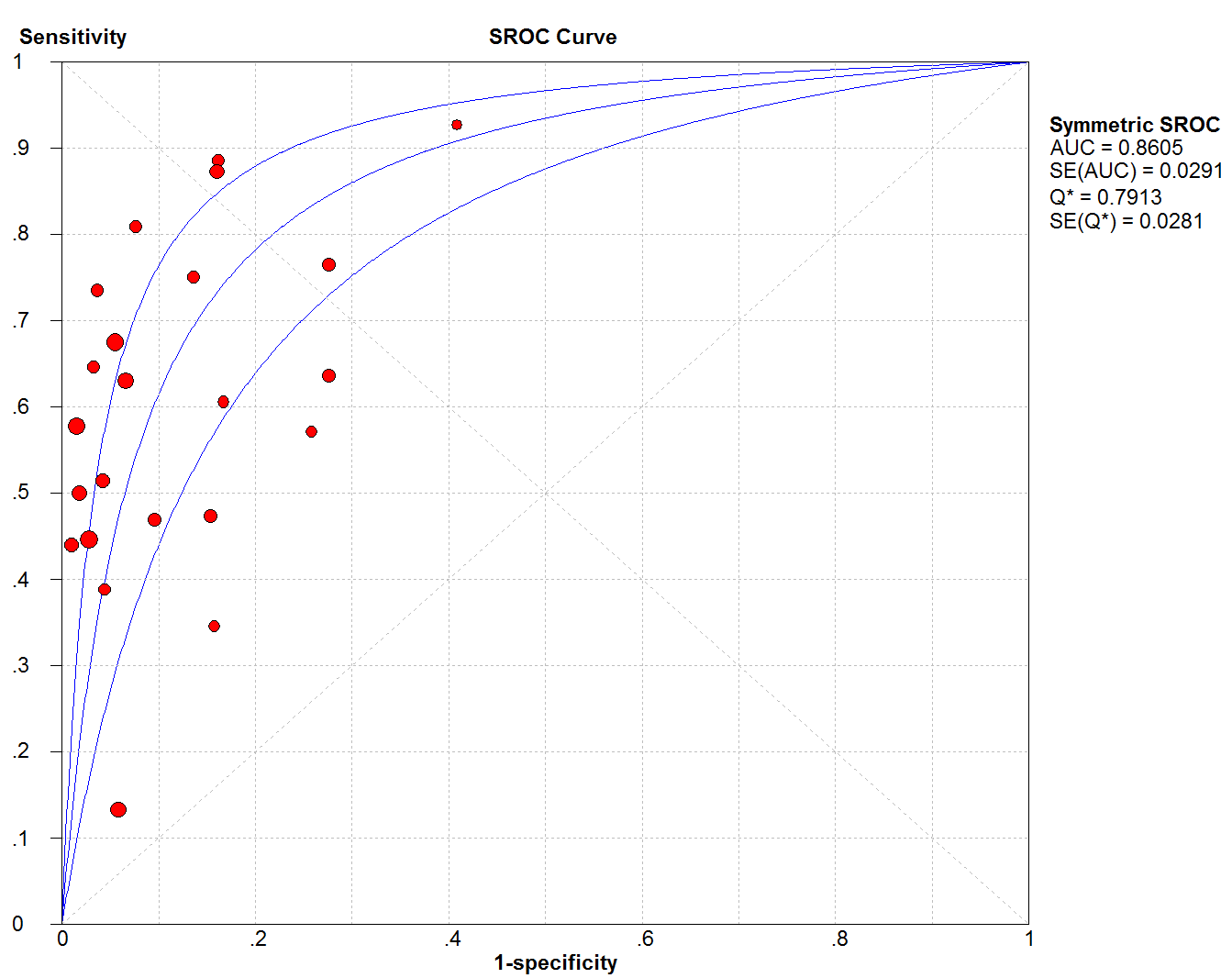

Supplement: S1 File — (ZIP) [file pone.0299045.s001.zip › statistical analysis/PET╩2╛▌/╤╟╫Θ╖╓╬÷/design retrospective/sroc.bmp]

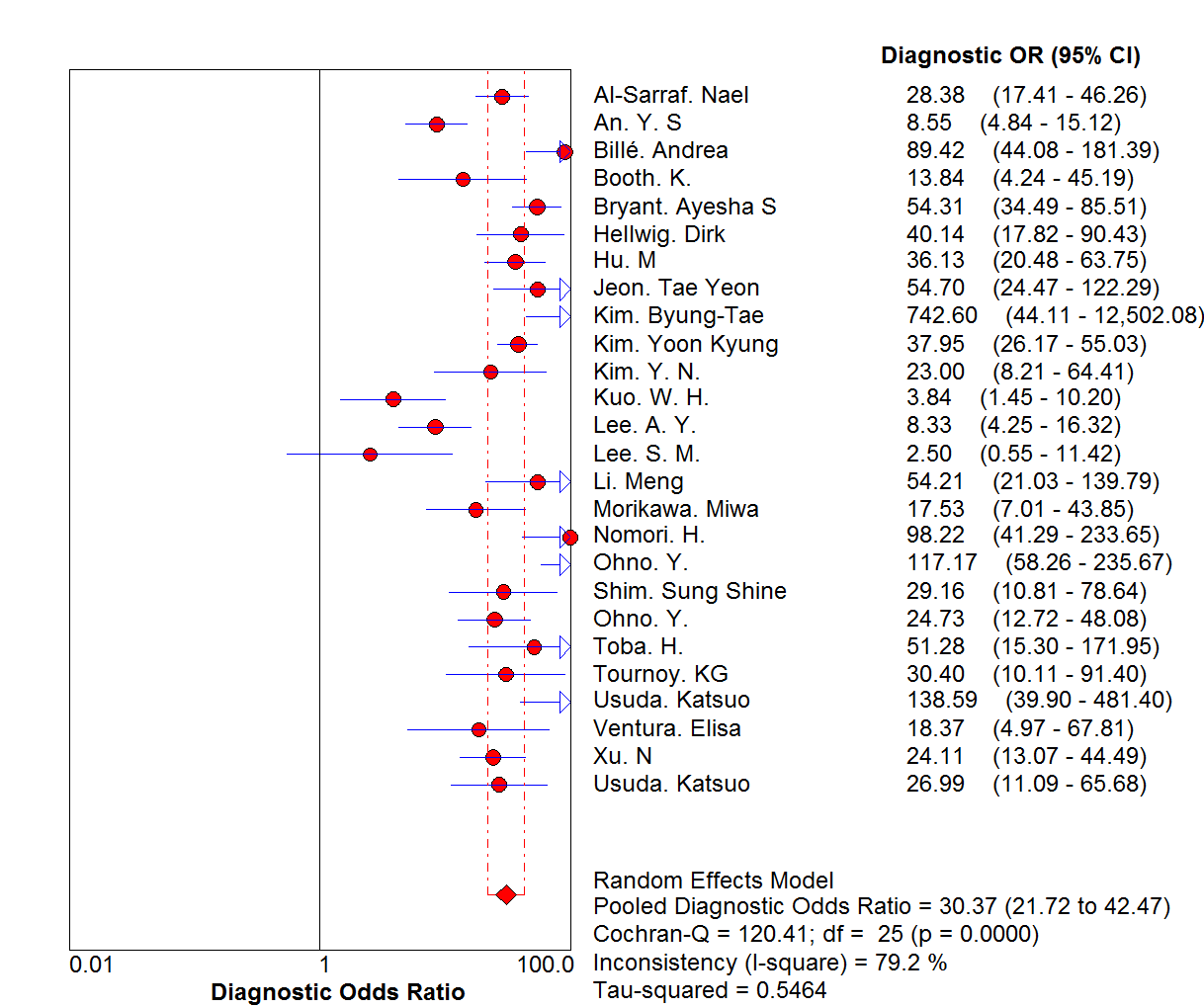

Supplement: S1 File — (ZIP) [file pone.0299045.s001.zip › statistical analysis/PET╩2╛▌/╤╟╫Θ╖╓╬÷/enrollment consecutive/dor.bmp]

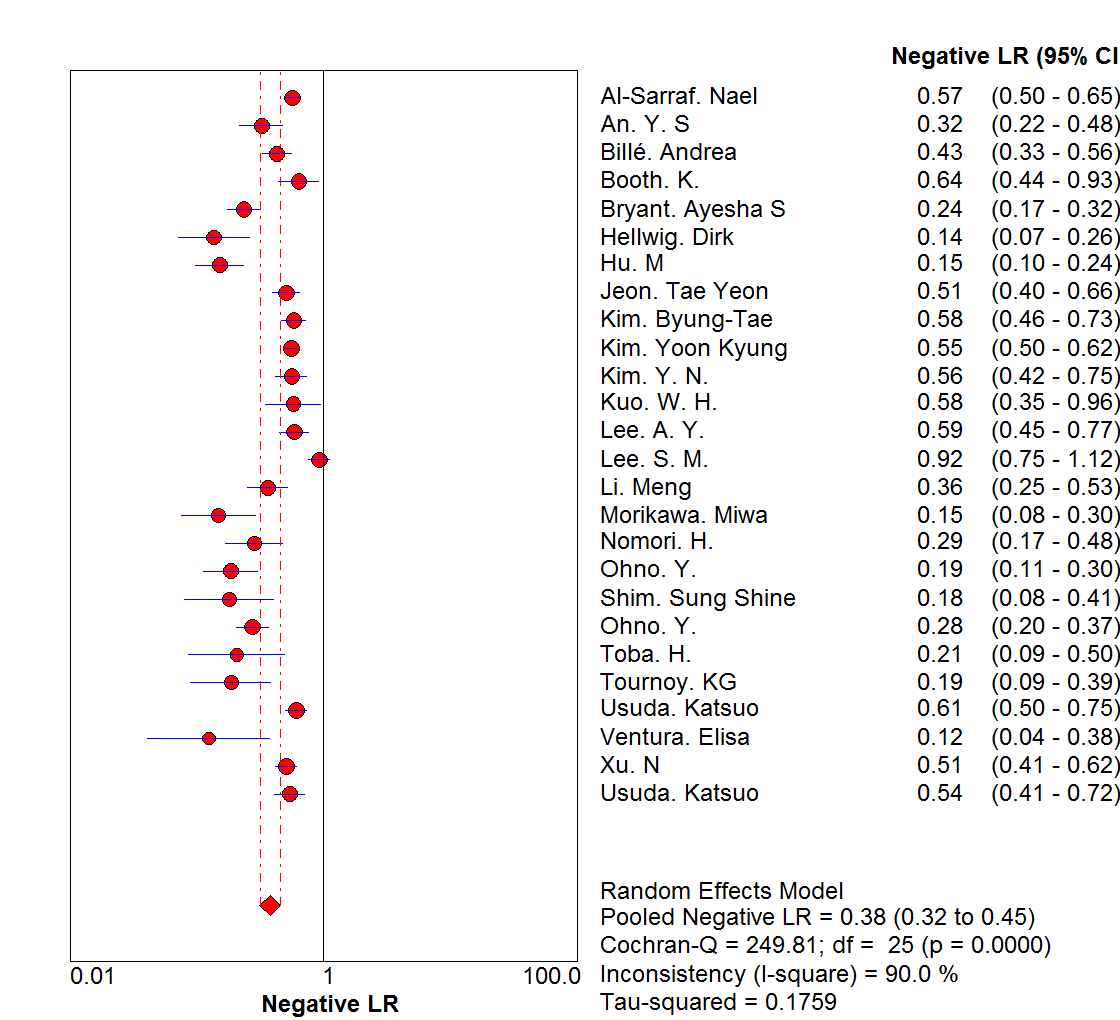

Supplement: S1 File — (ZIP) [file pone.0299045.s001.zip › statistical analysis/PET╩2╛▌/╤╟╫Θ╖╓╬÷/enrollment consecutive/nlr.bmp]

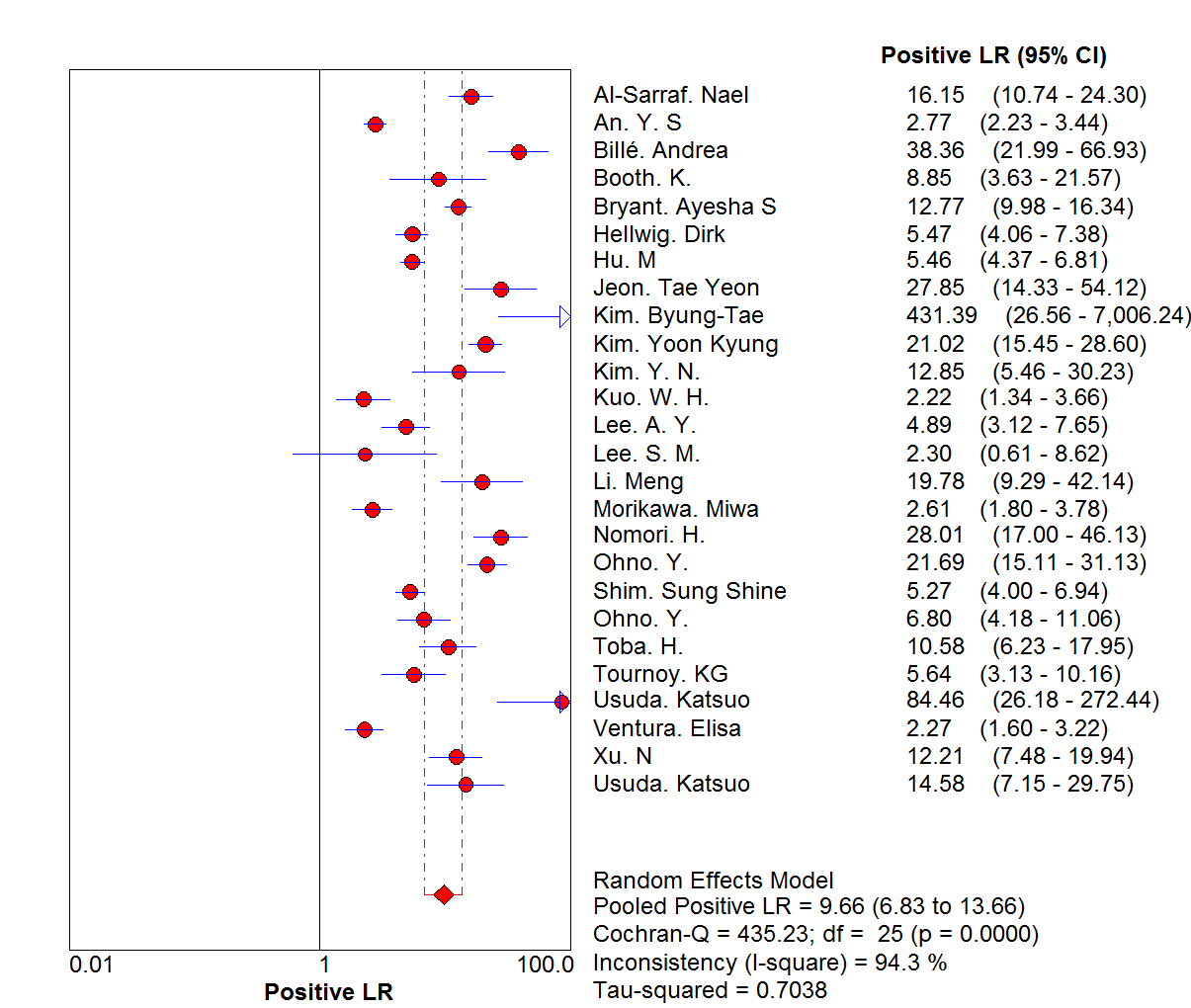

Supplement: S1 File — (ZIP) [file pone.0299045.s001.zip › statistical analysis/PET╩2╛▌/╤╟╫Θ╖╓╬÷/enrollment consecutive/plr.bmp]

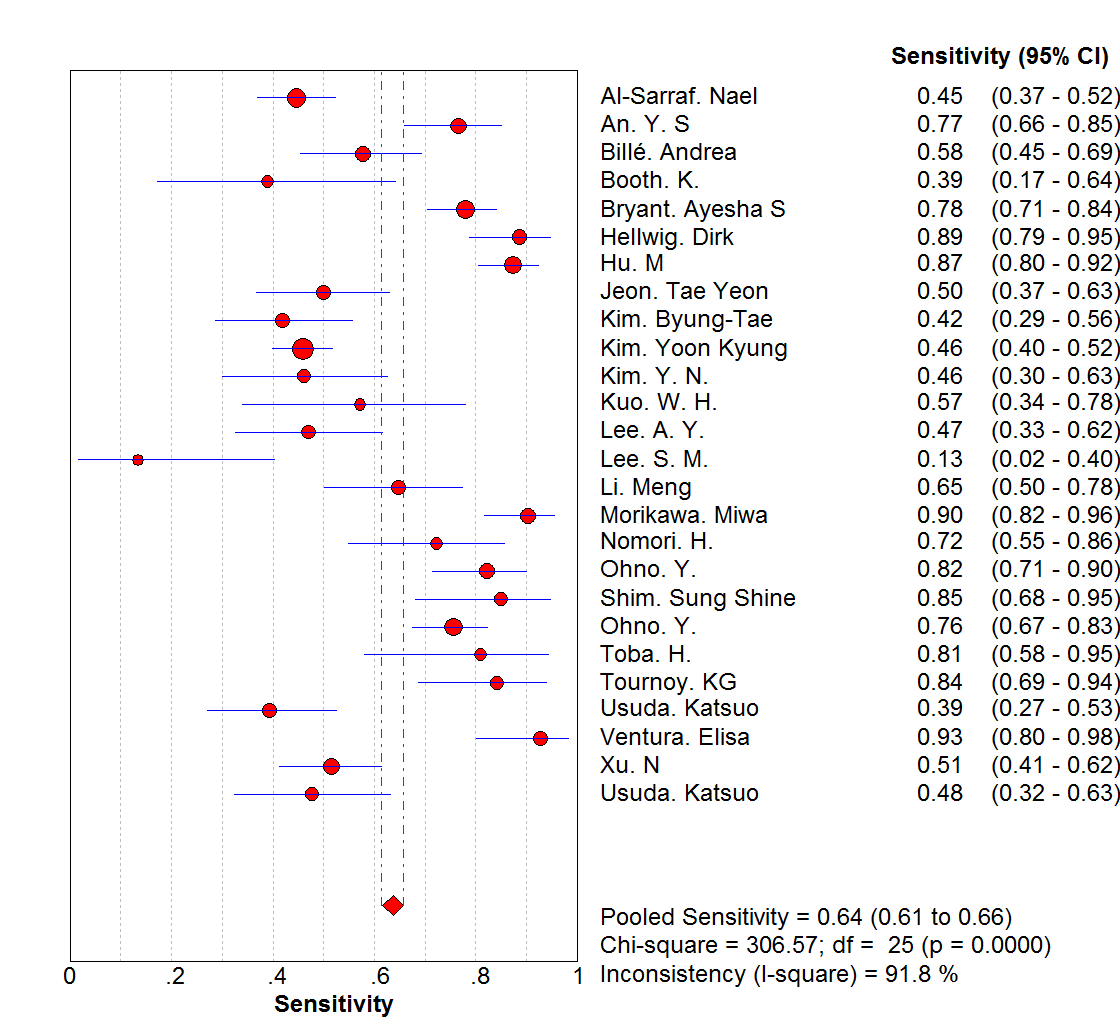

Supplement: S1 File — (ZIP) [file pone.0299045.s001.zip › statistical analysis/PET╩2╛▌/╤╟╫Θ╖╓╬÷/enrollment consecutive/sen.bmp]

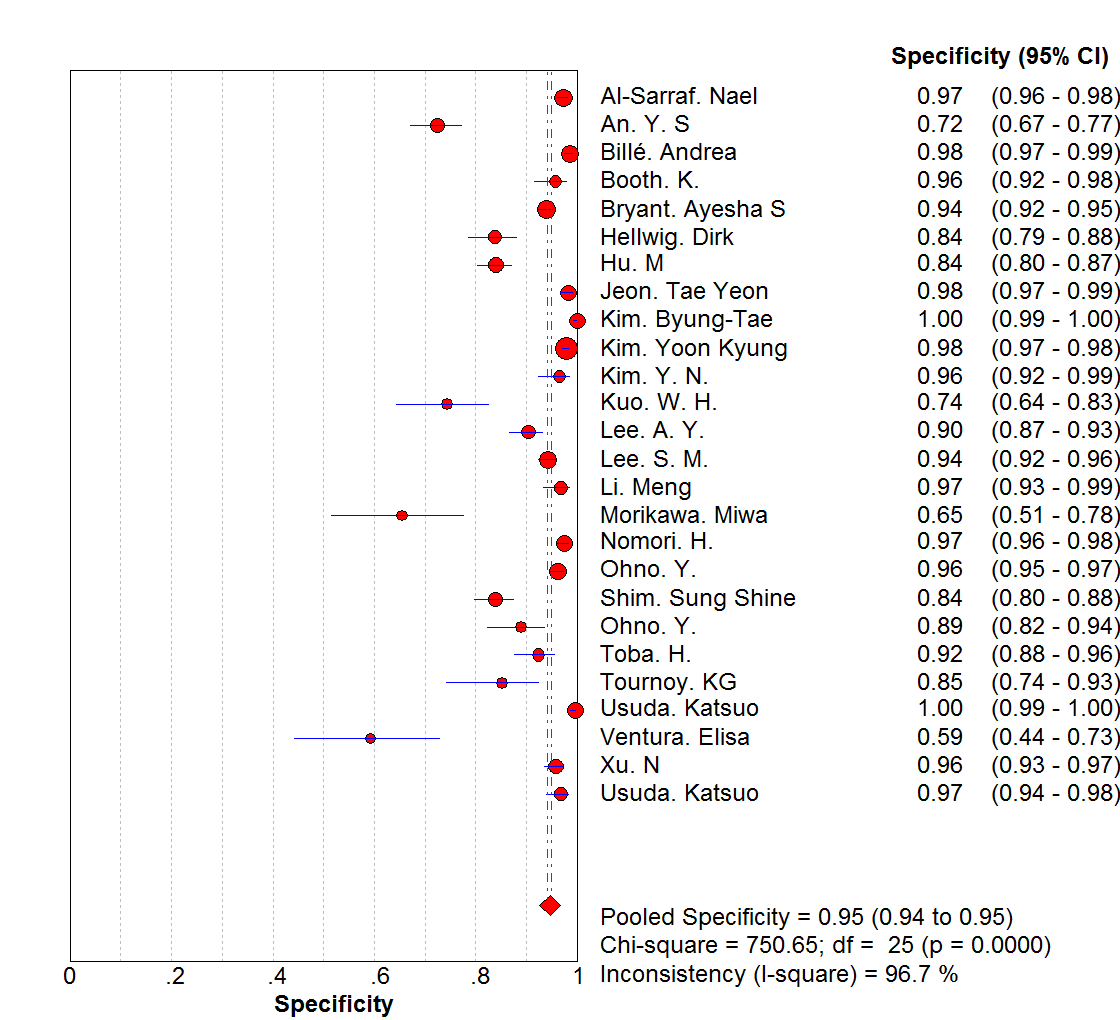

Supplement: S1 File — (ZIP) [file pone.0299045.s001.zip › statistical analysis/PET╩2╛▌/╤╟╫Θ╖╓╬÷/enrollment consecutive/spe.bmp]

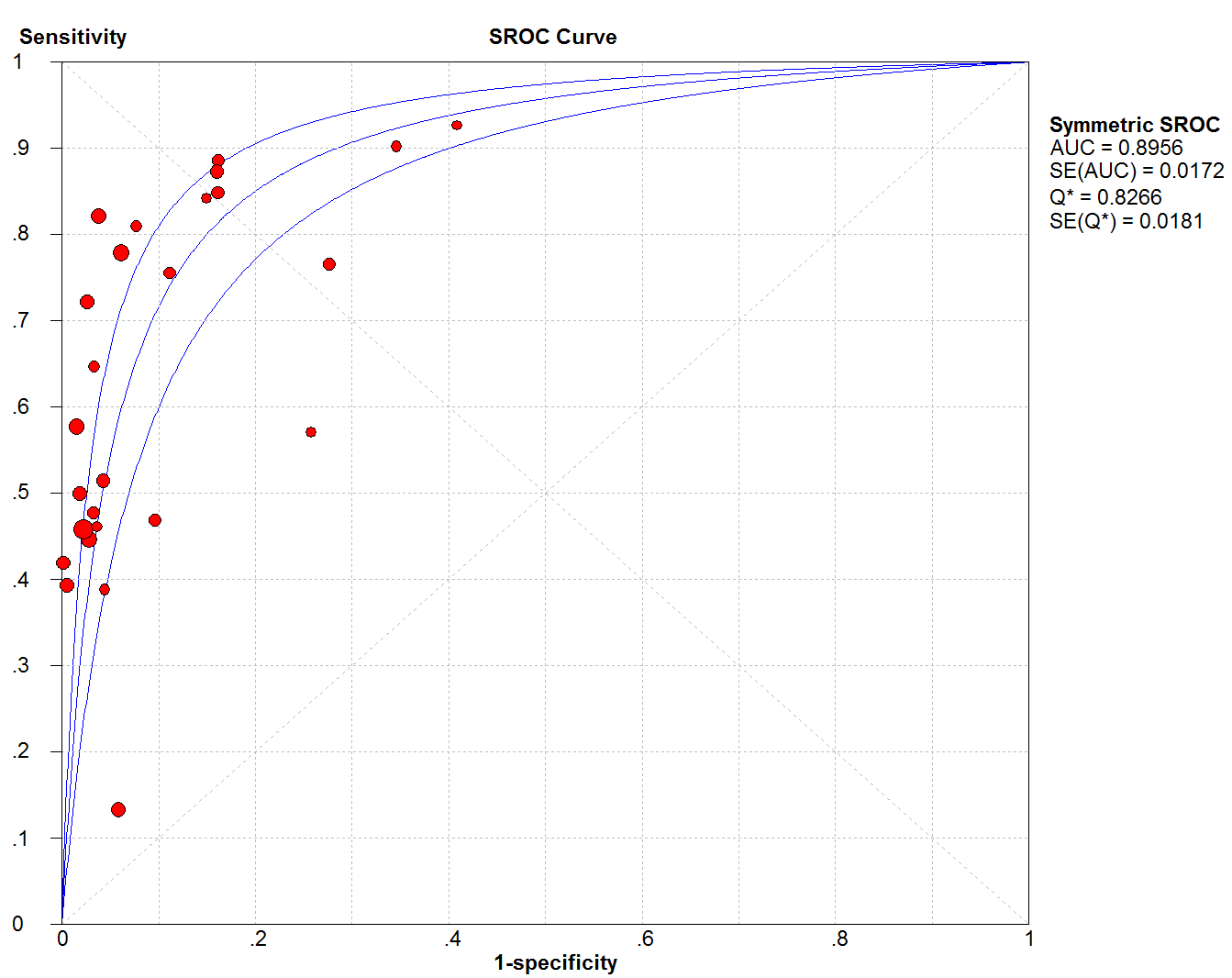

Supplement: S1 File — (ZIP) [file pone.0299045.s001.zip › statistical analysis/PET╩2╛▌/╤╟╫Θ╖╓╬÷/enrollment consecutive/sroc.bmp]

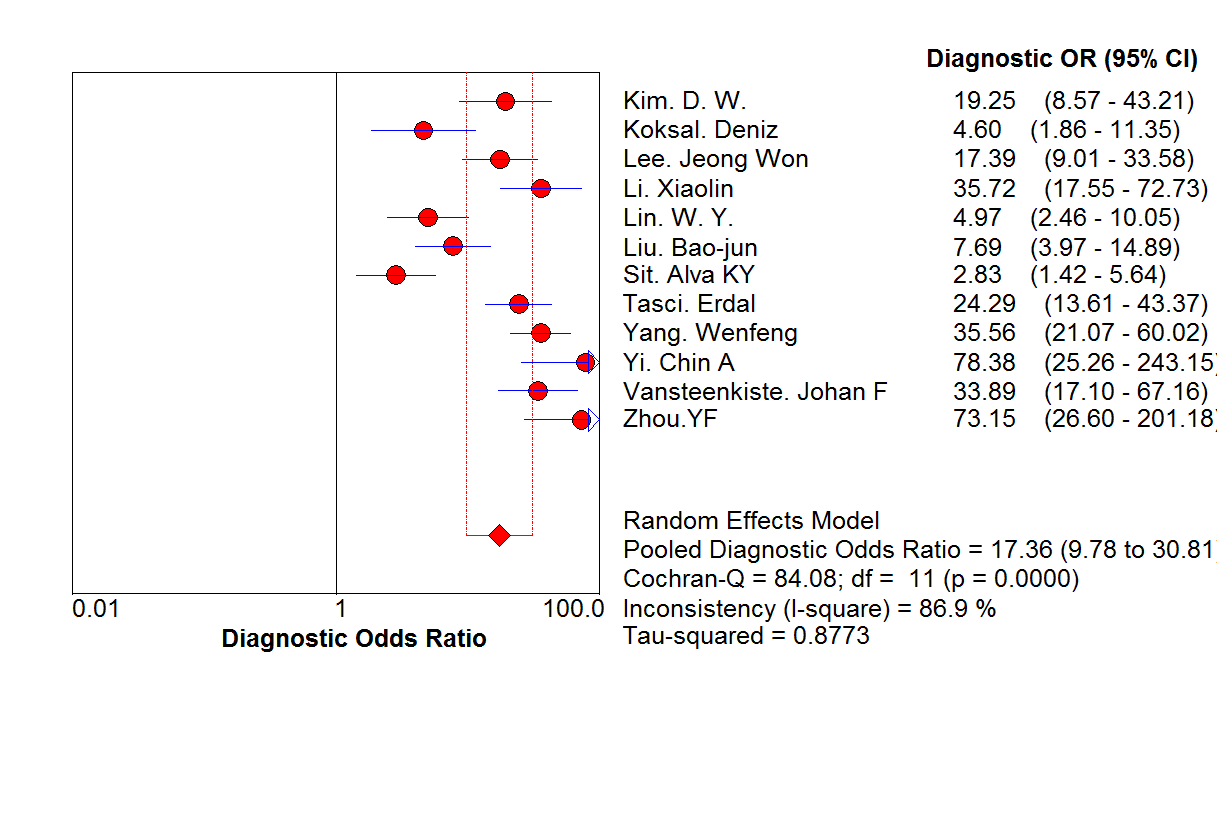

Supplement: S1 File — (ZIP) [file pone.0299045.s001.zip › statistical analysis/PET╩2╛▌/╤╟╫Θ╖╓╬÷/enrollment nonconsecutive/dor.bmp]

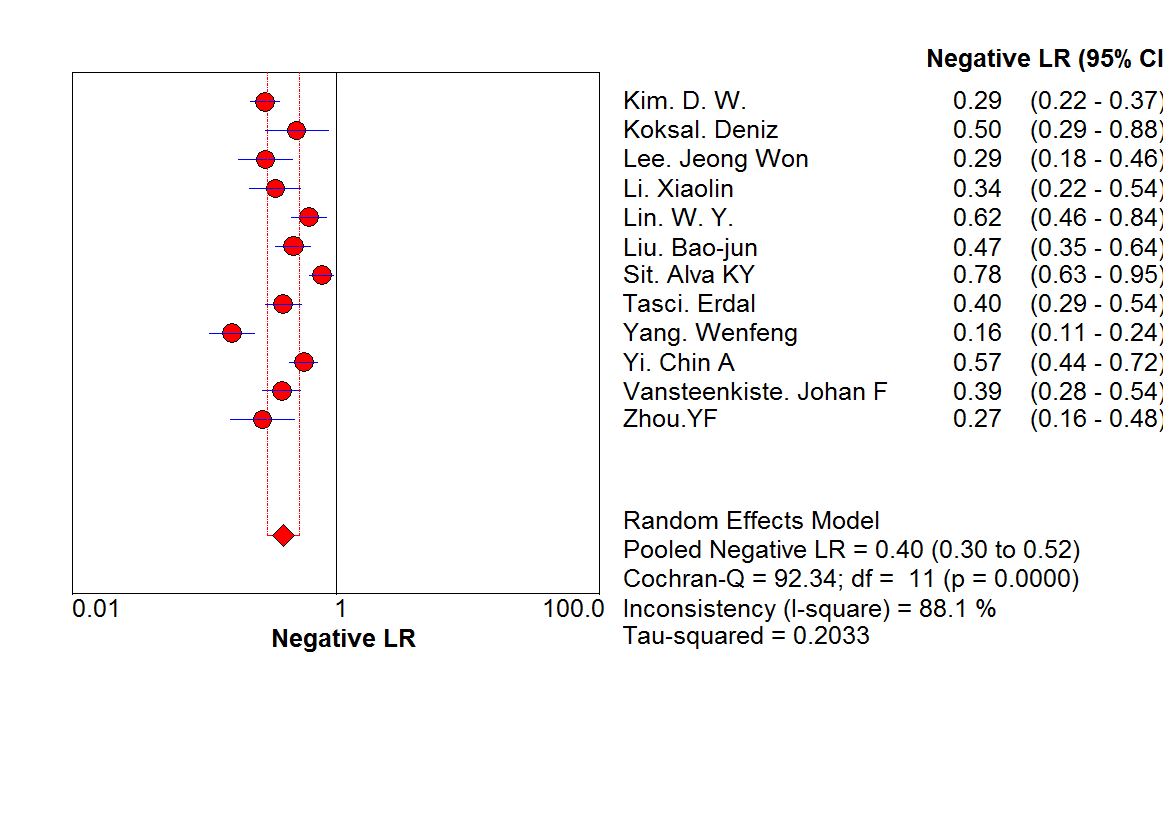

Supplement: S1 File — (ZIP) [file pone.0299045.s001.zip › statistical analysis/PET╩2╛▌/╤╟╫Θ╖╓╬÷/enrollment nonconsecutive/nlr.bmp]

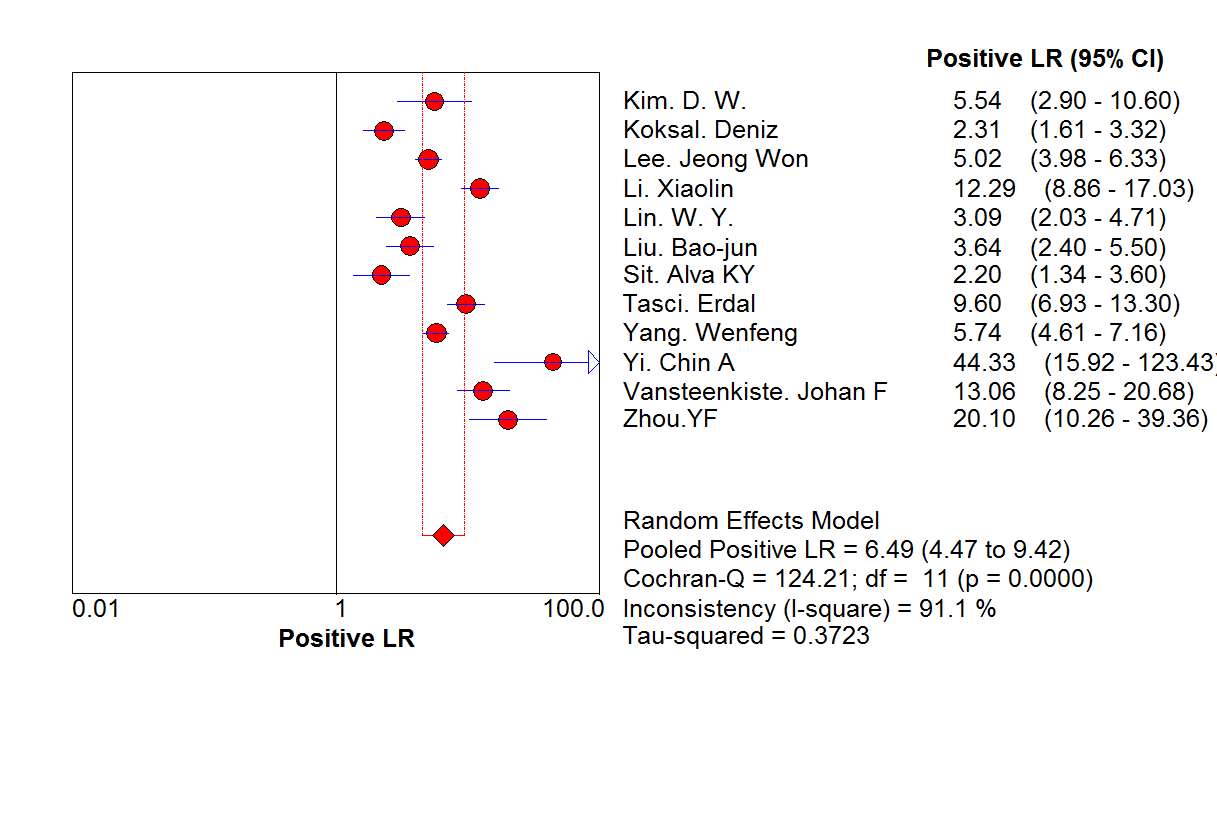

Supplement: S1 File — (ZIP) [file pone.0299045.s001.zip › statistical analysis/PET╩2╛▌/╤╟╫Θ╖╓╬÷/enrollment nonconsecutive/plr.bmp]
