# Supplementary material for: Expression of Concern: Comparison of 18F-FDG PET/CT and DWI for detection of mediastinal nodal metastasis in non-small cell lung cancer: A meta-analysis
Source: PLoS One. 2024 Feb 14;19(2):e0299045. doi: 10.1371/journal.pone.0299045 (PMC10866507; doi:10.1371/journal.pone.0299045)

Table 1. The principal characteristics of included studies.

| First author/year | Study design | Country | Consecutive | Mean age | No. of patients and lesions | Blind | Technique characteristics | TP | FP | FN | TN | Reference standard | Analysis method |
| --- | --- | --- | --- | --- | --- | --- | --- | --- | --- | --- | --- | --- | --- |
| *DWI* |  |  |  |  |  |  |  |  |  |  |  |  |  |
| Zhang/2013 | R | China | ND | 59 | 25/78 | Y | 3.0 T SE-EPI (0,800) | 29 | 13 | 6 | 30 | HP | QN |
| He/2011 | R | China | ND | 58 | 12/56 | ND | 1.5T ASSET/STIR/SE-EPI (0,500) | 18 | 4 | 16 | 18 | HP | QN |
| Usuda/2011 | P | Japan | C | 68 | 63/319 | Y | 1.5 T SS-EPI (0,800) | 33 | 3 | 11 | 272 | HP | QN |
| Zeng/2012 | R | China | ND | 58 | 45/68 | Y | 1.5 T SE-EPI (600,800,1000) | 23 | 3 | 9 | 33 | HP | QN |
| Ohno/2011 | P | Japan | C | 73 | 250/270 | Y | 1.5 T STIR-EPI (0,1000) | 101 | 17 | 34 | 118 | HP | QN |
| Nakayama/2010 | R | Japan | ND | 68 | 70/56 | Y | 1.5 T SS-SE-EPI (50,1000) | 19 | 5 | 4 | 28 | HP | QN |
| Nomori /2008 | P | Japan | C | 70 | 88/734 | Y | 1.5 T SE-EPI (0,1000) | 24 | 5 | 12 | 693 | HP | QN |
| Xu/2014 | P | China | C | 55 | 42/119 | Y | 1.5 T SS-SE-EPI (0,1000) | 29 | 7 | 6 | 77 | HP | QN |
| Usuda/2013 | P | Japan | C | 68 | 158/705 | Y | 1.5 T SS-EPI (0,800) | 39 | 5 | 22 | 639 | HP | QN |
| Kim/2012 | P | Korea | C | 62 | 49/206 | Y | 1.5 T SS-EPI (0,100,700) | 26 | 6 | 13 | 161 | HP | QN |
| *PET/CT* |  |  |  |  |  |  |  |  |  |  |  |  |  |
| Al-Sarraf, Nael/2008 | R | Ireland | C | 64.5 | 206/1145 | ND | PET-CT (Discovery ST, GE Medical systems).370MBq | 75 | 27 | 93 | 950 | HP | QN |
| An, Y. S/2008 | R | South Korea | C | 63 | 124/396 | Y | PET-CT (Discovery ST Scanner, GE Healthcare, Milwaukee, WI, USA) 370MBq | 62 | 87 | 19 | 228 | HP | QN |
| Billé, Andrea/2009 | R | Italy | C | 67 | 159/1001 | Y | PET/CT scanner (Discovery ST; GE Medical systems) 4.5-5.5 MBq/kg | 41 | 14 | 30 | 916 | HP | QL |
| Booth, K./2013 | R | England | C | 65 | 64/200 | Y | GE Discovery LS fusion PET/CT scanner 375 MBq | 7 | 8 | 11 | 174 | HP | QN/QL/ND |
| Bryant, Ayesha S/2006 | P | England | C | 67 | 143/1252 | Y | PET-CT scanner (GE Discovery LS, Milwaukee, WI). 555 MBq | 120 | 67 | 34 | 1031 | HP | QN |
| Hellwig, Dirk/2015 | R | Germany | C | 62 | 80/311 | Y | ECAT ART scanner (Siemens Medical Solutions) , 250+2 MBq | 62 | 39 | 8 | 202 | HP | QL |
| Hu, M/2008 | R | China | ND | 50 | 46/584 | ND | PET-CT scanner 7.4 MBq/kg | 117 | 72 | 17 | 378 | HP | QN |
| Jeon, Tae Yeon/2010 | R | Korea | C | 65 | 168/617 | Y | PET/CT device (Discovery LS, GE Healthcare) 370MBq | 30 | 10 | 30 | 547 | HP | QL |
| Kim, Byung-Tae/2006 | P | Korea | C | 59 | 150/568 | Y | PET/CT device (Discovery LS, GE Medical Systems) 370MBq | 23 | 0 | 32 | 513 | HP | QL |
| Kim, D. W./2012 | R | Korea | ND | 68.4 | 69/268 | ND | PET/CT (Biograph Sensation 16, Siemens Medical Systems) 4.0 MBq/kg | 157 | 8 | 52 | 51 | HP+CFU | QN |
| Kim, Yoon Kyung/2007 | P | Korea | C | 61 | 674/2477 | Y | PET/CT device (Discovery LS, GE Healthcare, Milwaukee, WI) 370 MBq | 126 | 48 | 149 | 2154 | HP | QL |
| Kim, Y. N./2012 | P | Korea | C | 62 | 49/206 | Y | PET/CT device (Discovery STE, GE Healthcare, Milwaukee, WI, USA) 370 MBq | 18 | 6 | 21 | 161 | HP | QL |
| Koksal, Deniz/2013 | R | Turkey | ND | 59.8 | 81/334 | Y | PET/CT scanner (Siemens, Biograph-6- True Point) 145 μCi/kg | 14 | 86 | 8 | 226 | HP | QL |
| Kuo, W. H./2012 | R | Taiwan | C | 63.1 | 102/118 | Y | PET/CT scanner Discovery ST16 scanner (GE Medical Systems, Milwaukee, WI), 370 to 555 MBq | 12 | 25 | 9 | 72 | HP | QL |
| Lee, A. Y./2014 | R | Korea | C | 64.5 | 104/372 | ND | PET/CT scanner (Discovery STE, GE Healthcare, Milwaukee, WI, USA), 370 MBq | 23 | 31 | 26 | 292 | HP | QN |
| Lee, Jeong Won/2009 | P | Korea | ND | 60.7 | 182/778 | ND | a Gemini PET/CT system (Philips, Milpitas). 5.18 MBq/kg | 40 | 109 | 13 | 616 | HP | QL |
| Lee, S. M./2012 | R | Korea | C | 60.0 | 160/756 | ND | Gemini PET/CT (Philips Medical Systems, Cleveland, OH, USA) 5.2 MBq/kg | 2 | 43 | 13 | 698 | HP | QN |
| Li, Meng/2012 | R | China | C | 58 | 80/265 | Y | PET–CT device (GE Discovery ST 16), 3.70–4.44 MBq/kg | 33 | 7 | 18 | 207 | HP | QN |
| Li, Xiaolin/2011 | R | China | ND | 60 | 200/1132 | ND | PET/CT scanner (GE Discovery LS, ST, or DST) 5.55–7.40 MBq/kg | 27 | 60 | 13 | 1032 | HP | QN |
| Lin, W. Y./2012 | R | Taiwan | ND | 66 | 83/364 | ND | PET-CT scanner (Discovery VCT; GE Healthcare,Waukesha, Wisconsin, USA), 370 MBq | 18 | 50 | 20 | 276 | HP | QN |
| Liu, Bao-jun/2009 | R | China | ND | 57.5 | 39/208 | Y | PET/CT scanner (Siemens Biograph Sensation 16, Siemens, Germany) 7.4MBq/kg | 40 | 24 | 26 | 120 | HP | QN/QL |
| Morikawa, Miwa/2009 | P | Japan | C | 66.1 | 93/137 | Y | PET/CT scanner (Discovery LS; GE Healthcare). 185 MBq | 74 | 19 | 8 | 36 | HP | QN |
| Nomori, H./2008 | P | Japan | C | 70 | 88/734 | ND | PET-CT device (Discovery ST; GE  Medical Systems), 3.7 MBq/kg | 26 | 18 | 10 | 680 | HP | QN |
| Ohno, Y./2007 | P | Japan | C | 68 | 115/891 | ND | PET scanner (ALLEGRO; Philips)+ CT scanner, Aquilion 16 (Toshiba Medical Systems, Ohtawara, Japan), 4.44 MBq/kg | 60 | 31 | 13 | 787 | HP | QN |
| Shim, Sung Shine/2005 | P | Korea | C | 56 | 106/393 | Y | PET/CT device (Discovery LS; GE Medical Systems, Milwaukee, Wis), 370 MBq | 28 | 58 | 5 | 302 | HP | QL |
| Sit, Alva KY/2010 | R | China | ND | 61 | 107/249 | ND | PET/CT scanner, ND | 18 | 31 | 34 | 166 | HP | QN |
| Ohno, Y./2011 | P | Japan | C | 73 | 250/270 | Y | PET/CT scanner (Discovery ST; GE Healthcare, Milwaukee, Wis). 3.3 MBq/kg | 102 | 15 | 33 | 120 | HP | QN |
| Tasci, Erdal/2010 | R | Turkey | ND | 58.2 | 127/826 | ND | on a Biograph PET/CT (Siemens/CTI) scanner, 555MBq | 41 | 50 | 24 | 711 | HP | QL |
| Toba, H./2010 | R | Japan | C | 68.0 | 42/217 | ND | PET/CT scanner Aquiduo (Toshiba Medical Systems, Tokyo, Japan) | 17 | 15 | 4 | 181 | HP | QL |
| Tournoy, KG/2007 | P | Belgium | C | 68 | 52/105 | Y | FDG-PET/CT scanner (Philips Gemini FDG-PET/CT, Philips Medical Systems, Cleveland, Ohio, USA), 4 MBq/kg | 32 | 10 | 6 | 57 | HP | QN |
| Usuda, Katsuo/2013 | P | Japan | C | 68 | 158/705 | Y | PET-CT (SIEMENS Biography Sensation 16, Erlangenm Germany), 3.7 MBq/Kg | 24 | 3 | 37 | 641 | HP | QN |
| Ventura, Elisa/2010 | R | USA | C | 66.32 | 31/90 | Y | PET (CTI Molecular Imaging, Knoxville, TN, USA)+PET/CT Siemens Molecular Imaging, Knoxville, TN, USA), 555-740MBq | 38 | 20 | 3 | 29 | HP | QL |
| Xu, N/2014 | R | China | C | 61 | 101/528 | Y | PET/CT scanner, 4.5-5.5 MBq/kg | 52 | 18 | 49 | 409 | HP | QL |
| Usuda, Katsuo/2011 | P | Japan | C | 68 | 63/319 | Y | PET/CT scanner (Siemens Biography Sensation 16), 185 MBq | 21 | 9 | 23 | 266 | HP | QN |
| Yang, Wenfeng/2009 | P | China | ND | 69 | 122/639 | Y | PET/CT system (Discovery LS; GE Healthcare), 370 MBq | 132 | 73 | 21 | 413 | HP | QL |
| Yi, Chin A/2007 | R | Korea | N | 60 | 143/453 | Y | PET/CT device (Discovery LS, GE Healthcare), 370 MBq | 22 | 4 | 28 | 399 | HP | QN |
| Vansteenkiste, Johan F/1998 | P | Belgium | ND | 62 | 56/493 | Y | PET scanner (CTI-Siemens 931/08/12), 6.5 MBq/kg | 38 | 21 | 22 | 412 | HP | QL |
| Zhou,YF/2014 | R | China | ND | 60 | 64/280 | ND | PET/CT scanner (Philips Gemini TF 16), 2.96MBq/kg | 25 | 9 | 9 | 237 | HP | QN/QL |

ND: no documented; No.: number; TP: true positive; FP: false positive; FN: false negative; TN: true negative. P: prospective; R: retrospective; Y: yes; QL: qualitative analysis; QN: quantitative analysis; HP: histopathology; C: consecutive

Table 2. The principal characteristics of included studies.

| First author/year | No. of patients and lesions | TP | FP | FN | TN | Data extraction |
| --- | --- | --- | --- | --- | --- | --- |
| *DWI* |  |  |  |  |  |  |
| Zhang/2013 | 25/78 | 29 | 13 | 6 | 30 | Calculated |
| He/2011 | 12/56 | 18 | 4 | 16 | 18 | Calculated |
| Usuda/2011 | 63/319 | 33 | 3 | 11 | 272 | Directly extracted |
| Zeng/2012 | 45/68 | 23 | 3 | 9 | 33 | Calculated |
| Ohno/2011 | 250/270 | 101 | 17 | 34 | 118 | Directly extracted |
| Nakayama/2010 | 70/56 | 19 | 5 | 4 | 28 | Directly extracted |
| Nomori /2008 | 88/734 | 24 | 5 | 12 | 693 | Directly extracted |
| Xu/2014 | 42/119 | 29 | 7 | 6 | 77 | Calculated |
| Usuda/2013 | 158/705 | 39 | 5 | 22 | 639 | Directly extracted |
| Kim/2012 | 49/206 | 26 | 6 | 13 | 161 | Calculated |
| *PET/CT* |  |  |  |  |  |  |
| Al-Sarraf, Nael/2008 | 206/1145 | 75 | 27 | 93 | 950 | Calculated |
| An, Y. S/2008 | 124/396 | 62 | 87 | 19 | 228 | Calculated |
| Billé, Andrea/2009 | 159/1001 | 41 | 14 | 30 | 916 | Directly extracted |
| Booth, K./2013 | 64/200 | 7 | 8 | 11 | 174 | Directly extracted |
| Bryant, Ayesha S/2006 | 143/1252 | 120 | 67 | 34 | 1031 | Calculated |
| Hellwig, Dirk/2015 | 80/311 | 62 | 39 | 8 | 202 | Directly extracted |
| Hu, M/2008 | 46/584 | 117 | 72 | 17 | 378 | Calculated |
| Jeon, Tae Yeon/2010 | 168/617 | 30 | 10 | 30 | 547 | Directly extracted |
| Kim, Byung-Tae/2006 | 150/568 | 23 | 0 | 32 | 513 | Directly extracted |
| Kim, D. W./2012 | 69/268 | 157 | 8 | 52 | 51 | Calculated |
| Kim, Yoon Kyung/2007 | 674/2477 | 126 | 48 | 149 | 2154 | Directly extracted |
| Kim, Y. N./2012 | 49/206 | 18 | 6 | 21 | 161 | Directly extracted |
| Koksal, Deniz/2013 | 81/334 | 14 | 86 | 8 | 226 | Directly extracted |
| Kuo, W. H./2012 | 102/118 | 12 | 25 | 9 | 72 | Calculated |
| Lee, A. Y./2014 | 104/372 | 23 | 31 | 26 | 292 | Calculated |
| Lee, Jeong Won/2009 | 182/778 | 40 | 109 | 13 | 616 | Directly extracted |
| Lee, S. M./2012 | 160/756 | 2 | 43 | 13 | 698 | Directly extracted |
| Li, Meng/2012 | 80/265 | 33 | 7 | 18 | 207 | Directly extracted |
| Li, Xiaolin/2011 | 200/1132 | 27 | 60 | 13 | 1032 | Directly extracted |
| Lin, W. Y./2012 | 83/364 | 18 | 50 | 20 | 276 | Directly extracted |
| Liu, Bao-jun/2009 | 39/208 | 40 | 24 | 26 | 120 | Calculated |
| Morikawa, Miwa/2009 | 93/137 | 74 | 19 | 8 | 36 | Directly extracted |
| Nomori, H./2008 | 88/734 | 26 | 18 | 10 | 680 | Directly extracted |
| Ohno, Y./2007 | 115/891 | 60 | 31 | 13 | 787 | Directly extracted |
| Shim, Sung Shine/2005 | 106/393 | 28 | 58 | 5 | 302 | Directly extracted |
| Sit, Alva KY/2010 | 107/249 | 18 | 31 | 34 | 166 | Calculated |
| Ohno, Y./2011 | 250/270 | 102 | 15 | 33 | 120 | Directly extracted |
| Tasci, Erdal/2010 | 127/826 | 41 | 50 | 24 | 711 | Directly extracted |
| Toba, H./2010 | 42/217 | 17 | 15 | 4 | 181 | Directly extracted |
| Tournoy, KG/2007 | 52/105 | 32 | 10 | 6 | 57 | Calculated |
| Usuda, Katsuo/2013 | 158/705 | 24 | 3 | 37 | 641 | Directly extracted |
| Ventura, Elisa/2010 | 31/90 | 38 | 20 | 3 | 29 | Calculated |
| Xu, N/2014 | 101/528 | 52 | 18 | 49 | 409 | Calculated |
| Usuda, Katsuo/2011 | 63/319 | 21 | 9 | 23 | 266 | Directly extracted |
| Yang, Wenfeng/2009 | 122/639 | 132 | 73 | 21 | 413 | Directly extracted |
| Yi, Chin A/2007 | 143/453 | 22 | 4 | 28 | 399 | Directly extracted |
| Vansteenkiste, Johan F/1998 | 56/493 | 38 | 21 | 22 | 412 | Calculated |
| Zhou,YF/2014 | 64/280 | 25 | 9 | 9 | 237 | Directly extracted |

TP: true positive; FP: false positive; FN: false negative; TN: true negative.

These figures showed pooled results based on either 10 or 9 DWI studies using Metadisc software, consisting of pooled sensitivity, specificity, positive and negative LR, and diagnostic OR.


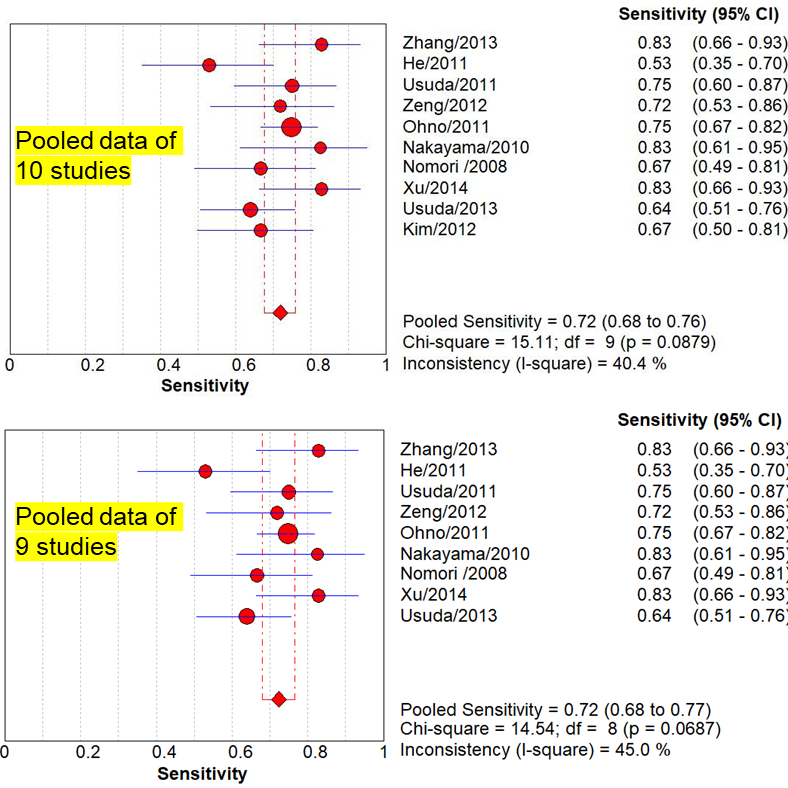


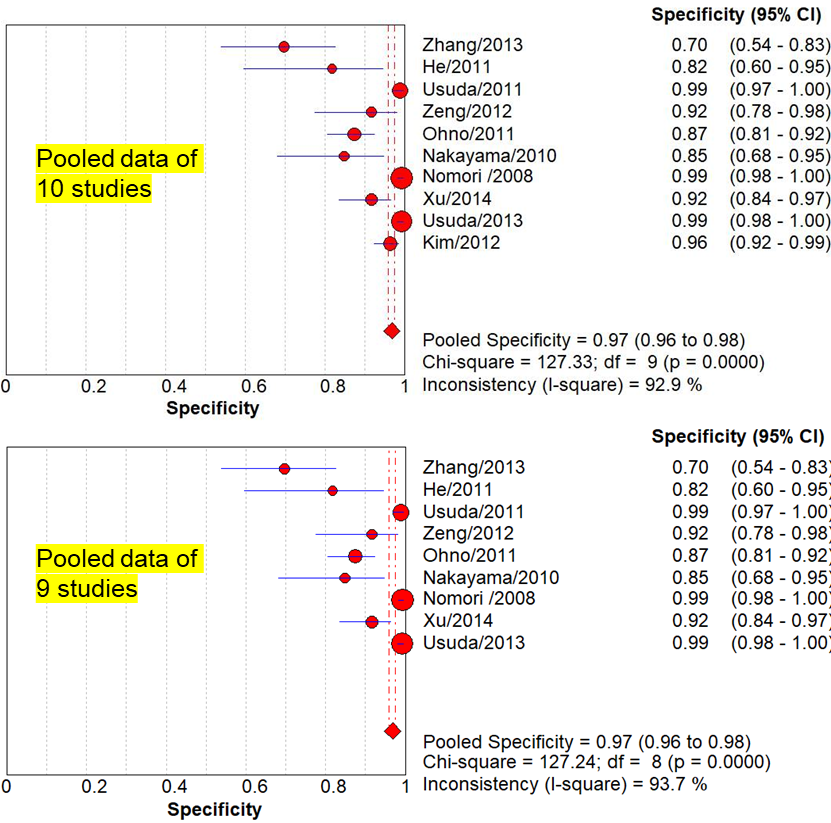


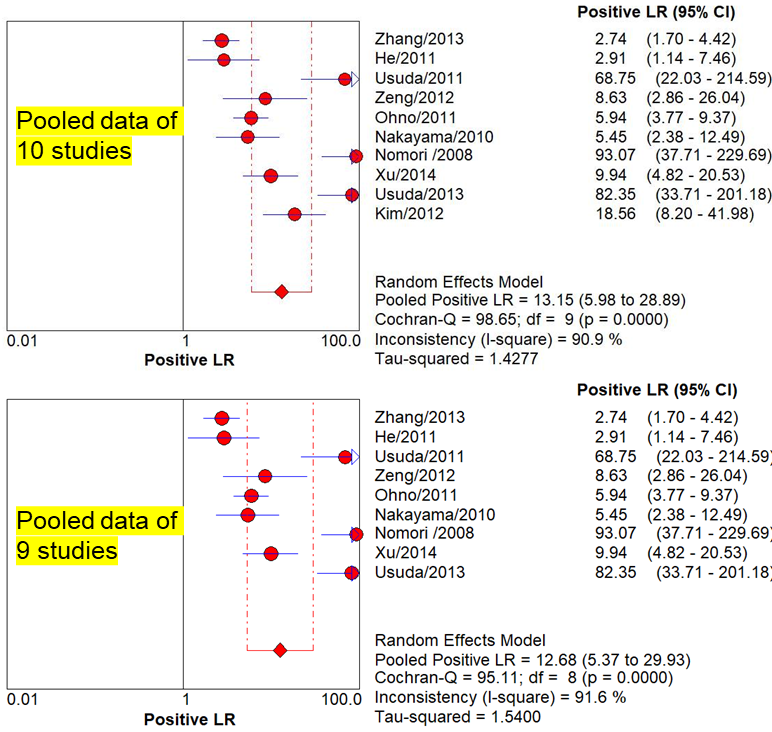


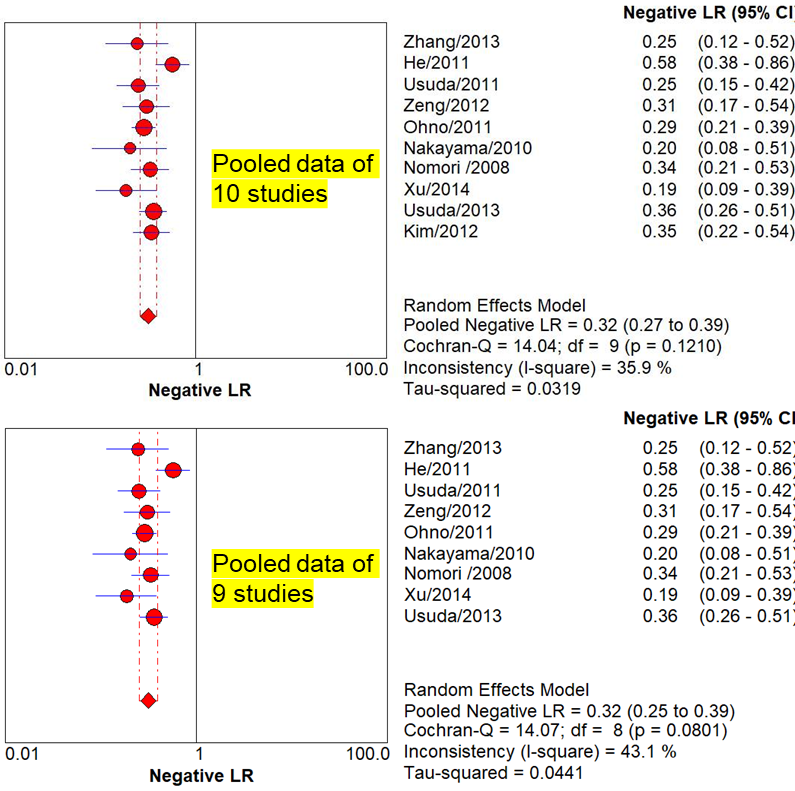


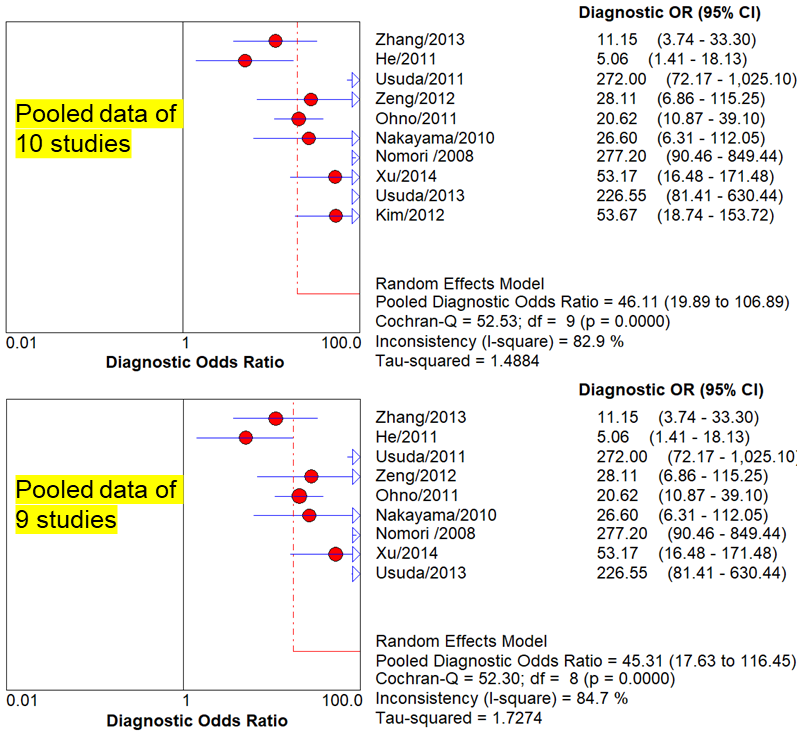


These two figures were SROC curves of DWI and PET obtained by Stata with the midas command.


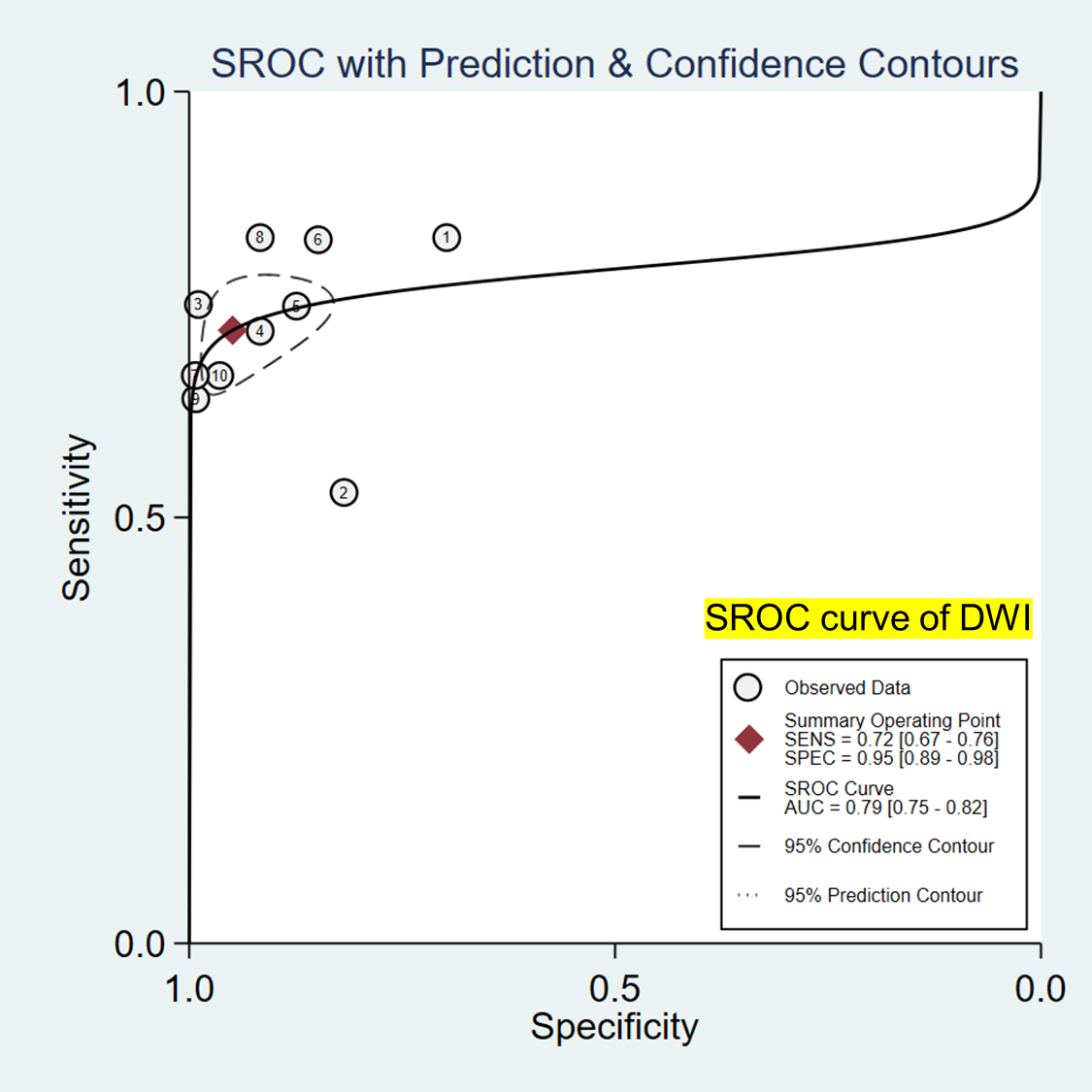


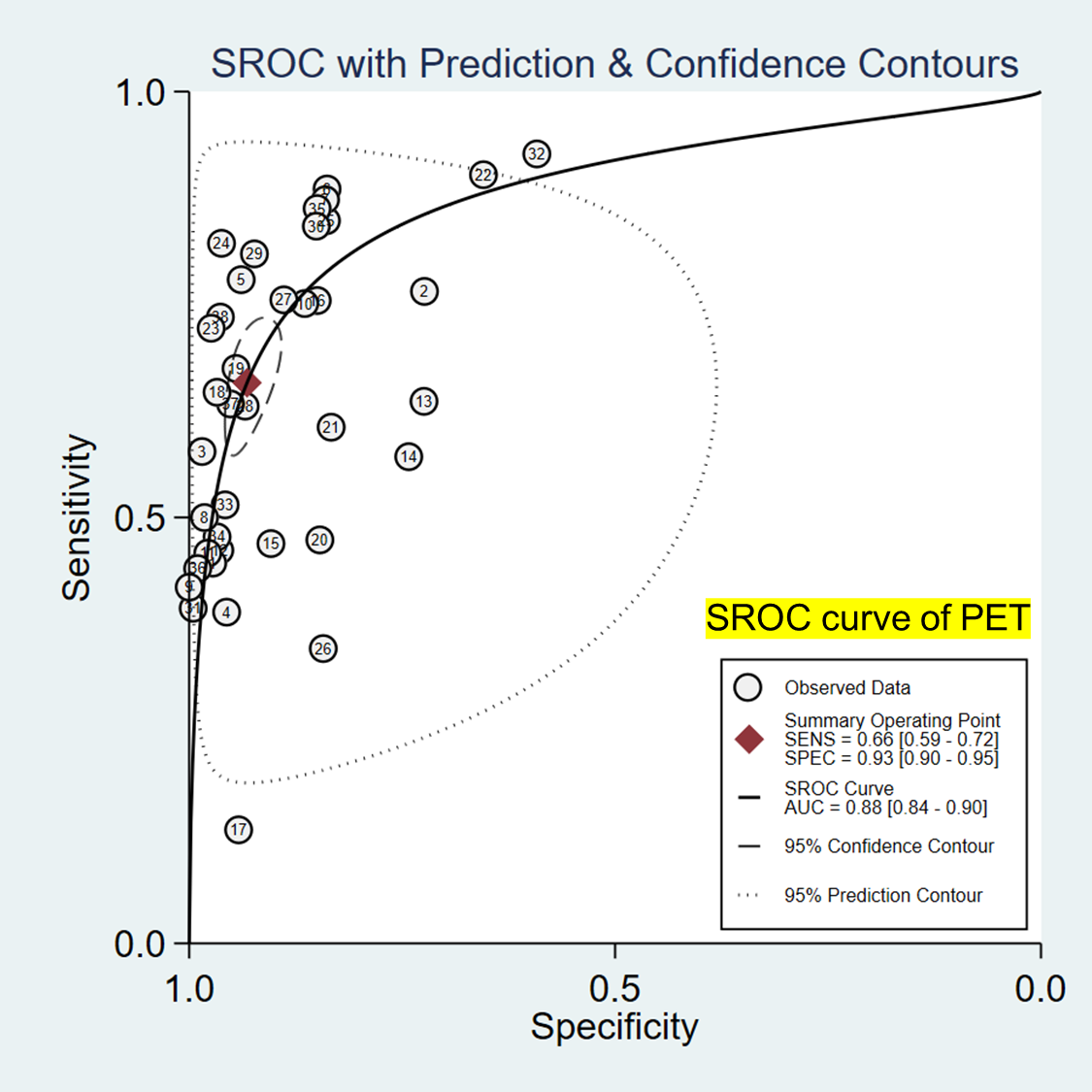

Supplement: S2 File — (DOCX) [file pone.0299045.s002.docx]
